# Thiocarbazate building blocks enable the construction of azapeptides for rapid development of therapeutic candidates

**DOI:** 10.1038/s41467-022-34712-9

**Published:** 2022-11-28

**Authors:** Ahmad Altiti, Mingzhu He, Sonya VanPatten, Kai Fan Cheng, Umair Ahmed, Pui Yan Chiu, Ibrahim T. Mughrabi, Bayan Al Jabari, Ronald M. Burch, Kirk R. Manogue, Kevin J. Tracey, Betty Diamond, Christine N. Metz, Huan Yang, LaQueta K. Hudson, Stavros Zanos, Myoungsun Son, Barbara Sherry, Thomas R. Coleman, Yousef Al-Abed

**Affiliations:** 1 Institute of Bioelectronic Medicine, Feinstein Institutes for Medical Research, Northwell Health, Manhasset, NY USA; 2 Institute of Molecular Medicine, Feinstein Institutes for Medical Research, Manhasset, NY USA; 3 Applied Immunotherapeutics, Inc, Morris, CT USA; 4 Center for Molecular Innovation, Feinstein Institutes for Medical Research, Manhasset, NY USA; 5 Donald and Barbara Zucker School of Medicine at Hofstra/Northwell, Hempstead, NY USA

**Keywords:** Solid-phase synthesis, Biomimetic synthesis, Peptides

## Abstract

Peptides, polymers of amino acids, comprise a vital and expanding therapeutic approach. Their rapid degradation by proteases, however, represents a major limitation to their therapeutic utility and chemical modifications to native peptides have been employed to mitigate this weakness. Herein, we describe functionalized thiocarbazate scaffolds as precursors of aza-amino acids, that, upon activation, can be integrated in a peptide sequence to generate azapeptides using conventional peptide synthetic methods. This methodology facilitates peptide editing—replacing targeted amino acid(s) with aza-amino acid(s) within a peptide—to form azapeptides with preferred therapeutic characteristics (extending half-life/bioavailability, while at the same time typically preserving structural features and biological activities). We demonstrate the convenience of this azapeptide synthesis platform in two well-studied peptides with short half-lives: FSSE/P5779, a tetrapeptide inhibitor of HMGB1/MD-2/TLR4 complex formation, and bradykinin, a nine-residue vasoactive peptide. This bench-stable thiocarbazate platform offers a robust and universal approach to optimize peptide-based therapeutics.

## Abstract (web summary)

The rapid protease degradation of peptides is currently limiting their therapeutic utility. Here, the authors report functionalised thiocarbazate scaffolds as precursors of aza-amino acids that can be integrated in peptide sequences, extending their bioavailability, and demonstrate this on FSSE/P5779 and bradykinin.

## Introduction

Peptides are short sections of proteins, made up of amino acids—the functional building blocks of life. Therapeutic peptides typically comprise one or more functional domain(s) of the parent protein, and, as such, are typically (1) easier to produce than full length proteins, (2) more selective in activity, and (3) less immunogenic^1^. Peptides have a myriad of potential functions and are used clinically to treat multiple diseases and conditions^2^. One frequent drawback, however, is their short half-lives (seconds to minutes), due to rapid catabolism by proteases and peptidases in the gastrointestinal track and bloodstream, which limits their overall effectiveness and route of administration^3^. Thus, with a very short half-life, the peptide has a minimal therapeutic window and must be dosed multiple times a day. Alterations of native peptides have been employed to temper this intrinsic instability and generate peptidomimetics. One class of peptidomimetics, the azapeptides, is very appealing in that aza-amino acids—surrogates of natural amino acids wherein the amino acid α-carbon has been substituted by a nitrogen atom—can be incorporated by replacing specific residues within a peptide sequence^4,5^. Through selective aza-amino acid substitution(s) of amino acids that reside at protease-sensitive cleavage sites, one can create azapeptide derivatives that possess preferred characteristics—namely extended bioavailability coupled with retained peptide structure—resulting in maximum biological efficacy^6–10^. Atazanavir^11^, an HIV protease inhibitor, and Goserelin^12^, a luteinizing hormone releasing hormone analogue used in breast and prostate cancer are two well-known FDA-approved azapeptides, and underscore the potential of this class of therapeutics. These successful examples of azapeptide-based peptidomimetics that have advanced into clinical service exemplify the importance of new synthetic methods to prepare these valuable targets.

Historically, azapeptides have been considered as a synthetically challenging class of peptidomimetics despite efforts in development and refinement of preparation methods, but to date, a convenient, universal, and robust synthetic platform remains an unmet goal. In general, aza-amino acids are synthesized by activating either the hydrazine moiety or the N-terminal amine of peptides with carbonyl donating reagents^6,7,13,14^, such as chloroformates^15–20^, 2,4-dinitrophenyl formate^21–23^, carbonyldiimidazole^24^, carbonyltriazole^25^, perfluorophenylformate^26,27^, N-hydroxy-succinimidyl formate^28^ and N-hydroxy-benzotriazolyl carbamate^29,30^. Among these, phosgene-based carbamoyl chlorides are the most common carbonyl donating sources, but they are beset with toxicity and stability problems, which make them unappealing as carbonyl donating reagents^15,17,20^. Recently, the functionalization of Schiff base peptide-bound azaGly residues represented a direct route to regio-selectively alkylate the peptidyl chain using simple alkyl halides^31^, Michael addition^32^ or Mitsunobu reaction^33^. These methods established and enabled the field of azapeptides, however, these protocols are not ideal for building libraries because of the reagents and harsh conditions that are not fully compatible with solid-phase peptide synthesis (SPPS).

**Fig. 1:**
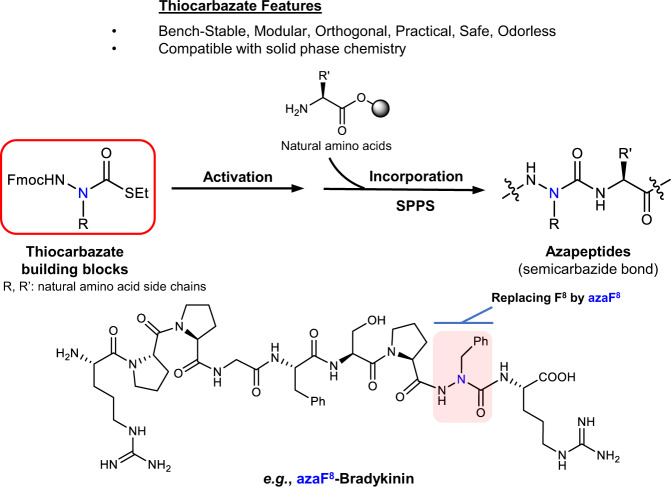
Overview of thiocarbazate-directed azapeptide synthesis platform and applications. Thiocarbazate building blocks can be incorporated into a peptide sequence using solid-phase peptide synthesis (SPPS) in a rapid and practical manner to construct libraries of azapeptide-substituted candidates for biological screening. Phenyl (abbr. Ph) = −C_6_H_5_.

Herein, we identify thiocarbazate building blocks as stable precursors of carbonyl donating reagents. We also develop a method to activate these thiocarbazates for coupling and establish protocols to incorporate these activated aza-amino acids using solution-phase and standard SPPS methods (Fig.1). To demonstrate the therapeutic potential of our technology, we employ two distinct peptides, FSSE (aka P5779) and bradykinin, that have been well-characterized in the literature. Using our methodology, we edit individual amino acids in both peptides using an aza-scan methodology, whereby a small library of aza-amino acid-substituted peptide analogues is strategically engineered for subsequent biological testing. These studies validate our azapeptide synthesis platform as a robust and convenient tool to rapidly generate libraries of azapeptides that can be used to probe the structural characteristics, bioavailability, and functional activities of potential therapeutic peptides. This advancement will promote azapeptides as a relevant therapeutic option in numerous disease conditions.

## Results and discussion

### S-Ethyl thiocarbazate building blocks for targeted aza-amino acid substitutions (aza-scan): synthesis, activation and coupling

**Fig. 2:**
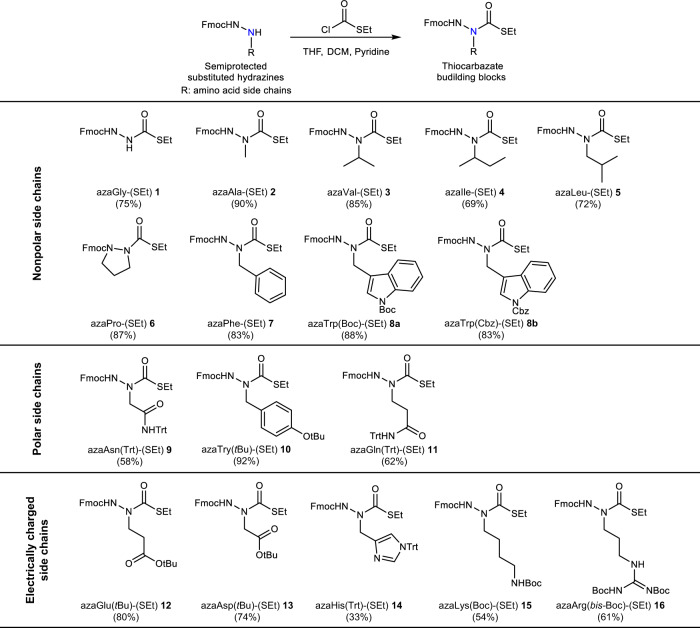
Synthesis of S-ethyl thiocarbazate as bench-stable and latent aza-amino acid surrogates with natural amino acid side chains. Sixteen Fluorenylmethyloxycarbonyl (Fmoc)-protected thiocarbazate amino acid building blocks are outlined. Synthesis and characterization of these aza-amino acid conjugates are detailed in Supplementary Information.

Recognizing that the acylating agent of an amino acid surrogate is a key intermediate in building azapeptides, we wished to identify a stable precursor that could be easily activated. We hypothesized that a precursor carrying a thiolate functional group could serve the purpose. Assembly of an amino acid surrogate with a thiolate functionality yielded the building block S-ethyl thiocarbazate (Fig. 1). First, sixteen natural amino acid surrogates as S-ethyl thiocarbazate derivatives were synthesized from known hydrazines via standard condensation, reduction, and subsequent coupling with S-ethyl chlorothioformate in good to excellent yields (Fig. 2, Supplementary Table 1 and Supplementary Information)^17,34^. Of the remaining four natural amino acids, serine, threonine, methionine and cysteine are incompatible with our activation protocol or inherently unstable aza-amino acid residues^17^.

While previous methods to activate thioester-containing compounds utilized harsh or impractical reaction conditions^35–40^, our strategy of treatment of the S-ethyl thiocarbazates^41^ with N-chloroimides produced the desired acylating agents under relatively gentle reaction conditions. Indeed, robust activation was achieved within 5 min at room temperature by pretreatment of S-ethyl thiocarbazate with tetrabutylammonium chloride (TBACl) followed by trichloroisocyanuric acid (TCCA) in dichloromethane (DCM), forming a reactive acyl chloride. To study the alkylthiocarbazates as latent aza-amino acid surrogates and to evaluate their potential in routine azapeptide syntheses, the reactivity of the acylating agent was probed by coupling under solution-phase and solid-phase peptide synthesis (SPPS).

### Solution phase azapeptide synthesis

As a model reaction, optimized activation conditions of azaPhe thiocarbazate (**7**) was achieved by treatment with 0.85 equivalent of TCCA and 1.5 equivalent of TBACl at 0 °C to produce the acylium intermediate which was characterized as Fmoc-azaPhe acyl chloride (**17**) (Table 1, Supplementary Table 4). The structure of compound **17** was confirmed by X-ray analysis (Table 1, Supplementary Fig. 1a, CCDC-2195262). Without purification, reactive acyl chloride **17** successfully coupled with *tert* butyl *L*-valinate, and the azadipeptide **18** was furnished in 88.5-93.7% yield (Table 1, entry 1, Supplementary Table 4,entry 8). Of note, Fmoc-chlorinated byproduct was observed at 1.4% yield (Supplementary Table 4, Supplementary Fig. 1). Next, we assessed the activation and coupling of the remaining thiocarbazates (Table 1). Most thiocarbazates performed well: the resulting azadipeptides were isolated in 61.0–95.0% yields and characterized by HPLC/HRMS and NMR spectroscopy (see Supplementary Information). Only Fmoc-aza-tryptophan (Table 1, entry 9 &10) and Fmoc-aza-histidine (Table 1, entry 11) did not work well (Table 1, protocol A) due to the chlorination on the reactive aromatic moieties in their side chains. To overcome this obstacle, the activation protocol was adjusted by reducing the stoichiometry of the TCCA to 0.39 equivalent and the reaction temperature to −10 °C (Table 1, protocol B, Supplementary information). Eventually, azadipeptides **26**, **27**, and **28** were collected in 43.0%, 41.0%, and 27.0% yields respectively in addition to some chlorinated side products and recovered starting materials. Lastly, the lability of indolyl residue in azaTrp **8a** under acidic conditions^17^ has been overcome by replacing the side chain protecting group N-Boc **8a** with N-Cbz **8b**. Deprotection of Cbz in the azadipeptide **27** was achieved by treating with Et_3_SiH/Pd black reduction system and the deprotected azadipeptide Fmoc-azaWV(OtBu) was collected in 71% yield (see Supplementary information). This example proves that peptides that comprise sensitive aza-amino acid residues like aza-tryptophan and aza-tyrosine can be accessed easily under mild and neutral conditions using both solution-phase and solid-phase chemistries, vide infra.

**Table 1:** General and modified protocols for thiocarbazates activation and coupling in solution-phase chemistry


Entry	Sequence	aza dipeptides	Protocol	Isolated yield	HPLC purity (254 nm)	HRMS cal. Na^+^	HRMS obs. Na^+^
1	Fmoc-azaFV(OtBu)	**18**	A	93.7%	98.3%	566.2625	566.2627
2	Fmoc-azaGV(OtBu)	**19**	A	70.6%	95.6%	476.2156	476.2158
3	Fmoc-azaAV(OtBu)	**20**	A	76.6%	99.8%	490.2312	490.2312
4	Fmoc-azaVV(OtBu)	**21**	A	80.0%	97.4%	518.2625	518.2628
5	Fmoc-azaLV(OtBu)	**22**	A	94.0%	94.5%	532.2782	532.2785
6	Fmoc-azaIV(OtBu)	**23**	A	95.0%	99.5%	532.2782	532.2785
7	Fmoc-azaPV(OtBu)	**24**	A	73.6%	97.8%	516.2469	516.2471
8	Fmoc-azaY(tBu)V(OtBu)	**25**	A	79.0%	94.3%	638.3201	638.3202
9	Fmoc-azaW(Boc)V(OtBu)	**26**	B	42.0% (67.0%)*	97.5%	705.3259	705.3257
10	Fmoc-azaW(Cbz)V(OtBu)	**27**	B	41.0% (63.0%)*	95.6%	739.3102	739.3106
11	Fmoc-azaH(Trt)V(OtBu)	**28**	B	27.0%	91.3%	798.3626	798.3636
12	Fmoc-azaD(tBu)V(OtBu)	**29**	A	80.5%	98.8%	590.2837	590.2840
13	Fmoc-azaE(tBu)V(OtBu)	**30**	A	82.7%	96.8%	604.2993	604.2996
14	Fmoc-azaK(Boc)V(OtBu)	**31**	A	61.0%	95.9%	647.3415	647.3439
15	Fmoc-azaR(*bis*-Boc)V(OtBu)	**32**	A	63.0%	98.8%	775.4001	775.4009
16	Fmoc-azaN(Trt)V(OtBu)	**33**	A	84.0%	99.3%	775.3466	775.3472
17	Fmoc-azaQ(Trt)V(OtBu)	**34**	A	53.0%	99.6%	789.3623	789.3623

Reaction conditions: protocol A-The activation step is carried out at 0 °C for 10 min, 1 eq. of corresponding thiocarbazate, 0.85 eq. of TCCA, 1.5 eq. of TBACl; followed by in situ coupling with 2.1 eq. of t-Bu *L*-valinate and 4.4 eq of DIPEA at 0 °C; protocol B-The activation step is carried out at -10 °C for 10 min, 1 eq. of corresponding thiocarbazate, 0.39 eq. of TCCA, 1.5 eq of TBACl; followed by in situ coupling with 2.1 eq of t-Bu *L*-valinate and 4.4 eq of DIPEA with gradual warm up to room temperature. DCM was used as solvent in both protocols and the reaction was monitored by TLC. Inset: X-ray analysis of the anticipated aza-amino acyl chloride intermediate **17** shown. *Yields between parenthesis are reported based on recovered starting material (b.r.s.m). Synthesis and characterization of these azadipeptides are detailed in Supplementary Information.

### Solid-Phase Azapeptide Synthesis

Armed with successful protocols to build and utilize thiocarbazates in solution phase synthesis, we focused on exploring the potential of these scaffolds in SPPS. Table 2 displays a representative SPPS cycle to integrate an aza-amino acid residue into a peptide sequence. In our experimental design, the syntheses were conducted on 0.1 mmol of amino acid loaded into a Wang or Rink Amide resin. Each SPPS cycle consists of the following steps: (1) swelling, (2) Fmoc deprotection (20% piperidine in DMF), (3) successive washes (DMF and DCM), and (4) peptide bond formation (using the activated thiocarbazate platform). The activation of the thiocarbazates, as depicted in Table 2, was run at ambient temperature. However, cold activation is recommended to improve performance and accommodate reactive side-chain residues. We utilized 5.0 equivalents of each thiocarbazate (0.1 mmole) relative to the bound resin with 7.5 equivalents of TBACl in DCM which was treated with 5.0 equivalents of TCCA before it was agitated for 5 min using a vortex. The mixture was then centrifuged, and the clear supernatant was added to the primed resin with N-methyl morpholine (NMM) as a non-nucleophilic base. To obtain optimal conversion rates, we typically coupled for 16 h. However, our data showed that >65% of the primed N-peptidyl chain is functionalized with aza-amino acid residue within 20 min (see Supplementary information). All sixteen thiocarbazates were coupled to phenylalanine bound to Wang resin (Table 2). The conversion rates were assessed after resin cleavage, based on the integration of the unreacted L-phenylalanine as detected in the HPLC analysis at 215 nm, excellent conversions (82.1–100%) were achieved for all thiocarbazates (Table 2). The resulting azadipeptides **35**-**50** were successfully collected in 56.0–93.7% crude purity. In sum, we have established conditions wherein thiocarbazates function well as azapeptide precursors using convenient solid phase synthesis.

**Table 2:** Schematic protocol to activate and utilize thiocarbazates in solid phase synthesis manifested in the delivery of a library of azadipeptides


Entry	Sequence	aza dipeptides	Conversion rate	RT (min)	Crude purity (254 nm)	HRMS cal. H^+^	HRMS obs. H^+^
1	Fmoc-azaFF-OH	**35**	91.1%	11.50	89.8%	536.2180	536.2182
2	Fmoc-azaVF-OH	**36**	82.6%	10.89	82.6%	488.2180	488.2186
3	Fmoc-azaIF-OH	**37**	100%	11.20	87.0%	502.2336	502.2338
4	Fmoc-azaLF-OH	**38**	96.7%	11.42	92.5%	502.2336	502.2339
5	Fmoc-azaGF-OH	**39**	90.5%	9.87	71.8%	446.1710	446.1712
6	Fmoc-azaAF-OH	**40**	82.4%	10.28	81.7%	460.1867	460.1870
7	Fmoc-azaPF-OH	**41**	87.4%	11.39	82.1%	486.2023	486.2027
8	Fmoc-azaNF-OH	**42**	90.2%	9.45	71.1%	503.1925	503.1931
9	Fmoc-azaYF-OH^*^	**43**	93.4%	10.42	84.0%	552.2129	552.2134
10	Fmoc-azaQF-OH	**44**	82.1%	9.37	86.0%	517.2082	517.2099
11	Fmoc-azaDF-OH	**45**	93.0%	9.81	93.7%	504.1765	504.1768
12	Fmoc-azaEF-OH	**46**	95.6%	9.75	85.2%	518.1922	518.1926
13	Fmoc-azaHF-OH	**47**	92.9%	8.25	74.4%	526.2085	526.2087
14	Fmoc-azaKF-OH	**48**	96.7%	8.00	89.6%	517.2445	517.2449
15	Fmoc-azaRF-OH	**49**	91.2%	8.24	90.3%	545.2507	545.2506
16	Fmoc-azaW(Cbz)F-OH^#^	**50**	86.8%	14.94	56.0%	709.2657	709.2661

Synthesis and characterization of these azadipeptides are detailed in Supplementary Information. *Fmoc-azaYF-OH (entry 9) was cleaved using a solution of TFA/DCM (50:50 v/v) at 0 °C; ^#^Fmoc-azaWF-OH derivative (entry 16) was cleaved using 5–10% TFA in DCM at room temperature. Conversion rates were reported based on recovered starting material (b.r.s.m).

### Aza-FSSE analogues have increased efficacy as inhibitors of HMGB1 signaling

To test the thiocarbazate building block peptide editing potential in a real-world setting, we chose a well-characterized and extremely labile four amino acid fragment (FSSE/P5779) of the damage-associated molecular pattern (DAMP) protein, high mobility group-box 1 (HMGB1). This peptide fragment functions as an antagonist of the HMGB1/myeloid differentiation factor-2 (MD-2)/ toll-like receptor 4 (TLR4) pathway and dose-dependently inhibits HMGB1 signaling^42^. The tetrapeptide FSSE (referred to in the literature as P5779), is an ideal candidate to test the azapeptide platform– not only is it therapeutic in a wide array of in vitro assays and in vivo disease models (inflammation, sepsis, pulmonary arterial hypertension, and acetaminophen toxicity^42–44^) but it is also extremely labile (*t*_1/2_ < 2 min) (Table 3). As discussed above, the two central serine residues of FSSE could not be replaced with aza-analogues, which limited our ability to synthesize a comprehensive azapeptide library based on this tetramer. Since the C-terminal glutamic acid (E) shares a similar side chain with glutamine (Q) however, an array of aza-FSSE/Q analogues (Table 3) were synthesized and characterized. Thus, eight aza-analogues of FSSE/Q were successfully synthesized, and final purities were >95%. Fmoc protected FSSE/Q azapeptides showed good crude purities (41.0-93.0%). After removing Fmoc, final azapeptides were obtained with isolated yields of 20.6–63.0% (Table 3); an overview of the example of azaF^1^SSazaE^4^ (**51**) is presented (Table 3). This synthesis was initiated by aza-glutamic acid integration on a Rink amide, producing azaGlu (**60**); under SPPS conditions, Fmoc-protected tripeptide SSazaE^4^ (**61**) was obtained, followed by aza-phenylalanine integration, acid cleavage and deprotection, generating azaF^1^SSazaE^4^ (**51**) (Supplementary Information).

**Table 3:** Summary of HMGB1/MD-2/TLR4 antagonist (FSSE) and FSSE azapeptide analogues


Entry	Sequence	azapeptides	Crude purity (254 nm)*	Isolated Yield*	Purity (215 nm)	Ex-vivo stability t _1/2_ (min)	HMGb1:MD-2 inhibition (IC_50_)
1	FSSE-NH_2_ (P5779)*		-	-	95.9%	2.0 ± 0.06	68.5 nM
2	azaF^1^SSazaE^4^-NH_2_	**51**	67.0%	20.6%	97.9%	>120	90.0 nM
3	azaF^1^SSE-NH_2_	**52**	76.0%	29.5%	96.4%	>120	249 nM
4	FSSazaE^4^-NH_2_	**53**	93.0%	46.0%	97.8%	2.6 ± 0.11	396.8 nM
5	azaF^1^SSE-OH	**54**	90.2%	63.0%	98.0%	> 120	127.4 nM
6	azaF^1^SSQ-OH	**55**	71.0%	29.5%	95.8%	24.4 ± 1.6	159.3 nM
7	azaF^1^SSQ-NH_2_	**56**	82.0%	47.0%	96.4%	>120	348.7 nM
8	azaF^1^SSazaQ^4^-NH_2_	**57**	41.0%	53.0%	96.8%	>120	83.0 nM
9	FSSazaQ^4^-NH_2_	**58**	50.2%	39.0%	97.9%	2.9 ± 0.20	422.9 nM

FSSE-based azapeptides were synthesized by SPPS (aza-position indicated by superscript number); crude or final purity of each peptide was analyzed by HPLC. *Crude purities were measured from Fmoc protected final FSSE azapeptides; Isolated yields were last step (removing Fmoc) purification yield. *FSSE (P5779) was purchased from commercial source; ex-vivo stability, indicated by half-life of FSSE azapeptides in male C57BL/6 J mouse whole blood, was analyzed by LC-MS/MS (mean ± SD, *n* = 3 animals) (Supplementary Table 2a); inhibitory effect against HMGB1/MD-2 binding was analyzed by surface plasmon resonance (SPR) assays, and indicated by inhibitory concentration-50 (IC_50_) values. Data are expressed as means ± SD for independent triplicate measurements (Supplementary Fig. 2). Standard SPPS model reactions for generating capped FSSE azapeptide azaF^1^SSazaE^4^ (**51**) (Supplementary Information).

**Fig. 3:**
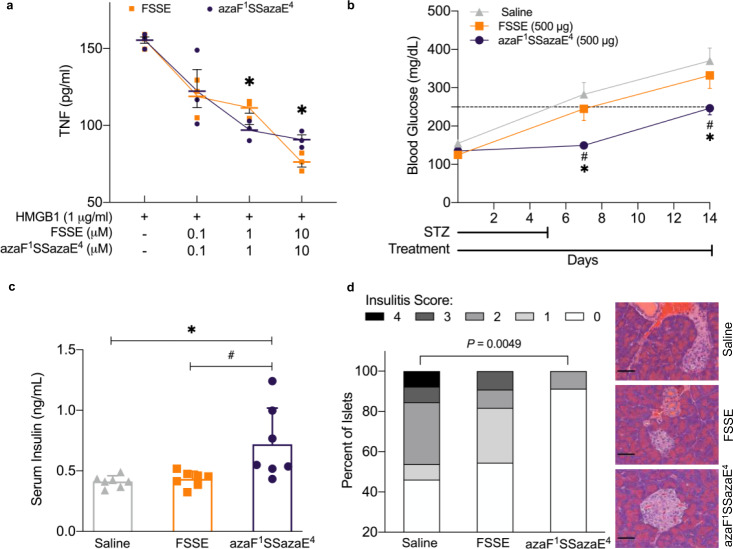
Bioactivities of end-capped azapeptide azaF^1^SSazaE^4^ (**51**). **a** HMGB1/MD-2/TLR4 antagonist FSSE and azaF^1^SSazaE^4^ dose-dependently reduce in vitro HMGB1-stimulated TNF release in murine monocytes. Splenic monocytes from C57BL/6 J mice were incubated with 0.1, 1 or 10 µM of FSSE or azaF^1^SSazaE^4^ in the presence of HMGB1 (1 µg/ml) for 24 h. ELISA measured TNF from cell supernatants. Data are presented as means ± SD. Representative graph of three independent experiments with triplicates. Values are measured by Unpaired *t*-test with Welch correction. *p = 0.046 vs. FSSE group (at 1 µM), *p = 0.033 vs. FSSE group (at 10 µM). **b-d** HMGB1/MD-2/TLR4 antagonist azapeptide analogue azaF^1^SSazaE^4^ delays hyperglycemia in mice challenged with low-dose streptozotocin (STZ). **b** C57BL/6 J male mice (6 weeks of age) were injected i.p. with STZ (50 mg/kg) in sodium citrate buffer (pH 4.5) once daily for 5 days. Mice were simultaneously injected i.p. with saline, FSSE (500 µg/mouse/day), or azaF^1^SSazaE^4^ (500 µg/mouse/day). Blood glucose was measured on day 0, 7 and 14. Number of mice per group: saline *n* = 7; FSSE *n* = 8; azaF^1^SSazaE^4^ *n* = 8. Data are presented as mean values ± SEM. Values are measured by two-way ANOVA *p = 0.0004 vs. saline group on day 7, *p = 0.0009 vs. saline group on day 14; #*p* = 0.0094 vs. FSSE group on day 7, #*p* = 0.0218 vs. FSSE group on day 14. Values above the dashed line indicate type 1 diabetes. **c** Serum levels of insulin are increased in animals treated with azaF^1^SSazaE^4^. Serum samples from mice challenged with STZ were collected at day 14 and analyzed by ELISA. *n* = 7 mice per group. Data are presented as mean values ± SEM. Values are measured by 1-way ANOVA with Tukey’s multiple comparison test, *p = 0.0124 vs. saline group, #*p* = 0.0198 vs. FSSE group. **d** Insulitis is reduced in mice treated with FSSE and azaF^1^SSazaE^4^ as compared to saline treated mice. Percentage of total islets exhibiting various degrees of insulitis (score 0-4) following 2 weeks of treatment with saline, FSSE, or azaF^1^SSazaE^4^ (left panel). Number of islets per group: saline *n* = 3, 13 islets; FSSE *n* = 2, 11 islets; azaF^1^SSazaE^4^ *n* = 3, 23 islets. *P*-value was calculated using two-sided Fisher’s exact test to compare islets without (score 0) and with (score 1-4) insulitis. Pancreas tissue was histologically analyzed from 2–3 animals per group. Representative hematoxylin and eosin (H&E) stained images of islets from each group are shown (right panel). Scale bars, 50 μm.

The ex vivo stability of FSSE azapeptide analogues was investigated in mouse whole blood by measuring the recovery of intact azapeptides over 2 h at 37 °C (Table 3; t_1/2_). While FSSE was rapidly degraded (t_1/2_ of 2 min), certain FSSE azapeptide derivatives displayed markedly increased stability (t_1/2_ of 24–120 min) for azaF^1^ (aza-phenylalanine-position 1)-containing analogues (Table 3: **51** (t_1/2_ > 120 mins), **52** (t_1/2_ > 120 mins), **54** (t_1/2_ > 120 mins), **55** (t_1/2_ 24.4 ± 1.6 mins), **56** (t_1/2_ > 120 mins) and **57** (t_1/2_ > 120 mins)). In contrast, only replacing glutamic acid (**53**, t_1/2_ 2.6 ± 0.11 mins) or glutamine at position 4 (**58**, t_1/2_ 2.9 ± 0.2 mins) with the corresponding aza-analogue failed to improve stability. To evaluate the activity of azapeptides as MD-2 antagonists, FSSE aza-analogues were interrogated for their ability to interrupt the HMGB1/MD-2 interaction using a surface plasmon resonance (SPR)-based binding inhibition assay. Compared to FSSE (IC_50_ 68.5 nM), azaF^1^SSazaE^4^ (**51**) and azaF^1^SSazaQ^4^ (**57**) exhibited comparable inhibitory effects with IC_50_ values of 90.0 nM and 83.0 nM, respectively, while other analogues showed lesser inhibitory activities indicated by their higher IC_50_ values (Table 3, Supplementary Fig. 2). It was speculated that incorporating aza-amino acid residues at both position 1 and position 4 of FSSE would be essential for stability and activity, however the respective FSSE azapeptides half-lives did not always correlate directly with binding inhibition results, suggesting that the 3D structure of the aza-analogues also played an important role. It is known that aza-substitution can influence peptide secondary structure in both desired and undesired respects based on the particular aza-amino acid and position^45^. Significantly, end-capped azaF^1^SSazaE^4^ (**51**) demonstrated extended whole blood stability (*t*_1/2_ > 120 min) and preserved in vitro biological activity. The azaF^1^SSazaE^4^ (**51**) also displayed comparable potency to FSSE at antagonizing HMGB1 binding to MD-2 as assessed in SPR binding inhibition studies, and both FSSE and azaF^1^SSazaE^4^ (**51**) dose-dependently reduced HMGB1-induced tumor necrosis factor-α (TNF) production in murine monocytes (Fig. 3a). FSSE has been used to improve survival in acute HMGB1/TLR4-driven inflammatory diseases, such as influenza^46^ and burn infection^47^. To assess if administration of FSSE and an aza-derivative affects chronic inflammation, streptozotocin (STZ)-induced type 1 diabetic (T1D) mice were treated with FSSE and azaF^1^SSazaE^4^ (**51**) and compared side-by-side (Fig. 3b–d). Compared with the diabetic (saline) group, azaF^1^SSazaE^4^ **(51)** delayed the development of hyperglycemia in mice, whereas FSSE had no significant effect (Fig. 3b–d): the level of blood glucose was significantly attenuated in the azaF^1^SSazaE^4^ (**51**) receiving group (Fig. 3b, Supplementary Fig. 3a); the azapeptide analogue (**51**) successfully restored the serum levels of insulin (Fig. 3c) and hindered diabetes-induced body weight loss (Supplementary Fig. 3b). Moreover, the severity of insulitis examined in azaF^1^SSazaE^4^-treated mice was much lower than that of FSSE-treated mice as evaluated by insulitis scores (Fig. 3d, Supplementary Fig. 4). Additionally, azaF^1^SSazaE^4^ (**51**) was also found to be protective in a mouse model of acetaminophen-induced liver toxicity (N-acetyl-para-aminophenol (APAP) injection) (Supplementary Fig. 5a) and improved liver function enzymes as well as plasma cytokines (Supplementary Fig. 5b). Taken together, these results indicate that azaF^1^SSazaE^4^ (**51**) not only retains (or manifests greater) bioactivity (inhibition of target HMGB1 and therapeutic efficacy), but also has a longer (>60-fold) half-life (increased bioavailability), making it a more attractive therapeutic candidate than the native FSSE peptide.

### Aza-bradykinin analogues have increased efficacy in lowering blood pressure

To further interrogate the limits and potential of our azapeptide platform technology, a more synthetically-challenging peptide, the nine amino acid peptide bradykinin^48^ (BK) (1060.2 Daltons) was chosen. Briefly, BK is a small bioactive peptide liberated by the action of kallikrein on high molecular weight kininogen, a component of the plasma kallikrein-kinin system. Although labile in vivo, BK and its breakdown products have profound effects on pain, inflammation, edema/vasodilation, and blood pressure through their interactions with the bradykinin-1 & 2 receptors (B1R, B2R)^49–51^. Thus, BK and its metabolites represent attractive peptide drug candidates^52^. Additionally, regarding our azapeptide chemistry platform, BK offers a diverse composition of amino acid residues (basic, aromatic, and aliphatic). In this regard, BK allowed us to not only interrogate a larger peptide, but also to identify potential integration and post-functionalization challenges.

Bradykinin is an exquisitely regulated peptide that is rapidly metabolized by multiple proteases and also broken down by chemical means^53–56^. Angiotensin-converting enzyme (ACE), a carboxy dipeptidase (kininase II), converts BK into the inactive metabolite BK (1–7) by removing the C-terminal phe-arg residues. A second cleavage removes residues ser-pro resulting in the pentapeptide BK (1-5). Aminopeptidase P (APP) cleaves BK at the amino-terminal side, transforming BK into the inactive peptide BK (2–9) (des-Arg^1^-BK) by removing the N-terminal arginine. Carboxypeptidase N (CPN) (kininase I) degrades BK to octapeptide des-Arg^9^-BK by removing the C-terminal arginine. In the case of des-Arg^9^-BK (BK 1-8), this biologically active metabolite of BK is further inactivated by ACE and APP. BK is also prone to chemical degradation^56^, spontaneous cleavage at Pro^2^-Pro^3^ occurs in the absence of enzymes and is regulated by the conformation of Arg^1^-Pro^2^.

Based on bradykinin catabolism, a library of BK-based azapeptide analogues was created in which aza-amino acid residues were not only substituted at several protease-susceptible positions in the sequence, but also in a systematic staggered manner throughout the peptide (aza-scan). By applying aza-phenylalanine, aza-proline, aza-glycine^57^, and aza-arginine, nine aza-BK derivatives were generated with respectable crude purities (19.2–85.0%) and yields (5.40–35.4%) (Table 4). To demonstrate these methods, the azapeptide analogue azaF^8^-BK (**68**) (Table 4) is highlighted. The azaF^8^-BK (**68**) was synthesized with an aza-phenylalanine incorporated at position 8 using our original SPPS-compatible platform synthetic methodology. Briefly, aza-phenylalanine was integrated into the free arginine-pre-loaded Wang resin (**72)** to generate dipeptide azaF^8^R (**73**). Using SPPS, azaF^8^-BK (**68**) was produced and isolated with good crude purity of 68.4% (Supplementary Information)^34^.

**Table 4:** Summary of bradykinin and BK-based azapeptide analogues


Entry	Sequence	azapeptides		Crude purity (215 nm)	Isolated Yield	Purity (215 nm)	Ex-vivo stability t _1/2_ (min)
1	RPPGFSPFR	Bradykinin		85.9%	48.0%	98.3%	5.29 ± 0.5
2	azaR^1^PPGFSPFR	azaR^1^-BK	**62**	85.0%	NA*	NA*	2.46 ± 0.05
3	RazaP^2^PGFSPFR	azaP^2^-BK	**63**	38.0%	35.4%	97.0%	5.40 ± 1.1
4	RPazaP^3^GFSPFR	azaP^3^-BK	**64**	53.5%	41.0%	96.9%	2.63 ± 0.25
5	RPPazaG^4^FSPFR	azaG^4^-BK	**65**	34.0%	17.7%	97.6%	4.33 ± 1.3
6	RPPGazaF^5^SPFR	azaF^5^-BK	**66**	66.0%	16.5%	95.3%	14.7 ± 1.6
7	RPPGFSazaP^7^FR	azaP^7^-BK	**67**	49.6%	23.6%	95.0%	2.38 ± 0.03
8	RPPGFSPazaF^8^R	azaF^8^-BK	**68**	68.4%	14.0%	97.6%	39.3 ± 2.5
9	RPPGFSPFazaR^9^	azaR^9^-BK	**69**	19.2%	5.40%	95.9%	42.6 ± 0.8
10	RazaP^2^PGFSPazaF^8^R	[azaP^2^, azaF^8^]-BK	**70**	42.8%	21.0%	98.5%	29.2 ± 3.8
11	RPPGazaF^5^SPazaF^8^R	[azaF^5^, azaF^8^]-BK	**71**	66.0%	17.7%	95.3%	105.8 ± 1.8

Bradykinin-based azapeptides were synthesized by SPPS with respectable yield (aza-positions labeled with superscript); crude or final purity of each peptide was analyzed by HPLC; ex-vivo stability and half-lives of bradykinin-based azapeptides in male C57BL/6 J mouse whole blood were analyzed by LCMS/MS (means ± SD, *n* = 3) *Not available (Supplementary Table 2b). Standard SPPS model reactions for generating bradykinin-based azapeptide (azaF^8^-BK) (Supplementary Information).

**Fig. 4:**
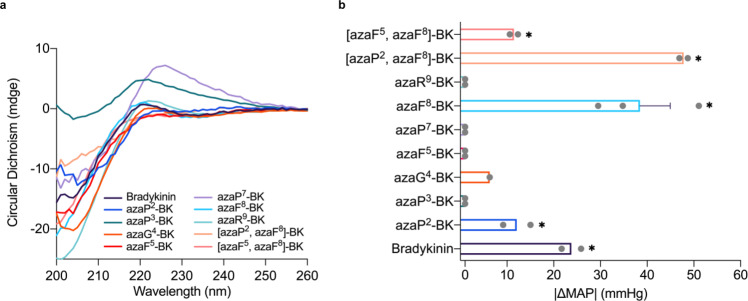
Structural activities and hypotensive effects of bradykinin and BK-based azapeptide analogues. **a** Circular Dichroism (CD) spectra of bradykinin-based azapeptides. Bradykinin azapeptide analogues were scanned from 200 to 260 nm on CD spectrometer for 3 repeats per peptide. The CD spectra were plotted by using the average value of three repeats. **b** Hemodynamic effects (hypotensive) of intravenous bolus injections (6.4 µg/kg) of bradykinin and bradykinin azapeptides indicated by absolute change in mean arterial pressure (MAP) in rats. Each grey data point represents one animal. Mean values (with SEM where indicated) for animals given experimental agent were compared with vehicle. Asterisks denote *p* < 0.05 using a one-tailed T test. *n* = 2 animals for [azaF^5^, azaF^8^]-BK, *p = 0.0029; *n* = 2 animals for [azaP^2^, azaF^8^]-BK, *p = 0.0002; *n* = 3 animals for azaF^8^-BK, (*p = 0.002), *n* = 1 animal for azaG^4^-BK; *n* = 2 animals for azaP^2^-BK, *p = 0.0267; *n* = 2 animals for azaP^3^-BK, azaF^5^-BK, azaP^7^-BK and azaR^9^-BK; *n* = 2 animals for Bradykinin, *p = 0.0038.

To explore the potential loss of chirality at the Cα positions due to integration of aza-amino acids^58^, the conformation and secondary structure of aza-BK analogues was examined using circular dichroism (CD) analysis (Fig. 4a and Supplementary Table 3). While the CD spectra of many of the BK azapeptide analogues indicated a similar conformation to that of BK^59^, a divergent spectral pattern was noticed in the cases of azaP^3^ (**64**) and azaP^7^-BK (**67**) (Fig. 4a and Supplementary Table 3). The absence of negative peaks at 234 nm in these two aza-BK analogues might suggest disruptions of intramolecular hydrogen-bond configurations^60^. In summary, only two of the nine aza-BK analogues appear to possess dramatically altered conformations relative to the native peptide.

Thereafter, these aza-BK analogues were screened for ex vivo whole blood stability and in vivo for vascular reactivity, as measured by the mean arterial blood pressure (MAP) in rats (Table 4 and Fig. 4b). As anticipated from the previous ex vivo stability studies with FSSE, we discovered five aza-BK analogues exhibited improved stability profiles with around 3- to 21-fold longer whole blood half-lives (t_1/2_) than native BK. Of these, azaPhe^8^-containing analogues such as azaF^8^-BK (**68**) (t_1/2_ 39.3 ± 2.5 mins), [azaP^2^, azaF^8^]-BK (**70**) (t_1/2_ 29.2 ± 3.8 mins) and [azaF^5^, azaF^8^]-BK (**71**) (t_1/2_ 105.8 ± 1.8 mins), had significantly extended whole blood stability. Replacing Phe^5^ or Arg^9^ with aza-phenylalanine or aza-arginine (azaF^5^-BK (**66**) and azaR^9^-BK (**69**), respectively) also extended significantly the t_1/2_ (14.7 ± 1.6, 42.6 ± 0.8 min, respectively) in ex vivo whole blood assays. Not surprisingly, aza-amino acid residues incorporated at protease-susceptible cleavage sites resulted in the most stable aza-BK analogues.

Next, in vivo bioactivity of aza-BK analogues was evaluated in an acute blood pressure monitoring study in rats^61^ based on the known blood pressure lowering effect of BK. Briefly, rats were anesthetized and changes in mean arterial blood pressure (MAP) were recorded during intravenous (IV) injection of test agents (6.4 µg/kg) (Fig. 4b). As expected, the injection of BK induced a decrease in MAP (∼Δ 23 mmHg) and both azaF^8^-BK (**68**) and [azaP^2^, azaF^8^]-BK (**70**) (which both had longer t_1/2_’s indicating blood stability) significantly decreased MAP, inducing a ∼2 fold greater reduction than BK. Surprisingly, other more stable BK-azapeptides, such as azaF^5^-BK (**66**), azaR^9^-BK (**69**) and [azaF^5^, azaF^8^]-BK (**71**), had no significant effect on MAP which could be explained by weak binding to B2R. Furthermore, aza-BK analogues with shorter t_1/2_’s (reduced blood stability) including azaP^2^-BK (**63**) and azaG^4^-BK (**65**) showed weak but measurable hypotensive effects. Taken together, these results support the notion that specific azapeptide substitutions can have a range of effects (positive, negative, no effect) on overall 3D structure and proteolytic stability, and therefore biological activities. While there is not always a correlation between these factors, the aza-substitutions at protease-susceptible positions P^2^ and F^8^ in [azaP^2^, azaF^8^]-BK (**70**) improved both the stability (t_1/2_) and enhanced one of the biological activities (ΔMAP) as compared with the native BK peptide.

**Fig. 5:**
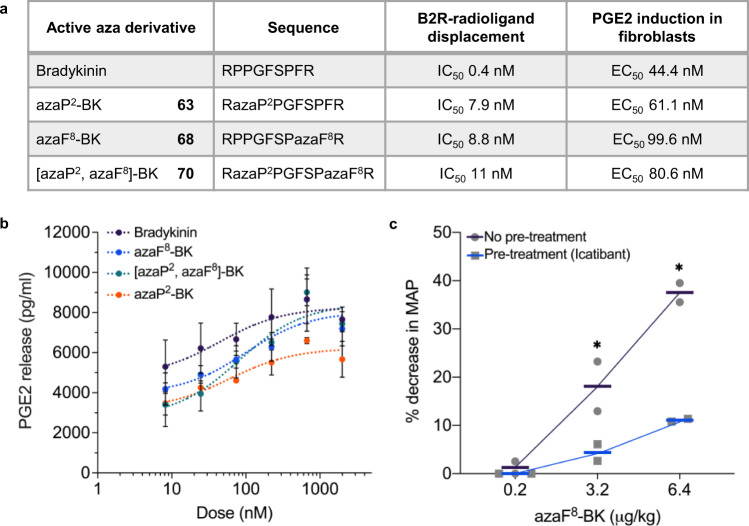
Bioactivities of selected bradykinin azapeptide analogues. **a** Selected bradykinin azapeptide analogues displace a radioactive B2R receptor ligand in the nanomolar range (IC_50_’s) and induce fibroblast prostaglandin E2 (PGE2) production (EC_50_) in vitro. EC_50_’s for bradykinin- and bradykinin azapeptide analogue-induced PGE2 release (right most column) were determined by least squares regression analysis of data plotted in Fig. 5b without weighting and were not significantly different between data sets, as determined by one-way ANOVA on ranks (Kruskal–Wallis *H*-test). **b** Bradykinin and bradykinin azapeptide analogues azaP^2^-BK (**63**), azaF^8^-BK (**68**) and [azaP^2^, azaF^8^]-BK (**70**) dose-dependently stimulate the production of PGE2 in 3T3 cells. Subconfluent monolayers of 3T3 cells were incubated with test compounds for 7 min in serum-free medium and supernatants were collected and analyzed for PGE2 by ELISA. Values are means ± SEM of raw PGE2 ELISA data from three independent dose-response experiments performed with triplicates (see Supplementary Fig. 6 for an alternative bar plot showing individual data points from each of the three independent experiments used to calculate the mean ± SEM of PGE2 release data plotted in Fig. 5b as well as the amount of PGE2 released by control-treated cells). **c** B2R antagonist Icatibant blocks lowering effects of bradykinin azapeptide (azaF^8^-BK (**68**) on mean arterial pressure (MAP) in rats). Icatibant (10 µg/kg) was injected as IV-bolus 15 min before azaF^8^-BK administration. The solid horizontal lines represent mean values from individual rats (*n* = 2). Asterisks denote *p* < 0.05 one-tailed (*p*-values from left to right: no pre-treatment 0.0362, 0.0014; pre-treatment 0.0003).

Bradykinin acts through two receptors termed B1R and B2R, but activation of B2R plays the major role in the most prevalent physiological actions of BK. B2R agonists may have clinical value in the treatment and prevention of different cardiovascular disorders. From the experiments delineated above, three leading aza-BK analogues (Fig. 5) were selected to explore their potential as B2R agonists. Initially, these azapeptides were assessed for receptor binding (B2R) using standardized agonist radioligand displacement assays. All three BK-azapeptides showed agonist displacement in the same magnitude (low nM range), comparable to native BK (Fig. 5a, Supplementary Fig. 7 (Source Data file)). Next, aza-BK analogues’ effects on prostaglandin E2 (PGE2) release from fibroblasts was evaluated; azaP^2^-BK (**63**), azaF^8^-BK (**68**) and [azaP^2^, azaF^8^]-BK (**70**) were able to dose-dependently stimulate PGE2 production with EC_50_’s in the same nanomolar range as BK (Fig. 5a & b). Since BK-induced PGE2 release is known to occur through B2R^62^, it was surmised that these aza-BK analogues functioned as B2R agonists. To confirm this, the clinically-approved B2R antagonist icatibant was used to test whether the blood pressure lowering effect (ΔMAP) was dependent on the B2R. Pretreatment with icatibant elicited a partial inhibition of the azaF^8^-BK (**68**)-induced lowering of blood pressure (Fig. 5c). Taken together, these results indicate that our azapeptide methodology is convenient and universal (broadly applicable; functioning well on diverse amino acid residues) and is able to produce BK azapeptide analogues that possess increased stability(t_1/2_), while maintaining many biological activities (B2R binding, PGE2 induction, hemodynamic activity) of the native BK peptide.

We report the rationale for, and systematic development of, S-ethyl thiocarbazate building blocks and associated methods as a broadly applicable platform technology to synthesize azapeptides. Our research substantiates that substituted thiocarbazates can function as stable and modular aza-amino acid building blocks. These thiocarbazate amino acid building blocks are: (1) efficient as acyl-transfer agents upon activation under mild conditions; (2) exceedingly compatible with conventional amino acid protecting groups; and (3) easily targeted to specific sites within a desired peptide sequence to generate azapeptides, using solution phase and/or standard solid-phase peptide synthesis approaches.

In theory, synthesis of thiocarbazate amino acid building blocks could be achieved for all twenty natural amino acids; however, in this study we synthesized and characterized only sixteen thiocarbazate amino acids. While serine and threonine are compatible for synthesis using our methods, we expected that upon activation, the resultant oxygen at the radical site will be unstable in an aqueous environment due to the intrinsic properties of the hemi-aminal. Additionally, the remaining two sulfur-containing amino acids (methionine and cysteine) would be incompatible with the activation condition because of their reactivity with haloamides.

As proof-of-concept, using our platform synthetic technology we have built curated azapeptide libraries around two well-characterized peptides, FSSE and bradykinin. We found that an azapeptide azaF^1^SSazaE^4^ (**51**) derived from the previously studied HMGB1/MD-2/TLR4 peptide antagonist FSSE showed increased stability, improved efficacy on in vitro parameters related to inflammation (HMGB1-induced TNF induction), and reduced disease severity in a streptozotocin model of T1D. Additionally, the aza-scan synthesis (systematic targeted aza-amino acid substitutions) of bradykinin azapeptide analogues revealed no integration or post-functionalization challenges inherent in producing analogues of this larger, and more diverse peptide. Further, several bradykinin azapeptides possessed, not only extended stability, but also agonistic biological activities both in vitro and in vivo (B2R binding, PGE2 induction, and blood pressure regulation). Taken together, the results from these potential therapeutic candidates serve as a demonstration of the capabilities of our azapeptide synthesis platform.

As a whole, our platform methods simplify solid phase azapeptide synthesis and allow for safer and more efficient generation of azapeptide analogues that can be screened for desired biological activities. The potential for peptide and peptidomimetic therapeutics in disease outbreaks and pandemic situations is well-appreciated^63^, thus, the use of our methodology to rapidly synthesize and screen azapeptide analogues could serve to advance the class of azapeptides as drug candidates. We envision this platform azapeptide synthesis technology could accelerate the advancement of both existing and unique peptide-based agents as therapeutics.

## Methods

### General procedure for the synthesis of thiocarbazate amino acid building blocks

To a solution of substituted hydrazine (1 mmol) in THF (5 mL), S-ethyl chlorothioformate 0.5 M solution was added with DCM (2 mL) and pyridine (1 mmol). The reaction mixture was stirred at 0 °C for 1 h and gradually warmed up to room temperature and stirred for an additional 1 h. Then, the reaction mixture was quenched with water and transferred to a separatory funnel. The organic layer was washed with brine, dried over Na_2_SO_4_, filtered, and evaporated under vacuum. The crude material was purified on silica with the use of a gradient of hexanes/EtOAc. Synthesis and characterization of thiocarbazates can be found in Supplementary Information.

### General procedure for the synthesis of azadipeptide (18-34) in solution phase

#### Protocol A for synthesis of 18-25 and 29-34

An appropriate thiocarbazate (0.1 mmol) and TBACl (0.15 mmol) was introduced to a small vial charged with a magnetic stirrer bar. The solids were dissolved entirely in DCM (2.0 mL) and cooled to 0 °C using an ice bath. After 10 min, the reaction mixture was treated with a freshly pulverized TCCA (0.085 mmol). The reaction mixture was then stirred vigorously at 0 °C for 10 min. At this point, the mixture was treated with an appropriate amino acid ester (tert-butyl L-Valinate) (0.21 mmol) and appropriate tertiary amine (Et_3_N) (0.44 mmol) at 0 °C. The ice bath was removed, and the stirring continued until complete conversion based on TLC (35–60 min). The reaction mixture was treated with a saturated solution of Na_2_S_2_O_3_ (1.0 mL) and NaHSO_4_ (1.0 mL). The aqueous layer was extracted with EtOAc (2.0 mL × 3). The combined organic layer was washed with brine (2.0 mL), dried over anhydrous Na_2_SO_4_, filtered, and evaporated under vacuum. The resulting crude material was then purified using flash chromatography using silica gel and gradient of EtOAc/hexanes. HPLC analysis is run to confirm the purity before submitting the samples to NMR analysis and HRMS.

#### Protocol B for synthesis of 26-28

An appropriate thiocarbazate (0.1 mmol) and TBACl (0.15 mmol) was introduced to a small vial charged with a magnetic stirrer bar. The solids were dissolved entirely in DCM (2.0 mL) and cooled to −10 °C using an ice/acetone bath. After 10 min, the reaction mixture was treated with a freshly pulverized TCCA (0.039 mmol). The reaction mixture was then stirred vigorously at −10 °C for 10 min. At this point, the mixture was treated with an appropriate amino acid ester (tert-butyl L-Valinate) (0.21 mmol) and appropriate tertiary amine (Et_3_N) (0.44 mmol) at 0 °C. The ice bath was removed, and the stirring continued until complete conversion based on TLC (35–60 min). The reaction mixture was treated with a saturated solution of Na_2_S_2_O_3_ (1.0 mL) and NaHSO_4_ (1.0 mL). The aqueous layer was extracted with EtOAc (2.0 mL X 3). The combined organic layer was washed with brine (2.0 mL), dried over anhydrous Na_2_SO_4_, filtered, and evaporated under vacuum. The resulting crude material was then purified using flash chromatography using silica gel and gradient of EtOAc/hexanes. HPLC analysis was run to confirm the purity before submitting the samples to NMR analysis and HRMS.

### General procedure for solid-phase synthesis (SPPS)

All solid phase peptide couplings were performed at ambient temperature using a Tribute® Peptide synthesizer from Gyros Protein Technologies, Inc following standard protocol which is described sequentially below:

1. Swelling: The resin (loaded with the first Fmoc-protected amino acid) was swelled twice successively for 20 min in DMF, each swelling step was followed by a drainage and drying step.
2. Fmoc Cleavage: the protected amino acid/or peptide was shaken for 2.0 min with 20% piperidine solution in DMF to remove the Fmoc group. The process was repeated twice, followed by several washing steps with DMF (3-5 times).
3. Amino acid coupling: 5 equivalents of the next acylating component (Fmoc-protected amino acid), 5 equivalents of HATU (coupling reagent), and 10 equivalents of N-methylmorpholine (base) were used to add the next amino acid in the sequence. This step is fully automated and was run according to the software installed on the Tribute® synthesizer. The amino acid including coupling reagent was delivered to the reaction vessel from the specified loading position upon dissolution. Then the base was added as 0.4 M solution in DMF, the total volume of solvent was adjusted to give 0.2 M solution. The coupling time was limited to 15 min shaking followed by drainage then washing steps. Step 2 and step 3 were repeated until the desired peptide sequence was achieved.
4. Washing: repeated washing steps were performed after each cleavage or coupling event using DMF as solvent (2-3 times). At the final coupling or cleavage steps additional washing with DCM was performed (5-6 times) to remove any trace of DMF. The process was usually followed by a drying step.
5. Cleavage from the resin: 5.0 mL of a freshly made solution of TFA/H_2_O/TIPS (95:2.5:2.5. v/v/v) was cooled down to 0 °C and added at 0 °C to a 0.3 mmol of resin. The mixture was shaken for 2 h, filtered, and the remaining resin was further washed with a 0.5–1.0 mL of TFA/H_2_O (95:5,v/v) solution. The filtrate was precipitated by adding 10 mL of 1:1 solution of ether: hexane. Upon centrifugation, the resulting solid was dissolved in a 1:1 solution of CH_3_CN: H_2_O. The resulting solution was lyophilized.

Purification: Purification of the peptidomimetics were performed on a preparative HPLC purification system (Waters Prep 150 LC system combining 2545 Binary Gradient Module using XSelect Peptide CSH C18 OBD Prep Column, 130 Å, 5 µm, 19 mm × 150 mm). Chromatography was performed at ambient temperature with a flow rate of 18 mL/min with a linear gradient from water (0.1% FA): CH_3_CN (0.1% FA)[95:5] to water (0.1% FA): CH_3_CN (0.1% TFA) [5:95] in 12 min, monitored by 2998 Photodiode Array (PDA) Detector UV at 254 nm and/or 215 nM.

#### Integration of the thiocarbazate amino acid in SPPS

##### Procedure A

Wang or Rink amide amino acid-loaded resin (0.1 mmol) was swelled for 10 min in DMF (2.5 mL × 2), followed by Fmoc cleavage using 20% solution of piperidine in DMF (2.5 mL × 2)/2 min per cleavage cycle. After successive washes with DMF (2.5 mL × 3)/30 s per wash and with DCM (2.5 mL × 5)/ 30 s per wash. The resin was then suspended in CH_3_CN (0.5 mL) and treated with N-methylmorpholine base (NMM) (1.0 mmol, 10 equiv.) for 10 min. During this time, and separately, in a small vial, thiocarbazate (0.5 mmol, 5 equiv) and tetrabutylammonium chloride (TBACl) (0.5 mmol, 5 equiv) were dissolved completely in DCM (2.0 mL). Then, the reaction mixture was treated with trichloroisocyanuric acid (TCCA) (0.5 mmol, 5 equiv). The reaction mixture was stirred at rt for 5 min and then centrifuged. The clear supernatant was added directly to the resin, and the reaction mixture was shaken for 16 h, followed by 5 cycles of washing and then drying. A small amount of the resin was cleaved using a freshly made solution of TFA/H_2_O/TIPS (95:2.5:2.5 v/v/v) and the resulting peptide was analyzed by HPLC.

##### Procedure B

(A modified version of procedure A to accommodate the reactive side chains, specifically for the tryptophan and histidine residues) Wang or Rink amide amino acid-loaded resin (0.1 mmol) was swelled for 10 min in DMF (2.5 mLx2), followed by Fmoc cleavage using 20% solution of piperidine in DMF (2.5 mL × 2)/ two min per cleavage cycle. After successive washes with DMF (2.5 mL × 3)/ 30 s per wash and with DCM (2.5 mL × 5)/30 s per wash. The resin was then suspended in CH_3_CN (0.5 mL) and treated with N-methyl morpholine base (NMM) (1.0 mmol, 10 equiv.) for 10 min. During this time, and separately, in a small vial, thiocarbazate (0.5 mmol. 5 equiv) and tetrabutylammonium chloride (TBACl) (0.75 mmol, 7.5 equiv) were dissolved completely in DCM (2.0 mL). Then, the reaction mixture was cooled down to −10 °C using an ice/acetone bath. After 10 min at −10 °C, the mixture was treated with trichloroisocyanuric acid (TCCA) (0.195 mmol, 0.39 equiv). The reaction mixture was stirred at −10 °C for 15 min and then treated with indole (0.5 mmol, 5.0 equiv). The mixture was allowed to reach rt and centrifuged. The clear supernatant was added directly to the resin, and the reaction mixture was shaken for 16 h, followed by five cycles of washing and then drying. A small amount of the resin was cleaved using a freshly made solution of TFA/H_2_O/TIPS (95:2.5:2.5. v/v/v) and the resulting peptide was analyzed by HPLC.

#### Coupling to the aza-amino acids bound to the peptidyl chain

Functionalization of the aza-amino acid moieties for the solid phase was performed using two different protocols: coupling to the aza-amino acid using amino acid and hexafluorophosphate azabenzotriazole tetramethyl uronium (HATU) as coupling agent, and coupling to the aza-amino acid using amino acid acyl chloride and solid NaHCO_3_. In one example described in Table 4, azaF^8^-BK (**68**) was synthesized with the second coupling method. All eight FSSE azapeptide analogues (Table 3) and ten aza-BK derivatives (Table 4) were created using the same protocol by integrating the aza-amino acid moiety at different positions of the parent sequence. Synthesis and characterization of azapeptides analogues can be found in Supplementary Information.

### In vitro and in vivo animal experiments

In vitro and in vivo materials and methods can be found in Supplementary Information. Mice and rats were given free access to water and standard rodent chow and were acclimated to their environment for at least 1 week before experimentation. All animals were housed in the Center for Comparative Physiology of the Feinstein Institutes for Medical Research under standard temperature and humidity, 12 h light and dark cycle conditions. All animal procedures were approved by the Feinstein Institutes for Medical Research Institutional Animal Care and Use Committee (IACUC, protocols #2009-048, #2011-035, #2013-021, #2018-011) and adhere to current guidelines. The facility is accredited by the Association of the Assessment of Laboratory Animal Care, international, (AAALAC); (PHS Assurance: #D16-00107 (formerly A3168-01), USDA Registration: #21R0107, NYS Registration: #A-060, AAALAC: #000751).

As the methods documented in this manuscript and supplemental information detail the chemical synthesis of reagents for creation of an unlimited array of customized azapeptides using standard SPPS instruments, the authors will not provide these starting materials as it would be cost-prohibitive. These methods can be used for research purposes only and aza-building blocks and methods have been patented and licensed by the Feinstein Institutes for Medical Research (contact: Dr. Kirk Manogue (kmanogue@northwell.edu)).

### Reporting summary

Further information on research design is available in the Nature Portfolio Reporting Summary linked to this article.

## Supplementary information

Supplementary Information

Reporting Summary

## Data Availability

All relevant data to the manuscript generated for these studies are included in the article or supplemental information. The X-ray crystallographic coordinates for structures reported in this study have been deposited in the Cambridge Crystallographic Data Centre (CCDC), under deposition numbers CCDC-2195262 and CCDC-2195263. Raw data from Figures (Figs. 2, 3, 4, 5) are provided in Supplementary information. Raw data for B2R binding inhibition are provided in a Source Data file. Source data are provided with this paper.

## Source data

Source Data
