# Supplementary material for: Thiocarbazate building blocks enable the construction of azapeptides for rapid development of therapeutic candidates
Source: Nat Commun. 2022 Nov 28;13:7127. doi: 10.1038/s41467-022-34712-9 (PMC9705435; doi:10.1038/s41467-022-34712-9)
Supplement: Supplementary file 1 — Supplementary Information [file 41467_2022_34712_MOESM1_ESM.pdf]

SUPPLEMENTARY INFORMATION

Thiocarbazate building blocks enable the construction of azapeptides for rapid development of therapeutic candidates

Ahmad Altiti[1#*], Mingzhu He[1#], Sonya VanPatten[1], Kai Fan Cheng[1], Umair Ahmed[1], Pui Yan Chiu[2], Ibrahim T Mughrabi[1], Bayan Al Jabari[1], Ronald M. Burch[3], Kirk R. Manogue[4], Kevin J. Tracey[1], Betty Diamond[2], Christine N. Metz[2,5], Huan Yang[1], LaQueta K Hudson[5], Stavros Zanos[1], Myoungsun Son[2], Barbara Sherry[2], Thomas R. Coleman[4], Yousef Al-Abed[1,4*]

[1]Institute of Bioelectronic Medicine, Feinstein Institutes for Medical Research, Northwell Health, Manhasset, NY, USA
[2]Institute of Molecular Medicine, Feinstein Institutes for Medical Research, Manhasset, NY, USA
[3]Applied Immunotherapeutics, Inc., Morris, CT, USA
[4]Center for Molecular Innovation, Feinstein Institutes for Medical Research, Manhasset, NY, USA
[5] Donald and Barbara Zucker School of Medicine at Hofstra/Northwell, Hempstead, NY, USA

[#]equal contribution
*corresponding authors:
ahmadtiti@gmail.com
yalabed@northwell.edu

Table of Contents

Supplementary Methods……………………………………………………………………………3
1. Chemistry………………………………………………………………………………3
   1.1 Chemicals…………………………………………………………………………......3
   1.2 Instrumentation for synthesis, purification, and chemical characterization……………………3
2. Solution and Solid phase peptide synthesis (SPPS)……………………………………………3
   2.1 Synthesis and characterization of 16 thiocarbazate amino acid building blocks………………3
   2.2 Synthesis and characterization of azadipeptide in solution phase (18-34)……………………9
   2.3 General procedure for solid phase peptide synthesis (SPPS)…………………………………15
   2.4 Synthesis and Characterization of FSSE azapeptide analogues………………………………17
   2.5 Synthesis and Characterization of bradykinin azapeptide analogues…………………………21
3 Ex vivo stabilities and half-life experiments of FSSE and bradykinin-based azapeptides………25
4 FSSE and aza-analogue studies……………………………………………………………..25
5. Bradykinin and aza-analogues studies…………………………………………………………27

Supplementary Discussion….……………………………………………………………..28
1. Supplementary Table 1. Fmoc-protected thiocarbazate amino acid building blocks…………….28
2. Supplementary Table 2. Ex-vivo Stabilities of FSSE and bradykinin-based azapeptides……….29
3. Supplementary Table 3. The ratio of secondary structures of bradykinin-based azapeptides…….30
4. Supplementary Table 4. Optimized reaction conditions for the thiocarbazates activation and coupling in solution-phase chemistry……………………………………………………………31
5. Supplementary Figure 1: X-ray crystal structures of Fmoc-azaPhe chloride………………...31-32
6. Supplementary Figure 2. FSSE-based azapeptides inhibit HMGB1/MD-2 directly binding……32
7. Supplementary Figure 3. HMGB1/MD-2/TLR4 antagonist azapeptide analogue azaF^{1}SSazaE^4 delays hyperglycemia in mice challenged with low-dose streptozotocin………………………...33
8. Supplementary Figure 4. Distribution of insulitis score of H&E stained pancreatic islets from the indicated group ……………………………………………………………………………..33
9. Supplementary Figure 5. azaF^{1}SSazaE^4 reduces APAP-induced liver injury…………………...34
10. Supplementary Figure 6. Alternative bar plot representation of the dose-dependent PGE2 release data plotted in Figure 5b of the main text ……………………………………………………….35
11. Supplementary Figure 7. Human B2 receptor binding assay for lead bradykinin azapeptides….36

Supplementary Notes……………………………………………………………………...37
1. X-ray crystallographic data…………………………………………………………………37
2. Copy of NMR spectra -Supplementary Figures 8-60…….……………………………...40-92

Supplementary References………………………………………………………………..93

Supplementary Methods

1. CHEMISTRY

1.1 Chemicals

Preloaded Fmoc-L-Arg(Pbf) Wang resin (200-400 mesh, 0.3 mmol/g) and Rink amide MBHA resin (0.3 mmol/g), and premixed Fmoc amino acids with HATU coupling agent were purchased from Pure Pep TM/ Gyros Protein Technologies, Inc. All solvents, and reagents used in synthesis are peptide-grade and were purchased from Gyros Protein Technologies or from Sigma-Aldrich (St. Louis, MO). The S-ethyl chlorothioformate was obtained from different sources: from Sigma Aldrich, E17909, CAS: 2941-64-2 and Acros Organics, Code 347160050, Lot: A0257285, CAS: 2941-64-2. When the reagent was discontinued, we ordered large amounts of the equivalent S-isopropyl chlorothioformate from Enamine Chemicals, Cat #: EN300-7186918, CAS: 13889-93-5. We also tested the production of the S-ethyl chlorothioformate in our labs using the procedure described in the following reference[3] "Synthesis, 2006, 4, 659-665". The material we made as a stock solution of 0.5 M concentration and used directly without further purification or isolation, the synthetic procedure and characterization of S-ethyl chlorothioformate are provided below. The solvents and reagents were used without further treatment or drying.

1.2 Instrumentation for synthesis, purification and chemical characterization

Solid phase peptide syntheses (SPPS) were executed using Tribute® Peptide synthesizer from Gyros Protein Technologies, Inc. The machine is fully automated with two independent reaction vessels with polytetrafluoroethylene frits, five solvent positions and 101 amino acid positions. The aza-amino acids were synthesized in our labs and integrated in the synthesis with minimal interruption to the automation, i.e, the activated aza-amino acid residues were added manually, by interrupting the synthesis momentarily, then the rest of the steps including the cleavage, washing, and drying were maintained automated. Analyses were performed using Water's technology HPLC equipped with a 1525 binary pump, and the use of analytical column (Phenomenex kinetex 2.6 mm EVO C18 analytical column 100Å 150x4.6 mm). Chromatography was performed at ambient temperature with flow rate of 1.0 mL/min with linear gradient from Water (0.05% TFA): CH_3CN (0.05% TFA) [95:5] to Water (0.05% TFA): CH_3CN (0.05% TFA) [5:95] and resolved peaks were detected by 2998 photodiode Array (PDA) Detector at 254 and/or 215 nm and characterized by low resolution mass spectrometry instrument (Thermo Scientific LTQXL ™) with ESI ion-source and positive mode ionization and high resolution mass spectrometry instrument (Agilent 6550 iFunnel QTOF LC/MS). Purification of the all peptidomimetics were performed on preparative HPLC purification system (Waters Prep 150 LC system combining 2545 Binary Gradient Module using XSelect Peptide CSH C18 OBD Prep Column, 130Å, 5 µm, 19 mm X 150 mm. Chromatography was performed at ambient temperature with a flow rate of 18mL/min with a linear gradient from Water (0.1% FA): CH_3CN (0.1% FA)[95:5] to Water (0.1% FA): CH_3CN (0.1% TFA) [5:95] in 12 minutes, monitored by 2998 Photodiode Array (PDA) Detector UV at 254 nm and/or 215. NMR spectra were recorded in acetone-d6, $CDCl_3$, D_2O, and DMSO-d6 with TMS for 1H (500 and/or 600 MHz) and 13C (125 and/or 150 MHz) as an internal reference. NMR spectra were recorded on a Bruker 600 MHz Avance III Spectrometer or a 500 MHz Avance DRX Cryoprobe Spectrometer. NMR data are reported as follows: chemical shifts in parts per million (ppm) referring to the solvent residual peak, multiplicities (s = singlet, d = doublet, t = triplet, q =quartet; m = multiplet, br = broad) and coupling constant values in Hz.

2. SOLUTION AND SOLID PHASE PEPTIDE SYNTHESIS (SPPS)

2.1 Synthesis and characterization of 16 thiocarbazate amino acid building blocks

General procedure for the synthesis of thiocarbazate amino acid building blocks: To a solution of substituted hydrazine (1 mmol) in THF (5 mL), an S-ethyl chlorothioformate 0.5 M solution was added in DCM (2 mL) and pyridine (1 mmol). The reaction mixture was stirred at 0 °C for 1 h and gradually warmed up to room temperature and stirred for an additional 1 hour. Then, the reaction mixture was quenched with water and transferred to a separatory funnel. The organic layer was washed with brine, dried over Na_2SO_4, filtered, and evaporated under vacuum. The crude material was purified by CombiFlash® Teledyne Isco Chromatography with a linear gradient from hexanes: EtOAc [95:5] to hexanes: EtOAc [5:95] in 20 minutes. Characterization of 16 thiocarbazates can be found in following section.

Starting materials (substituted hydrazines) were reported previously, all Fmoc protected hydrazines were either purchased from Sigma-Aldrich or synthesized according to published literatures[4, 5].

S-ethyl chlorothioformate 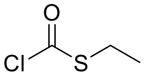

To a 10 mL round-bottom flask charged with deuterated dichloromethane CD_2Cl_2 (1.0 mL) was introduced ethanethiol (28.5 mL, 0.4 mmol) and triphosgene (50.8 mg, 0.17 mmol). The reaction mixture was cooled down to -10 °C using an ice bath/ acetone. Then, the mixture was treated with deuterated pyridine-*d5* (32 uL, 0.4 mmol). The resulting mixture was stirred at 0 °C for another hour. The reaction mixture was quenched with D_2O (0.5 mL). The organic phase was isolated and washed with D_2O (0.5 mL X2). The CD_2Cl_2 layer was dried over Na_2SO_4, filtered, and transferred directly to an NMR tube for analysis. ^{1}HNMR (CD_2Cl_2, 600 MHz) δ 2.97 (q, *J* = 7.4 Hz, 2H), 1.35 (t, *J* = 7.4 Hz, 3H). ^{13}CNMR (CD_2Cl_2, 150 MHz), δ 166.3, 29.3, 14.3.

(9H-fluoren-9-yl)methyl 2-((ethylthio)carbonyl)hydrazine-1-carboxylate (1)

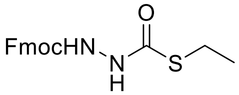

To a solution of Fmoc hydrazine (1 mmol) in THF (5 mL) was added S-ethyl chlorothioformate 0.5 M solution in DCM (2 mL) and pyridine (1mmol). The reaction mixture was stirred at 0 °C for one hour, gradually warmed up to rt, and stirred for an additional hour. Then, the reaction mixture was mixed with water and transferred to a separatory funnel. The organic layer was washed with brine, dried over Na_2SO_4, filtered, and evaporated under vacuum. The crude material was purified on silica and the use of the gradient of Hexanes/EtOAc. azaGly thiocarbazate (**5**) was collected as a white powder in 75% yield. With purity of 96.9 % based on HPLC, RT = 11.17 (flow rate of 1 mL/min with a linear gradient from water (0.05% TFA): AcCN (0.05% TFA) [95:5] to water (0.05%TFA): AcCN (0.05% TFA) [5:95] in 15 min, monitored /detected UV at 254 nM by photodiode Array Detector. ^{1}HNMR for the major isomer (500MHz, DMSO-d6) δ 9.89 (bs, 1H), 9.62 (bs, 1H), 7.90 (d, *J* = 7.5 Hz, 2H), 7.71 (m, 2H), 7.47-7.06 (m, 4H), 4.36 (m, 2H), 4.25 (m, 1H), 2.71 (m, 2H), 1.17 (m, 3H). ^{13}CNMR (125 MHz, DMSOd6) δ 173.8, 157.0, 144.6, 144.5, 141.7, 130.2, 129.3, 128.7, 128.5, 128.1, 127.6, 121.2, 67.2, 47.4, 24.0, 16.3. LRMS m/z calculated for $C_{18}H_{18}N_2O_3S$ [M+H] 342.10 found 343.00. HRMS m/z calculated for $C_{18}H_{18}N_2O_3S$ [M+Na] 365.0936 found 365.0932

(9H-fluoren-9-yl)methyl 2-((ethylthio)carbonyl)-2-methylhydrazine-1-carboxylate (2)

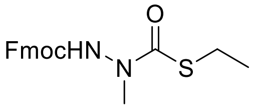

This compound was made according to the general procedure and collected as white fluffy powder in 90% yield. With purity of 97.7% based on HPLC, RT = 10.09 min (flow rate of 1 mL/min with a linear gradient from water (0.05% TFA): AcCN (0.05% TFA) [75:25] to water (0.05%TFA): AcCN (0.05% TFA) [10:90] in 14 min, monitored /detected UV at 254 nM by photodiode Array Detector. ^{1}HNMR (500MHz, DMSO-d_6) δ 10.05 (s, 1H), 7.91 (d, *J* = 7.40 Hz, 2H), 7.72 (m, 2H), 7.43 (t, *J* = 7.2 Hz, 2H), 7.35 (m, 2H), 4.53-4.41 (m, 2H), 4.29 (t, *J* = 6.4 Hz, 1H), 3.02 (s, 3H), 2.70 (m, 2H), 1.18 (t, *J* = 8.0 Hz, 3H). ^{13}CNMR (125 MHz, DMSOd_6) δ 172.0, 155.9, 144.4, 144.3, 141.8, 128.7, 128.1, 126.1, 121.1, 67.3, 47.5, 36.9, 24.5, 16.1 LRMS m/z calculated for $C_{19}H_{21}N_2O_3SH$ [M+H] 357.13 found 357.00. HRMS m/z calculated for $C_{19}H_{21}N_2O_3SH$ [M+H] 357.1196 found 357.1266.

(9H-fluoren-9-yl)methyl 2-((ethylthio)carbonyl)-2-isopropylhydrazine-1-carboxylate (3)

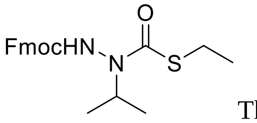

This compound was made according to the general procedure and collected as white fluffy powder in 85% yield. With purity of 96.5% based on HPLC, RT = 12.87 min (flow rate of 1 mL/min with a linear gradient from water (0.05% TFA): AcCN (0.05% TFA) [95:5] to water (0.05%TFA): AcCN(0.05% TFA) [5:95] in 15 min, monitored /detected UV at 254 nM by photodiode Array Detector. ^{1}HNMR (500MHz, DMSO-d_6) d 9.88 (s, 1H), 7.92 (d, *J* = 7.55 Hz, 2H), 7.75 (m, 2H), 7.43 (t, *J* = 7.4 Hz, 2H), 7.35 (m, 2H), 4.4 (m, 3H), 4.28 (t, *J* = 6.6 Hz, 1H), 2.70 (q, *J* = 7.2, 14.5 Hz, 2H), 1.16 (t, *J* = 7.3 Hz, 3H), 1.00 (t, *J* = 7.2 Hz, 6H). ^{13}CNMR (125 MHz, DMSOd_6) d 171.4, 156.7, 144.5, 144.3, 141.8, 128.7, 128.1, 126.2, 126.1, 121.2, 121.1, 67.0, 55.9, 50.4, 47.7, 24.4, 20.4, 20.0, 16.1 LRMS m/z calculated for $C_{21}H_{25}N_2O_3S$ [M+H] 385.15 found 385.00. HRMS m/z calculated for $C_{21}H_{25}N_2O_3S$ [M+H] 385.1508 found 385.1579

(9H-fluoren-9-yl)methyl 2-(sec-butyl)-2-((ethylthio)carbonyl)hydrazine-1-carboxylate (4)

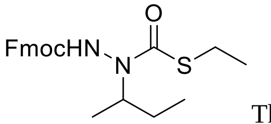

This compound was made according to the general procedure and collected as white fluffy powder in 69% yield. With purity of 99.0% based on HPLC, RT = 13.46 min (flow rate of 1 mL/min with a linear gradient from water (0.05% TFA): AcCN (0.05% TFA) [95:5] to water (0.05%TFA): AcCN (0.05% TFA) [5:95] in 15 min, monitored /detected UV at 254 nM by photodiode Array Detector. ^{1}HNMR (500MHz, DMSO-d_6) δ 9.86 (d, *J* = 8.3 Hz, 1H), 7.91 (d, *J* = 7.55 Hz, 2H), 7.75 (m, 2H), 7.43 (t, *J* = 7.4 Hz, 2H), 7.35 (t, *J* = 7.4 Hz, 2H), 4.47 (m, 2H), 4.27 (m, 2H), 2.70 (q, *J* = 7.0, 14.4 Hz, 2H), 1.43 (m, 1H), 1.15 (t, *J* = 7.3 Hz, 3H), 0.97 (d, *J* = 6.7 Hz, 3H), 0.76 (t, J = 7.3 Hz, 3H). ^{13}CNMR (125 MHz, DMSOd_6) δ 171.9, 156.8, 144.5, 144.3, 141.8, 128.7, 128.1, 126.2, 121.1, 67.0, 55.8, 47.7, 27.7, 24.5, 18.1, 16.0, 12.2, 11.8 LRMS m/z calculated for $C_{22}H_{27}N_2O_3S$ [M+H] 399.17 found 399.00. HRMS m/z calculated for $C_{22}H_{27}N_2O_3S$ [M+H] 399.1664 found 399.1738.

(9H-fluoren-9-yl)methyl 2-((ethylthio)carbonyl)-2-isobutylhydrazine-1-carboxylate (5)

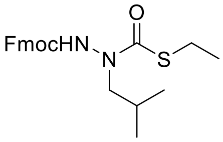

This compound was made according to the general procedure and collected as white fluffy powder in 72% yield. With purity of 87.2% based on HPLC, RT = 13.59 min (flow rate of 1 mL/min with a linear gradient from water (0.05% TFA): AcCN (0.05% TFA) [95:5] to water (0.05%TFA): AcCN(0.05% TFA) [5:95] in 15 min, monitored /detected UV at 254 nM by photodiode Array Detector. ^{1}HNMR (500MHz, DMSO-d_6) δ 10.13 (s, 1H), 7.91 (d, *J* = 7.50 Hz, 2H), 7.73 (d, *J* = 7.45 Hz, 2H), 7.43 (t, *J* = 7.35 Hz, 2H), 7.34 (t, *J* = 7.4 Hz, 2H), 4.50 (m, 2H), 4.28 (t, *J* = 6.55 Hz, 1H), 3.56 (dd, *J* = 9.2, 13.2 Hz, 1H), 2.92 (dd, *J* = 5.5, 19.90 Hz, 1H), 2.70 (q, *J* = 6.8, 14.0 Hz, 2H), 1.75 (m, 1H), 1.16 (t, *J* = 7.35 Hz, 3H), .86 (d, *J* = 6.55 Hz, 3H), 0.80 (d, *J* = 6.45 Hz, 3H). ^{13}CNMR (125 MHz, DMSOd_6) δ 172.3, 155.7, 144.5, 144.3, 141.8, 128.7, 128.1, 126.2, 126.1, 121.2, 121.1, 67.0, 56.1, 47.6, 26.8, 24.6, 20.8, 16.1 LRMS m/z calculated for $C_{22}H_{27}N_2O_3S$ [M+H] 399.17 found 399.00. HRMS m/z calculated for $C_{22}H_{27}N_2O_3S$ [M+H] 399.1664 found 399.1735.

(9H-fluoren-9-yl)methyl 2-((ethylthio)carbonyl)pyrazolidine-1-carboxylate (6)

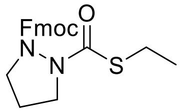

To a solution of (9H-fluoren-9-yl)methyl pyrazolidine-1-carboxylate) CAS: 1093755-81-7 (48 mg, 0.163 mmol) in anhydrous THF (1.0 mL) was added 0.5 M solution of S-ethyl chlorothioformate in DCM (0.32 mL, 0.163 mmol) at 0 °C. Then the reaction mixture was treated with pyridine (13 µL, 0.0163 mmol). The reaction mixture was brought up to rt gradually in 1.0 h. The reaction mixture was stopped by adding water (2.0 mL), and the mixture was extracted with EtOAc (2.0 mL x 3). The combined organic layer was washed with brine, dried over anhydrous Na_2SO_4, filtered and evaporated under vacuum. The resulting crude material was subjected to flash chromatography purification using silica gel and gradient of EtOAc/Hexane to give a white solid (53 mg, 87% yield). With purity of 91.1% based on HPLC, RT = 9.246 min (flow rate of 1 mL/min with a linear gradient from water (0.05% TFA): AcCN (0.05% TFA) [75:25] to water (0.05%TFA): AcCN (0.05% TFA) [10:90] in 14 min, monitored /detected UV at 254 nM by photodiode Array Detector. ^{1}HNMR (500 MHz, DMSO-d_6) *d* 7.91 (d, *J* = 7.55 Hz, 2H), 7.68 (m, 2H), 7.43 (m, 2H), 7.33 (m, 2H), 4.52 (m, 2H), 4.30 (t, *J* = 6.5 Hz, 1H), 3.93 (m, 1H), 3.69 (m, 1H), 3.03 (m, 2H), 2.73 (m, 2H), 1.95 (m, 1H), 1.81 (m, 1H), 1.18 (t, *J* = 7.40 Hz, 3H); ^{13}C NMR (125 MHz, DMSO-d_6) *d* 175.1, 158.3, 144.4, 144.2, 141.8, 128.7, 128.1, 127.7, 126.2, 126.1, 121.1, 120.8, 68.6, 47.9, 47.5, 47.2, 25.9, 24.5, 15.90. LRMS (ESI, M+Na) m/z calc for $C_{21}H_{22}N_2O_3SNa$ 405.12, found 405.3; HRMS (ESI, M+Na) m/z calc for $C_{21}H_{22}N_2O_3SNa$ 405.1249, found 405.1241

(9H-fluoren-9-yl) methyl 2-benzyl-2-((ethylthio)carbonyl) hydrazine-1-carboxylate (7)

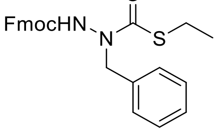

This compound was made according to the general procedure and collected as white fluffy powder in 83% yield. With purity of 94.4% based on HPLC, RT = 9.90 min (flow rate of 1 mL/min with a linear gradient from water (0.05% TFA): AcCN (0.05% TFA) [75:25] to water (0.05%TFA): AcCN (0.05% TFA) [10:90] in 14 min, monitored /detected UV at

254 nM by photodiode Array Detector. ^{1}HNMR (500MHz, DMSO-d_6) δ 10.15 (bs, 1H), 7.90 (d, *J* = 6.3 Hz, 2H), 7.70 (m, 2H), 7.42 (t, *J* = 6.2 Hz, 2H), 7.34-7.14 (m, 7H), 4.62 (ABq, *J* = 11.9 Hz, $\Delta\ \delta$ = 0.93 ppm, 2H), 4.45 (m, 2H), 4.25 (m, 1H), 2.75 (m, 2H), 1.19 (t, *J* =5.6 Hz, 3H). ^{13}CNMR (125 MHz, DMSOd_6) δ 172.5, 155.8, 144.4, 144.3, 141.8, 136.9, 129.3, 128.7, 128.5, 128.0, 126.3, 121.1, 67.1, 55.9, 52.8, 47.5, 24.7, 16.0 LRMS m/z calculated for $C_{25}H_{24}N_2O_3S$ [M+H] 433.16 found 433.00. HRMS m/z calculated for $C_{25}H_{24}N_2O_3S$ [M+H] 433.1508 found 433.1579

tert-butyl 3-((2-(((9H-fluoren-9-yl)methoxy)carbonyl)-1-((ethylthio)carbonyl)hydrazineyl)methyl)-1H-indole-1-carboxylate (8a)

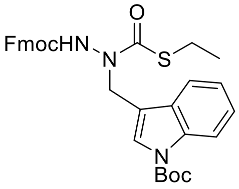

This compound was made according to the general procedure and collected as white fluffy powder in 88% yield. With purity of 94.7% based on HPLC, RT = 11.88 min (flow rate of 1 mL/min with a linear gradient from water (0.05% TFA): AcCN (0.05% TFA) [75:25] to water (0.05%TFA): AcCN (0.05% TFA) [10:90] in 14 min, monitored /detected UV at 254 nM by photodiode Array Detector. ^{1}HNMR (500MHz, DMSO-d_6) δ 10.13 (s, 1H), 8.10 (m, 1H), 7.94 (m, 2H), 7.77-7.59 (m, 4H), 7.46 (t, *J* = 5.7 Hz, 2H), 7.39-7.29 (m, 4H), 5.27 (m, 1H), 4.50-4.18 (m, 4H), 3.42 (m, 1H), 2.83 (m, 2H), 1.27 (s, 9H), 1.24 (m, 3H). ^{13}CNMR (125 MHz, DMSOd_6) δ 172.4, 155.8, 149.9, 144.3, 144.3, 141.7, 135.9, 130.3, 128.7, 128.1, 128.0, 126.7, 126.2, 126.1, 125.7, 123.7, 121.1, 116.1, 115.7, 84.8, 67.4, 60.7, 47.5, 43.3, 28.6, 24.7, 21.7, 16.1. LRMS m/z calculated for $C_{32}H_{33}N_3O_5SNa$ [M+Na] 594.20 found 594.25; HRMS m/z calculated for $C_{32}H_{33}N_3O_5SNa$ [M+Na] 594.2039 found 594.2031

Benzyl 3-((2-(((9*H*-fluoren-9-yl)methoxy)carbonyl)-1-((ethylthio)carbonyl)hydrazineyl)methyl)-1*H*-indole-1 carboxylate (8b)

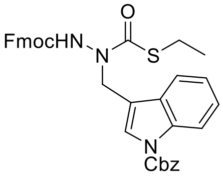

Triethylamine (2.0 mL, 15.0 mmol, 1.50 equiv) was added via syringe to a solution of 3-formylindole (14.5 g, 10.0 mmol, 1 equiv) and 4-dimethylaminopyridine (122 mg, 1.0 mmol, 0.100 equiv) in dichloromethane (50.0 mL) at 23 °C. Benzyl chloroformate (2.0 mL, 14.0 mmol, 1.40 equiv) was added dropwise to the solution via syringe. After 1h, the reaction mixture was diluted with dichloromethane (100 mL) and washed with saturated aqueous sodium bicarbonate solution (50 mL). The aqueous layer was further extracted with dichloromethane (50 mL). The combined organic layers were washed with aqueous 10% HCl (50.0 mL) and the resulting aqueous layer was extracted with dichloromethane (2 × 25 mL). The combined organic layers were dried over anhydrous sodium sulfate, were filtered, and were concentrated under reduced pressure. The resulting aldehyde (10.0 mmol) was mixed with Fmoc-hydrazide (CAS: 35661-51-9) (2.4 g, 9.5 mmol) in DCM (40 mL) and THF (40 mL). The reaction mixture was heated to reflux for 30 mins, then the volatiles were removed under vacuum to give the corresponding hydrazone, which was used without further purifications (10.0 mmol). The crude material was dissolved in DCM (25.0 mL), and MeOH (9.0 m). The mixture was treated with dimethylamine borane (DMAB) (928.0 mg, 16.0 mmol) and PTSA (9.6 g, 50.0 mmol) at 0 C. The reaction mixture was stirred at 0 C for 30 min then was stopped by adding MeOH (20 mL) and 10% Na_2CO_3 Solution (100 mL). The resultant mixture was heated to reflux for 30 min, then, the aqueous layer was extracted with EtOAc. The organic layer was evaporated under vacuum and the resulting crude was purified using silica gel and gradient of EtOAC/Hexane (94% yield). The resulting substituted hydrazine form the previous step (1.8 g, 3.5 mmol) was dissolved in THF (20.0 mL) and cooled down to 0°C using ice bath. To the solution was added 0.5 M solution of S-ethyl chlorothioformate in DCM (7.0 mL, 3.5 mmol) followed by dropwise addition of pyridine (304.0 uL, 3.85 mmol) in 1.0 mL THF solution. The reaction mixture was stirred at this temperature for 30 mins, then, was stopped by adding 20.0 mL water. The aqueous layer was extracted with DCM (20.0 mL x 3). The combined organic layer was washed with brine 20.0 mL, dried over Na_2SO_4, filtered, and evaporated under vacuum. The crude material was purified by CombiFlash® Teledyne Isco Chromatography and gradient of EtOAc: Hexane to give the Fmoc-azaTrp(Cbz)-SEt **8b** (1.75 g, 83% yield). The product is 93.5 % pure based on HPLC, RT = 13.47 min (flow rate of 1 mL/min with a linear gradient from water (0.05% TFA): AcCN (0.05% TFA) [75:25] to water (0.05%TFA): AcCN (0.05% TFA) [10:90] in 14 min, monitored /detected UV at 254 nM by

photodiode Array Detector. ^{1}HNMR (600MHz, DMSO-d_6) δ 10.12 (s, 1H), 8.15 (m, 1H), 7.90 (m, 2H), 7.71-7.54 (m, 6H), 7.45-7.31 (m, 6H), 7.31 (m, 3H), 5.51 (m, 2H), 5.29 (m, 1H), 4.49-4.26 (m, 4H), 2.82 (m, 2H), 1.23 (t, J = 8.0 Hz, 3H). ^{13}CNMR (125 MHz, DMSOd_6) δ 172.4, 155.8, 151.0, 144.3, 141.7, 136.2, 136.0, 130.2, 129.5, 129.3, 128.6, 127.9, 126.3, 126.0, 125.7, 124.0, 121.0, 120.5, 116.9, 115.6, 69.3, 67.3, 47.5, 43.3, 24.7, 16.0. HRMS m/z calculated for $C_{35}H_{31}N_3O_5SNa$ [M+Na] 628.1882 found 628.1878.

(9H-fluoren-9-yl)methyl 2-((ethylthio)carbonyl)-2-(2-oxo-2-(tritylamino)ethyl)hydrazine-1-carboxylate (9)

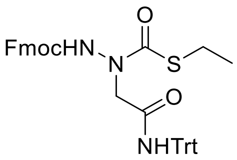

This compound was made according to the general procedure and collected as white fluffy powder in 58% yield. With purity of 98.9% based on HPLC, RT = 11.46 min (flow rate of 1 mL/min with a linear gradient from water (0.05% TFA): AcCN (0.05% TFA) [75:25] to water (0.05%TFA): AcCN(0.05% TFA) [10:90] in 14 min, monitored /detected UV at 254 nM by photodiode Array Detector. ^{1}HNMR (500MHz, DMSO-d_6) δ 10.43 (s, 1H), 8.85 (s, 1H), 7.91 (d, J = 7.55 Hz, 2H), 7.76 (m, 2H), 7.43 (t, J = 7.4 Hz, 2H), 7.32-7.18 (m, 17H), 4.50-4.37 (m, 3H), 4.27 (t, J = 6.8 Hz, 1H), 3.91 (d, J = 17.0 Hz, 1H), 2.75 (q, J = 7.25 Hz, 2H), 1.18 (t, J = 5.3 Hz, 3H). ^{13}CNMR (125 MHz, DMSOd_6) δ 172.6, 167.4, 156.5, 145.6, 144.5, 144.3, 141.7, 129.5, 128.7, 128.5, 128.1, 127.5, 126.4, 121.1, 70.3, 67.7, 54.5, 47.5, 24.7, 16.1 LRMS m/z calculated for $C_{39}H_{35}N_3O_4S$ [M+ H] 641.78 found 641.92. HRMS m/z calculated for $C_{39}H_{35}N_3O_4SNa$ [M+Na] 664.2246 found 664.2237.

(9H-fluoren-9-yl)methyl 2-(4-(tert-butoxy)benzyl)-2-((ethylthio)carbonyl)hydrazine-1-carboxylate (10)

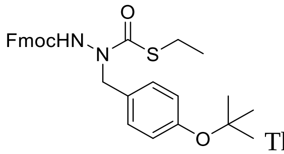

This compound was made according to the general procedure and collected as white fluffy powder in 92% yield. With purity of 98.5% based on HPLC, RT = 10.96 min (flow rate of 1 mL/min with a linear gradient from water (0.05% TFA): AcCN (0.05% TFA) [75:25] to water (0.05%TFA): AcCN (0.05% TFA) [10:90] in 14 min, monitored /detected UV at 254 nM by photodiode Array Detector. ^{1}HNMR (500MHz, DMSO-d_6) δ 10.15 (s, 1H), 7.91 (d, J = 7.5 Hz, 2H), 7.71 (d, J = 7.5 Hz, 2H), 7.43 (t, J = 7.5 Hz, 2H), 7.34 (t, J = 7.5 Hz, 2H), 7.11 (d, J = 8.0 Hz, 2H), 6.91 (d, J = 8.2 Hz, 2H), 4.56 (ABq, J = 14.9 Hz, $\Delta\ \delta$= 0.89 ppm, 2H), 4.49-4.40 (m, 2H), 4.26 (m, 1H), 2.75 (m, 2H), 1.27 (s, 9H), 1.19 (t, J =7.3 Hz, 3H). ^{13}CNMR (125 MHz, DMSOd_6) δ 172.4, 155.5, 144.4, 141.8, 131.3, 130.3, 128.7, 128.1, 128.0, 126.3, 126.1, 124.4, 121.1, 78.9, 67.2, 52.2, 47.5, 29.5, 24.7, 16.0 LRMS m/z calculated for $C_{29}H_{33}N_2O_4S$ [M+H] 505.22 found N.A. HRMS m/z calculated for $C_{29}H_{33}N_2O_4SNa$ [M+Na] 527.1980 found 527.1975.

(9H-fluoren-9-yl)methyl 2-((ethylthio)carbonyl)-2-(3-oxo-3-(tritylamino)propyl)hydrazine-1-carboxylate (11)

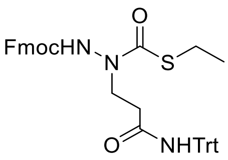

This compound was made from Fmoc azaE thiocarbazate (**12)** according to the following modified procedure and collected as white fluffy powder in 62% yield. To a solution of **12** (635 mg, 1.35 mmol) in DCM (2.0 mL) was added TFA (2.0 mL). The reaction mixture was stirred at rt for 30, then the volatiles were removed under vacuum. The crude acid was dissolved in DCM (5.0 mL) and then treated with thionyl chloride (1.0 mL) and one drop DMF at 0 ˚C. The reaction mixture was warmed to rt in 30 min, then all volatiles were removed under vacuum. The crude acyl chloride was dissolved in DCM (10 mL) and added slowly to a 0.05% Na_2CO_3 solution mixed with NH_4OH (255 uL, 2.03 mmol). The biphasic solution was stirred vigorously at 0 °C until a white copious ppt was observed. The formed ppt was collected by filtration, washed with water and hexanes, and then allowed to dry under high vacuum for 2 h. The dried white solid was dissolved in benzene (10 mL) and treated with triphenyl methanol (351 mg, 1.35 mmol) and PTSA (85.0 mg, 0.45 mmol), the reaction mixture was heated to 90 °C and azeotroped using Dean-Stark tube. The reaction was stopped after the disappearance of the SM based on TLC (90 min). The resulting crude material was purified on silica using gradient EtOAc/Hexanes to give 11 as white solid (550 mg, 62% yield over 4 steps). With purity of 94.6% based on HPLC, RT = 20.89 min (flow rate of 0.8

mL/min with a linear gradient from water (0.05%TFA): MeOH (0.05% TFA) [95: 5] to (0.05%TFA): MeOH (0.05% TFA) [5:95] in 20 min, monitored /detected UV at 254 nM by photodiode Array Detector. ^{1}HNMR for **11** (500MHz. DMSO-*d6*) *δ* 10.0 (s, 1H), 8.66 (s, 1H), 7.90 (m, 2H), 7.70 (m, 2H), 7.43-7.16 (m 19H), 4.56-4.45 (m, 2H), 4.28 (m, 1H), 3.89 (m, 1H), 3.33 (m, 1H), 2.72 (m, 2H), 2.45 (m, 2H), 1.16 (t, *J* = 7.3 Hz, 3H). ^{13}CNMR (125 MHz, DMSO-*d6*) δ 172.0, 170.0, 155.9, 145.6, 144.3, 144.3, 141.7, 129.4, 128.6, 128.4, 128.0 127.2, 126.0, 121.1, 70.2, 67.2, 47.5, 46.1, 34.9, 24.5, 16.0; LRMS m/z calculated for $C_{40}H_{37}N_3O_4S$ [M+H] 655.26 found 656.08.

(9H-fluoren-9-yl)methyl 2-(3-(tert-butoxy)-3-oxopropyl)-2-((ethylthio)carbonyl)hydrazine-1-carboxylate (12)

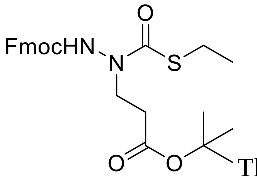

This compound was made according to the general procedure and collected as white fluffy powder in 80% yield. With purity of 92.6% based on HPLC, RT = 10.06 min (flow rate of 1 mL/min with a linear gradient from water (0.05% TFA): AcCN (0.05% TFA) [75:25] to water (0.05%TFA): AcCN(0.05% TFA) [10:90] in 14 min, monitored /detected UV at 254 nM by photodiode Array Detector. ^{1}HNMR (500MHz, DMSO-d_6) δ 10.09 (s, 1H), 7.91 (d, *J* = 7.91 Hz, 2H), 7.73 (d, *J* = 7.55 Hz, 2H), 7.43 (t, *J* = 7.4 Hz, 2H), 7.33 (t, *J* = 7.36 Hz, 2H), 4.53-4.43 (m, 2H), 4.28 (t, *J* = 6.55Hz, 1H), 3.90 (m, 1H), 3.37 (m, 1H), 2.70 (m, 2H), 2.41 (t, *J* = 7.2 Hz, 1H), 1.41 (s, 9H), 1.16 (m, 3H). ^{13}CNMR (125 MHz, DMSOd_6) δ 172.1, 170.9, 156.0, 144.5, 144.3, 141.8, 128.7, 128.1, 126.2, 126.1, 121.2, 81.1, 67.2, 60.7, 47.6, 45.0, 33.7, 28.7, 24.6, 21.7, 16.0. LRMS m/z calculated for $C_{25}H_{30}N_2O_5SNa$ [M+Na] 493.18 found 493.17. HRMS m/z calculated for $C_{25}H_{30}N_2O_5SNa$ [M+Na] 493.1773 found 493.1768.

(9H-fluoren-9-yl)methyl 2-(2-(tert-butoxy)-2-oxoethyl)-2-((ethylthio)carbonyl)hydrazine-1-carboxylate (13)

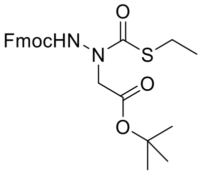

This compound was made according to the general procedure and collected as white fluffy powder in 74% yield. With purity of 93.4% based on HPLC, RT = 10.02 min (flow rate of 1 mL/min with a linear gradient from water (0.05% TFA): AcCN (0.05% TFA) [75:25] to water (0.05%TFA): AcCN (0.05% TFA) [10:90] in 14 min, monitored /detected UV at 254 nM by photodiode Array Detector. ^{1}HNMR (500MHz, DMSO-d_6) δ 10.38 (s, 1H), 7.89 (d, *J* = 6.3 Hz, 2H), 7.74 (m, 2H), 7.43 (t, *J* = 6.0 Hz, 2H), 7.33 (t, *J* = 6.0 Hz, 2H), 4.54-4.33 (m, 2H), 4.27 (t, *J* = 5.8 Hz, 1H), 3.77-3.44 (m, 2H), 2.73 (m, 2H), 1.43 (s, 9H), 1.17 (t, *J* = 5.3 Hz, 3H). ^{13}CNMR (125 MHz, DMSOd_6) δ 172.0, 156.5, 155.9, 144.5, 141.8, 130.4, 128.7, 128.1, 126.2, 126.1, 121.1, 78.3, 67.1, 60.7, 48.7, 47.6, 29.2, 27.5, 24.8, 24.5, 16.1; LRMS m/z calculated for $C_{24}H_{28}N_2O_5SNa$ [M+Na] 479.16 found 479.25.

(9H-fluoren-9-yl)methyl 2-((ethylthio)carbonyl)-2-((1-trityl-1*H*-imidazol-4-yl)methyl)hydrazine-1 carboxylate (14)

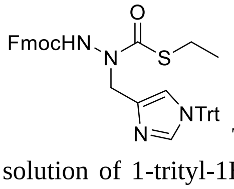

To a solution of Fmoc-hydrazide (CAS: 35661-51-9) (254 mg, 1.0 mmol) in DCM (20 mL) was added a solution of 1-trityl-1H-imidazole-4-carbaldehyde CAS: 33016-47-6) (338 mg, 1.0 mmol) in DCM (5.0 mL). The reaction mixture was heated to reflux for 30 mins, then the volatiles were removed under vacuum to give the corresponding hydrazone, which was used without further purifications (460 mg, 0.8 mmol). The crude material was dissolved in CH_3CN (15.0 mL), and was treated with dimethylamine borane (DMAB) (186.0 mg, 3.2 mmol) and camphor-10-sulfonic acid (CSA) (743.36 mg, 3.2 mmol) at rt. The reaction mixture was stirred at rt for 30 min then was stopped by adding saturated solution of $NaHSO_3$ (20 mL). The resultant mixture was heated to reflux for 30 min, then, the aqueous layer was extracted with EtOAc. The organic layer was evaporated under vacuum and the resulting crude was purified using C18 flash chromatography by CombiFlash® Teledyne Isco Chromatography. The isolated fraction was found with 78% purity based on HPLC analysis (290 mg, 49% yield). The resulting substituted hydrazine form the previous step (290 mg, 0.39 mmol) was dissolved in THF (2.0 mL) and cooled down to -10°C using ice/acetone bath. To the solution was added 0.5 M solution of S-ethyl chlorothioformate in DCM

(1.0 mL, 0.5 mmol) followed by dropwise addition of pyridine (40.0 uL, 0.5 mmol) in 1.0 mL THF solution. The reaction mixture was stirred at this temperature for 30 mins, then, was stopped by adding 2.0 mL water. The aqueous layer was extracted with DCM (2.0 mL x 3). The combined organic layer was washed with brine 2.0 mL, dried over Na_2SO_4, filtered, and evaporated under vacuum. The crude material was purified by CombiFlash® Teledyne Isco Chromatography and gradient of EtOAc: Hexane to give azaHis thiocarbazate (**14**) (120 mg, 46% yield). HPLC analysis indicated that the collected product is 78% pure, the resulting product was repurified again using silica and 2% MeOH/DCM as mobile phase, **14** was collected as white amorphous solid (85 mg, 33% yield over 2 purifications). With purity of 87.9% based on HPLC, RT = 8.2 (flow rate of 1 mL/min with a linear gradient from water (0.05% TFA): MeOH (0.05% TFA) [75:25] to water (0.05%TFA): MeOH (0.05% TFA) [10:90] in 14 min, monitored /detected UV at 254 nM by photodiode Array Detector. ^{1}HNMR for **14** (600MHz, DMSO-d6) δ10.04 (s, 1H) 7.89-7.08 (m, 25H), 4.85 (m, 1H), 4.39-4.19 (m, 4H), 2.72 (m, 2H), 1.17 (m, 3H). ^{13}CNMR (125 MHz, DMSO-d6) δ 172.1, 155.9, 154.1, 144.4, 144.2, 142.9, 141.7, 139.3, 139.0, 130.2, 130.1, 129.2, 129.2, 129.0, 128.7, 128.0, 126.3, 126.1, 121.7, 121.2, 121.1, 76.0, 75.8, 47.5, 47.4, 26.7, 24.6, 16.1. LRMS m/z calculated for $C_{41}H_{36}N_4O_3S$ [M+H] 665.25 found 665.17. HRMS m/z calculated for $C_{41}H_{36}N_4O_3S$ [M+H] 665.2508 found 665.2580

(9H-fluoren-9-yl)methyl 2-(4-((tert-butoxycarbonyl)amino)butyl)-2-((ethylthio)carbonyl) hydrazine-1-carboxylate (15)

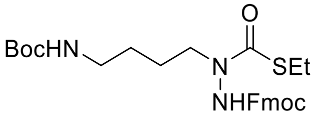

This compound was made according to the general procedure and collected as white fluffy powder in 54% yield. With purity of 87.4% based on HPLC, RT = 9.75 min (flow rate of 1 mL/min with a linear gradient from water (0.05% TFA): AcCN (0.05% TFA) [75:25] to water (0.05%TFA): AcCN(0.05% TFA) [10:90] in 14 min, monitored /detected UV at 254 nM by photodiode Array Detector. ^{1}HNMR (500MHz, DMSO-d_6) δ 10.05 (s, 1H), 7.90 (m, 2H), 7.72 (m, 2H), 7.43 (m, 2H), 7.35 (m, 2H), 6.74 (s, 1H), 4.49 (m, 2H), 4.28 (t, *J* = 5.45 Hz, 1H), 3.73 (m, 1H), 3.09 (m, 1H), 2.91 (m, 2H), 2.70 (m, 2H), 1.38 (m, 13H), 1.16 (m, 3H). ^{13}CNMR (125 MHz, DMSOd_6) δ 172.6, 167.9, 156.0, 144.5, 144.4, 141.7, 128.7, 128.0, 126.3, 126.2, 121.1, 82.4, 67.6, 52.0, 47.5, 28.6, 24.7, 16.0 LRMS m/z calculated for $C_{27}H_{36}N_3O_5S$ [M+H] 514.24 found 513.92. HRMS m/z calculated for $C_{27}H_{36}N_3O_5S$ [M+Na] 536.2195 found 536.2187

(9H-fluoren-9-yl) methyl (E)-8-((tert-butoxycarbonyl) amino)-3-((ethylthio)carbonyl)-12,12-dimethyl-10-oxo-11-oxa-2,3,7,9-tetraazatridec-8-enoate (16)

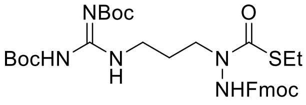

This compound was made according to the general procedure and collected as white fluffy powder in 61% yield. With purity of 96.3% based on HPLC, RT = 12.12 min (flow rate of 1 mL/min with a linear gradient from water (0.05% TFA): AcCN (0.05% TFA) [95:5] to water (0.05%TFA): AcCN(0.05% TFA) [5:95] in 15 min, monitored /detected UV at 254 nM by photodiode Array Detector. ^{1}HNMR (500MHz, DMSO-d_6) δ 11.51 (s, 1H), 10.04 (s, 1H), 8.34 (s, 1H), 7.91 (d, *J* = 7.5 Hz, 2H), 7.71 (d, *J* = 7.35 Hz, 2H), 7.43 (m, 2H), 7.34 (t, *J* = 7.25 Hz, 2H), 4.50 (m, 2H), 4.28 (m, 1H), 3.77 (m, 1H), 3.25 (m, 2H), 3.14 (m, 1H), 2.70 (m, 2H), 1.65 (m, 2H), 1.47 (s, 9H), 1.39 (s, 9H), 1.16 (m, 3H). ^{13}CNMR (125 MHz, DMSOd_6) δ 172.3, 164.0, 156.3, 155.9, 153.0, 144.5, 144.3, 141.8, 128.7, 128.0, 126.1, 121.1, 83.9, 79.2, 67.2, 47.6, 46.5, 38.5, 32.3, 29.2, 28.9, 28.8, 28.6, 27.1, 24.6, 21.7, 16.0. LRMS m/z calculated for $C_{32}H_{44}N_5O_7S$ [M+H] 642.30 found 642.25. HRMS m/z calculated for $C_{32}H_{44}N_5O_7S$ [M+H] 642.2883 found 642.2961.

2.2 Synthesis and characterization of azadipeptide in solution phase (18-34)

General procedure for the synthesis of azadipeptide (18-34)

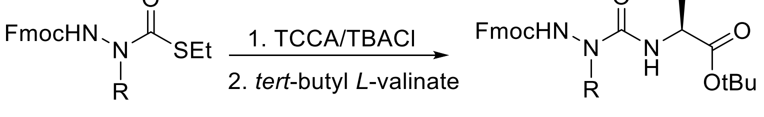

aza thiocarbazates **1-16** | azadipeptides **18-34**

General procedure A (protocol A for synthesis of 18-25 and 29-34):

An appropriate thiocarbazate (0.1 mmol) and TBACl (0.15 mmol) were introduced to a small vial charged with a magnetic stirrer bar. The solids were dissolved entirely in DCM (2.0 mL) and cooled to 0 °C using an ice bath. After 10 min, the

reaction mixture was treated with a freshly pulverized TCCA (0.085 mmol). The reaction mixture was then stirred vigorously at 0 °C for 10 min. At this point, the mixture was treated with an appropriate amino acid ester (tert-butyl L-Valinate) (0.21 mmol) and appropriate tertiary amine (Et_3N) (0.44 mmol) at 0 °C. The ice bath was removed, and the stirring continued until complete conversion based on TLC (35-60 min). The reaction mixture was treated with a saturated solution of $Na_2S_2O_3$ (1.0 mL) and $NaHSO_4$ (1.0 mL). The aqueous layer was extracted with EtOAc (2.0 mL x 3). The combined organic layer was washed with brine (2.0 mL), dried over anhydrous Na_2SO_4, filtered, and evaporated under vacuum. The resulting crude material was then purified using flash chromatography using silica gel and gradient of EtOAc/Hexanes. HPLC analysis is run to confirm the purity before submitting the samples to NMR analysis and HRMS.

General procedure B (protocol B for synthesis of 26-28):
An appropriate thiocarbazate (0.1 mmol) and TBACl (0.15 mmol) were introduced to a small vial charged with a magnetic stirrer bar. The solids were dissolved entirely in DCM (2.0 mL) and cooled to -10 °C using an ice/acetone bath. After 10 min, the reaction mixture was treated with a freshly pulverized TCCA (0.039 mmol). The reaction mixture was then stirred vigorously at -10 °C for 10 min. At this point, the mixture was treated with an appropriate amino acid ester (tert-butyl *L*-Valinate) (0.21 mmol) and appropriate tertiary amine (Et_3N) (0.44 mmol) at 0 °C. The ice bath was removed, and the stirring continued until complete conversion based on TLC (35-60 min). The reaction mixture was treated with a saturated solution of $Na_2S_2O_3$ (1.0 mL) and $NaHSO_4$ (1.0 mL). The aqueous layer was extracted with EtOAc (2.0 mL x 3). The combined organic layer was washed with brine (2.0 mL), dried over anhydrous Na_2SO_4, filtered, and evaporated under vacuum. The resulting crude material was then purified using flash chromatography using silica gel and gradient of EtOAc/Hexanes. HPLC analysis is run to confirm the purity before submitting the samples to NMR analysis and HRMS.

(9*H*-fluoren-9-yl)methyl 2-benzyl-2-((1-(*tert*-butoxy)-3-methyl-1-oxobutan-2-yl)carbamoyl)hydrazine-1-carboxylate 18

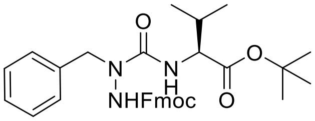

This compound was made according to the procedure A and collected as amorphous solid in 93.7% yield after purification over silica gel using gradient of EtOAc/Hexane R_f(20%EtOAc/Hexane) = 0.27. The purity of the titled compound assigned as 98.3% based on HPLC, RT = 10.46 min (flow rate of 1 mL/min with a linear gradient from water (0.05% TFA): AcCN (0.05% TFA) [75:25]24 to water (0.05%TFA): AcCN (0.05% TFA) [10:90] in 14 min, monitored /detected UV at 254 nM by photodiode Array Detector. ^{1}HNMR (600MHz, DMSO-d6) d 9.67 (m, 1H), 7.93 (d, *J* = 7.5 Hz, 2H), 7.70 (m, 2H), 7.46 (t, *J* = 7.5 Hz, 2H), 7.36-7.29 (m, 5H), 7.23 (d, *J* = 7.3 Hz, 2H), 6.08 (m, 1H), 4.89 (bm, 1H), 4.44 (m, 2H), 4.26 (m, 2H), 4.03 (m, 1H), 2.05 (m, 1H), 1.45 (m, 9H), 0.86 (m, 6H). ^{13}CNMR (125 MHz, DMSO-d6) d 172.4, 171.9, 158.0, 156.1, 144.4, 144.4, 141.7, 141.6, 138.3, 129.1, 128.6, 128.0, 126.1, 121.0, 81.8, 67.1, 59.6, 51.7, 47.5, 31.5, 28.5, 19.7, 18.6. HRMS m/z calculated for $C_{32}H_{37}N_3O_5Na$ [M+Na] 566.2625 found 566.2627.

(9*H*-fluoren-9-yl)methyl 2-((1-(*tert*-butoxy)-3-methyl-1-oxobutan-2-yl)carbamoyl)hydrazine-1-carboxylate (19)

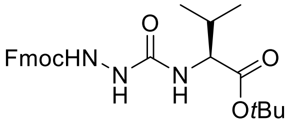

This compound was made according to the procedure A and collected as amorphous solid in 70.6% yield after purification over silica gel using gradient of EtOAc/Hexane R_f (40%EtOAc/Hexane) = 0.24. The purity of the titled compound assigned as 95.6 % based on HPLC, RT = 8.48 min (flow rate of 1 mL/min with a linear gradient from water (0.05% TFA): AcCN (0.05% TFA) [75:25] to water (0.05%TFA): AcCN (0.05% TFA) [10:95] in 14 min, monitored /detected UV at 254 nM by photodiode Array Detector. ^{1}HNMR (600MHz, DMSO-d6) d 9.06 (s, 1H), 7.91 (d, *J* = 7.6 Hz, 2H), 7.89 (bm, 1H), 7.75 (d, *J* = 7.4 Hz, 2H), 7.45 (t, *J* = 7.0 Hz, 2H), 7.36 (t, *J* = 7.4 Hz, 2H), 6.29 (m, 1H), 4.34-4.27 (m, 3H), 4.04 (m, 1H), 2.04 (m, 1H), 1.45 (m, 9H), 0.911 (d, J = 6.8 Hz, 3H), 0.88 (d, J = 6.8 H, 3H). ^{13}CNMR (125 MHz, DMSOd6) d 172.3, 158.7, 157.8, 144.6, 141.6, 128.7, 128.1, 126.3, 121.1, 81.7, 81.9, 67.1, 60.7, 58.9, 55.9, 47.6, 31.7, 28.6, 19.9, 18.6. HRMS m/z calculated for $C_{25}H_{31}N_3O_5Na^+$ [M+Na] 476.2156 found 476.2158.

(9*H*-fluoren-9-yl)methyl 2-((1-(*tert*-butoxy)-3-methyl-1-oxobutan-2-yl)carbamoyl)-2-methylhydrazine-1-carboxylate (20)

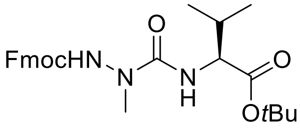 This compound was made according to the procedure A and collected as amorphous solid in 76.6% yield after purification over silica gel using gradient of EtOAc/Hexane R_f (30%EtOAc/Hexane) = 0.12. The purity of the titled compound assigned as 99.8 % based on HPLC, RT = 8.48 min (flow rate of 1 mL/min with a linear gradient from water (0.05% TFA): AcCN (0.05% TFA) [75:25] to water (0.05%TFA): AcCN (0.05% TFA) [10:90] in 14 min, monitored /detected UV at 254 nM by photodiode Array Detector. ^{1} HNMR (600MHz, DMSO-d6) d 9.57 (s, 1H), 7.93 (d, J = 7.6 Hz, 2H), 7.73 (m, 1H), 7.46 (t, J = 7.4 Hz, 2H), 7.37 (t, J = 7.4 Hz, 2H), 6.09 (m, 1H), 4.46 (m, 2H), 4.31 (m, 1H), 3.98 (m, 1H), 2.95 (s, 3H), 2.03 (m, 1H), 1.43 (m, 9H), 0.84 (m, 6H). ^{13}CNMR (125 MHz, DMSO-d6) d 172.1, 158.2, 156.4, 144.5, 144.4, 141.7, 128.6, 128.0, 126.1, 121.1, 81.7, 81.9, 67.1, 60.7, 47.5, 36.3, 31.4, 28.5, 19.7, 18.8. HRMS m/z calculated for $C_{26}H_{33}N_3O_5Na$ [M+Na] 490.2312 found 490.2312.

(9*H*-fluoren-9-yl)methyl 2-((1-(*tert*-butoxy)-3-methyl-1-oxobutan-2-yl)carbamoyl)-2-isopropylhydrazine-1-carboxylate (21)

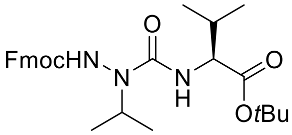 This compound was made according to the procedure A and collected as amorphous solid in 80% yield after purification over silica gel using gradient of EtOAc/Hexane R_f (20%EtOAc/Hexane) = 0.20. The purity of the titled compound assigned as 97.4% based on HPLC, RT = 9.88 min (flow rate of 1 mL/min with a linear gradient from water (0.05% TFA): AcCN (0.05% TFA) [75:25] to water (0.05%TFA): AcCN (0.05% TFA) [10:90] in 14 min, monitored /detected UV at 254 nM by photodiode Array Detector. ^{1} HNMR (600MHz, DMSO-d6) d 9.45 (m, 1H), 7.94 (m, 2H), 7.78 (m, 2H), 7.46 (m, 2H), 7.36 (m, 2H), 5.79 (m, 1H), 4.54 (m, 1H), 4.41 (m, 2H), 4.31 (m, 1H), 4.00 (m,1H), 2.03 (m, 1H), 1.46 (m, 9H), 1.00 (m, 6H), 0.84 (m, 6H). ^{13}CNMR (125 MHz, DMSOd6) d 172.2, 171.8, 157.4, 157.3, 144.4, 141.7, 128.6, 128.0, 126.1, 121.1, 81.7, 67.1, 59.2, 48.3, 47.5, 31.5, 28.6, 20.7, 20.2, 19.7, 18.8. HRMS m/z calculated for $C_{28}H_{37}N_3O_5Na$ [M+Na] 518.2625 found 518.2628.

(9*H*-fluoren-9-yl)methyl 2-((1-(*tert*-butoxy)-3-methyl-1-oxobutan-2-yl)carbamoyl)-2-isobutylhydrazine-1-carboxylate (22)

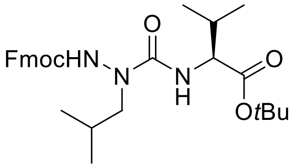 This compound was made according to the procedure A and collected as amorphous solid in 94% yield after purification over silica gel using gradient of EtOAc/Hexane R_f (20%EtOAc/Hexane) = 0.23. The purity of the titled compound assigned as 94.5% based on HPLC, RT = 10.43min (flow rate of 1 mL/min with a linear gradient from water (0.05% TFA): AcCN (0.05% TFA) [75:25] to water (0.05%TFA): AcCN (0.05% TFA) [10:90] in 14 min, monitored /detected UV at 254 nM by photodiode Array Detector. ^{1} HNMR (600MHz, DMSO-d6) d 9.66 (m, 1H), 7.93 (d, J = 7.4 Hz, 2H), 7.74 (m, 2H), 7.46 (t, J = 7.4 Hz, 2H), 7.36 (m, 2H), 5.92 (m, 1H), 4.49 (m, 2H), 4.30 (m, 1H), 3.98 (m,1H), 2.03 (m, 1H), 1.74 (m, 1H), 1.43 (m, 9H), 0.85 (m, 12H). ^{13}CNMR (125 MHz, DMSOd6) d 172.1,158.2, 156.1, 144.4, 141.7, 128.6, 128.0, 126.0, 121.1, 81.7, 66.9, 59.5, 55.1, 47.5, 28.5, 27.0, 20.8, 19.7, 18.6. HRMS m/z calculated for $C_{29}H_{39}N_3O_5Na$ [M+Na] 532.2782 found 532.2785.

(9*H*-fluoren-9-yl)methyl 2-((1-(*tert*-butoxy)-3-methyl-1-oxobutan-2-yl)carbamoyl)-2-(*sec*-butyl)hydrazine-1-carboxylate (23)

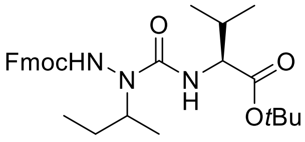 This compound was made according to the procedure A and collected as amorphous solid in 95% yield after purification over silica gel using gradient of EtOAc/Hexane R_f (20%EtOAc/Hexane) = 0.22. The purity of the

titled compound assigned as 99.51% based on HPLC, RT = 10.34 min (flow rate of 1 mL/min with a linear gradient from water (0.05% TFA): AcCN (0.05% TFA) [75:25] to water (0.05%TFA): AcCN (0.05% TFA) [10:90] in 14 min, monitored /detected UV at 254 nM by photodiode Array Detector. ^{1}HNMR (600MHz, DMSO-d6) d 9.34 (m, 1H), 7.93 (m, 2H), 7.78 (m, 2H), 7.46 (m, 2H), 7.36 (m, 2H), 5.79 (m, 1H), 4.49-4.01 (m, 4H), 3.97 (m,1H), 2.02 (m, 1H), 1.43 (m, 9H), 0.97-0.75 (m, 12H). ^{13}CNMR (125 MHz, DMSOd6) d 172.3,157.6, 157.3, 144.6, 144.4, 141.7, 128.6, 128.0, 126.0, 121.1, 81.9, 67.1, 59.4, 54.9, 47.7, 28.6, 27.6, 19.9 18.9, 18.5. HRMS m/z calculated for $C_{29}H_{39}N_3O_5Na$ [M+Na] 532.2782 found 532.2785.

(9*H*-fluoren-9-yl)methyl 2-((1-(*tert*-butoxy)-3-methyl-1-oxobutan-2-yl)carbamoyl)pyrazolidine-1-carboxylate (24)

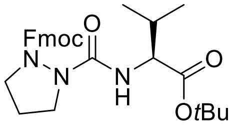

This compound was made according to the procedure A and collected as amorphous solid in 74% yield after purification over silica gel using gradient of EtOAc/Hexane R_f (30%EtOAc/Hexane) = 0.27. The purity of the titled compound assigned as 97.8% based on HPLC, RT = 10.04 min (flow rate of 1 mL/min with a linear gradient from water (0.05% TFA): AcCN (0.05% TFA) [75:25] to water (0.05%TFA): AcCN (0.05% TFA) [10:90] in 14 min, monitored /detected UV at 254 nM by photodiode Array Detector. ^{1}HNMR (600MHz, DMSO-d6) d 7.93 (d, *J* = 7.5 Hz, 2H), 7.70 (t, *J* = 7.3 Hz, 2H), 7.46 (t, *J* = 7.6 Hz, 2H), 7.36 (m, 2H), 6.81 (d, *J* = 8.5 Hz, 1H), 4.45 (m, 2H), 4.32 (t, *J* = 7.0 Hz, 1H), 3.99 (dd, *J* = 6.2, 8.5 Hz, 1H), 3.57 (bm, 2H), 3.10 (bm, 2H), 2.09 (m, 1H), 1.89 (m, 2H),1.41 (m, 9H), 0.89 (d, J = 6.9 Hz, 3H), 0.87 (d, J = 6.8 H, 3H). ^{13}CNMR (125 MHz, DMSOd6) d 171.8, 160.5, 158.4, 144.5, 144.4, 141.7, 128.7, 128.1, 126.2, 121.1, 81.6, 81.9, 68.4, 60.7, 60.1, 47.5, 47.3, 31.0, 28.5, 26.1, 19.9, 19.3. HRMS m/z calculated for $C_{28}H_{35}N_3O_5Na$ [M+Na] 516.2469 found 516.2471.

(9*H*-fluoren-9-yl)methyl 2-((1-(*tert*-butoxy)-3-methyl-1-oxobutan-2-yl)carbamoyl)-2-(4-(*tert*-butoxy)benzyl)hydrazine-1-carboxylate (25)

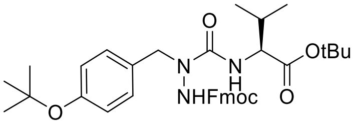

This compound was made according to the procedure A and collected as amorphous solid in 79% yield after purification over silica gel using gradient of EtOAc/Hexane R_f (20%EtOAc/Hexane) = 0.22. The purity of the titled compound assigned as 94.3% based on HPLC, RT = 11.48 min (flow rate of 1 mL/min with a linear gradient from water (0.05% TFA): AcCN (0.05% TFA) [75:25] to water (0.05%TFA): AcCN (0.05% TFA) [10:90] in 14 min, monitored /detected UV at 254 nM by photodiode Array Detector. ^{1}HNMR (600MHz, DMSO-d6) d 7.91 (d, *J* = 7.5 Hz, 2H), 7.69 (m, 2H), 7.45 (t, *J* = 7.6 Hz, 2H), 7.35 (t, *J* = 7.4 Hz, 2H), 7.12 (d, *J* = 8.1 Hz, 2H), 6.90 (d, *J* = 8.2 Hz, 2H), 6.05 (m, 1H), 4.78 (bm, 1H), 4.41 (m, 2H), 4.24 (m, 1H), 4.04 (m, 2H), 2.04 (m, 1H), 1.43 (m, 9H), 1.27 (m, 9H), 0.84 (m, 6H). ^{13}CNMR (125 MHz, DMSOd6) d 172.1, 158.2, 156.2, 155.3, 144.7, 144.5, 143.6, 141.8, 140.4, 138.5, 133.2, 132.8, 130.0, 129.5, 128.8, 128.4, 128.1, 126.2, 124.7, 124.4, 122.4, 121.2, 121.1, 110.8, 82.0, 78.9, 67.3, 59.6, 47.6, 31.6, 29.5, 28.6, 19.8, 18.8. HRMS m/z calculated for $C_{36}H_{45}N_3O_6Na$ [M+Na] 638.3201 found 638.3202.

***tert*-butyl 3-((2-(((9*H*-fluoren-9-yl)methoxy)carbonyl)-1-((1-(*tert*-butoxy)-3-methyl-1-oxobutan-2 yl)carbamoyl)hydrazineyl)methyl)-1*H*-indole-1-carboxylate (26)**

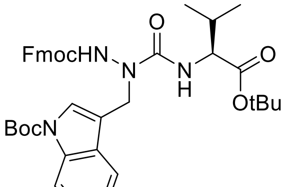

This compound was made according to the procedure B and collected as amorphous solid in 42% (67% b.r.s.m) yield after purification over silica gel using gradient of 20% EtOAc/Hexane R_f = 0.28. The purity of the titled compound assigned as 97.54% based on HPLC, RT = 12.19 min (flow rate of 1 mL/min with a linear gradient from water (0.05% TFA): AcCN (0.05% TFA) [75:25] to water (0.05%TFA): AcCN (0.05% TFA) [10:90] in 14 min, monitored /detected UV at 254 nM by photodiode Array Detector. ^{1}HNMR (600MHz, DMSO-d6) d 9.60 (s, 1H), 8.08 (d, *J* = 8.3 Hz, 1H), 7.91 (d, *J* = 7.6 Hz, 2H), 7.66 (m, 3H), 7.58 (m, 1H), 7.44 (t, *J* = 7.5 Hz, 2H), 7.34 (m, 3H), 7.23 (t, *J* = 7.44 Hz, 2H), 6.09 (m, 1H), 5.05 (bm, 1H), 4.43-4.16 (m, 3H), 4.04 (m, 1H), 2.05 (m, 1H), 1.61 (m, 9H), 1.44 (m, 9H), 0.85 (m, 6H).

^{13}CNMR (125 MHz, DMSOd6) d 172.0, 158.0, 156.2, 150.0, 144.4, 141.7, 136.0, 130.5, 128.7, 128.1, 128.0, 126.2, 126.0, 125.4, 123.5, 121.1, 120.8, 117.6, 115.6, 84.7, 81.9, 67.4, 60.7, 59.8, 47.4, 42.6, 31.5, 28.6, 19.8, 18.7. HRMS m/z calculated for $C_{39}H_{46}N_4O_7Na$ [M+Na] 705.3257 found 705.3257.

benzyl 3-((2-(((9*H*-fluoren-9-yl)methoxy)carbonyl)-1-((1-(*tert*-butoxy)-3-methyl-1-oxobutan-2-yl)carbamoyl)hydrazineyl)methyl)-1*H*-indole-1-carboxylate (27)

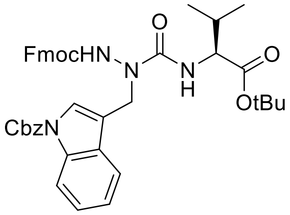

This compound was made according to the procedure B and collected as amorphous solid in 41% (63% b.r.s.m) yield after purification over silica gel using gradient of 20% EtOAc/Hexane R_f = 0.18. The purity of the titled compound assigned as 95.62% based on HPLC, RT = 12.15 min (flow rate of 1 mL/min with a linear gradient from water (0.05% TFA): AcCN (0.05% TFA) [75:25] to water (0.05%TFA): AcCN (0.05% TFA) [10:90] in 14 min, monitored /detected UV at 254 nM by photodiode Array Detector. [1] HNMR (600MHz, DMSO-d_6) δ 9.58 (s, 1H), 8.12 (m, 1H), 7.88 (m, 2H), 7.66 (m, 4H), 7.53 (m, 2H), 7.41 (m, 6H), 7.31 (m, 2H), 7.25 (m, 1H), 6.09 (m, 1H), 5.48 (s, 2H), 5.05 (bm, 1H), 4.38-4.22 (m, 3H), 4.07 (m, 1H), 2.04 (m, 1H), 1.45 (m, 9H), 0.86 (m, 6H). ^{13}CNMR (125 MHz, DMSO-d_6) δ 172.1, 159.0, 157.9, 156.2, 151.1, 144.4, 141.7, 141.6, 136.2, 136.0, 130.4, 129.5, 129.4, 129.3, 128.6, 128.0, 127.9, 126.1, 125.6, 123.8, 121.0, 120.9, 118.3, 115.5, 81.9, 69.3, 67.2, 60.7, 59.7, 47.4, 42.5, 31.5, 28.5, 19.8, 18.7. HRMS m/z calculated for $C_{42}H_{44}N_4O_7Na$ [M+Na] 739.3102 found 739.3106.

(9*H*-fluoren-9-yl)methyl 2-((1*H*-indol-3-yl)methyl)-2-((1-(*tert*-butoxy)-3-methyl-1-oxobutan-2-yl)carbamoyl)hydrazine-1-carboxylate (110)

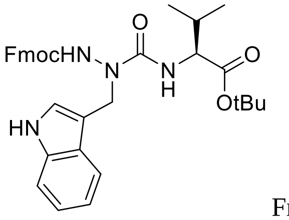

Fmoc-azaW(Cbz)V(OtBu) **27** (28 mg, 0.04 mmol) was introduced into a clean vial charged with a magnetic stirrer; the compound was dissolved in THF (100 uL) and EtOH (100 uL). Pd black (14 mg, 50% wt/wt) was added to the mixture, followed by the subsequent addition of Et_3SiH (130 ul, 0.8 mmol). Upon adding the silane, the vial was capped and secured tightly, and the reaction mixture was stirred vigorously at rt for 30 min. (**Cation: the reaction produces heat and excessive H_2 gas within 30 seconds of the silane addition**). When H_2 gas completely stops being released (around 45 min), the vial was opened carefully to monitor the reaction by TLC. Later, the mixture was filtered over a celite pad, and the excess solvent was evaporated to dryness. The crude material was purified using 40% EtOAc/Hexane (R_f = 0.48). The pure fraction was collected (17.5 mg, 77%) and analyzed by mass spec and NMR. [1] HNMR (600MHz, DMSO-d6) d 10.98 (s, 1H), 9.49 (s, 1H), 7.93 (m, 1H), 7.70 (m, 2H), 7.58 (d, J = 7.7 Hz, 1H), 7.45 (m, 2H), 7.39 (d, J = 8.0 Hz, 1H), 7.35 (m, 2H), 7.18 (m, 1H), 7.10 (m, 1H), 6.98 (m, 1H), 5.98 (bs, 1H), 5.19 (bm, 1H), 4.52-4.19 (m, 3H), 4.07 (m, 1H), 2.03 (m, 1H), 1.45 (m, 9H), 0.85 (m, 6H). ^{13}CNMR (125 MHz, DMSO-d6) d 172.2, 157.9, 156.2, 144.4, 141.7, 137.3, 129.8, 129.6, 128.2, 128.0, 127.9, 126.2, 126.1, 122.0, 121.0, 119.8, 119.5, 112.3, 111.0, 81.8, 67.2, 59.5, 47.4, 42.5, 31.5, 29.9, 28.5, 19.7, 18.6. LRMS m/z calculated for $C_{34}H_{39}N_4O_5$ [M+H] 583.29 found 583.08.

(9*H*-fluoren-9-yl)methyl 2-((1-(*tert*-butoxy)-3-methyl-1-oxobutan-2-yl)carbamoyl)-2-((1-trityl-1*H*-imidazol-4-yl)methyl)hydrazine-1-carboxylate (28)

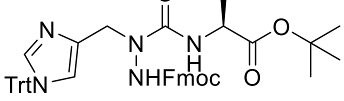

This compound was made according to the procedure B and collected as amorphous solid in 27% yield after purification over silica gel using gradient of $CHCl_3$/MeOH R_f (2% MeOH/$CHCl_3$) = 0.24. The purity of the titled compound assigned as 91.31% based on HPLC, RT = 9.00 min (flow rate of 1 mL/min with a linear gradient from

water (0.05% TFA): AcCN (0.05% TFA) [75:25] to water (0.05%TFA): AcCN (0.05% TFA) [10:90] in 14 min, monitored /detected UV at 254 nM by photodiode Array Detector. ^{1}HNMR (600MHz, DMSO-d6) δ 9.55 (m, 1H), 8.35 (s, 1H), 7.89 (m, 2H), 7.70 (m, 2H), 7.40 (m, 12H), 7.29 (m, 3H), 7.11 (m, 6H), 6.81 (m, 1H), 6.19 (m, 1H), 4.59 (bm, 1H), 4.32 (m, 2H), 4.23 (m, 2H), 4.01 (m, 1H), 2.03 (m, 1H), 1.43 (m, 9H), 0.86 (m, 6H). ^{13}CNMR (125 MHz, DMSOd6) δ 172.0, 158.0, 156.3, 144.4,144.3, 143.1, 141.6, 138.9, 137.2, 130.2, 129.8, 129.2, 129.0, 128.8, 128.6, 128.2, 128.0, 126.1, 122.3, 121.0, 120.9, 81.8, 80.1, 75.5, 67.3, 59.6, 50.1, 47.4, 31.6, 28.5, 19.7, 18.7. HRMS m/z calculated for $C_{48}H_{49}N_5O_5Na$ [M+Na] 798.3626 found 798.3636.

(9*H*-fluoren-9-yl)methyl 2-(2-(*tert*-butoxy)-2-oxoethyl)-2-((1-(*tert*-butoxy)-3-methyl-1-oxobutan-2-yl)carbamoyl)hydrazine-1-carboxylate (29)

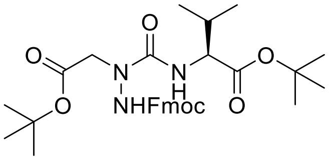

This compound was made according to the procedure A and collected as amorphous solid in 81% yield after purification over silica gel using gradient of EtOAc/Hexane R_f(20%EtOAc/Hexane) = 0.32. The purity of the titled compound assigned as 98.8% based on HPLC, RT = 10.76 min (flow rate of 1 mL/min with a linear gradient from water (0.05% TFA): AcCN (0.05% TFA) [75:25] to water (0.05%TFA): AcCN (0.05% TFA) [10:90] in 14 min, monitored /detected UV at 254 nM by photodiode Array Detector. ^{1}HNMR (600MHz, DMSO-d6) δ 9.77 (bs, 1H), 7.93 (d, *J* = 7.7 Hz, 2H), 7.74 (bm, 2H), 7.46 (d, *J* = 7.4 Hz, 2H), 7.35 (t, *J* = 7.0 Hz, 2H), 6.24 (m, 1H), 4.41 (m, 2H), 4.30 (t, *J* = 6.06 Hz, 1H), 3.98 (m, 1H), 2.03 (m, 1H), 1.46 (m, 9H), 1.43 (m, 9H), 0.85 (m, 6H). ^{13}CNMR (150 MHz, DMSOd6) δ 171.8, 169.1, 157.6, 156.1, 144.4, 141.7, 141.6, 128.7, 128.2, 128.0, 126.2, 121.1, 81.9, 67.5, 59.6, 47.4, 31.5, 28.6, 28.5, 19.6, 18.6. HRMS m/z calculated for $C_{31}H_{41}N_3O_7Na$ [M+Na] 590.2837 found 590.2840.

(9*H*-fluoren-9-yl)methyl 2-((1-(*tert*-butoxy)-3-methyl-1-oxobutan-2-yl)carbamoyl)-2-(3-(*tert*-butoxy)-3-oxopropyl)hydrazine-1-carboxylate (30)

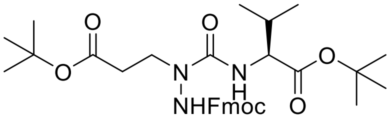

This compound was made according to the procedure A and collected as amorphous solid in 83% yield after purification over silica gel using gradient of EtOAc/Hexane R_f(20%EtOAc/Hexane) = 0.19. The purity of the titled compound assigned as 96.8% based on HPLC, RT = 10.82 min (flow rate of 1 mL/min with a linear gradient from water (0.05% TFA): AcCN (0.05% TFA) [75:25] to water (0.05%TFA): AcCN (0.05% TFA) [10:90] in 14 min, monitored /detected UV at 254 nM by photodiode Array Detector. ^{1}HNMR (600MHz, DMSO-d6) δ 9.59 (bs, 1H), 7.93 (d, J = 7.6 Hz, 2H), 7.74 (m, 2H), 7.46 (t, *J* = 7.4 Hz, 2H), 7.36 (t, *J* = 7.3 Hz, 2H), 6.10 (m, 1H), 4.47 (m, 2H), 4.30 (m, 1H), 3.97 (m, 1H), 3.6 (bm, 2H), 2.41 (m, 2H), 2.03 (m, 1H), 1.45 (m, 18H), 0.84 (m, 6H). ^{13}CNMR (150 MHz, DMSO-d6) δ 171.9, 171.3, 157.7, 156.4, 144.4, 144.3, 141.6, 128.6, 127.9, 126.0, 121.0, 81.7, 80.8, 67.1, 59.6, 47.5, 34.3, 31.4, 28.6, 28.5, 19.7, 18.7. HRMS m/z calculated for $C_{32}H_{43}N_3O_7Na$ [M+Na] 604.2993 found 604.2996.

***tert*-butyl 5-((((9*H*-fluoren-9-yl)methoxy)carbonyl)amino)-2-isopropyl-13,13-dimethyl-4,11-dioxo-12-oxa-3,5,10-triazatetradecanoate (31)**

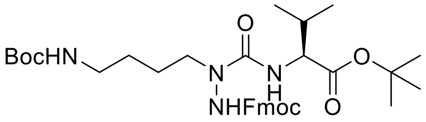

This compound was made according to the procedure A and collected as amorphous solid in 61% yield after purification over silica gel using gradient of EtOAc/Hexane R_f(30%EtOAc/Hexane) = 0.14. The purity of the titled compound assigned as 95.89% based on HPLC, RT = 10.48 min (flow rate of 1 mL/min with a linear gradient from water (0.05% TFA): AcCN (0.05% TFA) [75:25] to water (0.05%TFA): AcCN (0.05% TFA) [10:90] in 14 min, monitored /detected UV at 254 nM by photodiode Array Detector. ^{1}HNMR (600MHz, DMSO-d6) d 9.64 (bs, 1H), 7.93 (d, *J* = 7.4 Hz, 2H), 7.74 (m, 2H), 7.46 (t, *J* = 7.4 Hz, 2H), 7.37 (m, 2H), 6.76 (t, *J* = 5.1 H, 1H), 5.96 (bs, 1H), 4.47 (m, 2H), 4.30 (m, 1H), 3.98 (m, 1H), 3.60 (bm, 1H), 2.93 (m, 2H), 2.03 (m, 1H), 1.43 (m, 22H), 0.84 (m, 6H). ^{13}CNMR (125 MHz, DMSOd6) d 172.1, 157.9, 156.5, 156.3, 144.5, 144.4, 141.7, 128.6, 128.0, 126.0, 121.1, 81.8, 78.2, 67.0, 59.5, 47.5, 31.5, 29.9, 29.2, 28.5, 27.6, 19.7, 18.7. HRMS m/z calculated for $C_{34}H_{48}N_4O_7Na$ [M+Na] 647.3415 found 647.3422.

tert-butyl (_Z_)-11-((((9_H_-fluoren-9-yl)methoxy)carbonyl)amino)-6-((_tert_-butoxycarbonyl)amino)-14-isopropyl-2,2-dimethyl-4,12-dioxo-3-oxa-5,7,11,13-tetraazapentadec-5-en-15-oate (32)

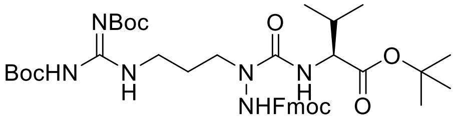

This compound was made according to the procedure A and collected as amorphous solid in 63% yield after purification over silica gel using gradient of EtOAc/Hexane R_f(30%EtOAc/Hexane) = 0.24. The purity of the titled compound assigned as 98.83% based on HPLC, RT = 9.82 min (flow rate of 1 mL/min with a linear gradient from water (0.05% TFA): AcCN (0.05% TFA) [75:25] to water (0.05%TFA): AcCN (0.05% TFA) [10:90] in 14 min, monitored /detected UV at 254 nM by photodiode Array Detector. ^{1}HNMR (600MHz, DMSO-d6) δ 11.48 (s, 1H), 9.57 (bs, 1H), 8.34 (m, 1H), 7.89 (d, *J* = 7.6 Hz, 2H), 7.69 (m, 2H), 7.42 (t, *J* = 7.4 Hz, 2H), 7.32 (t, *J* = 7.4 Hz, 2H), 6.00 (m, 1H), 4.47 (m, 2H), 4.27 (m, 1H), 3.96 (m, 1H), 3.63 (bm, 1H), 3.25 (m, 2H), 2.99 (bm, 1H), 1.99 (m, 1H), 1.60 (m, 2H), 1.47 (m, 9H), 1.39 (m, 18H), 0.84 (m, 6H). ^{13}CNMR (125 MHz, DMSO-d6) δ 172.0, 164.0, 163.9, 158.1, 156.3, 156.2, 156.1, 156.0, 155.9, 152.9, 152.8, 144.4, 141.7, 129.8, 128.6, 128.2, 128.0, 126.0, 122.3, 121.1, 120.5, 83.8, 81.8, 79.1, 67.1, 59.6, 47.5, 45.3, 38.4, 31.5, 28.9, 28.5, 27.4, 19.7, 18.7. HRMS m/z calculated for $C_{39}H_{56}N_6O_9Na$ [M+Na] 775.4001 found 775.4009.

(9_H_-fluoren-9-yl)methyl 2-((1-(_tert_-butoxy)-3-methyl-1-oxobutan-2-yl)carbamoyl)-2-(2-oxo-2-(tritylamino)ethyl)hydrazine-1-carboxylate (33)

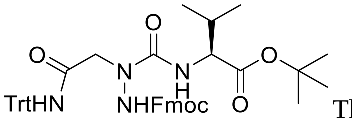

This compound was made according to the procedure A and collected as amorphous solid in 84% yield after purification over silica gel using gradient of EtOAc/Hexane R_f(50%EtOAc/Hexane) = 0.39. The purity of the titled compound assigned as 99.3% based on HPLC, RT = 12.9 min (flow rate of 1 mL/min with a linear gradient from water (0.05% TFA): AcCN (0.05% TFA) [75:25] to water (0.05%TFA): AcCN (0.05% TFA) [10:90] in 14 min, monitored /detected UV at 254 nM by photodiode Array Detector. ^{1}HNMR (600MHz, DMSO-d6) δ 9.83 (bs, 1H), 8.79 (bs, 1H), 7.94 (d, *J* = 7.6 Hz, 2H), 7.73 (m, 2H), 7.46 (d, *J* = 7.4 Hz, 2H), 7.357.23 (m, 17H), 6.42 (m, 1H), 4.45 (m, 2H), 4.30 (m, 1H), 4.04 (m, 1H), 3.97 (m, 2H), 2.08 (m, 1H), 1.43 (m, 9H), 0.88 (m, 6H). ^{13}CNMR (125 MHz, DMSOd6) δ 172.0, 168.6, 158.0, 145.6, 144.3, 141.7, 129.4, 128.6, 128.4, 128.0, 127.3, 126.1, 121.1, 81.8, 70.0, 67.5, 59.8, 55.0, 47.5, 31.4, 28.5, 19.8, 18.8. HRMS m/z calculated for $C_{46}H_{48}N_4O_6Na$ [M+Na] 775.3644 found 775.3472.

(9_H_-fluoren-9-yl)methyl 2-((1-(_tert_-butoxy)-3-methyl-1-oxobutan-2-yl)carbamoyl)-2-(3-oxo-3-(tritylamino)propyl)hydrazine-1-carboxylate (34)

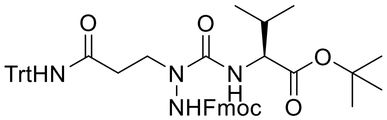

This compound was made according to the procedure A and collected as amorphous solid in 53% yield after purification over silica gel using gradient of EtOAc/Hexane R_f(50%EtOAc/Hexane) = 0.27. The purity of the titled compound assigned as 99.6% based on HPLC, RT = 12.18 min (flow rate of 1 mL/min with a linear gradient from water (0.05% TFA): AcCN (0.05% TFA) [75:25] to water (0.05%TFA): AcCN (0.05% TFA) [10:90] in 14 min, monitored /detected UV at 254 nM by photodiode Array Detector. ^{1}HNMR (600MHz, DMSO-d6) δ 9.55 (s, 1H), 8.69 (s, 1H), 7.93 (d, *J* = 7.5 Hz, 2H), 7.72 (m, 2H), 7.45 (t, *J* = 7.4 Hz, 2H), 7.34 (m, 2H), 7.30 (m, 6H), 7.24-7.20 (m, 9 H), 6.14 (m, 1H), 4.48 (m, 2H), 4.31 (m, 1H), 4.00 (m, 1H), 3.83-3.35 (bm, 2H), 2.51 (m, 2H), 2.01 (m, 1H), 1.42 (m, 9H), 0.82 (m, 6H). ^{13}CNMR (125 MHz, DMSOd6) δ 172.0, 171.2, 157.9, 156.5, 145.7, 144.4, 141.7, 129.5, 128.6, 128.3, 128.0, 127.2, 126.1, 121.1, 81.7, 70.2, 67.2, 59.5, 55.8, 47.5, 35.5, 31.5, 28.5, 19.7, 18.7. HRMS m/z calculated for $C_{47}H_{50}N_4O_6Na$ [M+Na] 789.3623 found 789.3623.

2.3 General procedure for solid phase peptide synthesis (SPPS)

All solid phase peptide couplings were performed at ambient temperature using Tribute® Peptide synthesizer from Gyros Protein Technologies, Inc following standard protocol which is described sequentially below:

1. Swelling: The resin (loaded with the first Fmoc-protected amino acid) was swelled twice successively for 20 min in DMF, each swelling step was followed by drainage and drying step.
2. Fmoc Cleavage: the protected amino acid/or peptide was shaken for 2.0 min with 20% piperidine solution in DMF to remove the Fmoc group. The process was repeated twice, followed by several washing steps with DMF (3-5 times).
3. Amino acid coupling: 5 equivalents of the next acylating component (Fmoc-protected amino acid), 5 equivalents of HATU (coupling reagent), and 10 equivalents of N-methylmorpholine (base) were used to add the next amino acid in the sequence. This step is fully automated and was run according to the software installed on the Tribute® synthesizer. The amino acid including coupling reagent was delivered to the reaction vessel from the specified loading position upon dissolution. Then the base was added as 0.4 M solution in DMF, the total volume of solvent was adjusted to give 0.2 M solution. The coupling time was limited to 15 min shaking followed by drainage then washing steps. Step 2 and step 3 were repeated until the desired peptide sequence was achieved.
4. Washing: repeated washing steps were performed after each cleavage or coupling event using DMF as solvent (2-3 times). At the final coupling or cleavage steps additional washing with DCM was performed (5-6 times) to remove any trace of DMF. The process was usually followed by a drying step.
5. Cleavage from the Resin: 5.0 mL of a freshly made solution of TFA/H_2O/TIPS (95:2.5:2.5. v/v/v) was cooled down to 0 °C and added at 0 °C to a 0.3 mmol of resin. The mixture was shaken for 2 h, filtered, and the remaining resin was further washed with a 0.5-1.0 mL of TFA/H_2O) (95:5,v/v) solution. The filtrate was precipitated by adding 10 mL of 1:1 solution of ether: hexane. Upon centrifugation, the resulting solid was dissolved in a 1:1 solution of CH_3CN: H_2O. The resulting solution was lyophilized.

Purification: Purification of the peptidomimetics were performed on a preparative HPLC purification system (Waters Prep 150 LC system combining 2545 Binary Gradient Module using XSelect Peptide CSH C18 OBD Prep Column, 130Å, 5 µm, 19 mm X 150 mm. Chromatography was performed at ambient temperature with a flow rate of 18mL/min with a linear gradient from water (0.1% FA): CH_3CN (0.1% FA)[95:5] to water (0.1% FA): CH_3CN (0.1% TFA) [5:95] in 12 minutes, monitored by 2998 Photodiode Array (PDA) Detector UV at 254 nm and/or 215 nM.

Integration of the thiocarbazate amino acid in SPPS:

Procedure A:

Wang or Rink amide amino acid-loaded resin (0.1 mmol) was swelled for 10 min in DMF (2.5 mLx2), followed by Fmoc cleavage using 20% solution of piperidine in DMF (2.5 mL x 2)/ two min per cleavage cycle. After successive washes with DMF (2.5 mL x 3)/ 30 seconds per wash and with DCM (2.5 mL x 5)/ 30 seconds per wash. The resin was then suspended in CH_3CN (0.5 mL) and treated with N-methylmorpholine base (NMM) (1.0 mmol, 10 equiv.) for 10 min. During this time, and separately, in a small vial, thiocarbazate (0.5 mmol, 5 equiv) and tetrabutylammonium chloride (TBACl) (0.5 mmol, 5 equiv) were dissolved completely in DCM (2.0 mL). Then, the reaction mixture was treated with trichloroisocyanuric acid (TCCA) (0.5 mmol, 5 equiv). The reaction mixture was stirred at rt for 5 min and then centrifuged. The clear supernatant was added directly to the resin, and the reaction mixture was shaken for 16 h, followed by 5 cycles of washing and then drying. A small amount of the resin was cleaved using a freshly made solution of TFA/H_2O/TIPS (95:2.5:2.5. v/v/v) and the resulting peptide was analyzed by HPLC.

Procedure B: (a modified version of procedure A to accommodate the reactive side chains, specifically for the tryptophan and histidine residues)

Wang or Rink amide amino acid-loaded resin (0.1 mmol) was swelled for 10 min in DMF (2.5 mLx2), followed by Fmoc cleavage using 20% solution of piperidine in DMF (2.5 mL x 2)/ two min per cleavage cycle. After successive washes with DMF (2.5 mL x 3)/ 30 seconds per wash and with DCM (2.5 mL x 5)/ 30 seconds per wash. The resin was then suspended in CH_3CN (0.5 mL) and treated with N-methyl morpholine base (NMM) (1.0 mmol, 10 equiv.) for 10 min. During this time, and separately, in a small vial, thiocarbazate (0.5 mmol. 5 equiv) and tetrabutylammonium chloride (TBACl) (0.75 mmol, 7.5 equiv) were dissolved completely in DCM (2.0 mL). Then, the reaction mixture was cooled down to -10 °C using an ice/acetone bath. After 10 min at -10 °C, the mixture was treated with trichloroisocyanuric acid (TCCA) (0.195 mmol, 0.39 equiv). The reaction mixture was stirred at -10 °C for 15 min and then treated with indole (0.5 mmol, 5.0 equiv). The mixture was allowed to reach rt and centrifuged. The clear supernatant was added directly to the resin, and the reaction mixture was shaken for 16 h, followed by 5 cycles of washing and then drying. A small amount of the resin was cleaved using a freshly made solution of TFA/H_2O/TIPS (95:2.5:2.5. v/v/v) and the resulting peptide was analyzed by HPLC.

Coupling to the aza-amino acids bound to the peptidyl chain: functionalization of the aza-amino acid moieties of the solid phase was performed using two different protocols as follow.

1. Coupling to the aza-amino acid using amino acid and HATU as coupling agent:

After cleavage of the Fmoc group from the peptidyl chain, the resin (0.1 mmol) was suspended in 0.4 M solution of NMM in DMF (2.5 mL, 1.0 mmol). Subsequently, a solution of the designated amino acid (0.5 mmol) mixed with HATU (0.5 mmol) in DMF (2.0 mL) was added to the resin. The resulting suspension was shaken for 2 h at rt, followed by drainage and multiple washes with DMF. The cycle was repeated once or twice depending on the conversion rate, which was established based on the HPLC analysis after a cleavage of small amount of the resin.

2. Coupling to the aza-amino acid using amino acid acyl chloride and solid $NaHCO_3$

Step 1: preparation of the acyl chloride.

To a solution of designated amino acid (4.0 mmol) in CH_2Cl_2 (5.0 ml), DMF (0.1 mL) and thionyl chloride (2.0 mL) were added. The reaction mixture was stirred at 0°C and warmed up gradually to rt. The reaction was stopped after 1 h by evaporating all volatiles under vacuum. The resulting crude preparation was triturated with hexane/ether (5.0 mL x 2). The resulting solid or semisolid was further dried under high vacuum for an additional hour. The dried amino acyl chloride was then stored at -20 °C for future use.

Step 2: Coupling to the aza-amino acid on the peptidyl chain.

The Fmoc protected aza-amino acid bound to the peptidyl chain (based on 0.1 mmol peptide) was treated with 20% piperidine solution in DMF (2.5 mL), the suspension was shaken for 2.0 min, and then the de-protection solution was drained. The Fmoc-cleavage protocol was repeated twice to ensure complete de-protection. After successive washes with DMF (1.0 mL x 3) and DCM (2.5 mL x 5), the dried resin was suspended with dioxane (2.5 mL) and treated with solid $NaHCO_3$ (250 mg, 3.0 mmol), the suspension was shaken for 10 min before it was treated with a solution of the designated amino acid acyl chloride (0.5 mmol) in dioxane (1.5 mL), which was prepared in the previous step. The reaction was shaken for 1 h, then the coupling solution was drained, and the resin was washed with water (2.5 mL x 2), DMF (2.5 mL x 3) and CH_2Cl_2 (2.5 mL x 5). A small amount of the resulting dried resin was subjected to TFA/H_2O/TIPS cleavage solution. The resulting cleaved peptide was triturated with ether at 0 ºC, the suspension was then centrifuged, and the dry solid was analyzed by HPLC to assess the conversion rate.

2.4 Synthesis and characterization of FSSE azapeptide analogues

Synthesis of the azaF^{1}SSazaE^4-NH_2 (51)

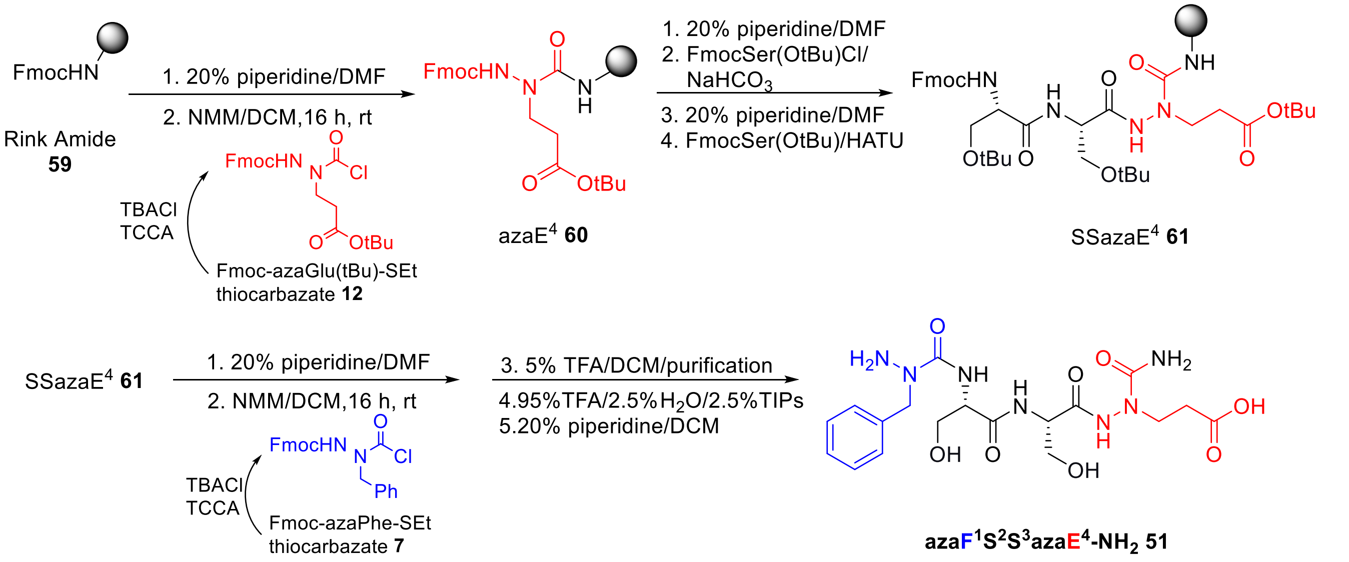

azaF^{1}SSE^4-NH_2 was prepared based on 0.5 mmol scale of Rink-amide. After the cleavage of the Fmoc using 20%Piperidine/DMF, the naked rink-amide was treated with 4.0 equivalents of the activated azaGlu thiocarbazates **12.** The semicarbazide azaE^4 (**60**) was functionalized with second amino acid in the sequence using FmocSer(Cl) (1.0 g, 2.5 mmol) and $NaHCO_3$ (1.26 g, 15 mmol) in Dioxane, the reaction was stopped after 90 min and sample analyzed by HPLC indicated the complete

conversion. The third amino acid was introduced using standard SPPS using FmocSer(OH) (957 mg, 2.5 mmol and HATU (950 mg, 2.5 mmol) in DMF and NMM as base. After 30 min, the resin was washed using the standard protocol and HPLC analysis confirmed the complete conversion. The resulting tripeptide **61** on resin was then treated sequentially with 20% piperidine and 4 equivalents of the activated azaPhe thiocarbazte **7** (2.0 mmol). The reaction was shaken for 16 h at rt. After standard washes, cleavage of small portion first using 5% TFA/DCM, then 95%TFA gave Fmoc protected final compound (67% crude purity at 254 nm). 25mg of which was treated with 20% piperidine/DCM (1.0 mL). The reaction mixture was stirred at rt for 30 min, then all volatiles were removed under vacuum, after trituration with ether, the resulting crude material was purified using prep C18 column and linear gradient from water (0.05%TFA): MeOH (0.05% TFA) [5:95] to water (0.05%TFA): MeOH (0.05% TFA) [95:5] in 12 min. After lyophilization, **51** was collected as fluffy powder (3.5 mg. 20.6 %). With purity of 97.9% based on HPLC, RT = 7.5 (flow rate of 0.8 mL/min with a linear gradient from Water (0.05% TFA): MeOH (0.05% TFA) [95:5] to water (0.05%TFA): MeOH (0.05% TFA) [5:95] in 20 min, monitored/detected UV at 215 nM by Photodiode Array (PDA) Detector. ^{1}HNMR for **17** (500MHz. MeOH-*d4*) δ 7.38-7.29 (m, 5H), 4.69 (ABq, $\Delta\delta$ = 0.09 Hz. *J* = 15.2 Hz, 2H), 4.36 (t, *J* = 4.85 Hz, 1H), 4.30 (t, *J* = 5.15 Hz, 1H), 3.97 (dd, *J* = 4.70, 10.90 Hz, 1H), 3.91 (dd, *J* = 5.4, 11.05 Hz, 1H). 3.81-3.71 (m, 4H), 2.59 (t, *J* = 7.3 Hz, 2H). ^{13}CNMR (125 MHz, MeOH-*d4*) δ 176.2, 175.3,173.0, 162.1, 161.7, 138.7, 130.8, 130.1, 130.0, 64.2, 63.3, 59.0, 57.4, 54.9, 46.0, 34.3. LRMS m/z calculated for $C_{18}H_{28}N_7O_8$ [M+H] 470.20 found 470.3, Ms2 found 427.3. HRMS m/z calculated for $C_{18}H_{28}N_7O_8$ [M+H] 470.1921 found 470.1996

Synthesis of the azaF^{1}SSE-NH$_2$ (52)

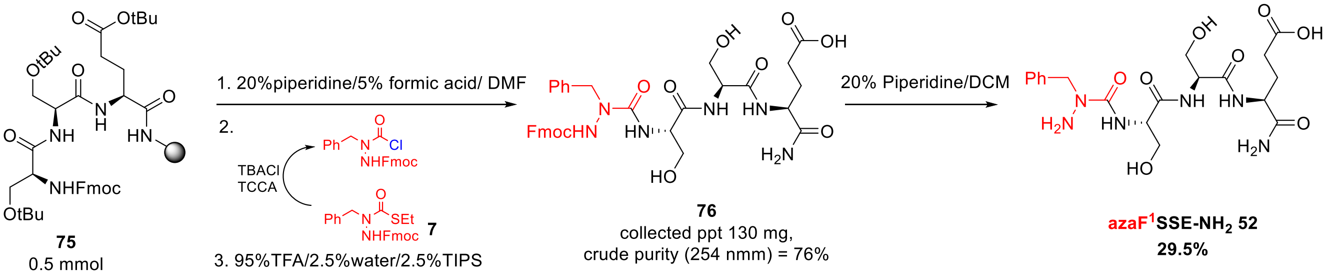

75 was built on 0.5 mmol using standard SPPS, treated with 4 equ. of the activated azaF thiocarbazate **7**, followed by cleavage of 10 mg of the peptide bound resin using fresh solution of TFA/H_2O/TIPS) (95:2.5:2.5, v/v/v, 0.25 mL) for 30 min at rt gave peptide **76** as white ppt in 76% crude yield based on HPLC analysis. **76** (50 mg, 0.072 mmol) was treated with 20% piperidine/DCM (1.0 mL). The reaction mixture was stirred at rt for 30 min, then all volatiles were removed under vacuum, after trituration with ether, the resulting crude material was purified using prep C18 column and linear gradient from water (0.05%TFA): MeOH (0.05% TFA) [95:5] to water (0.05%TFA): MeOH (0.05% TFA) [5:95] in 12 min. After lyophilization, **azaF^{1}SSE-NH$_2$** (**52**) was collected as fluffy powder (10 mg. 29.5%). With purity of 96.4% based on HPLC, RT = 8.4 (flow rate of 0.8 mL/min with a linear gradient from water (0.05% TFA): MeOH (0.05% TFA) [95:5] to water (0.05%TFA): MeOH (0.05% TFA) [5:95] in 20 min, monitored/detected UV at 215 nM by Photodiode Array (PDA) Detector.^{1}HNMR for **52** (600MHz. MeOH-*d4*) δ 7.36-7.29 (m, 5H), 4.69 (ABq, $\Delta\delta$= 0.08 Hz. *J* = 15.2 Hz, 2H), 4.38 (t, *J* = 4.50 Hz, 1H), 4.28 (t, *J* = 5.30 Hz, 1H), 3.97 (dd, *J* = 4.6, 11.1 Hz, 1H). 3.92 (dd, *J* = 5.4, 11.1 Hz, 1H), 3.82-3.79 (m, 2H), 2.42-2.33 (m, 2H), 2.20 (m, 1H), 1.94 (m, 1H). ^{13}CNMR (125 MHz, MeOH-*d4*) δ 177.5, 177.4, 175.4, 173.7, 162.3, 138.7, 130.8, 130.1, 129.7, 64.2, 63.2, 59.3, 58.5, 55.2, 54.8, 32.4, 28.7. LRMS m/z calculated for $C_{19}H_{29}N_6O_8$ [M+H] 469.20 found 469.30, Ms2 found 451.2 and 433.3. HRMS m/z calculated for $C_{19}H_{29}N_6O_8$ [M+H] 469.1969 found 469.2043

Synthesis of the FSSazaE^4-NH$_2$ (53)

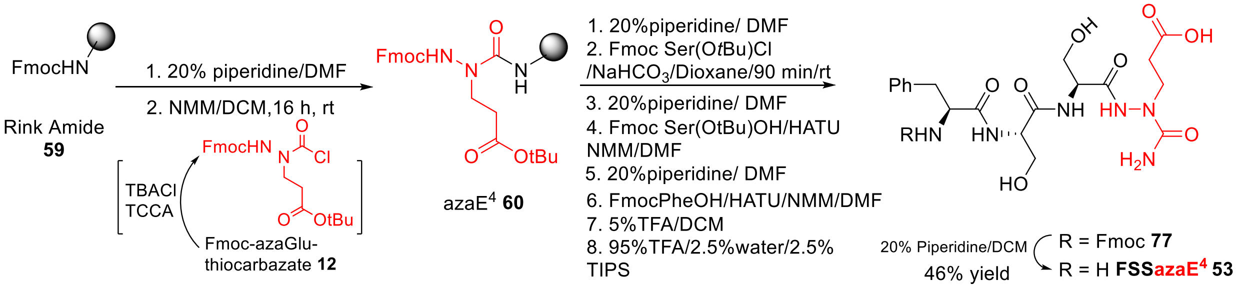

Tetrapeptide **FSSazaE⁴-NH₂** (**53**) was built on 0.6 mmol and 4eq of the activated azaGlu thiocarbazates **12** using the standard procedure mentioned above. Upon completion, the bound resin peptide was treated initially with 5% TFA/DCM solution (10 mL x 3). The combined fractions were evaporated to dryness at rt. The crude mixture was then treated with 95%TFA/2.5% H_2O/ 2.5% TIPS solution to give the tetrapeptide **77**, which was triturated with ether, dried, and analyzed by HPLC ($MeOH/H_2O$) and found in 93% crude purity (104 mg crude ppt, 25% yield). Fmoc derivative **77** (40 mg, 0.057 mmol) was treated with 20% piperidine/DCM (1.0 mL). The reaction mixture was stirred at rt for 30 min, then all volatiles were removed under vacuum, after trituration with ether, the resulting crude material was purified using prep C18 column and linear gradient from water (0.05%TFA): MeOH (0.05% TFA) [95:5] to water (0.05%TFA): MeOH (0.05% TFA) [5:95] in 12 min. After lyophilization, **FSSazaE⁴-NH₂** (**53**) was collected as fluffy powder (12.5 mg. 46 %). With purity of 97.8% based on HPLC, RT = 3.8 (flow rate of 0.8 mL/min with a linear gradient from water (0.05% TFA): MeOH (0.05% TFA) [95:5] to water (0.05%TFA): MeOH (0.05% TFA) [5:95] in 20 min, monitored/detected UV at 215 nM by PDA. ^{1}HNMR for **53** (600MHz. MeOH-*d4*) δ 7.37-7.30 (m, 5H), 4.55 (t, J = 6.06 Hz, 1H), 4.34 (t, J = 5.50 Hz, 1H), 4.18 (dd, J = 5.64, 8.64 Hz, 1H), 3.93 (dd, J = 5.1, 10.8 Hz, 1H), 3.85 (dd, J = 5.88, 10.9 Hz, 1H). 3.80-3.76 (m, 4H), 3.28 (m, 1H), 3.04 (dd, J = 8.64, 14.3 Hz, 1H) 2.59 (t, J = 7.32 Hz, 2H). ^{13}CNMR (125 MHz, MeOH-*d4*) δ 176.3, 173.2, 173.0, 170.8, 161.8, 136.5, 131.4, 131.1, 129.8, 63.9, 63.3, 57.3, 57.2, 56.5, 46.0, 39.5, 34.3. LRMS m/z calculated for $C_{19}H_{29}N_6O_8$ [M+H] 469.22 found 469.3, Ms2 found 426.2. HRMS m/z calculated for $C_{19}H_{29}N_6O_8$ [M+H] 469.1969 found 469.2043

Synthesis of the azaF¹SSE-OH (54)

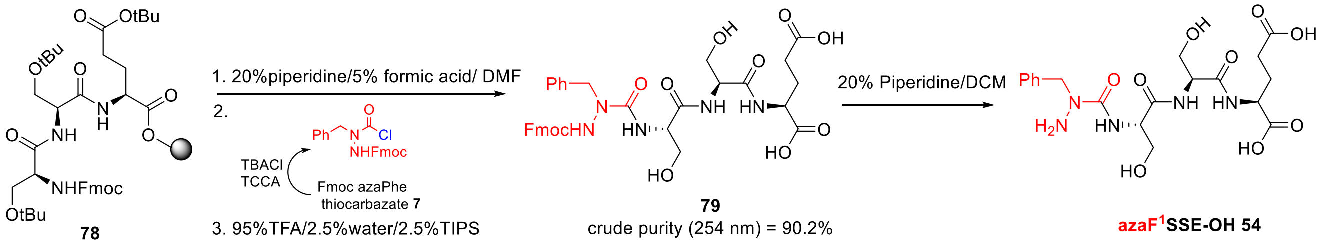

The titled compound was built on 0.5 mmol tripeptide **78** and 4 eq. azaF thiocarbazate **7** using the standard procedure above. Cleavage of 10 mg of the peptide bound resin using fresh solution of TFA/H_2O/TIPS) (95:2.5:2.5, v/v/v, 0.25 mL) for 30 min at rt gave peptide crude **79** with 90.2% crude purity analyzed by HPLC. **79** (50 mg, 0.072 mmol) was treated with 20% piperidine/DCM (1.0 mL). The reaction mixture was stirred at rt for 30 min, then all volatiles were removed under vacuum. After trituration with ether, the crude material was purified using prep C18 column and linear gradient from water (0.05%TFA): MeOH (0.05% TFA) [95:5] to water (0.05%TFA): MeOH (0.05% TFA) [5:95]in 12 min. After lyophilization, **azaF¹SSE-OH (54)** was collected as fluffy powder (21 mg. 63%). With purity of 98% based on HPLC, RT = 8.5 (flow rate of 0.8 mL/min with a linear gradient from Water (0.05% TFA): MeOH (0.05% TFA) [95:5] to water (0.05%TFA): MeOH (0.05% TFA) [5:95] in 20 min, monitored/detected UV at 215 nM by PDA. ^{1}HNMR for **54** (600MHz. MeOH-*d4*) δ 7.35-7.28 (m, 5H), 4.69 (m, 2H), 4.50 (t, J = 4.74 Hz, 1H), 4.46 (dd, J 5.0. 9.12 Hz, 1H), 4.34 (t, *0* 5.4 Hz, 1H), 3.93-3.91 (m, 2H), 3.84-3.77 (m, 2H), 2.40 (t, J = 7.6 Hz, 2H), 2.20 (m, 1H), 1.97 (m, 1H). ^{13}CNMR (125 MHz, MeOH-*d4*) δ 177.3, 175.5, 174.5, 173.3, 162.1, 138.6, 130.8, 130.1, 129.7, 64.4, 63.7, 58.8, 57.7, 54.9, 54.1, 32.1, 28.7; LRMS m/z calculated for $C_{19}H_{28}N_5O_9$ [M+H] 470.19 found 470.3. Ms2 found 452.2 and 434.3. HRMS m/z calculated for $C_{19}H_{28}N_5O_9$ [M+H] 470.1809 found 470.1884.

Synthesis of the azaF¹SSQ-OH (55)

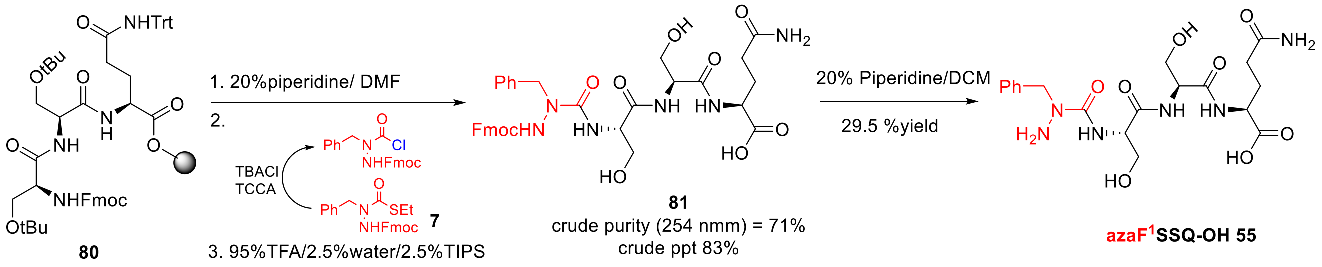

azaF[1]SSQ-OH **(55)** was built on 0.5 mmol resin and 4 equ. azaF thiocarbazate **7** using the standard procedure mentioned above. Cleavage of 10 mg of the peptide bound resin using fresh solution of TFA/H_2O/TIPS) (95:2.5:2.5, v/v/v, 0.25 mL) for 30 min at rt gave peptide **81** as white ppt in 71% crude yield analyzed by HPLC. **81** (50 mg, 0.073 mmol) was treated with 20% piperidine/DCM (1.0 mL). The reaction mixture was stirred at rt for 30 min, then all volatiles were removed under vacuum, after trituration with ether, the resulting crude material was purified using prep C18 column and linear gradient from water (0.05%TFA): MeOH (0.05% TFA) [95:5] to water (0.05%TFA): MeOH (0.05% TFA) [5:95] in 12 min. After lyophilization, **azaF[1]SSQ-OH** (**55**) was collected as fluffy powder (10 mg. 29.5%) With purity of 95.8% based on HPLC, RT = 7.5 (flow rate of 0.8 mL/min with a linear gradient from water (0.05% TFA): MeOH (0.05% TFA)[95:5] to water (0.05%TFA): MeOH (0.05% TFA) [5:95] in 20 min, monitored/detected UV at 215 nM by PDA.^{1}HNMR for **55** (600MHz. MeOH-*d4*) δ 7.36-7.29 (m, 5H), 4.69 (ABq, J = 15.24 Hz, 2H), 4.48 (t, J = 4.80 Hz, 1H), 4.42 (dd, J = 4.6, 9.5 Hz, 1H), 4.35 (t, J = 5.3 Hz, 1H), 3.93 (dd, J = 5.2, 11.0 Hz, 1H). 3.83 (dd, J = 4.6, 11.3 Hz, 1H), 3.79 (dd, J = 5.6, 11.0 Hz, 1H), 2.30 (m, 2H), 2.19 (m, 1H), 1.96 (m, 1H). ^{13}CNMR (125 MHz, MeOH-*d4*) δ 178.7, 175.4, 174.6, 173.3, 162.0, 138.6, 130.8, 130.1, 129.7, 64.4, 63.6, 58.7, 57.8, 54.9, 33.5, 29.4; LRMS m/z calculated for $C_{19}H_{29}N_7O_8$ [M+H] 469.22 found 469.3, Ms2 found 451.1 and 433.3. HRMS m/z calculated for $C_{19}H_{29}N_7O_8$ [M+H] 469.1969 found 469.2043.

Synthesis of the azaF[1]SSQ-NH_2 (56)

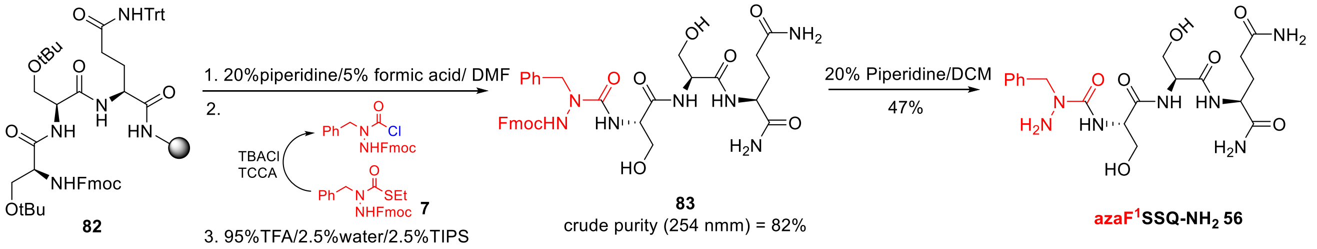

azaF[1]SSQ-NH_2 **(56)** was built on 0.6 mmol and 4 equ. azaF thiocarbazate **7** using the standard procedure mentioned above. Cleavage of 10 mg of the peptide bound resin using fresh solution of TFA/H_2O/TIPS) (95:2.5:2.5, v/v/v, 0.25 mL) for 30 min at rt gave peptide **83** as white ppt in 82% crude yield analyzed by HPLC. **83** (50 mg, 0.073 mmol) was treated with 20% piperidine/DCM (1.0 mL). The reaction mixture was stirred at rt for 30 min, then all volatiles were removed under vacuum, after trituration with ether, the resulting crude material was purified using prep C18 column and linear gradient from water (0.05%TFA): MeOH (0.05% TFA) [95:5] to water (0.05%TFA): MeOH (0.05% TFA) [5:95] in 12 min. After lyophilization, **azaF[1]SSQ-NH_2** (**56**) was collected as fluffy powder (16 mg. 47%). With purity of 96.4% based on HPLC, RT = 7.05 (flow rate of 0.8 mL/min with a linear gradient from water (0.05% TFA): MeOH (0.05% TFA) [95:5] to water (0.05%TFA): MeOH (0.05% TFA) [5:95] in 20 min, monitored/detected UV at 215 nM by PDA. ^{1}HNMR for **56** (600MHz. MeOH-*d4*) δ 7.28-7.18 (m, 5H), 4.60 (ABq, $\Delta\delta$= 0.06 Hz. J = 15.3 Hz, 2H), 4.28 (t, J = 4.68 Hz, 1H), 4.24 (dd, J = 4.3, 10.1 Hz, 1H), 4.21 (t, J = 5.28 Hz, 1H), 3.88 (dd, J = 4.6, 11.1 Hz, 1H). 3.83 (dd, J = 5.3, 11.0 Hz, 1H), 3.7127 (ddd, J = 4.7, 6.5, 7.02 Hz, 2H), 2.27 (m, 2H), 2.19 (m, 1H), 1.93 (m, 1H). ^{13}CNMR (125 MHz, MeOH-*d4*) δ 178.7, 177.4, 175.3, 173.6, 162.1, 138.6, 130.8, 130.0, 129.7, 64.2, 63.2, 59.1, 58.4, 55.1, 54.8, 33.7, 29.4. LRMS m/z calculated for $C_{19}H_{30}N_7O_7$ [M+H] 468.22 found 468.33, Ms2 found 450.25 and 432.25. HRMS m/z calculated for $C_{19}H_{30}N_7O_7$ [M+H] 468.2128 found 468.2201

Synthesis of the tetrapeptide azaF[1]SSazaQ[4]-NH_2 (57)

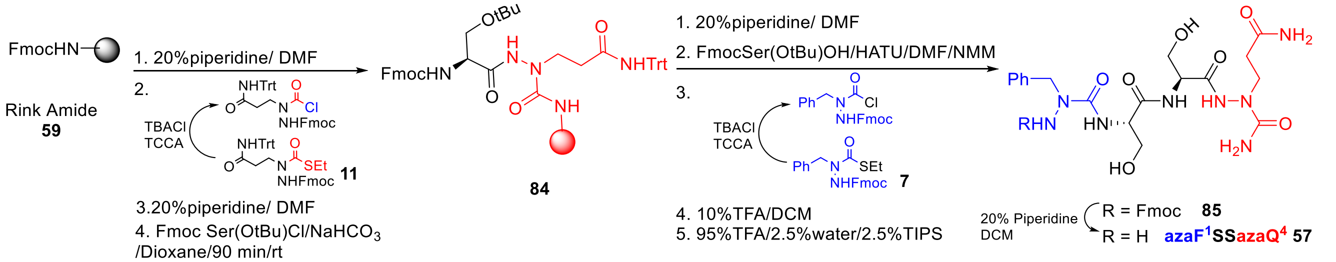

azaF[1]SSazaQ[4]-NH_2 (57) was built on 0.4 mmol Rink amide, 4 equ. azaGln thiocarbazate **11** and 3.0 equiv azaPhe thiocarbazate (**7**) using the standard procedure above. **51** (50 mg, 0.073 mmol, 41% crude purity) was treated with 20%

piperidine/DCM (1.0 mL). The reaction mixture was stirred at rt for 30 min, then all volatiles were removed under vacuum, after trituration with ether, the resulting crude material was purified using prep C18 column and linear gradient from water (0.05%TFA): MeOH (0.05% TFA) [95:5] to water (0.05%TFA): MeOH (0.05% TFA) [5:95] in 12 min. After lyophilization, **azaF¹SSazaQ⁴-NH₂ (57)** was collected as fluffy powder (18 mg. 53 %) With purity of 96.8% based on HPLC, RT = 7.05 (flow rate of 0.8 mL/min with a linear gradient from water (0.05% TFA): MeOH (0.05% TFA)[95:5] to water (0.05%TFA): MeOH (0.05% TFA) [5:95] in 20 min, monitored/detected UV at 215 nM by PDA.^{1}HNMR for **57** (600MHz. MeOH-*d4*) δ 7.35-7.29 (m, 5H), 4.69 (ABq, $\Delta\ \delta$= 0.10 Hz. J = 15.24 Hz, 2H), 4.36 (t, J = 4.80 Hz, 1H), 4.30 (t, J = 5.30 Hz, 1H), 3.98 (dd, J = 4.56, 10.9 Hz, 1H), 3.91 (dd, J = 5.34, 11.04 Hz, 1H). 3.83-3.75 (m, 4H), 2.44 (t, J = 7.02 Hz, 2H). ^{13}CNMR (125 MHz, MeOH-*d4*) δ 177.6, 175.3, 173.2, 162.4, 161.8, 138.6, 130.8, 130.7, 129.7, 64.2, 63.3, 59.1, 57.5, 54.9, 46.6, 35.6. LRMS m/z calculated for $C_{18}H_{29}N_8O_7$ [M+H] 469.22 found 469.3, Ms2 found 426.2. HRMS m/z calculated for $C_{18}H_{29}N_8O_7$ [M+H] 469.2081 found 469.2148

Synthesis of the FSSazaQ⁴-NH₂ (58)

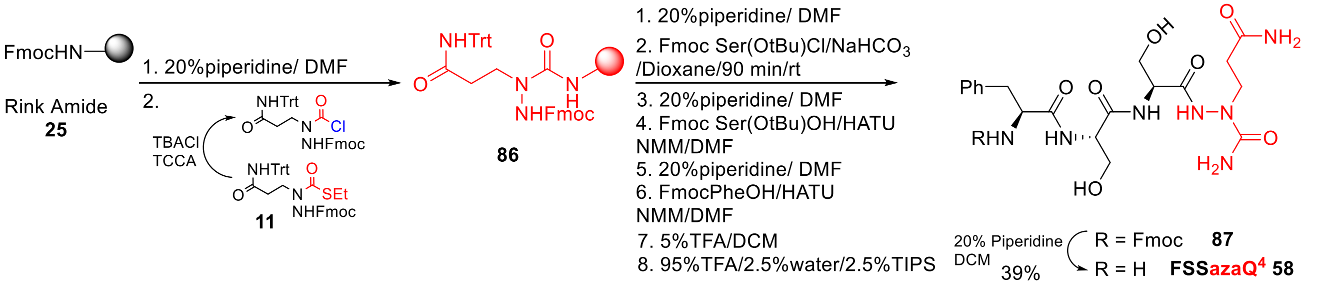

Tetrapeptide **FSSazaQ⁴-NH₂ (58)** was built on 0.3 mmol rink amide and 4 equ. azaGln thiocarbazate **11** using the standard procedure mentioned above. Upon completion, the bound resin peptide was treated initially with 5% TFA/DCM solution (10 mL x 3). The combined fractions were evaporated to dryness at rt. The crude mixture was then treated with 95%TFA/2.5% H_2O/ 2.5% TIPS solution to give **87** (120 mg crude ppt), with was subjected to multiple purification on silica using gradient of EtOAc/MeOH, as a result only 23 mg was collected (11.1%). **87** (23 mg, 0.033 mmol, 50.2% crude purity) was treated with 20% piperidine/DCM (1.0 mL). The reaction mixture was stirred at rt for 30 min, then all volatiles were removed under vacuum, after trituration with ether, the resulting crude material was purified using prep C18 column and linear gradient from water (0.05%TFA): MeOH (0.05% TFA) [95:5] to water (0.05%TFA): MeOH (0.05% TFA) [5:95] in 12 min. After lyophilization, **FSSazaQ⁴-NH₂ (58)** was collected as fluffy powder (6.0 mg. 39 %). With purity of 97.9% based on HPLC, RT = 3.63 (flow rate of 0.8 mL/min with a linear gradient from water (0.05% TFA): MeOH (0.05% TFA) [95:5] to water (0.05%TFA): MeOH (0.05% TFA) [5:95] in 20 min, monitored/detected UV at 215 nM by PDA. ^{1}HNMR for **58** (600MHz. MeOH-*d4*) δ 7.37-7.31 (m, 5H), 4.54 (t, J = 5.95 Hz, 1H), 4.35 (t, J = 5.40 Hz, 1H), 4.19 (dd, J = 5.63, 8.64 Hz, 1H), 3.94 (dd, J = 4.98, 10.8 Hz, 1H), 3.85 (dd, J = 5.83, 10.86 Hz, 1H). 3.82-3.76 (m, 4H), 3.30 (m, 1H), 3.05 (dd, J = 8.64, 14.3 Hz, 1H) 2.47 (t, J = 6.95 Hz, 2H). ^{13}CNMR (125 MHz, MeOH-*d4*) δ 177.7, 173.2, 173.1, 170.9, 161.8, 136.5, 131.4, 131.1, 129.8, 63.9, 63.3, 57.3, 56.5, 46.7, 39.5, 35.9. LRMS m/z calculated for $C_{19}H_{30}N_7O_7$ [M+H] 468.22 found 468.3. HRMS m/z calculated for $C_{19}H_{30}N_7O_7$ [M+H] 468.2128 found 468.2201

2.5 Synthesis and characterization of bradykinin azapeptide analogues

Synthesis of the azaR¹-BK (62) (azaR¹P²P³G⁴F⁵S⁶P⁷F⁸R⁹-OH)

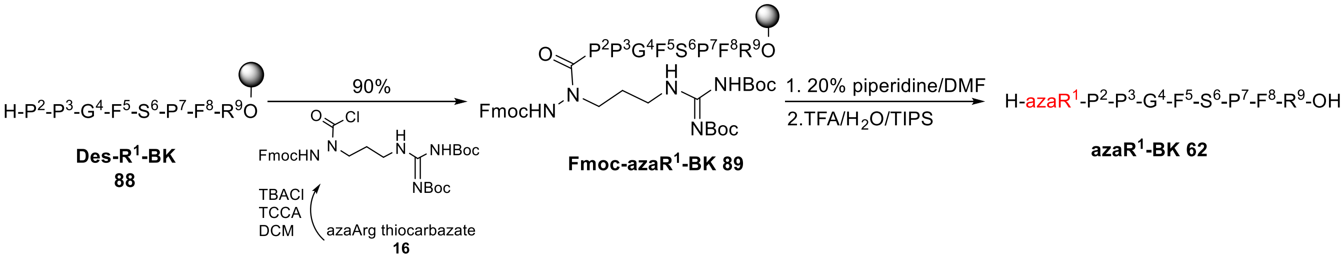

azaR¹-BK (**62**) was prepared based on 0.1 mmol scale following the general procedures above and collected as white fluffy powder in 85.7% crude purity based on HPLC, RT = 11.76 min (flow rate of 1.0 mL/min with linear gradient from Water (0.05% TFA): MeOH (0.05% TFA) [95:5] to water (0.05%TFA): MeOH (0.05% TFA) [5:95] in 20 min, monitored/detected

UV at 215 nM by Photodiode Array (PDA) Detector. LRMS m/z calculated for $C_{49}H_{72}N_{16}O_{11}$ [M+H] 1060.56 found 1061.75. However, azaR[1]-BK (**62**) was found to be unstable after deprotection and purification. This result is not unusual and matches with results reported by Fuller et al. [6]

Synthesis of the azaP[2]-BK (63) (R[1]azaP[2]P[3]G[4]F[5]S[6]P[7]F[8]R[9]-OH)

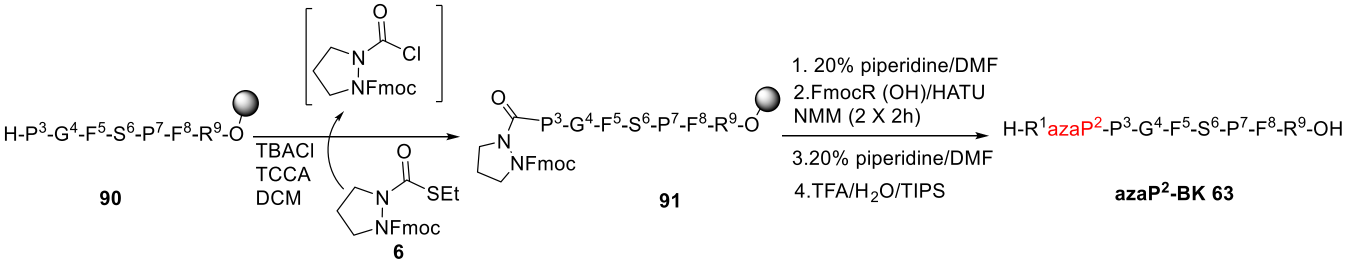

azaP[2]-BK (**63**) was prepared based on 0.1 mmol scale following the general procedures above and collected as white fluffy powder in 38% crude purity. The crude material was purified using prep HPLC system, after lyophilization, **63** was collected as fluffy powder (35.4% isolated yield), with purity of 97% based on HPLC, RT = 5.0 min (flow rate of 1.0 mL/min with linear gradient from water (0.05% TFA): CH_3CN (0.05% TFA) [95:5] to water (0.05% TFA): CH_3CN (0.05% TFA) [5:95] in 15 min, monitored/detected UV at 215 nM by Photodiode Array (PDA) Detector. LRMS m/z calculated for $C_{49}H_{72}N_{16}O_{11}$ [M+H] 1061.20 found 1061.75; HRMS m/z calculated for $C_{49}H_{72}N_{16}O_{11}$ [M+H] 1061.5566 found 1061.5691

Synthesis of the azaP[3]-BK (64) (R[1]P[2]azaP[3]G[4]F[5]S[6]P[7]F[8]R[9]-OH)

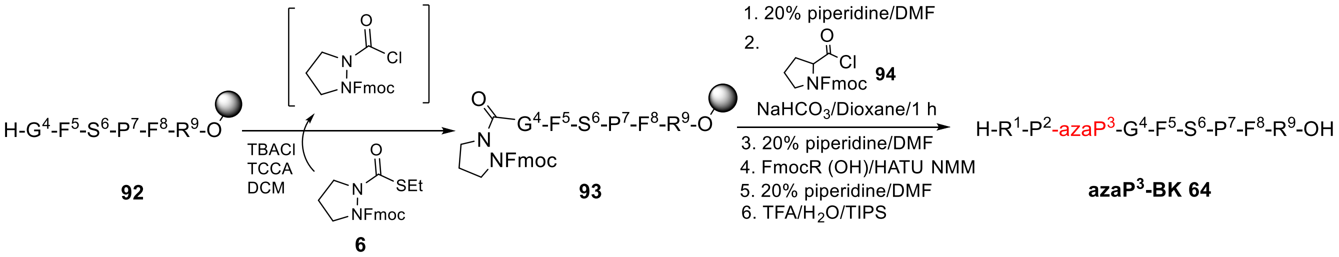

azaP[3]-BK (**64**) was prepared based on 0.1 mmol scale following the general procedures above and collected as white fluffy powder in 53.5% crude purity. The crude material was purified using prep HPLC system, after lyophilization, **64** was collected as fluffy powder (41% isolated yield). With purity of 96.9% based on HPLC, RT = 5.06 min (flow rate of 1.0 mL/min with linear gradient from water (0.05% TFA): CH_3CN (0.05% TFA) [95:5] to water (0.05% TFA): CH_3CN (0.05% TFA) [5:95] in 15 min, monitored/detected UV at 215 nM by Photodiode Array (PDA) Detector. LRMS m/z calculated for $C_{49}H_{72}N_{16}O_{11}$ [M+H] 1061.20 found 1061.75. HRMS m/z calculated for $C_{49}H_{72}N_{16}O_{11}$ [M+H] 1061.5566 found 1061.5811.

Synthesis of the azaG[4]-BK (65) (R[1]P[2]P[3]azaG[4]F[5]S[6]P[7]F[8]R[9]-OH)

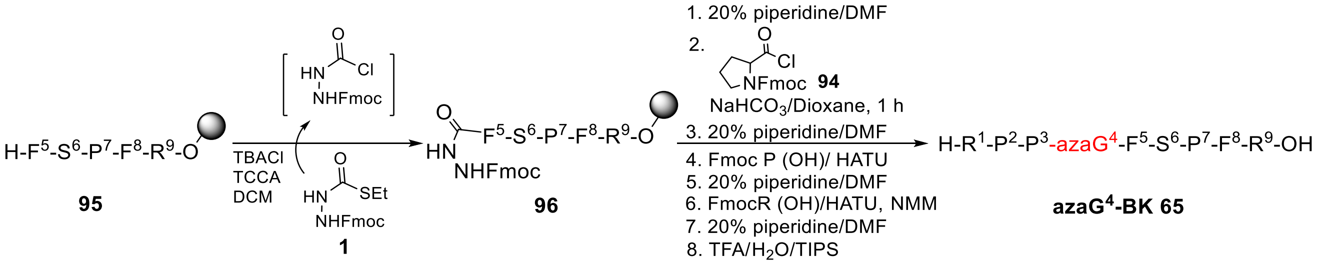

azaG[4]-BK (**65**) was prepared based on 0.1 mmol scale following the general procedures above and collected as white fluffy powder in 34% crude purity. The crude material was purified using prep HPLC system, after lyophilization, **65** was collected as fluffy powder (17.7% isolated yield). With purity of 97.6% based on HPLC, RT = 4.86 min (flow rate of 1.0 mL/min with linear gradient from water (0.05% TFA): CH_3CN (0.05% TFA) [95:5] to water (0.05% TFA): CH_3CN (0.05% TFA) [5:95] in 15 min, monitored/detected UV at 215 nM by Photodiode Array (PDA) Detector. LRMS m/z calculated for $C_{49}H_{72}N_{16}O_{11}$ [M+H] 1061.20 found 1061.67. HRMS m/z calculated for $C_{49}H_{72}N_{16}O_{11}$ [M+H] 1061.5566 found 1061.5686, [M+2H] 531.2861.

Synthesis of the azaF^5-BK (66) (H-R^{1}P^{2}P^{3}G^{4}azaF^{5}S^{6}P^{7}F^{8}R^9-OH)

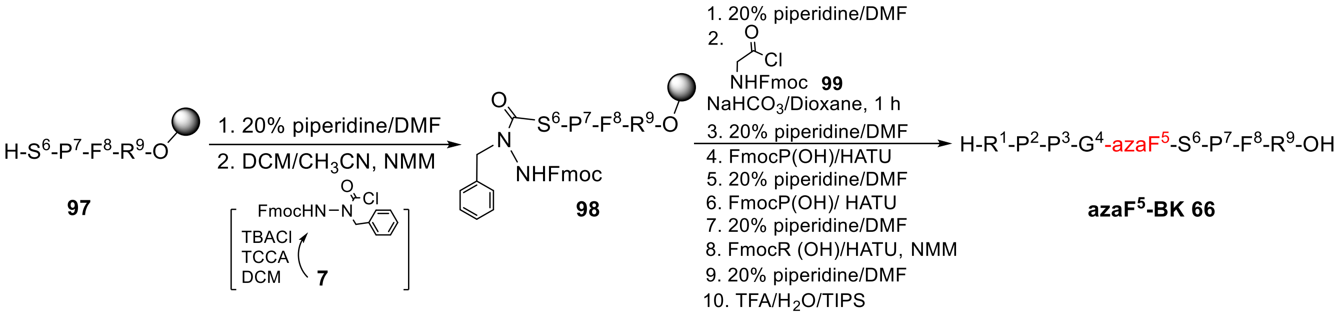

azaF^5-BK (**66**) was prepared based on 0.1 mmol scale following the general procedures above and collected as white fluffy powder in 66% crude purity. The crude material was purified using prep HPLC system, after lyophilization, **66** was collected as fluffy powder (16.5% isolated yield). With purity of 95.3% based on HPLC, RT = 5.04 min (flow rate of 1.0 mL/min with linear gradient from water (0.05% TFA): CH_3CN (0.05% TFA) [95:5] to water (0.05% TFA): CH_3CN (0.05% TFA) [5:95] in 15 min, monitored/detected UV at 215 nM by Photodiode Array (PDA) Detector. LRMS m/z calculated for $C_{49}H_{72}N_{16}O_{11}$ [M+H] 1061.20 found 1061.75. HRMS m/z calculated for $C_{49}H_{72}N_{16}O_{11}$ [M+H] 1061.5566 found 1061.5651, [M+2H] 531.2863.

Synthesis of the azaP^7-BK (67) (H-R^{1}P^{2}P^{3}G^{4}F^{5}S^{6}azaP^{7}F^{8}R^9-OH)

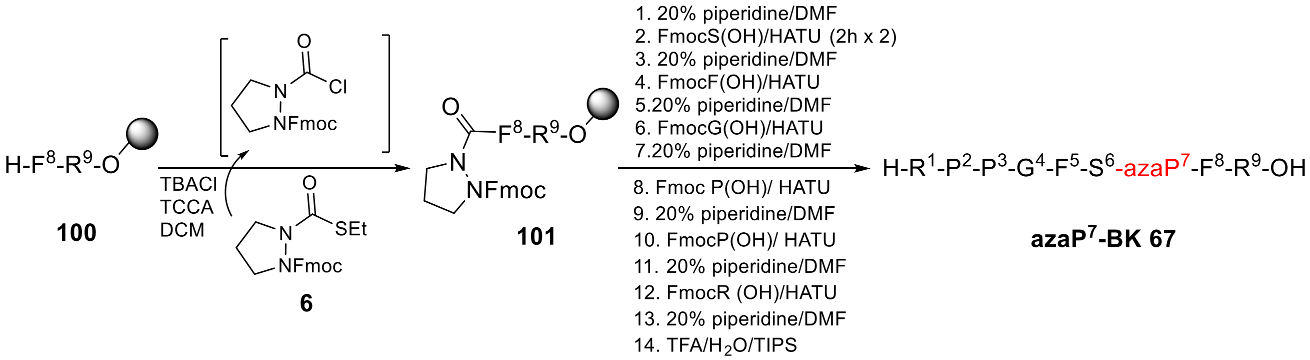

azaP^7-BK (**67**) was prepared based on 0.1 mmol scale following the general procedures above and collected as white fluffy powder in 49.6% crude purity. The crude material was purified using prep HPLC system, after lyophilization, **67** was collected as fluffy powder (23.6% isolated yield).With purity of 95% based on HPLC, RT = 4.99 min (flow rate of 1.0 mL/min with linear gradient from water (0.05% TFA): CH_3CN (0.05% TFA) [95:5] to water (0.05% TFA): CH_3CN (0.05% TFA) [5:95] in 15 min, monitored/detected UV at 215 nM by Photodiode Array (PDA) Detector. LRMS m/z calculated for $C_{49}H_{72}N_{16}O_{11}$ [M+H] 1061.20 found 1061.75. HRMS m/z calculated for $C_{49}H_{72}N_{16}O_{11}$ [M+H] 1061.5566 found 1061.5732, [M+2H] 531.2860

Synthesis of the azaF^8-BK (68) (H-R^{1}P^{2}P^{3}G^{4}F^{5}S^{6}P^{7}azaF^{8}R^9-OH)

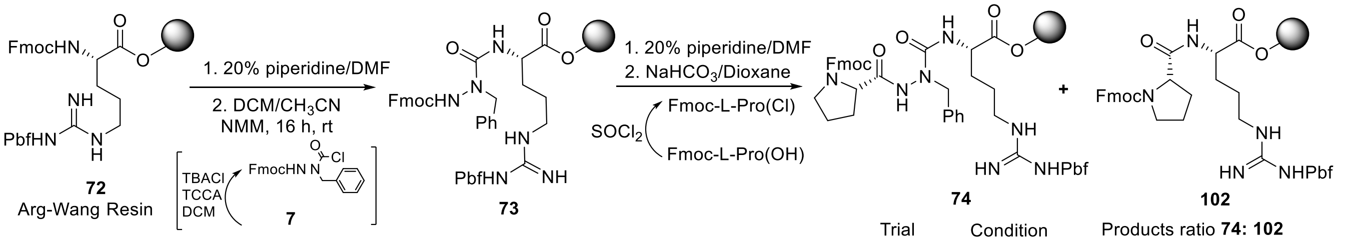

Trial	Condition	Products ratio **74: 102**
1	Shake, 20 min	3:1
2	Shake, 4h	3:1
3	Shake, 16h	20:1

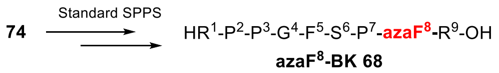

azaF^8-BK (**68**) was prepared based on 0.1 mmol scale following the general procedures above and collected as white fluffy

powder in 68.4% crude purity. The crude material was purified using prep HPLC system, after lyophilization, **68** was collected as fluffy powder (14% isolated yield). With purity of 97.6% based on HPLC, RT = 5.0 min (flow rate of 1.0 mL/min with linear gradient from water (0.05% TFA): CH_3CN (0.05% TFA) [95:5] to water (0.05% TFA): CH_3CN (0.05% TFA) [5:95] in 15 min, monitored/detected UV at 215 nM by Photodiode Array (PDA) Detector. LRMS m/z calculated for $C_{49}H_{72}N_{16}O_{11}$ [M+H] 1061.20 found 1061.75. HRMS m/z calculated for $C_{49}H_{72}N_{16}O_{11}$ [M+H] 1061.5566 found 1061.5885, [M+2H] 531.2864

The azaamino acid residue integration was optimized for coupling with **73**. The cleavage of a small amount of the resin using the TFA/H_2O/TIPS solution, followed by HPLC analysis, indicated that a 66% conversion of **73** to **74** could be achieved after 20 min. However, extending the mixing time to 4.0 h didn't change the conversion ratio much. Satisfyingly, **74** was produced as the only major product with crude purity >90% after 16 h. the ratio of **74** to **102** was established after the proline's integration in the next step to be 20:1, respectively. These findings come with great agreement with that reported by Gibson[7], Lubell[5], and Freeman[8].

Synthesis of the azaR⁹-BK (69) (H-R¹P²P³G⁴F⁵S⁶P⁷F⁸azaR⁹-NH₂)

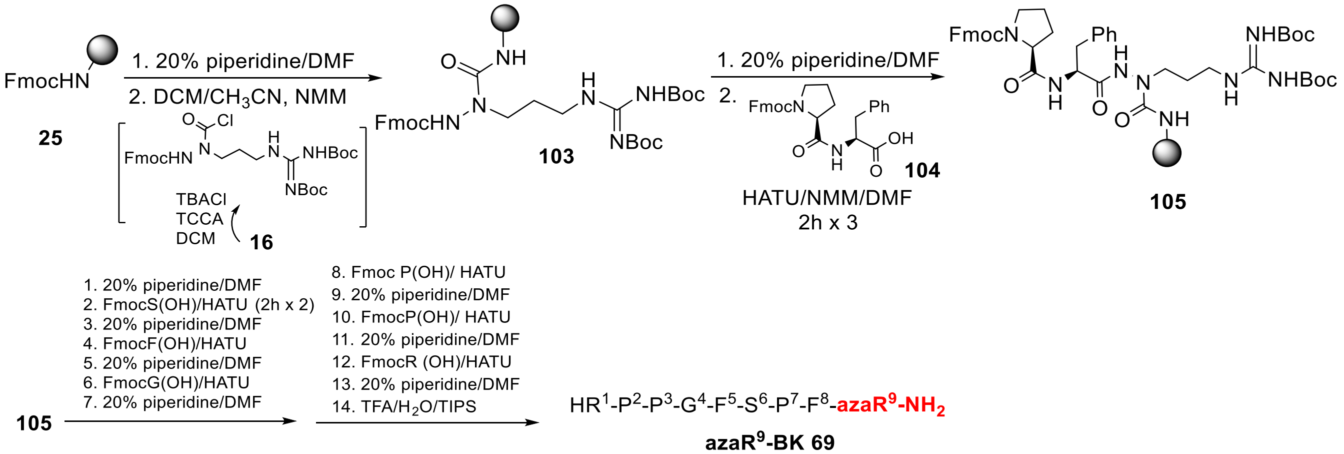

azaR⁹-BK 69

azaR⁹-BK (**69**) was prepared based on 0.1 mmol scale following the general procedures above and collected as white fluffy powder in 19.2% crude purity. The crude material was purified using prep HPLC system, after lyophilization, **69** was collected as fluffy powder (5.4% isolated yield). With purity of 95.9% based on HPLC, RT = 4.74 min (flow rate of 1.0 mL/min with linear gradient from water (0.05% TFA): CH_3CN (0.05% TFA) [95:5] to water (0.05% TFA): CH_3CN (0.05% TFA) [5:95] in 15 min, monitored/detected UV at 215 nM by Photodiode Array (PDA) Detector. LRMS m/z calculated for $C_{49}H_{73}N_{17}O_{10}$ [M+H] 1060.20 found 1060.67. HRMS m/z calculated for $C_{49}H_{73}N_{17}O_{10}$ [M+H] 1061.5726 found 1061.5905, [M+2H] 530.7940

Synthesis of the [azaP², azaF⁸]-BK (70) (H-R¹azaP²P³G⁴F⁵S⁶P⁷azaF⁸R⁹-OH)

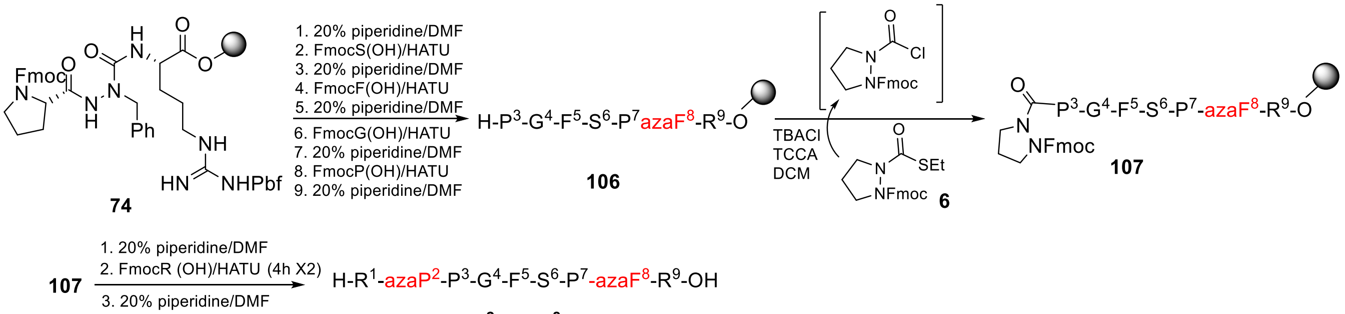

[azaP², azaF⁸]-BK 70

[azaP², azaF⁸]-BK (**70**) was prepared based on 0.1 mmol scale following the general procedures above and collected as white fluffy powder in 42.8% crude purity. The crude material was purified using prep HPLC system, after lyophilization, **70** was collected as fluffy powder (21% isolated yield).With purity of 98.5% based on HPLC, RT = 4.94 min (flow rate of 1.0 mL/min with linear gradient from water (0.05% TFA): CH_3CN (0.05% TFA) [95:5] to water (0.05% TFA): CH_3CN (0.05% TFA) [5:95]

in 15 min, monitored/detected UV at 215 nm by Photodiode Array (PDA) Detector. LRMS m/z calculated for $C_{48}H_{71}N_{17}O_{11}$ [M+H] 1062.19 found 1062.67. HRMS m/z calculated for $C_{48}H_{71}N_{17}O_{11}$ [M+2H] 531.2759 found 531.7872.

Synthesis of the [azaF^5, azaF^8]-BK (71) (H-R^{1}P^{2}P^{3}G^{4}azaF^{5}S^{6}P^{7}azaF^{8}R^9-OH)

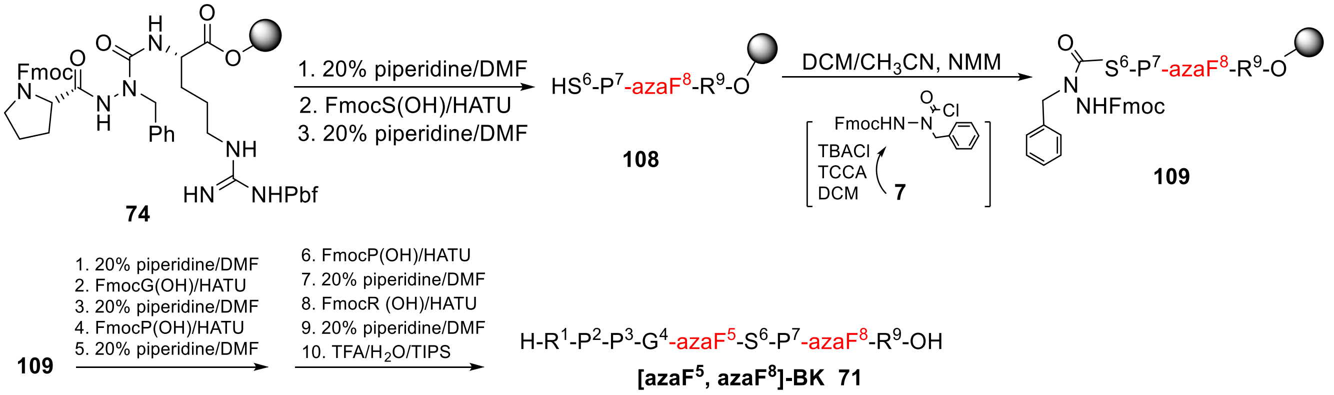

[azaF^5, azaF^8]-BK (**71**) was prepared based on 0.1 mmol scale following the general procedures above and collected as white fluffy powder in 66% crude purity. The crude material was purified using prep HPLC system, after lyophilization, **71** was collected as fluffy powder (17.7% isolated yield). With purity of 95.3% based on HPLC, RT = 4.9 min (flow rate of 1.0 mL/min with linear gradient from water (0.05% TFA): CH_3CN (0.05% TFA) [95:5] to water (0.05% TFA): CH_3CN (0.05% TFA) [5:95] in 15 min, monitored/detected UV at 215 nM by Photodiode Array (PDA) Detector. LRMS m/z calculated for $C_{48}H_{71}N_{17}O_{11}$ [M+H] 1062.19 found 1062.67. HRMS m/z calculated for $C_{48}H_{71}N_{17}O_{11}$ [M+2H] 531.2759 found 531.7854.

3. Ex vivo stabilities and half-life experiments of FSSE and bradykinin-based azapeptides

3.1 Preparation of the standard and quality control solutions of FSSE and BK analogues

A stock standard solution of FSSE or BK analogues was prepared in DMSO (5 mg/mL) and diluted with ion-free water to prepare working solutions at concentrations of 0.01, 0.1, 1, and 10 µg/ml, respectively. Standard solutions (2.5, 5, 10, 25, 50, 100, 250, 500, 1000 and 2500 ng/ml) of FSSE or BK analogues were prepared from the working solutions by dilution with 2% ACN in 0.1% formic acid water. Quality control solutions used in the validation were prepared in the same manner as the standard solutions. All solutions were stored at 4°C before use. FSSE and BK analogues were purified by flash chromatography or C18 column; structures were confirmed by MS and NMR.

3.2 Sample preparation and LC-MS conditions

Studies were carried out in male C57BL/6J mouse whole blood collected in sodium heparin. Blood was adjusted to pH 7.4 prior to initiating the experiments. DMSO stocks (5 mg/ml) were first prepared for the test article. Aliquots of the DMSO solutions were dosed into 2 mL whole blood, which had been pre-warmed to 37°C, at a final test article concentration of 0.1mg/mL. The vials were kept in a 37°C incubator for the duration of the experiment. Aliquots (200 µL) were taken at each time point (0, 5, 10, 15, 20, 30, 60 and 120 minutes) and added to vials which had been pre-filled with 400 µL of acetonitrile (ACN). Samples were stored at 4°C until the end of the experiment. After the final time point was sampled, the vials were mixed and then centrifuged at 18200 x g for 10 minutes. Aliquots of the supernatant were removed, diluted 1:1 into 2% ACN in 0.1% formic acid water, and analyzed by LCMS/MS. The final solution was transferred into polypropylene vials, and then 10 µl was injected and analyzed on a LTQ XL™ Linear Ion Trap mass spectrometry coupled to a Vanquish UHPLC system (Thermo Scientific), with a Accucore™ Vanquish™ C18+ UHPLC Column (1.5 µm, 2.1x50 mm) at 45ºC. A linear gradient of 5-95% acetonitrile in water (0.1% formic acid) was used for 5 min with a flow rate of 0.2 mL/min. Data were analyzed using Xcalibur 3.1 (Thermo Scientific, USA). The peak area response ratio (PARR) to calibration curve was compared to the PARR at time 0 to determine the percent of test article remaining at each time point. Half-lives were calculated using Excel.

4. FSSE and aza-analogue studies

4.1 Materials

Fetal bovine serum was obtained from Gibco BRL (Carlsbad, CA). Acetaminophen was obtained from Sigma (Sigma Aldrich, St. Louis, MO). Alanine transaminase (ALT) and aspartate aminotransferase (AST) assay kits were from BIOO Scientific Corp. (Austin, TX).

4.2 HMGB1 protein preparation
Recombinant cytokine-inducing HMGB1 was expressed in *E. coli* and purified to homogeneity as previously described [9]. HMGB1 was extracted with Triton X-114 to remove any contaminating LPS as described previously. LPS contamination was measured by the Chromogenic Limulus Amebocyte Lysate Assay (Catalog # 50-647U, Lonza, Walkersville, MD). The LPS content in HMGB1 preparations was consistently less than 10 pg/mg protein.

4.3 *In vitro* Studies-Monocyte isolation and cell culture
For monocytes from Male C57BL/6J mice (protocol # #2009-048) from Jackson Labs, splenocytes were enriched using an EasySep Mouse Monocyte Isolation Kit (Stem Cell Technology, 19861). Purified monocytes (2×10^6 cells per mL) were cultured in 96-well plates in X-Vivo 15 serum-free medium (Lonza) with or without the disulfide HMGB1 (1 µg/mL), FSSE or azaF[1]SSazaE[4] (0, 0.1, 1 or 10µM) treatment and cultured at 37°C and 5% CO_2 for 24 hours. For TNF ELISA experiments, a Perkin Elmer Victor3 microplate reader was used to measure absorbance at 450 nm, ELISA data were analyzed using Microsoft Excel and GraphPad Prism Version 9.0.1.

4.4 Animals
Male C57BL/6J mice (6-8 weeks old) for diabetes study were acquired from Taconic Biosciences (Rensselaer, NY) and Male C57BL/6J mice (8-12 weeks old) for APAP study were obtained from the Jackson Laboratory (Bar Harbor, ME), mice were given free access to water and standard rodent chow and were acclimated to their environment for at least 1 week before experimentation. All animal procedures were approved by the Feinstein Institutes for Medical Research Institutional Animal Care and Use Committee (IACUC, protocol #2011-035, 2013-021). Mice were housed in the Center for Comparative Physiology of the Feinstein Institutes for Medical Research under standard temperature, 12 hr light and dark cycle conditions.

4.5 STZ-induced type 1 diabetes in mice
Induction of Diabetes T1D was induced by administration of streptozotocin (STZ) (Sigma-Aldrich) (50 mg/kg dissolved in sodium citrate buffer, pH 4.5) to 6-8 weeks old C57Bl/6J mice intraperitoneally (i.p.) once each day for 5 consecutive days. Mice were given water supplemented with 10% sucrose (Sigma-Aldrich) for six days to prevent sudden hypoglycemia during streptozotocin administration. For studies with FSSE and azapeptide analogue, mice received daily administration of 500 µg of FSSE, azaF[1]SSazaE[4] or saline. Blood glucose with either FreeStyle Lite blood glucose meter (Abbot Diabetes Care, Inc.) or Bayer Contour blood glucose meter (Bayer HealthCare, LLC.) and body weight were measured once weekly. At the end of each study, mice were euthanized by CO_2 asphyxiation. Blood and pancreas were collected for further analysis.

4.5.1 Histology
Pancreas were fixed in 10% formalin and processed for paraffin embedding (AML Laboratory and Genecopoeia). 7-µm-thick sections were stained with hematoxylin/eosin (Genecopoeia) and the degree of insulitis was evaluated microscopically. Islets were analyzed in multiple sections at 20×. Images were captured by Zeiss Axiovert 20-inverted microscope, using the AxioVision V5 software (Zeiss). Insulitis was scored by infiltration grade 0 to 4 (0 = no visible infiltration; 1 = perivascular/periductular infiltrates with leukocytes touching islet perimeters, but not penetrating; 2 = leukocytic penetration of up to 25% of islet mass; 3 = leukocytic penetration of up to 75% of islet mass; 4 = end-stage insulitis)[1]

4.5.2 Serum insulin levels determination
The quantitative estimation of Insulin in mice serum was done by ELISA method.

Statistical Analysis. T1D data were analyzed using GraphPad Prism Version 9.3.1. Data are presented as means ± SEM. Differences between treatment groups were determined by two-way ANOVA, 1-way ANOVA followed by Turkey's multiple comparison test and two-sided Fisher's exact test; p value less than 0.05 was considered statistically significant.

4.6 Acetaminophen-induced liver toxicity in mice
Male C57BL/6J mice were fasted for 12 hours, followed by an injection with acetaminophen (200 mg/kg, injected intraperitoneally). azaF[1]SSazaE[4]or scrambled peptide control was given at 2 and 7 hours post-APAP. Mice were euthanized 24 h after APAP administration; blood and livers were harvested. Hepatotoxicity was examined by activities of serum alanine aminotransferase (ALT) and aspartate aminotransferase (AST) as described before using color endpoint assay kits [9]. In separate experiments, mice (n=15 per group) were fasted for 12 hours and given an injection of acetaminophen (400 mg/kg; injected intraperitoneally) or 0.9% saline as vehicle control. azaF[1]SSazaE[4] (at 50 µg/mouse) or scrambled peptide control (500 µg/mouse)) was administered at 2 h post-acetaminophen (IP), followed by injection once a day for 5 days. Survival was monitored for two weeks.

4.6.1 Serum collection

Whole blood was collected from animals by cardiac puncture following euthanasia by CO_2 asphyxiation. Blood was allowed to clot in a polypropylene tube at room temperature for 60 minutes. To obtain serum, the tubes were centrifuged two times, first at 5000 x g for 10 minutes, followed by 10,000 x g for 2 minutes. The supernatant serum was collected in a clean tube and stored at -20°C until further processing.

4.6.2 Cytokine measurements

Serum levels of TNF and IL-6 (R&D System Inc., Minneapolis, MN) and HMGB1 in mice were measured by commercially obtained ELISA kits (IBL International, Hamburg, Germany). TNF levels from culture supernatants were measured by Mouse TNF-alpha DuoSet ELISA kit (R&D System Inc.).

Statistical analysis. APAP data were analyzed using GraphPad Prism Version 9.3.1. Data are presented as means ± SEM unless otherwise stated. Differences between treatment groups were determined by 2-tailed Unpaired t-test; p value less than 0.05 was considered statistically significant. Differences between treatment groups in animal survival were determined by Kaplan-Meier survival analysis and p value is measured by the log-rank test. P values less than 0.05 were considered statistically significant.

5. Bradykinin and aza-analogues studies

5.1 In vitro PGE2 induction

3T3 Swiss Albino cell line (ATCC CCL-92) was purchased from American Type Culture Collection (Manassas, VA). 3T3 fibroblasts were grown in Dulbecco's Modified Eagle's Medium (DMEM) (ATCC 30-2002) containing 10% heat inactivated fetal bovine serum (Atlanta Biologicals, Flowery Branch, GA) and penicillin-streptomycin (Gibco, Gaithersburg, MD) (DMEM-Complete) in a humidified atmosphere of 95% air and 5% CO2. When monolayers were 80% confluent, cells were washed with serum-free DMEM, trypsinized with 0.25% (w/v) trypsin-0.53mM EDTA solution. Following trypsinization, cells were washed and resuspended in DMEM-Complete at a cell density of 0.03 x 10^6 cells per ml. Cells were seeded into 96 -well flat-bottomed culture plates (6 x 10^3 cells per well in 200 µl). After one day of culture, 3T3 cell monolayers were washed two times in pre-warmed DMEM supplemented with 20 mM HEPES (DMEM-HEPES) and DMEM-HEPES containing DMSO or bradykinin (negative and positive control treatments, respectively) or experimental compounds (tested at 0.9, 2.7, 8.2, 24.6, 74, 222, 666 and 2000 nM) were added to the appropriate wells. Cells were incubated with control and test compounds for 7 minutes at 37°C in a humidified atmosphere of 95% air and 5% CO_2. At the 7-minute time-point supernatants were collected and immediately frozen at -20°C for future PGE2 analyses. PGE2 levels in supernatants were determined using PGE2 ELISA kit (Enzo Life Sciences, Farmingdale, NY) following manufacturer's instructions. Samples were assayed in quadruplicate. PGE2 ELISA data were analyzed using GraphPad Prism Version 9.3.1.

5.2 Surgical procedure and physiological blood pressure measurement

Adult male Sprague Dawley rats (n = 22, weight: 300 – 350 gm; age: 8 – 10 weeks) from Charles River Labs were used under the approval of Institutional Animal Care and Use Committee at The Feinstein Institutes for Medical Research (protocol #2018-011). Rats were anesthetized using isoflurane at 1-3 %. Body temperature was maintained at 37°C using a heating pad. Physiological sensors were placed including electrocardiogram (ECG) leads, nasal temperature sensor, pulse oximeter and rectal temperature probe to capture heart rate, breathing rate, blood oxygen level and body temperature, respectively. All physiological signals were registered using PowerLab, BioAmp and BridgeAmp (ADInstruments, CO, USA), which were recorded on a computer using LabChart v8 software (ADInstruments, CO, USA). Once physiological sensors are placed, a 2 cm skin incision was made at the left femoral triangle and left femoral vein was isolated. A PE-50 tubing was used to catheterize femoral vein using a technique described before [11]. It was used as an intravenous (IV) line for injecting peptides. A 2 cm midline second incision was given on the neck and right carotid artery was isolated, micro-tip catheter (SPR-320, Millar, TX, USA) was used to register arterial blood pressure using previously described technique[12]. Mean arterial pressure (MAP) was calculated using blood pressure. Animal was given sufficient time for stabilization of physiological signals before starting the experiment. In-vivo animal data were analyzed using Matlab 2016a (MathWorks) software, MA, USA.

5.3 Dose preparation

Dose was prepared by following previous bradykinin study[13]. Briefly, a vehicle of isotonic saline (0.9 % NaCl) with 0.1 % BSA was used to dissolve peptides. BSA was used to prevent adsorption of peptide to plastic surfaces. All injections were given as IV boluses of 100 µl followed by 100 µl saline to flush dead space of a catheter. A dose response curve was obtained by IV injections of a peptide in an incremental pattern (0.2, 0.6, 1.6, 3.2, 6.4 µg/kg).

5.4 Blood pressure monitoring experimental procedure

A baseline MAP was measured for a period of 15 min. The experiment was started by injecting saline-BSA vehicle (control), followed by lowest to highest dose of a peptide. After every dose, 20-25 min was given for physiological signals to return to baseline before injecting the next dose. In a few experiments, to study the mechanism of action of peptides used in this study, a selective B2R antagonist, Icatibant (dose: 10 µg/kg (HOE-140, Tocris Bioscience, Bristol, UK)) was used as a pre-treatment 15 min before the injection of peptide. A dose response curve of a peptide was acquired, before and after injecting antagonist. At the end of the experiments, animals were euthanized by exsanguination under deep anesthesia.

Supplementary Discussion

1. Supplementary Table 1: Fmoc-protected thiocarbazate amino acid building blocks (1-16)

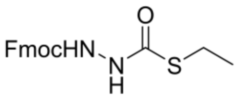<br>azaGly-thiocarbazate (**1**)<br>Yield: 75%; Purity: 96.9%;<br>NMR/MS✓	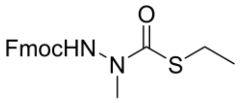<br>azaAla-thiocarbazate (**2**)<br>Yield: 90%; Purity: 97.8%;<br>NMR/MS✓	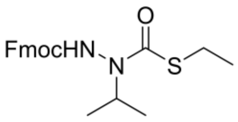<br>azaVal-thiocarbazate (**3**)<br>Yield: 85%; Purity: 96.5%;<br>NMR/MS✓	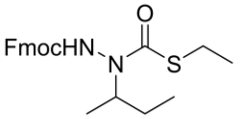<br>azaIle-thiocarbazate (**4**)<br>Yield: 69%; Purity: 99.0%;<br>NMR/MS✓	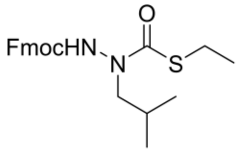<br>azaLeu-thiocarbazate (**5**)<br>Yield: 72%; Purity: 87.3%;<br>NMR/MS✓
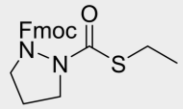<br>azaPro-thiocarbazate (**6**)<br>Yield: 87%; Purity: 91.1%;<br>NMR/MS✓	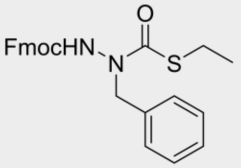<br>azaPhe-thiocarbazate (**7**)<br>Yield: 83%; Purity: 94.4%;<br>NMR/MS✓	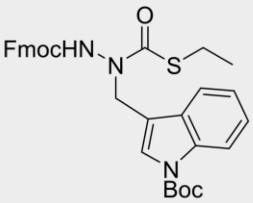<br>azaTrp-thiocarbazate (**8a**)<br>Yield: 88%; Purity: 94.7%;<br>NMR/MS✓	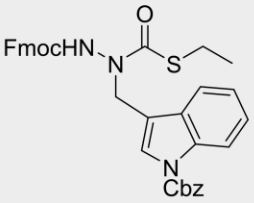<br>azaTrp-thiocarbazate (**8b**)<br>Yield: 83%; Purity: 93.5%;<br>NMR/MS✓	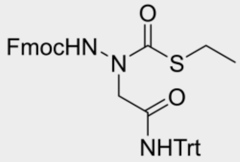<br>azaAsn-thiocarbazate (**9**)<br>Yield: 58%; Purity: 98.9%;<br>NMR/MS✓
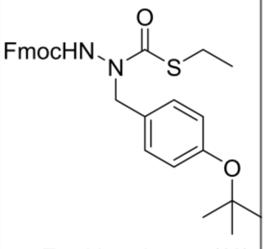<br>azaTry-thiocarbazate (**10**)<br>Yield: 92%; Purity: 98.5%;<br>NMR/MS✓	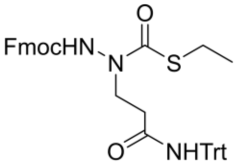<br>azaGln-thiocarbazate (11)<br>Yield: 62%; Purity: 94.6%;<br>NMR/MS ✓	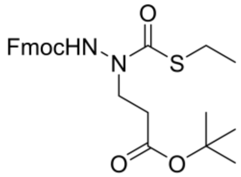<br>azaGlu-thiocarbazate (**12**)<br>Yield: 80%; Purity:92.6%;<br>NMR/MS✓	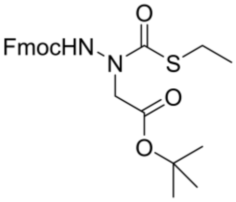<br>azaAsp-thiocarbazate (**13**)<br>Yield: 74%; Purity: 93.4%;<br>NMR/MS✓	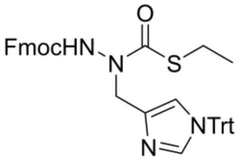<br>azaHis-thiocarbazate (**14**)<br>Yield: 33%; Purity: 87.9%;<br>NMR/MS✓
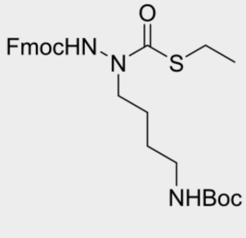<br>azaLys-thiocarbazate (**15**)<br>Yield: 54%; Purity: 87.4%;<br>NMR/MS✓	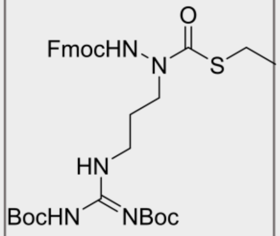<br>azaArg-thiocarbazate (**16**)<br>Yield: 61%; Purity: 96.3%;<br>NMR/MS✓			

2. **Supplementary Table 2: Ex-vivo stabilities (percent remaining) and half-lives (minute) of FSSE-based azapeptides (51-58) (a) and bradykinin-based azapeptides (62-71) (b) in male C57BL/6J mouse whole blood.**

a

Test article	Percent remaining							Half-life (min) $t_{1/2}$
	0 (min)	5	10	20	30	60	120	
FSSE (P5779)*	100	15.9	3.06	<1.0	<1.0	<1.0	<1.0	2.0 ± 0.1
azaF^{1}SSazaE^4 **51**	100	103.5	92.7	86.6	94.8	97.4	104.5	> 120
azaF^{1}SSE-NH$_2$ **52**	100	82.6	80.2	72.7	78.9	79.2	95.1	> 120
FSSazaE^4-NH$_2$ **53**	100	13.5	<1.0	<1.0	<1.0	<1.0	<1.0	2.6 ± 0.11
azaF^{1}SSE-OH **54**	100	103.6	91.6	94.1	84.9	72.3	56.4	> 120
azaF^{1}SSQ-OH **55**	100	87.7	79.3	60.0	44.4	20.6	4.2	24.4 ± 1.6
azaF^{1}SSQ-NH$_2$ **56**	100	94.4	97.1	90.4	95.7	93.2	96.4	> 120
azaF^{1}SSazaQ^4-NH$_2$ **57**	100	101.2	98.4	92.4	92.6	96.1	114.3	> 120
FSSazaQ^4-NH$_2$ **58**	100	21.7	3.84	<1.0	<1.0	<1.0	<1.0	2.9 ± 0.2

b

Test article	Percent remaining							Half-life (min) $t_{1/2}$
	0 (min)	5	10	20	30	60	120	
Bradykinin	100	53.4	2.85	<1.0	<1.0	<1.0	<1.0	5.29 ± 0.5
azaR^1-BK **62**	100	8.8	<1.0	<1.0	<1.0	<1.0	<1.0	2.46 ± 0.05
azaP^2-BK **63**	100	52.9	25.2	9.0	<1.0	<1.0	<1.0	5.4 ± 1.1
azaP^3-BK **64**	100	13.5	1.24	<1.0	<1.0	<1.0	<1.0	2.63 ± 0.25
azaG^4-BK **65**	100	41.7	4.75	<1.0	<1.0	<1.0	<1.0	4.33 ± 1.3
azaF^5-BK **66**	100	74.0	58.3	28.5	<1.0	<1.0	<1.0	14.7 ± 1.6
azaP^7-BK **67**	100	6.1	2.4	<1.0	<1.0	<1.0	<1.0	2.38 ± 0.03
azaF^8-BK **68**	100	92.4	83.2	78.5	66.8	23.5	3.1	39.3 ± 2.5
azaR^9-BK **69**	100	88.6	80.7	71.9	64.7	34.1	1.8	42.6 ± 0.8
[azaP^2, azaF^8]-BK **70**	100	87.5	80.8	61.4	49.9	19.9	<1.0	29.2 ± 3.8
[azaF^5, azaF^8]-BK **71**	100	94.3	92.6	83.7	79.5	70.8	43.0	105.8 ± 1.8

Analyzed by LCMS/MS, the peak area response ratio (PARR) to calibration curve was compared to the PARR at time 0 to determine the percent of test article remaining at each time point; Half-lives were calculated using excel. $T_{1/2}$ results are means ± SD from three blood samples, each data point was run in duplicate.

3. **Supplementary Table 3: The ratio of different secondary structures of bradykinin and bradykinin-based azapeptides (63-71)**

		a-helix	Antiparallel β-structure	parallel β-structure	β-turn	Random coil
Bradykinin		11.18%	16.41%	14.20%	18.29%	39.92%
azaP^{2}-BK	**63**	12.53%	15.62%	13.78%	18.30%	39.77%
azaP^{3}-BK	**64**	8.99%	16.69%	15.32%	17.45%	41.54%
azaG^{4}-BK	**65**	12.84%	16.07%	13.52%	18.79%	38.78%
azaF^{5}-BK	**66**	11.97%	16.13%	13.88%	18.45%	39.57%
azaP^{7}-BK	**67**	10.55%	16.99%	14.41%	18.36%	39.69%
azaF^{8}-BK	**68**	10.69%	16.84%	14.33%	18.38%	39.76%
azaR^{9}-BK	**69**	12.03%	16.74%	13.71%	18.92%	38.52%
[azaP^{2}, azaF^{8}]-BK	**70**	11.17%	15.95%	14.33%	17.98%	40.57%
[azaF^{5}, azaF^{8}]-BK	**71**	10.99%	16.53%	14.25%	18.32%	39.98%

Bradykinin analogues were scanned from 190 to 260 nm on CD spectrometer ((Applied Photophysics Ltd, UK); for 3 repeats per peptide. The CD spectra were plotted by using the average value of 3 repeats. All samples were dissolved in PBS to a final concentration of 0.3 mg/mL. The analysis parameters were set as follows: the path length was 10 nm; the scan speed was set to 0.05 s/point, the temperature was set to 20 °C. The secondary structures were determined by CDNN 2.1 (Circular Dichroism analysis using Neural Networks) software and were processed with Prism X (GraphPad Software Inc.), all spectra were plotted by using the average value of 3 repeats.

4. Supplementary Table 4

Optimized reaction conditions for the thiocarbazates activation and coupling in solution-phase chemistry

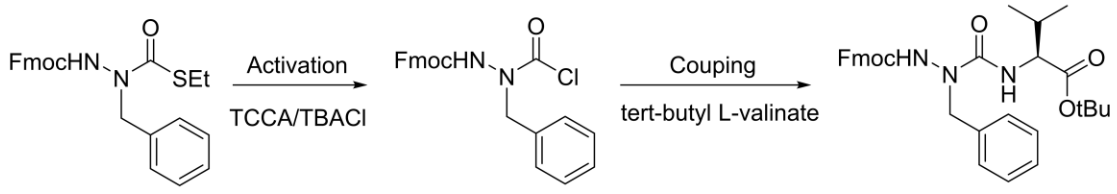

aza-Phe thiocarbazate **7** | Reactive acyl chloride Fmoc-azaF(Cl) **17** | azadipeptide azaF[1]V **18**

Entry	TBACl (equiv)	TCCA (equiv)	Amine (equiv)	Et_3N (equiv)	Temp	Activation time (min)	Coupling time (min)	Isolated yield	% of chlorinated side product
1	0	1.0	1.5	2.0	rt	5	60	19.0%	N.D
2	0	1.0	2.1	4.4	0 °C	10	60	51.8%	N.D
**3**	**1.0**	**1.0**	**1.5**	**4.0**	**rt**	**5**	**45**	**59.6%**	**11.6%**
4	1.0	1.0	1.5	4.0	0 °C-rt	10	30	55.4%	5.6%
5	1.0	1.0	1.5	4.0	0 °C	10	60	77.4%	1.1%
6	1.5	1.0	2.1	4.4	0 °C	10	60	83.2%	3.2%
7	1.5	0.75	2.1	4.4	0 °C	10	60	79.8%	1.1%
**8**	**1.5**	**0.85**	**2.1**	**4.4**	**0 °C**	**10**	**60**	**88.5%**	**1.4%**

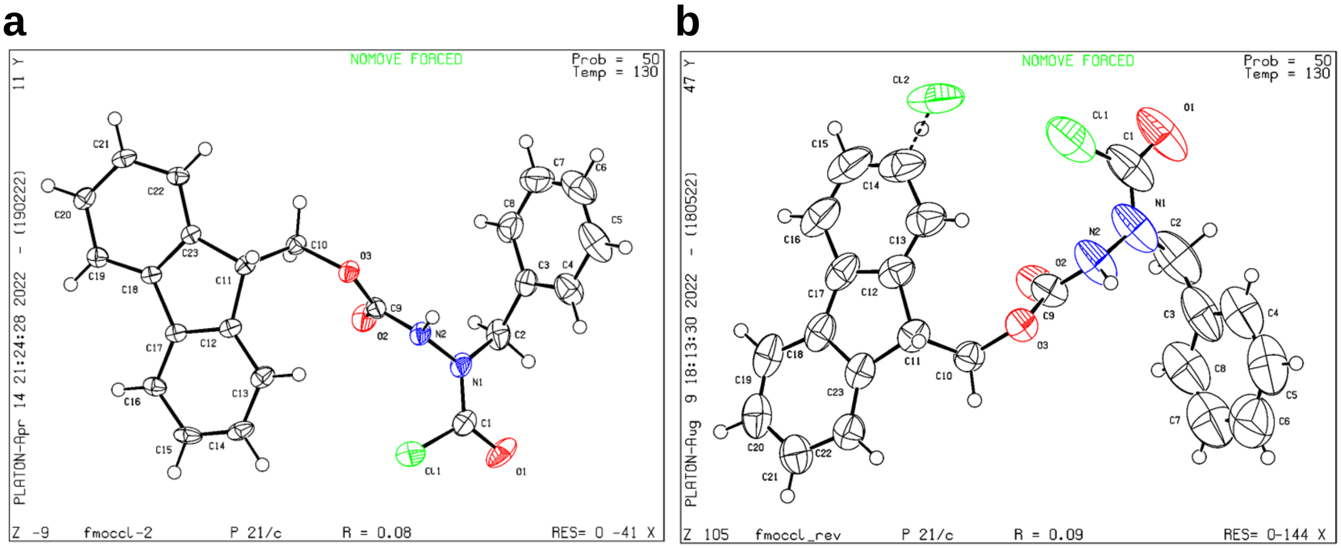

X-ray analysis for Fmoc-azaPhe chloride **17** (activation at 0°C, entry 8)

X-ray analysis for Fmoc-azaPhe chloride **17** (activation at rt, entry 3)

5. Supplementary Figure 1

X-ray crystal structures of Fmoc-azaPhe chloride.

X-ray analysis of **17** a) entry 8 of Supplementary Table 4 (CCDC-2195262) showed no disorder at all this time – there is no Cl substitution on the Fmoc group; X-ray analysis of **17** b) entry 3 of Supplementary Table 4 (CCDC-2195263) showed that,

there is a disorder between the Fmoc-unsubstituted C-H product (78% occupancy) and the Fmoc-substituted C-Cl product (22% occupancy). The R value is somewhat high (9%), and some of the thermal ellipsoids are elongated. This maybe because there are actually two different molecules present. The C-H and C-Cl packing is similar enough that modeling full molecule disorder did not lead to a stable refinement, but different enough that you can see the imperfections. It is likely that the chlorinated side product is enriched during the crystal formation as the sample submitted to the X-ray analysis was not representative.

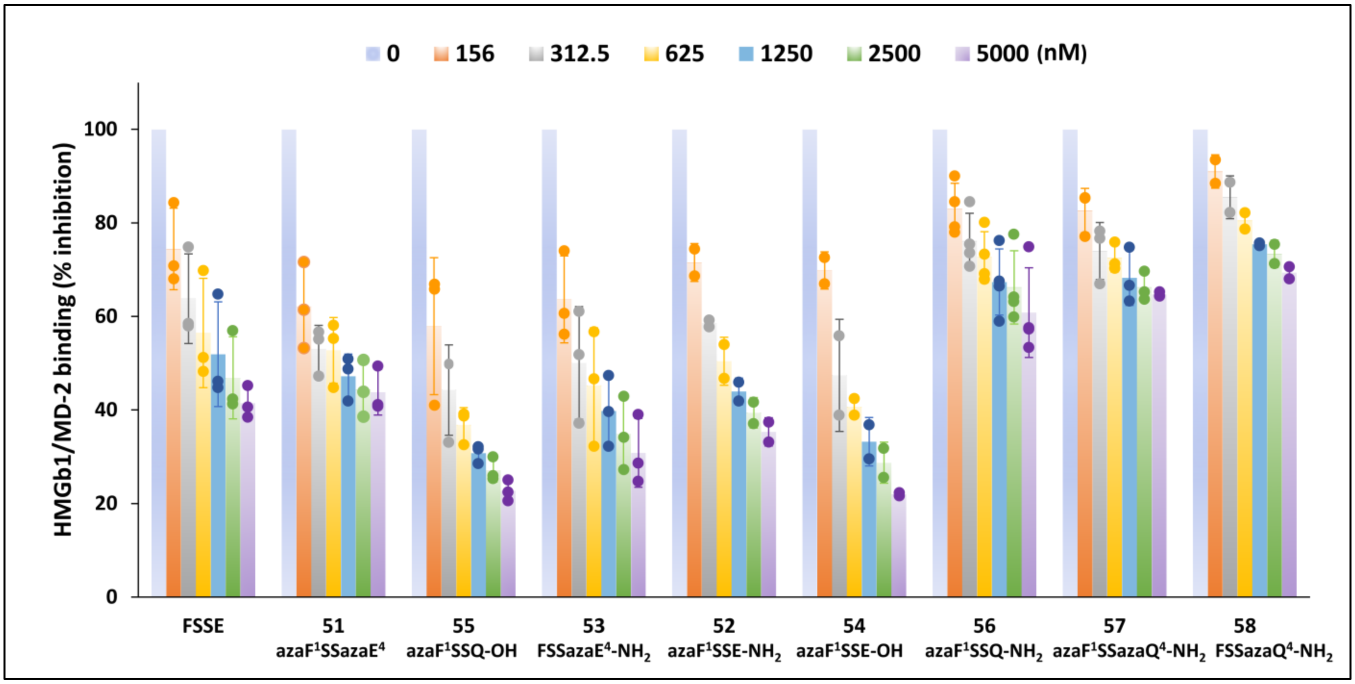

6. **Supplementary Figure 2**

FSSE-based azapeptides (51-58) inhibit HMGB1/MD-2 directly binding.

Surface plasmon resonance (SPR) analysis

Biacore T200 (Cytiva, GE Healthcare, USA) was used for real-time binding interaction studies. Recombinant human TLR4/MD2 (R&D 3146-TM-050) complex protein and MD2 (R&D1787-MD) protein were purchased from R&D Systems (Minneapolis, USA). For binding analyses, CM5 series chips (GE Healthcare) were activated and the reference flow-cell was blocked by 1 methanolamine (pH = 8.5). The ligand protein the disulfide HMGB1 (20 µg/mL in 10 mM acetate buffer pH = 4.5) was immobilized onto a CM5 at a flow rate of 10 µL/min until the surface Plasmon resonance reached 400-500 RU. MD-2 (0.25 µM) was incubated with or without FSSE analogues (the concentrations were varied (2X) from 5uM to 156 nM) for 15 minutes prior to injection of the mixture on the chip at 30 µL/min for 60s. The dissociation time was set for 1 minute. Binding experiments were conducted in 1XPBS + 0.05% Tween 20 as the running buffer, and at least 2 independent experiments were performed. IC_{50} was obtained to evaluate the inhibitory effect by using the BIAEvaluation 3.1 software (Cytiva, GE Healthcare). Experimental data are expressed as means ±SD for triplicate estimates of individual experiments (n = 2-4 experiments). differences between individual experiments for each compound were determined by single factor ANOVA.
FSSE: n = 3, P-value = 0.0110; Compound **51**: n = 3, P-value = 0.0338; Compound **55**: n = 3, P-value = 0.0010; Compound **53**: n = 3, P-value = 0.0151; Compound **52**: n = 2, P-value = 0.00035; Compound **54**: n = 2, P-value = 0.001958; Compound **56**: n = 4, P-value = 0.0048; Compound **57**: n = 3, P-value = 0.0031; Compound **58**: n = 2, P-value = 0.00239.

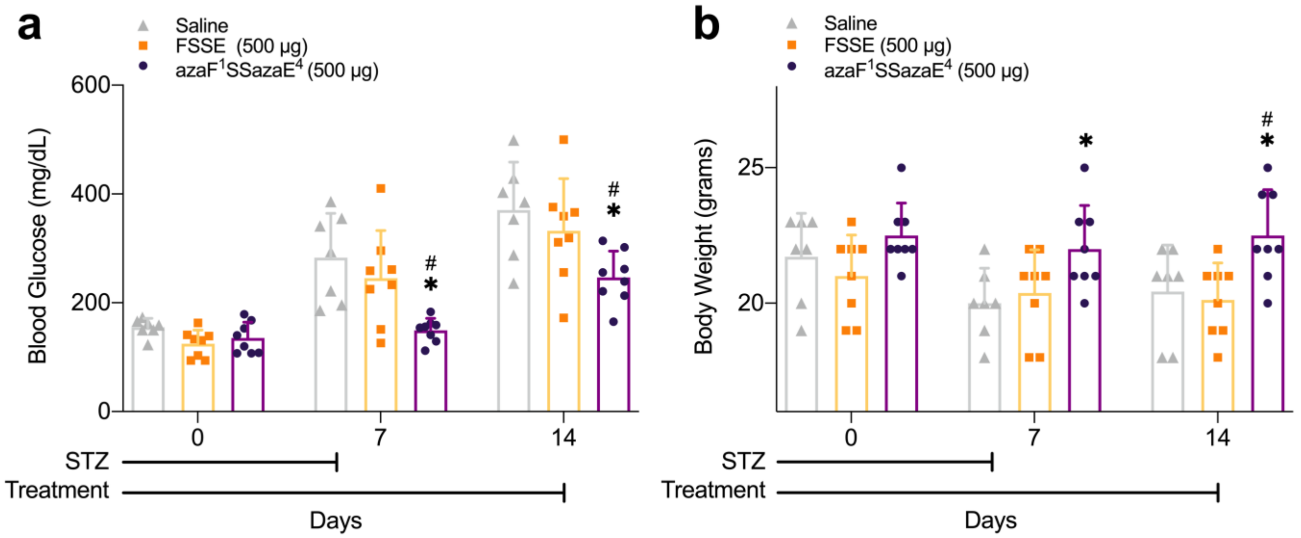

7. Supplementary Figure 3

HMGB1/MD-2/TLR4 antagonist azapeptide analogue azaF^{1}SSazaE^4 delays hyperglycemia in mice challenged with low-dose streptozotocin.

C57Bl/6J male mice (6 weeks of age) were injected i.p. with STZ (50mg/kg) in sodium citrate buffer (pH 4.5) once daily for 5 days. Mice were simultaneously injected i.p. with saline, FSSE (500µg/mouse/day), or azaF^{1}SSazaE^4 (500µg/mouse/day). **a**. Blood glucose was measured on day 0, 7 and 14. Number of mice per group: saline n = 7; FSSE n = 8; azaF^{1}SSazaE^4 n = 8. Data are presented as mean values ± SEM. Values are measured by two-way ANOVA *p = 0.0004 vs. saline group on day 7, *p = 0.0009 vs. saline group on day 14; #p = 0.0094 vs. FSSE group on day 7, #p = 0.0218 vs. FSSE group on day 14. **b**. Body weight was measured on day 0, 7 and 14. Number of mice per group: saline n = 7; FSSE n = 8; azaF^{1}SSazaE^4 n = 8. Data are presented as mean values ± SEM. Values are measured by two-way ANOVA *p = 0.0352 vs. saline group on day 7, *p = 0.0281 vs. saline group on day 14; #p = 0.0074 vs. FSSE group on day 14.

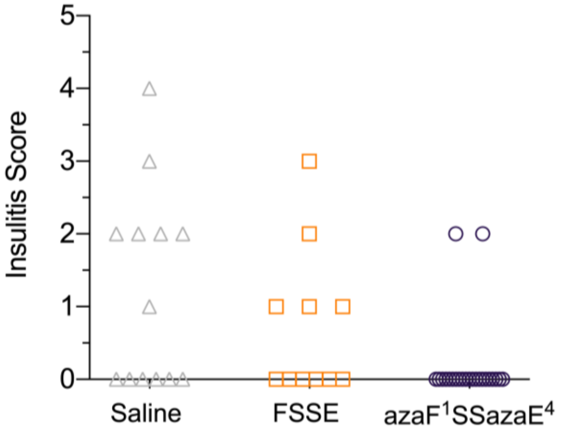

8. Supplementary Figure 4

Distribution of insulitis score of H&E stained pancreatic islets from the indicated group.

Islets per group: saline n = 3, 13 islets; FSSE n = 2; 11 islets; azaF^{1}SSazaE^4 n = 3, 23 islets. Insulitis was scored by infiltration grade 0 to 4 (0 = no visible infiltration; 1 = perivascular/periductular infiltrates with leukocytes touching islet perimeters, but not penetrating; 2 = leukocytic penetration of up to 25% of islet mass; 3 = leukocytic penetration of up to 75% of islet mass; 4 = end-stage insulitis)[1, 2].

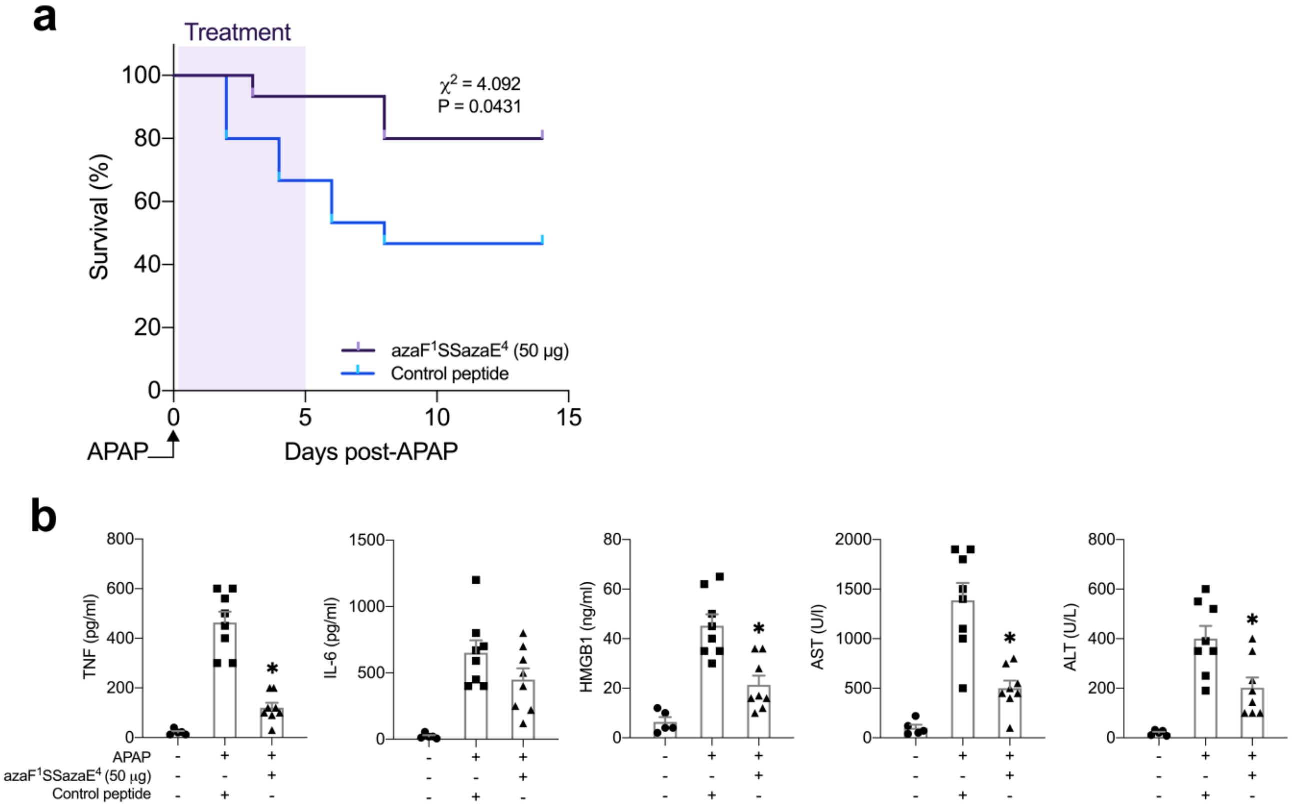

9. Supplementary Figure 5

azaF1SSazaE4 improves survival in acetaminophen toxicity (APAP) model.

a. Male C57BL/6J mice (8-12 weeks of age) were fasted for 12 hours and received acetaminophen byproduct APAP (400mg/kg); azaF1SSazaE4 (at 50 µg/mouse, or scrambled peptide control SFSE-amide) was administered at 2 h post-APAP (IP), followed by injection once a day for 5 days (n = 15 per group). Kaplan-Meier survival curves of mice treated with azaF1SSazaE4 or control peptide were compared by the log-rank test (chi-squared and p value in figure legend). **b**. azaF1SSazaE4 reduced APAP-induced pro-inflammatory markers and serum liver enzymes. C57BL/6J mice (male, 8-12 weeks of age) were fasted for 12 hours and received APAP injected (IP, 200 mg/kg). azaF1SSazaE4 (at 50 µg/mouse) or scrambled peptide control SFSE-amide (500µg/mouse) was given at 2 and 7 hours post-APAP. Mice were euthanized 24 h after APAP administration (Number of mice per group: control vehicle n = 5; control peptide n = 8; azaF1SSazaE4 n = 8). Data are presented as means ± SEM. Differences between treatment groups were determined by 2-tailed Unpaired t-test, p value less than 0.05 was considered statistically significant. *p = 0.0001 vs. control peptide group for TNF, *p = 0.0012 vs. control peptide group for HMGB1, *p = 0.0004 vs. control peptide group for AST, *p = 0.0098 vs. control peptide group for ALT.

In this experiment, azaF1SSazaE4 was compared in parallel with scrambled peptide in the acute (APAP) model instead of using FSSE as control, and further studies on the equivalency or superiority of azaF1SSazaE4 in this model are ongoing.

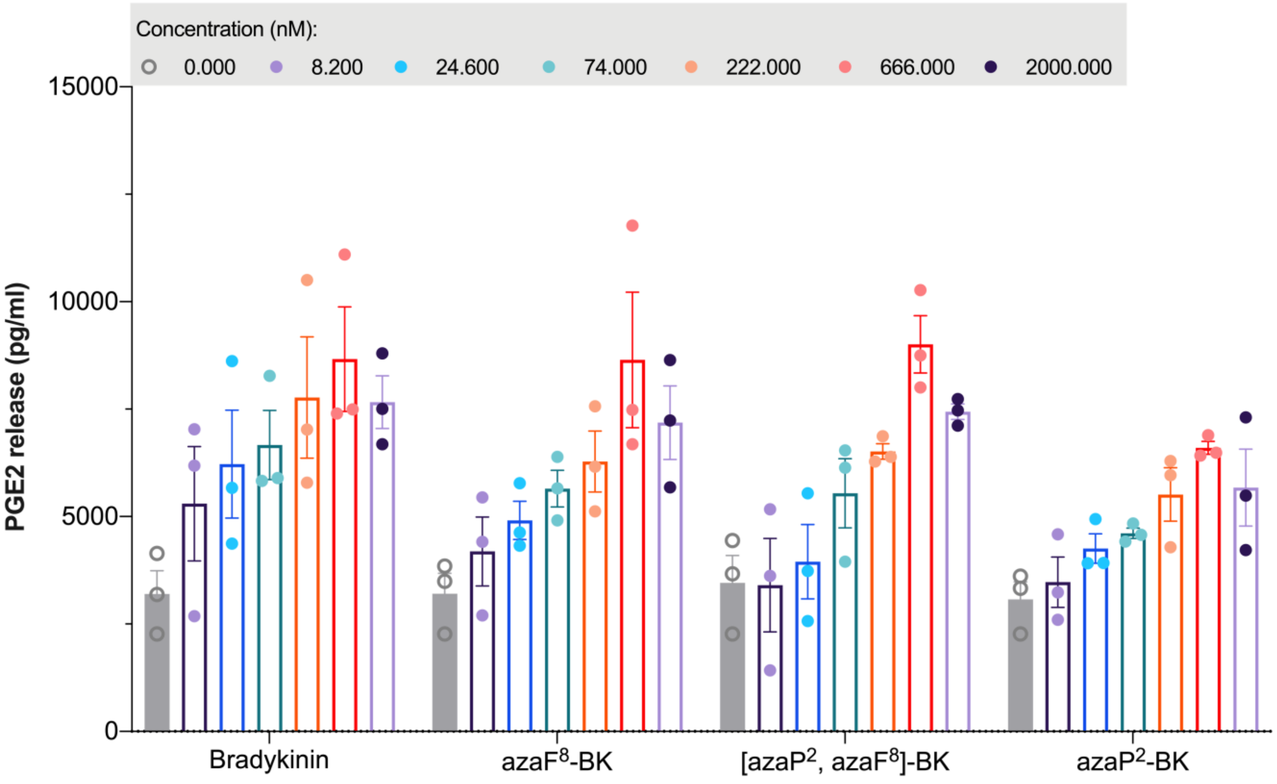

10. Supplementary Figure 6

Alternative bar plot representation of the dose-dependent PGE2 release data plotted in Figure 8b of the main text.

This alternative bar plot representation shows individual data points for each of the 3 experiments used to calculate the mean ± SEM PGE2 release data that is plotted in Figure 5b of the main text. This alternative plot also shows the amount of PGE2 released by control-treated cells (filled grey bar; PGE2 release by cells treated with DMSO, the solvent used to dilute bradykinin and bradykinin azapeptide analogues) for each experiment and treatment). Note, PGE2 release by control-treated 3T3 cells was not significantly different from PGE2 released by untreated cells.

Compound I.D.	Client Compound I.D.	IC_{50}(M)	K_i(M)	nH	Test Concentration	% Inhibition of Control Specific Binding 1st	2nd	Mean
**B_2 *(h)* (agonist radioligand)**								
100053815-1	[azaP2, azaF8]-BK	**1.1E-08 M**	5.6E-09 M	1.0	1.0E-09 M	-9.2	8.0	-0.6
					1.0E-08 M	36.3	48.4	42.3
					3.0E-08 M	67.6	73.7	70.6
					1.0E-07 M	84.7	91.4	88.1
					1.0E-06 M	97.1	99.5	98.3
100053815-3	azaF8-BK	**8.8E-09 M**	4.5E-09 M	1.3	1.0E-09 M	0.2	-3.4	-1.6
					1.0E-08 M	58.5	56.4	57.4
					3.0E-08 M	86.3	63.9	75.1
					1.0E-07 M	94.6	95.1	94.9
					1.0E-06 M	98.9	98.6	98.8
100053815-2	azaP2-BK	**7.9E-09 M**	4.1E-09 M	1.0	1.0E-09 M	8.0	2.9	5.4
					1.0E-08 M	61.5	66.7	64.1
					3.0E-08 M	72.4	64.6	68.5
					1.0E-07 M	98.0	95.2	96.6
					1.0E-06 M	99.7	100.1	99.9

a

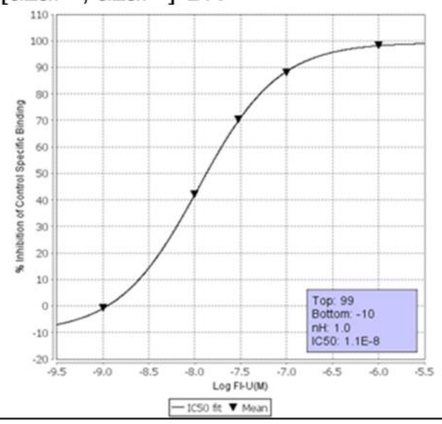

b

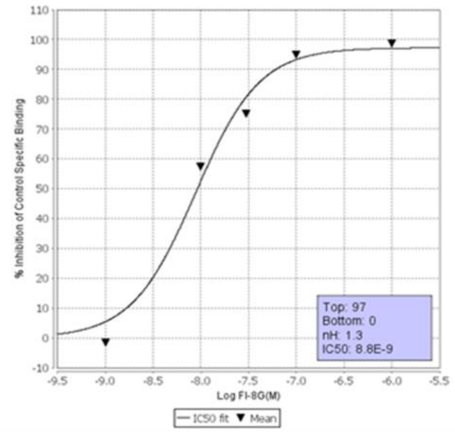

c

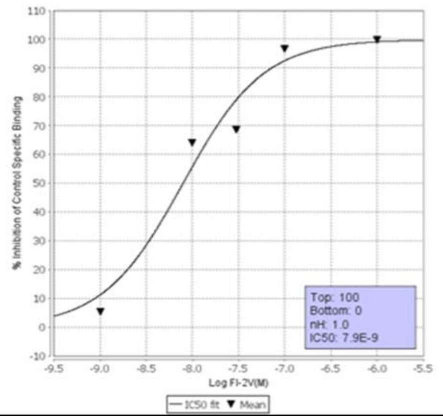

11. Supplementary Figure 7

In vitro pharmacology: Human B2 (h) (agonist radioligand) receptor binding assay for lead bradykinin azapeptides a, [azaP[2], azaF[8]]-BK (70); b, azaF[8]-BK (68); c, azaP[2]-BK (63).

The data was collected by CRO Eurofins Cerep (France) and the result is attached as an additional data file. B2R radioligand agonist binding displacement assay for determination of IC-50.

Test compounds (FI-U / [azaP^2, azaF^8-BK (**70**), FI-2V / azaF^8-BK (**68**), and FI-8G / azaP^2-BK (**63**)) were supplied to Eurofins in a blinded manner and run at five concentrations to determine the IC-50's. Standard company control test compounds were used for assay validation. Experimental assays achieved inhibition of more than 50% and were considered to represent significant effects of the test compounds. Tritium-labeled bradykinin (B2(h)) was used as the agonist for B2R receptor binding. The results are expressed as a percent of control specific binding (measured specific binding /control specific binding)*100, and as a percent inhibition of control specific binding (100 minus (measured specific binding/ control specific binding)*100) obtained in the presence of the test compounds. The IC50 values (concentration causing a half-maximal inhibition of control specific binding) and Hill coefficients (nH) were determined by non-linear regression analysis of the competition curves generated with mean replicate values using Hill equation curve fitting $Y=D+[A-D / 1+(C/C50)^{nH}]$ where Y = specific binding, A = left asymptote of the curve, D = right asymptote of the curve, C = compound concentration, C50 = IC50, and nH = slope factor. This analysis was performed using software developed at Cerep (Hill software) and validated by comparison with data generated by the commercial software SigmaPlot® 4.0 for Windows® (© 1997 by SPSS Inc.). The inhibition constants (Ki) were calculated using the Cheng Prusoff equation. Ki=IC50/(1+L/Kd) where L = concentration of radioligand in the assay, and Kd = affinity of the radioligand for the receptor. A scatchard plot is used to determine the Kd.

Supplementary Notes

1. X-ray crystallographic data

1.1 Crystal data and structure refinement for compound 17 (activate at rt)

X-ray diffraction data were collected on a Bruker D8 VENTURE diffractometer using Cu Kα radiation. Crystal data, data collection and refinement parameters are summarized in Table 1. The structure was solved using a dual-space method and standard difference map techniques and was refined by full-matrix least-squares procedures on F^2 with SHELXTL (Version 2018/3)[14, 15]. All hydrogen atoms bound to carbon were placed in calculated positions and refined with a riding model [U_{iso}(H) = 1.2–1.5U_{eq}(C)], while the hydrogen atom bound to nitrogen was located on the difference map and freely refined.

Supplementary Table 5. Crystal, intensity collection, and refinement data.

	**Fmoc- azaPhe chloride (activation at rt)**
lattice	Monoclinic
formula	$0.78(C_{23}H_{19}ClN_2O_3)\bullet0.22(C_{23}H_{18}Cl_2N_2O_3)$
formula weight	414.43
space group	$P2_1/c$
a/Å	5.4730(2)
b/Å	11.3092(5)
c/Å	32.9130(14)
α/°	90
β/°	94.351(2)
γ/°	90
V/Å^3	2031.29(15)
Z	4
temperature (K)	130(2)
radiation (λ, Å)	1.54178
ρ (calcd.) g cm^{-3}	1.355
μ (Cu Kα), mm^{-1}	2.156
θ max, deg.	75.630
no. of data collected	50347
no. of data	4172
no. of parameters	276
R_1 [$I > 2\sigma(I)$]	0.0891
wR_2 [$I > 2\sigma(I)$]	0.2558
R_1 [all data]	0.1048
wR_2 [all data]	0.2715
GOF	1.061
R_{int}	0.1006

1.2 Crystal data and structure refinement for compound 17 (activate at 0 °C)

X-ray diffraction data were collected on a Bruker D8 VENTURE diffractometer using Cu Kα radiation. Crystal data, data collection and refinement parameters are summarized in Table 2. The structure was solved using a dual-space method and standard difference map techniques and was refined by full-matrix least-squares procedures on F^2 with SHELXTL (Version 2018/3)[14, 15]. All hydrogen atoms bound to carbon were placed in calculated positions and refined with a riding model [U_{iso}(H) = 1.2–1.5U_{eq}(C)], while the hydrogen atom bound to nitrogen was located on the difference map and freely refined.

Supplementary Table 6. Crystal, intensity collection, and refinement data.

	**Fmoc-azaPhe chloride (activation at 0 °C)**
lattice	Monoclinic
formula	$C_{23}H_{19}ClN_2O_3$
formula weight	406.85
space group	$P2_1/c$
a/Å	26.2852(12)
b/Å	5.5828(3)
c/Å	13.4292(6)
α/°	90
β/°	90.105(3)
γ/°	90
V/Å^3	1970.66(16)
Z	4
temperature (K)	130(2)
radiation (λ, Å)	1.54178
ρ (calcd.) g cm^{-3}	1.371
μ (Cu Kα), mm^{-1}	1.944
θ max, deg.	74.423
no. of data collected	50544
no. of data	3999
no. of parameters	266
R_1 [$I > 2\sigma(I)$]	0.0754
wR_2 [$I > 2\sigma(I)$]	0.1980
R_1 [all data]	0.0830
wR_2 [all data]	0.2036
GOF	1.081
R_{int}	0.1385

2. Copy of NMR spectra -Supplementary Figures 8-60

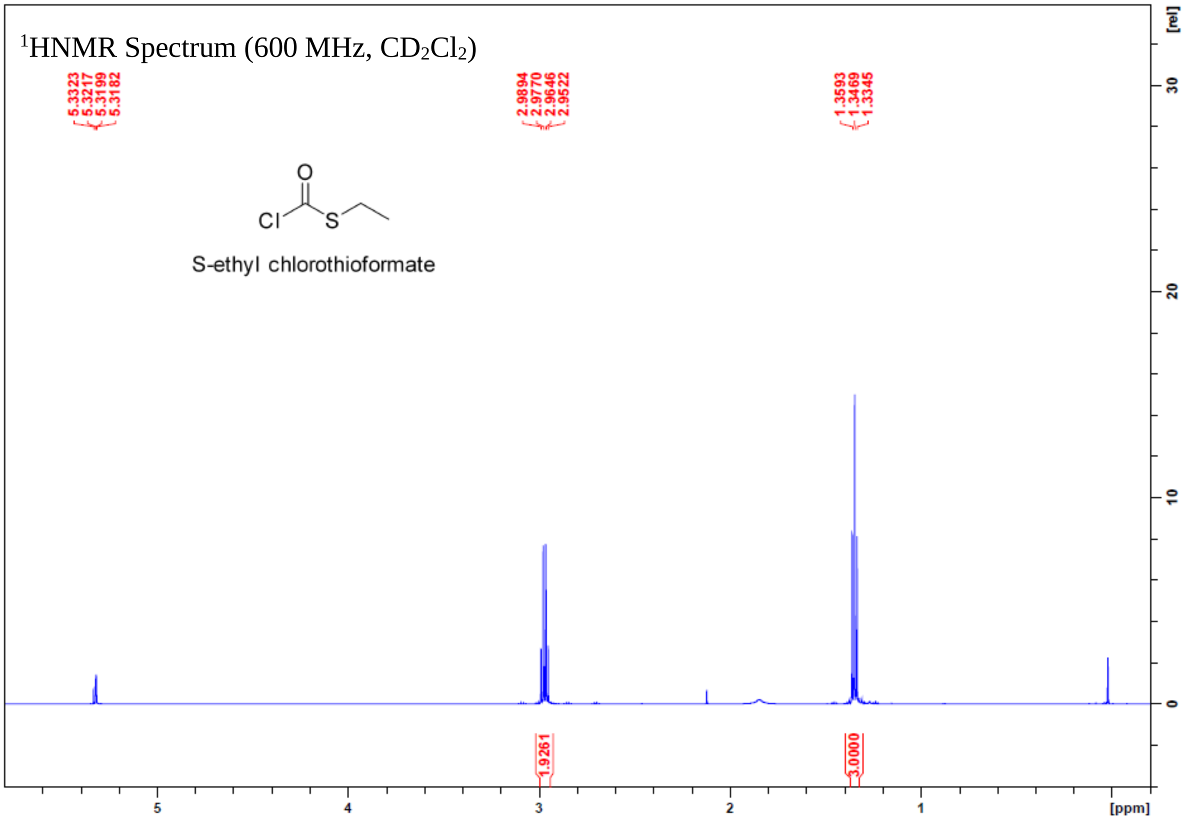

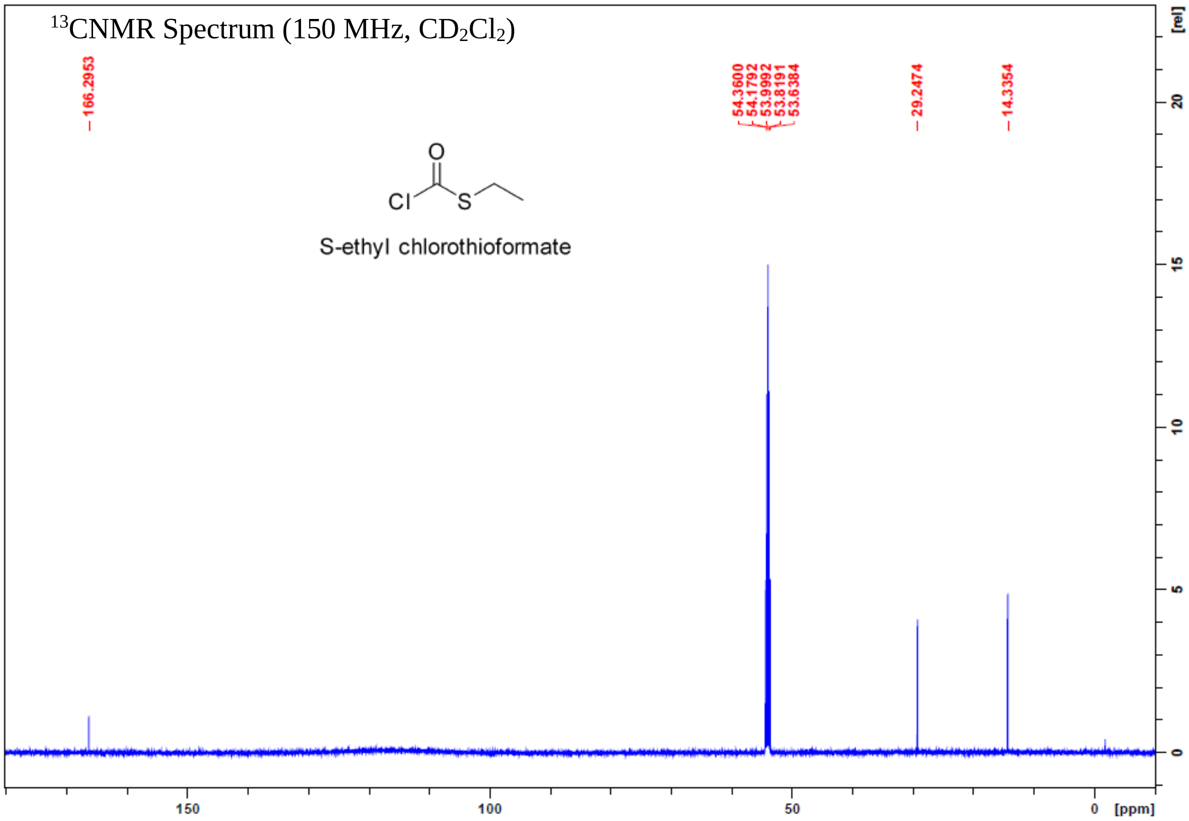

Supplementary Figure 8. NMR spectra of S-ethyl chlorothioformate.

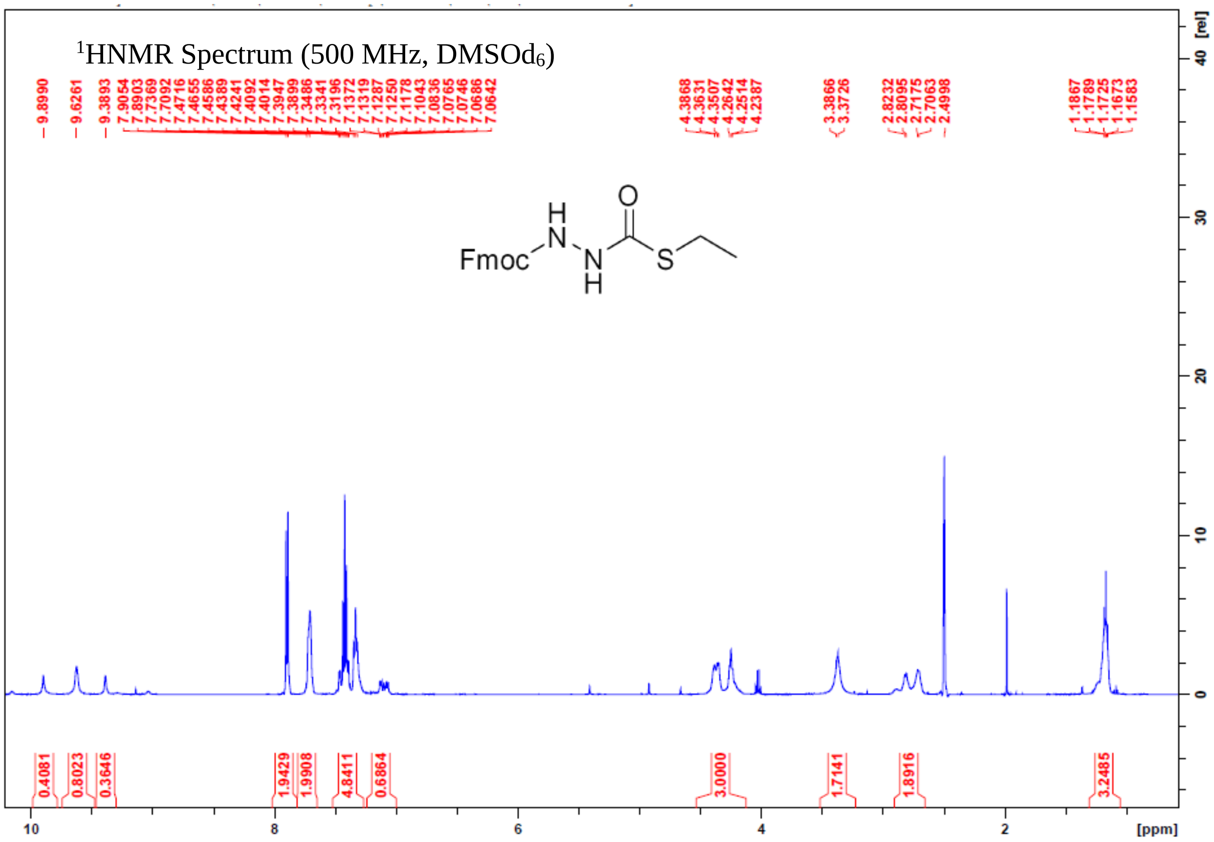

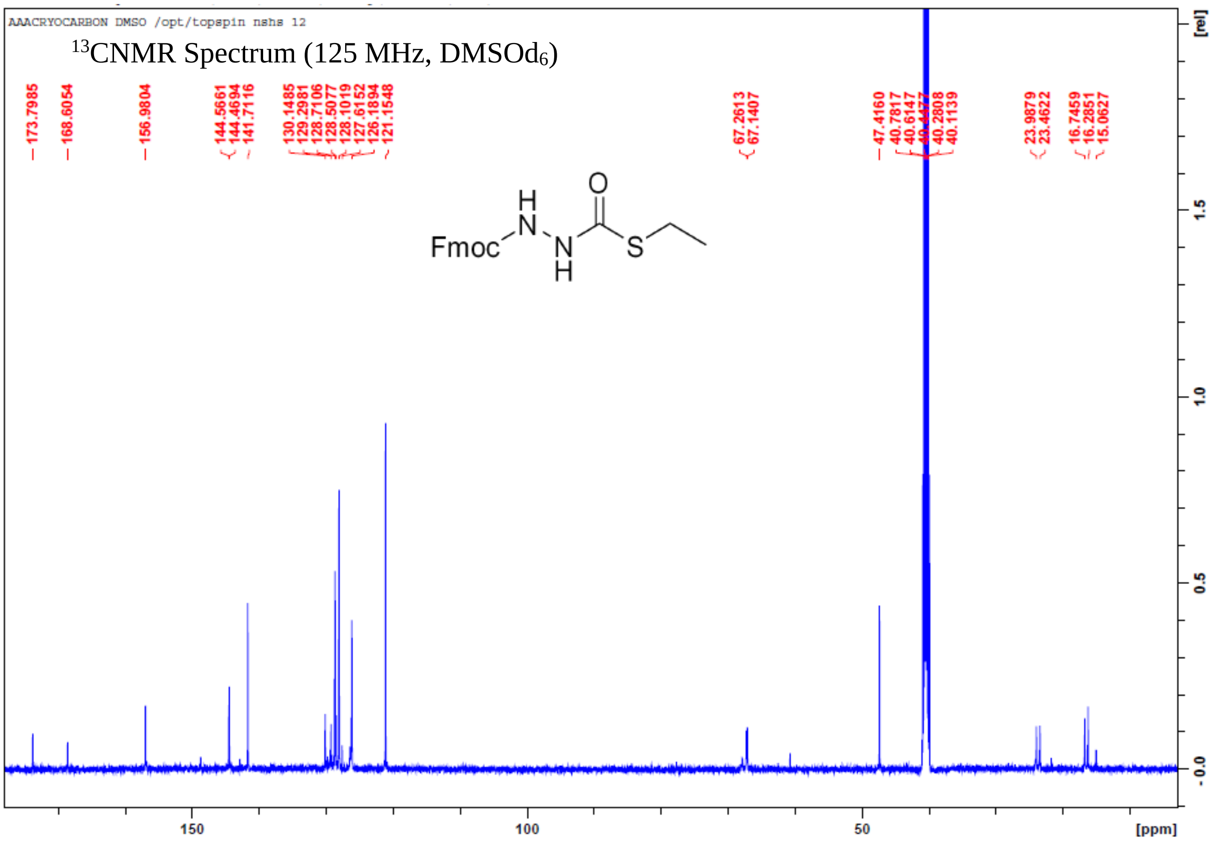

Supplementary Figure 9. NMR spectra of compound 1.

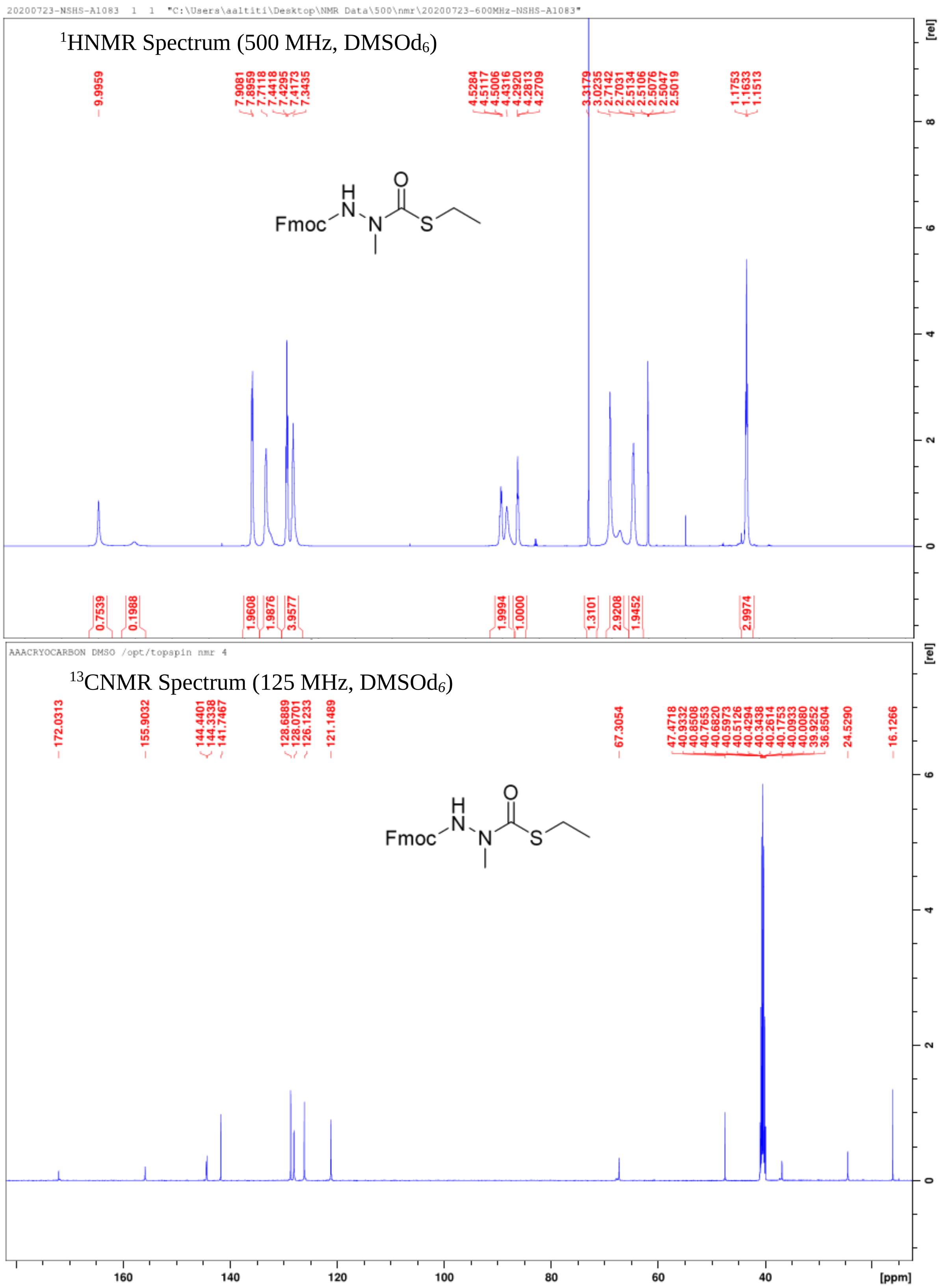

Supplementary Figure 10. NMR spectra of compound 2.

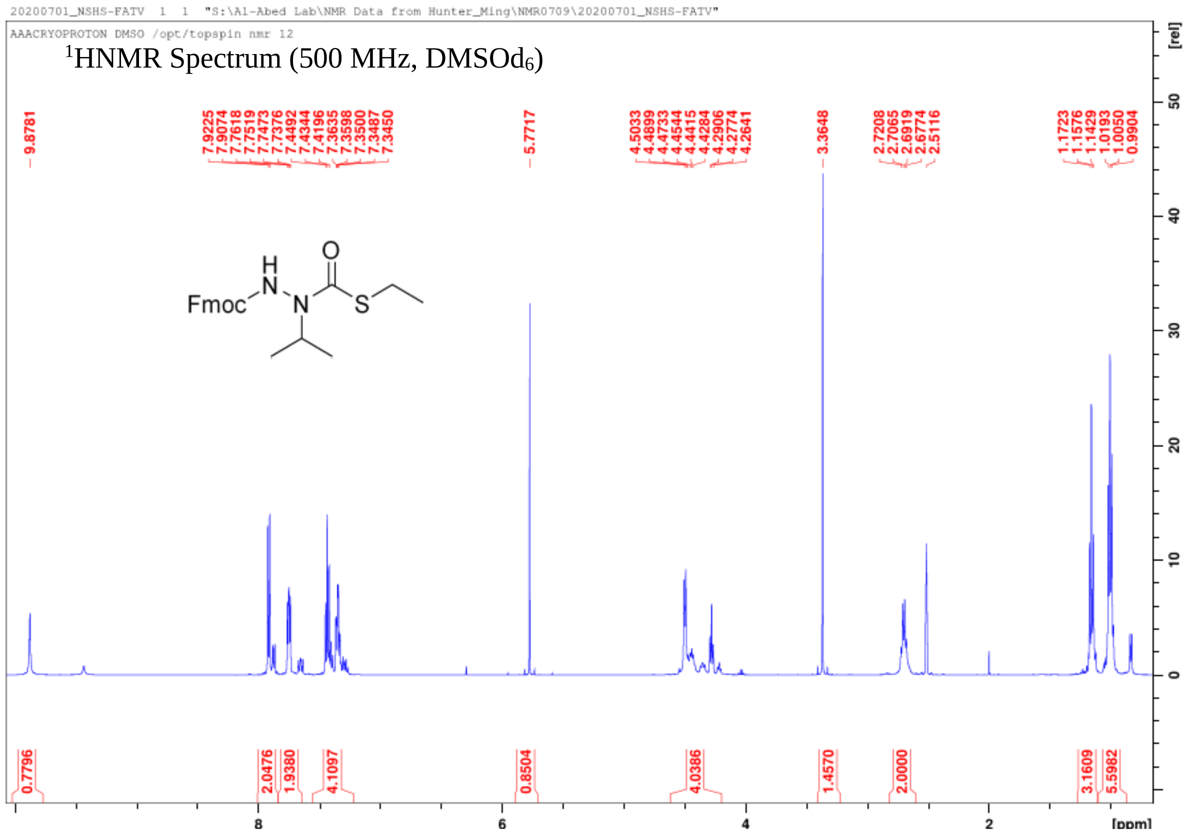

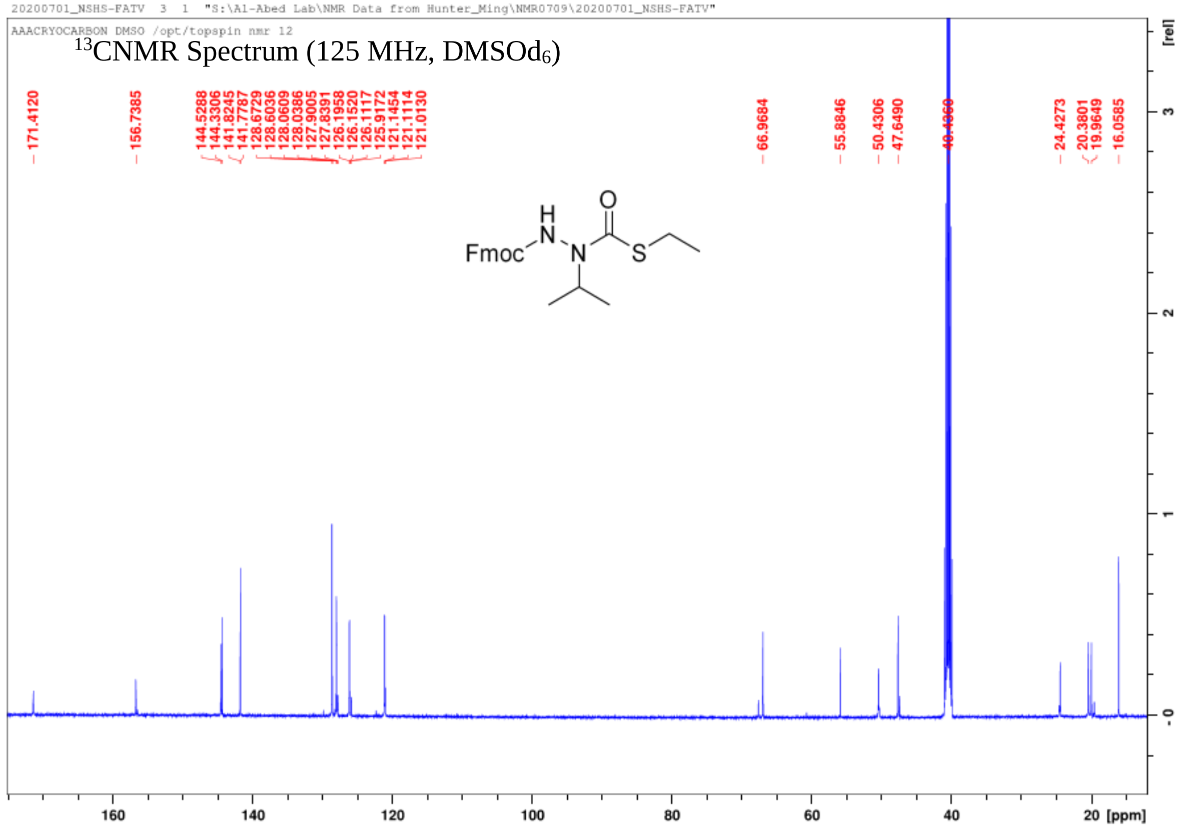

Supplementary Figure 11. NMR spectra of compound 3.

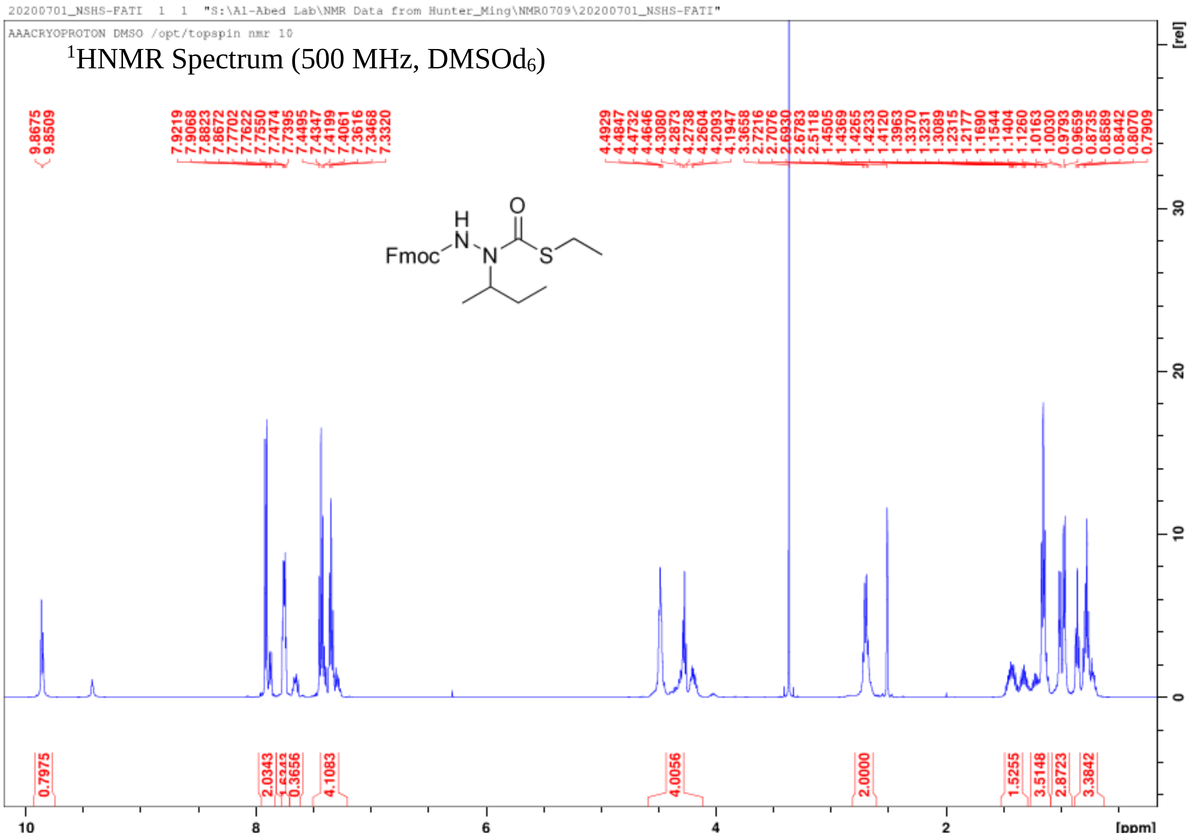

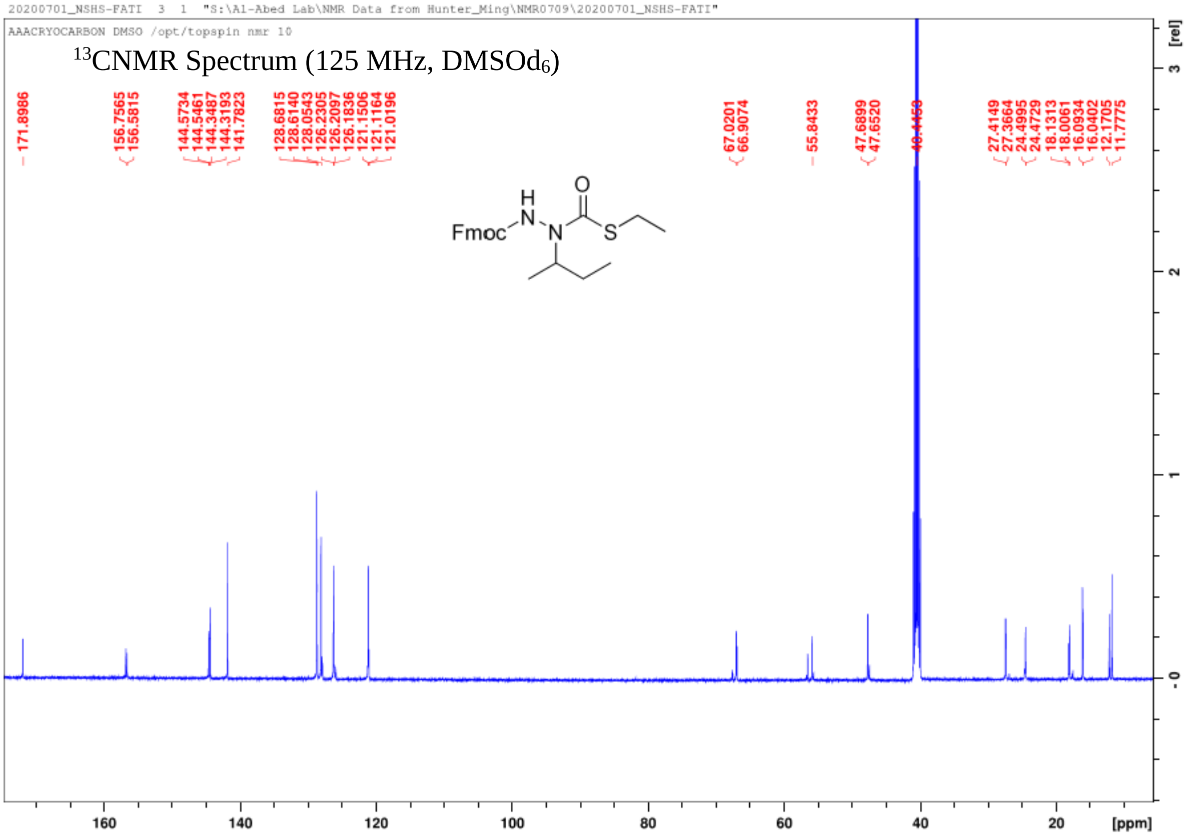

Supplementary Figure 12. NMR spectra of compound 4.

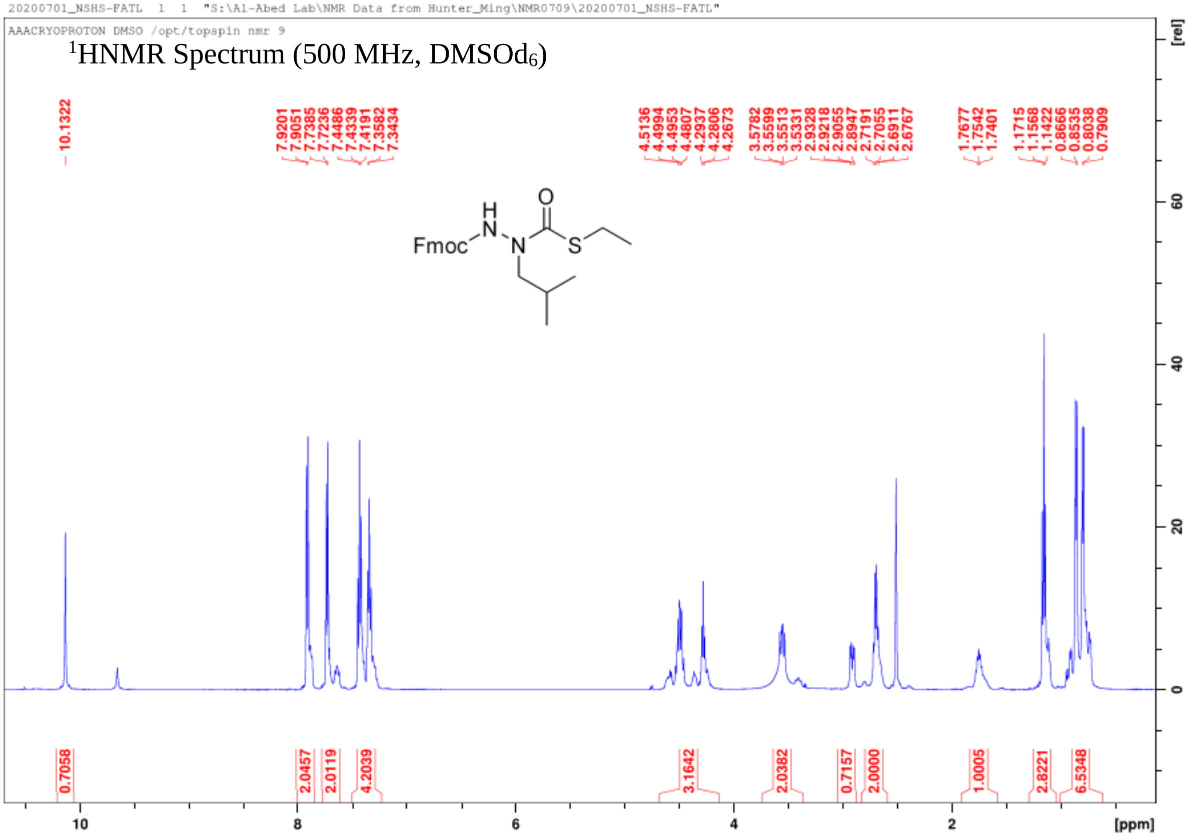

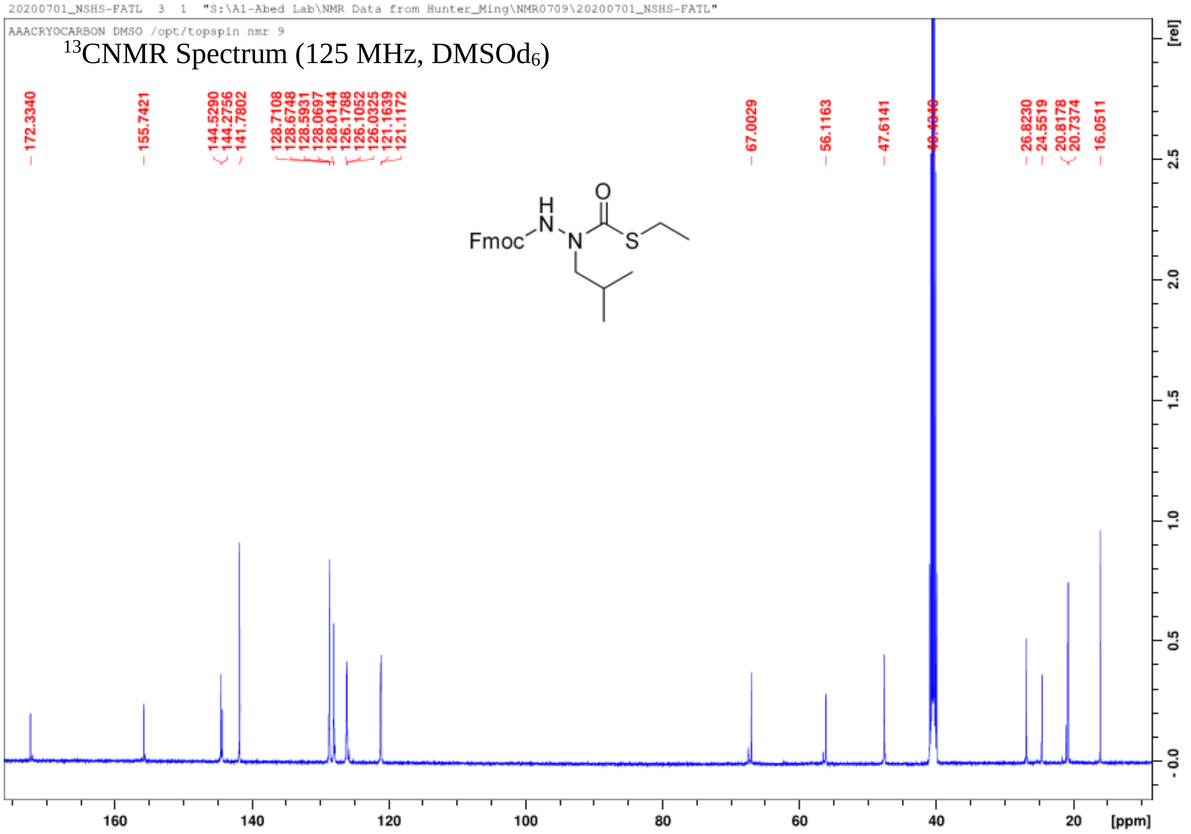

Supplementary Figure 13. NMR spectra of compound 5.

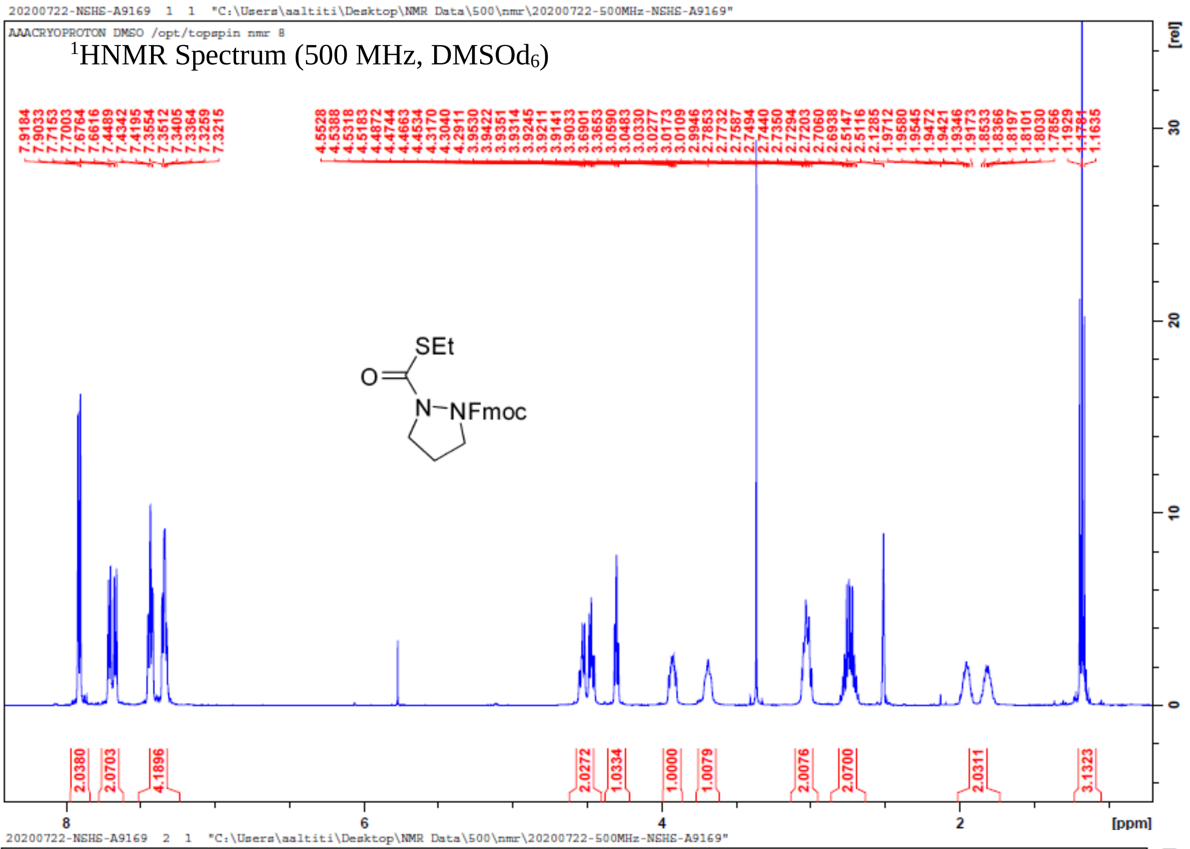

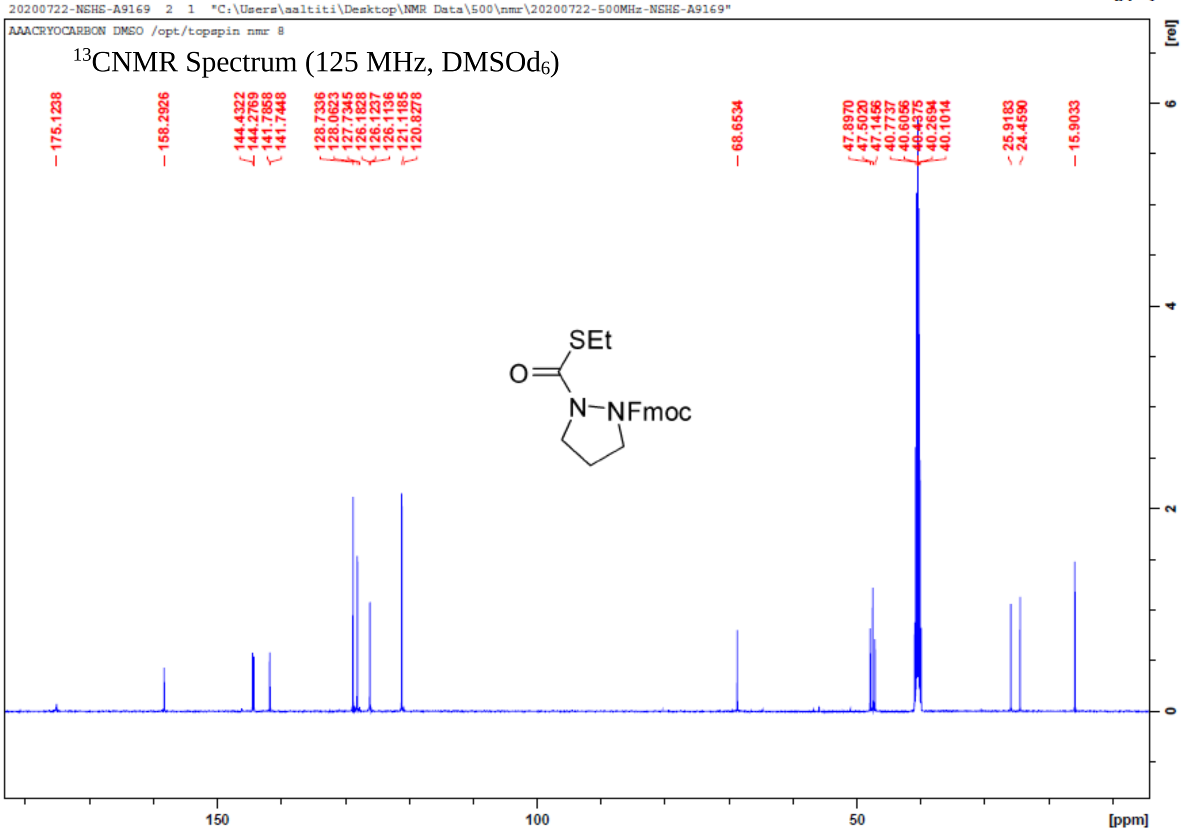

Supplementary Figure 14. NMR spectra of compound 6.

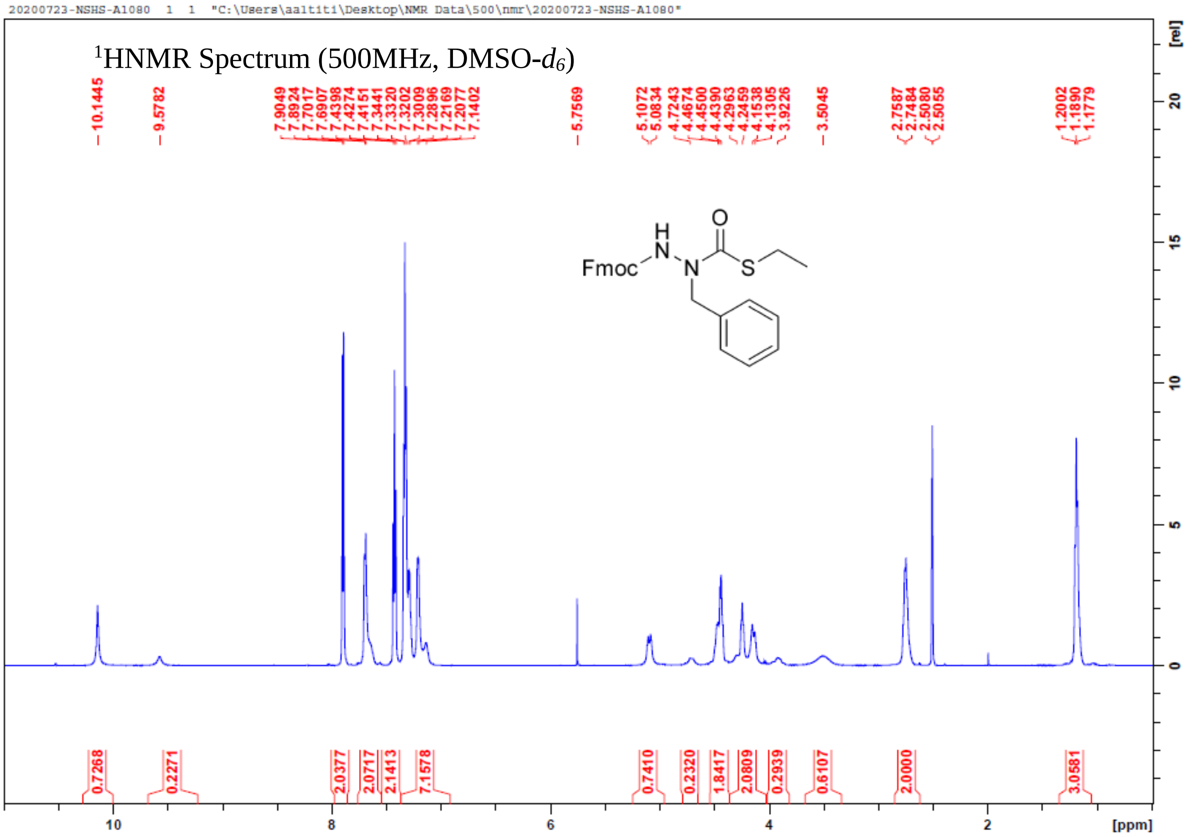

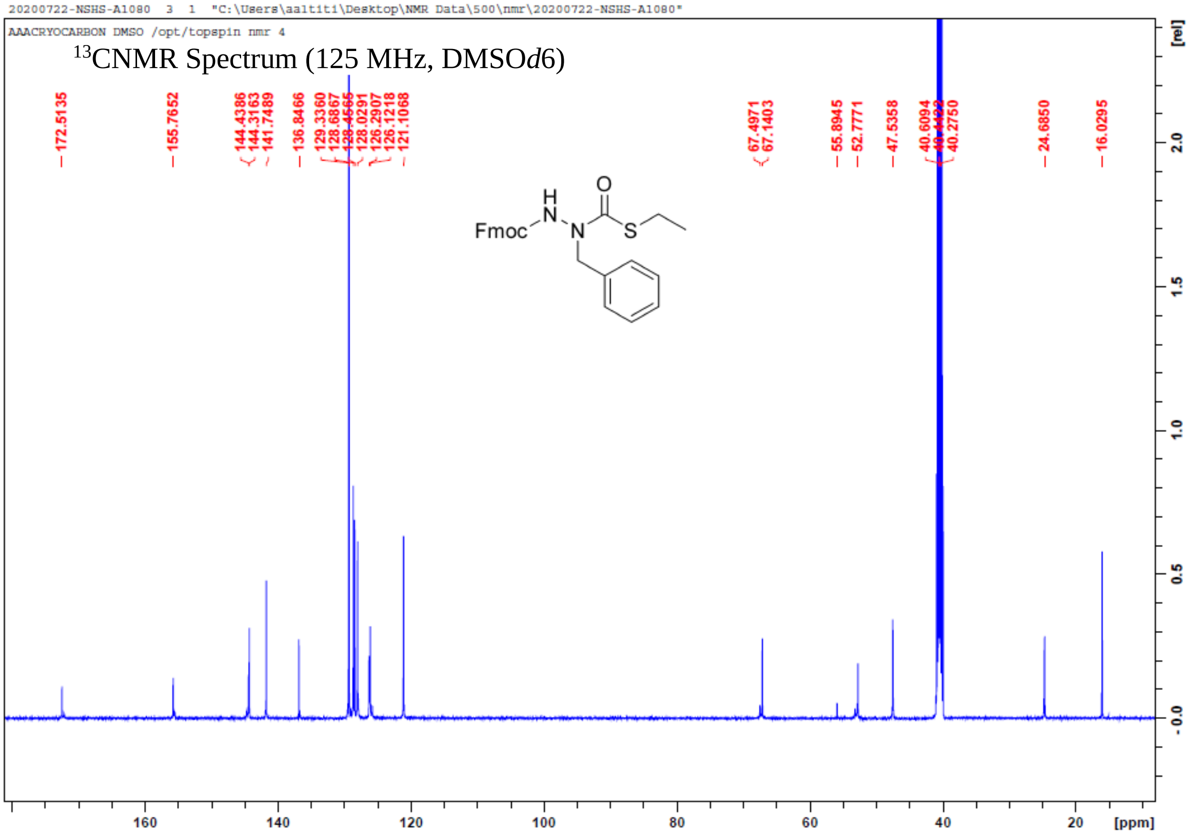

Supplementary Figure 15. NMR spectra of compound 7.

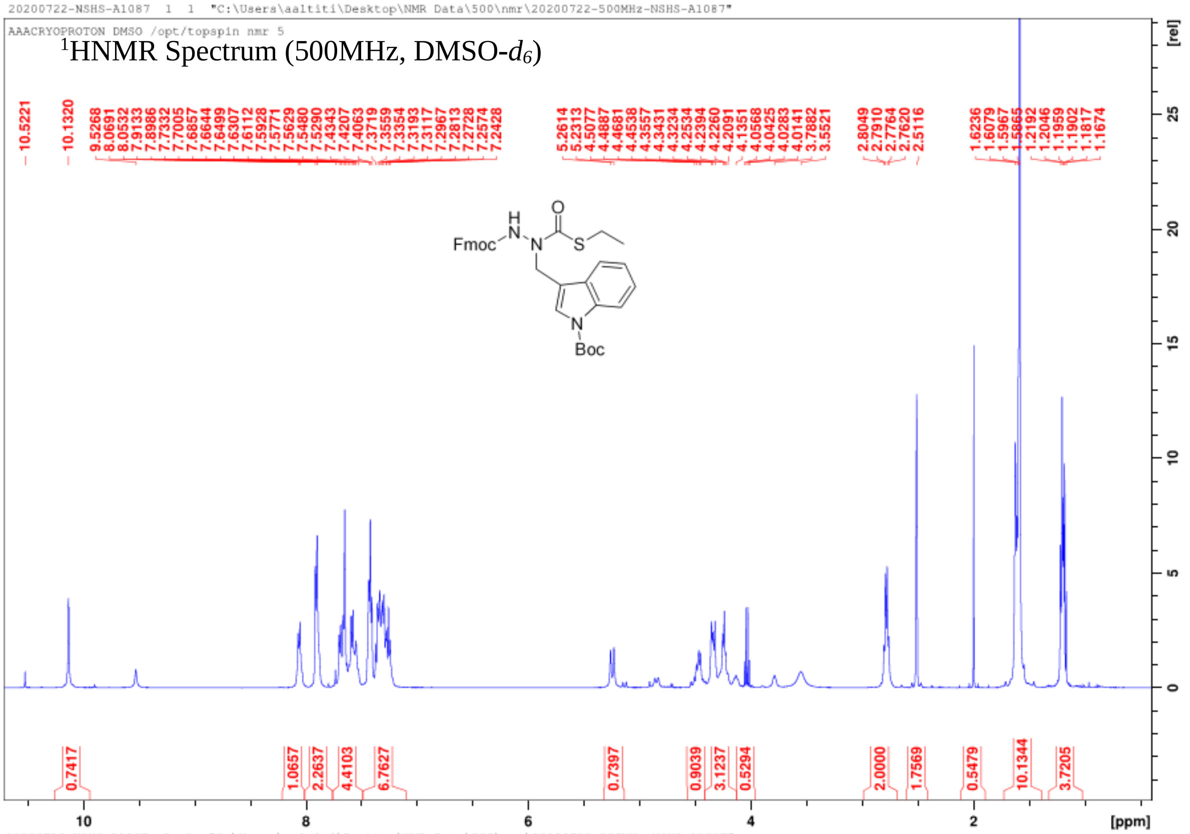

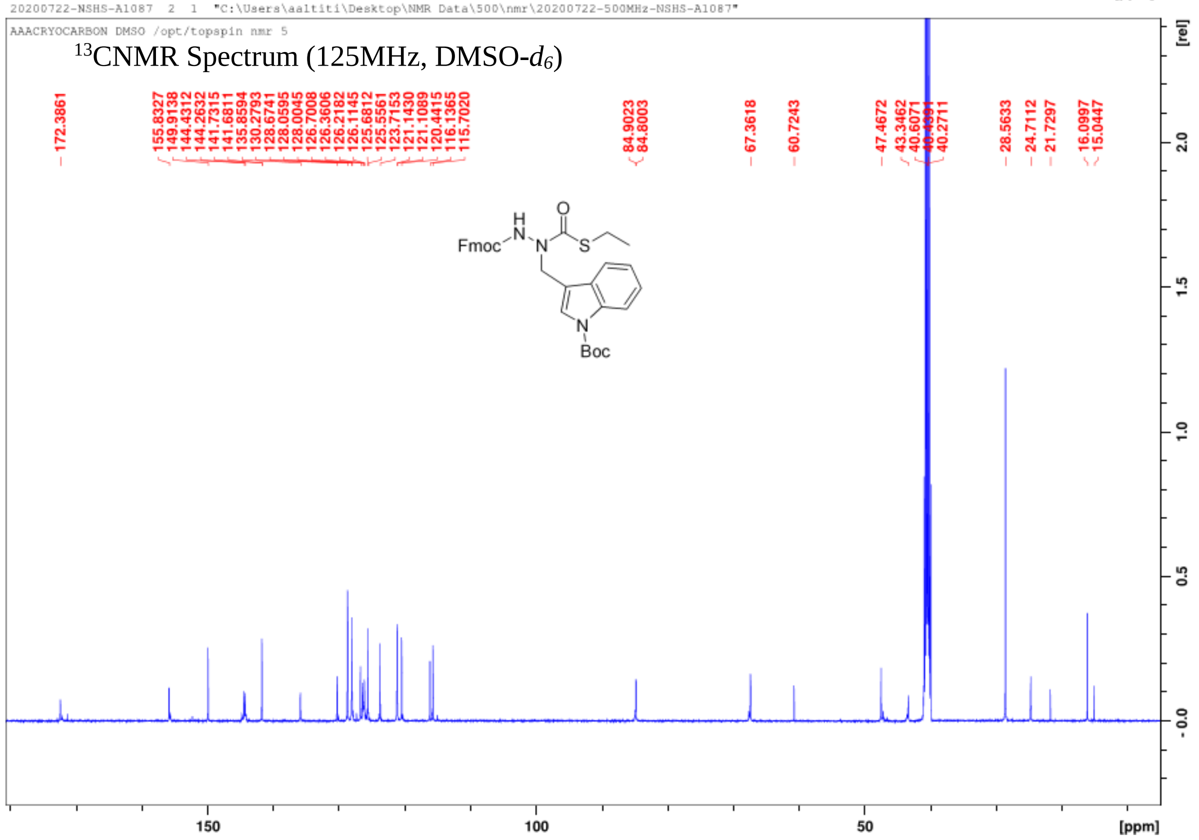

Supplementary Figure 16. NMR spectra of compound 8a.

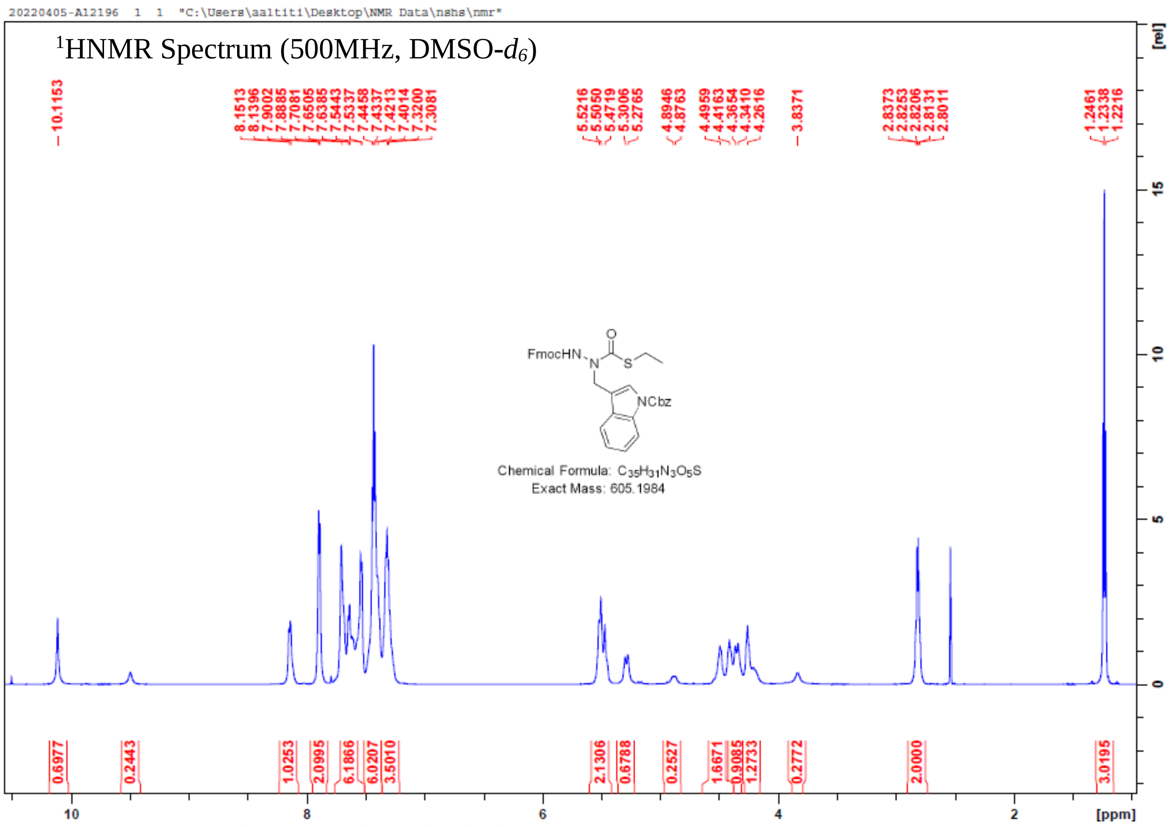

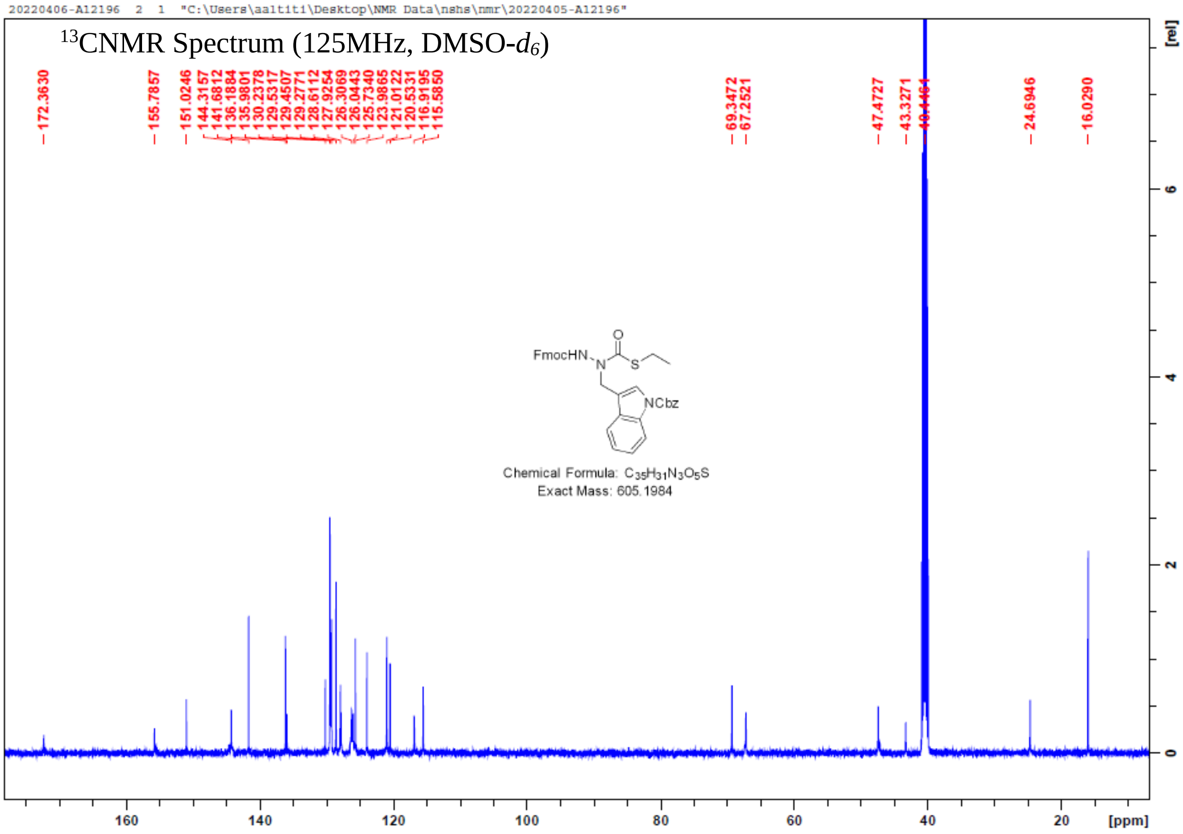

Supplementary Figure 17. NMR spectra of compound 8b.

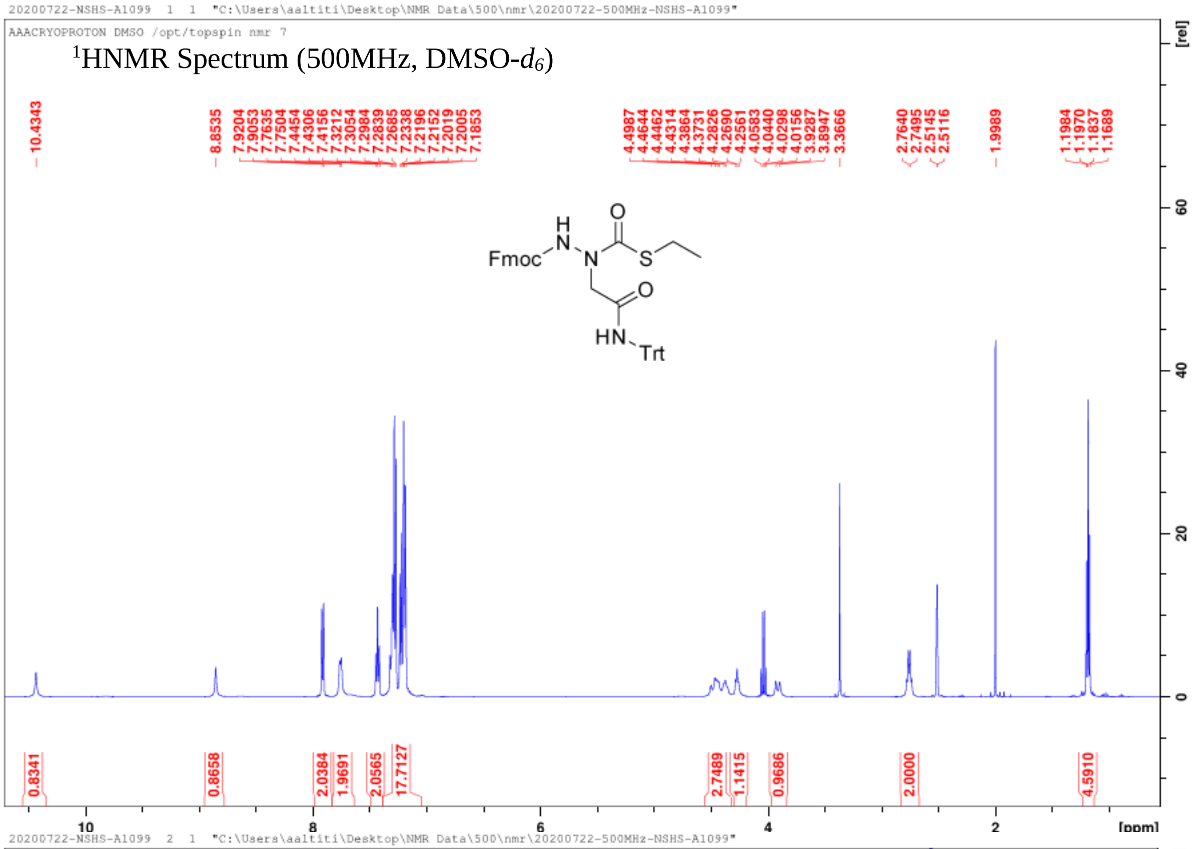

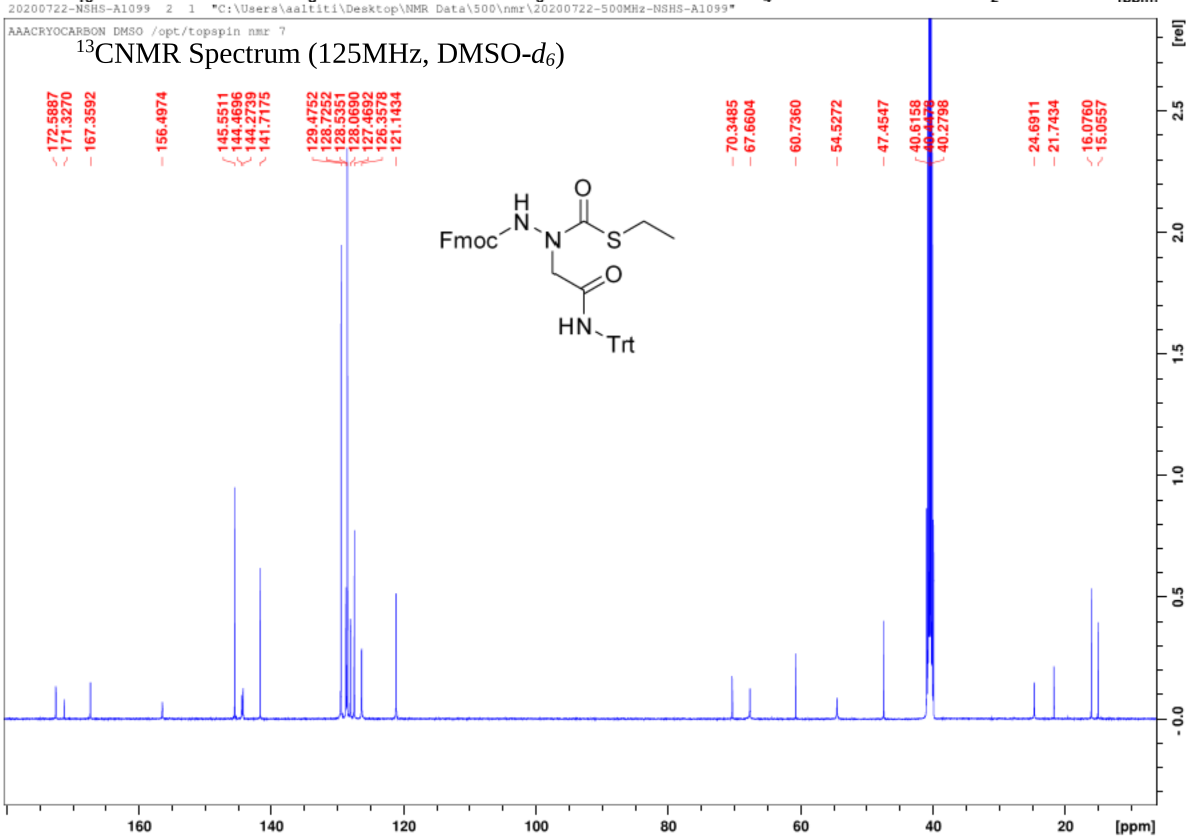

Supplementary Figure 18. NMR spectra of compound 9.

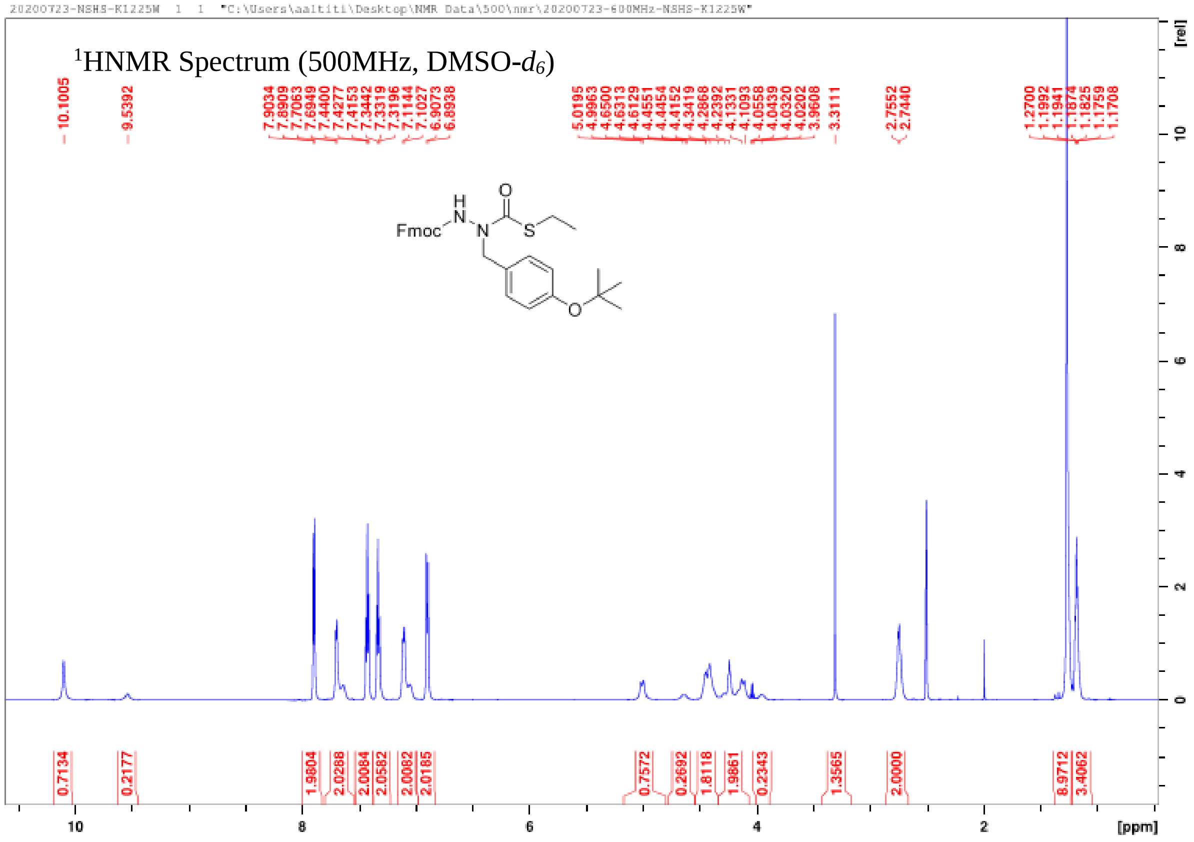

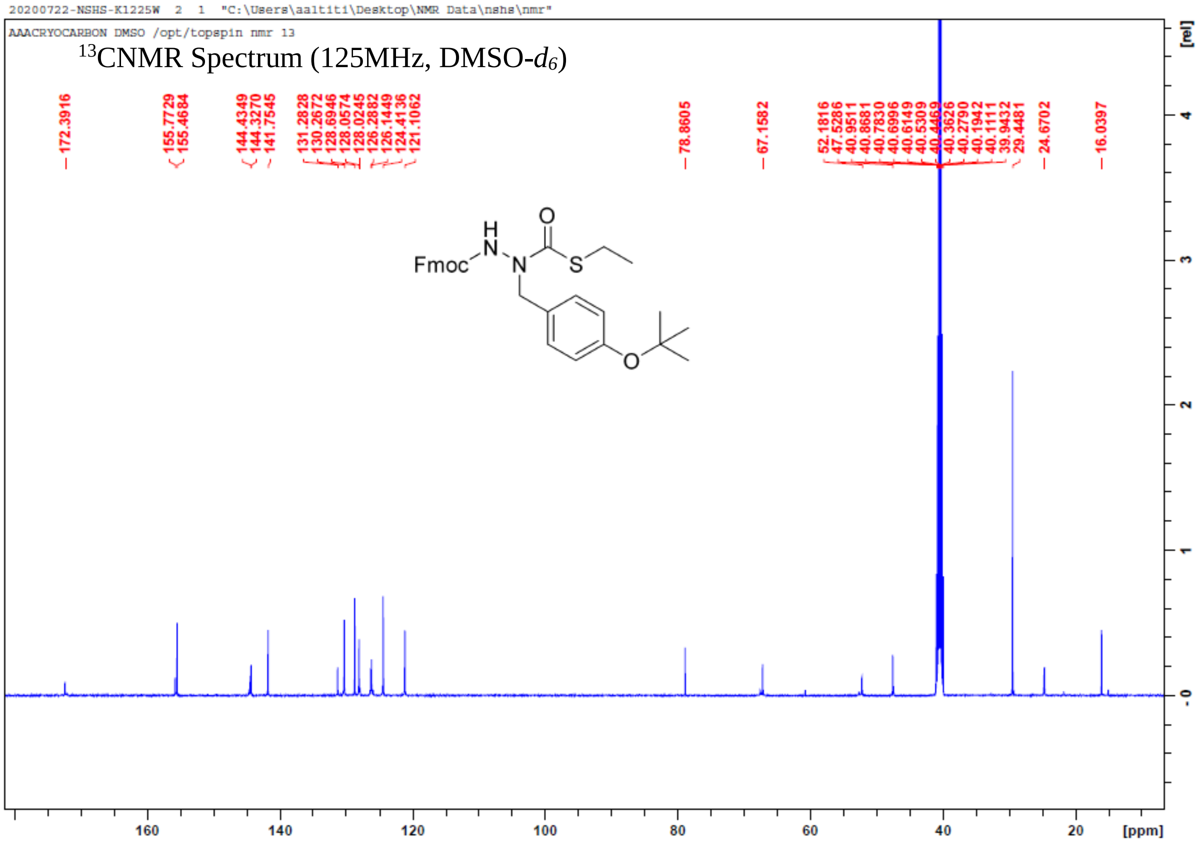

Supplementary Figure 19. NMR spectra of compound 10.

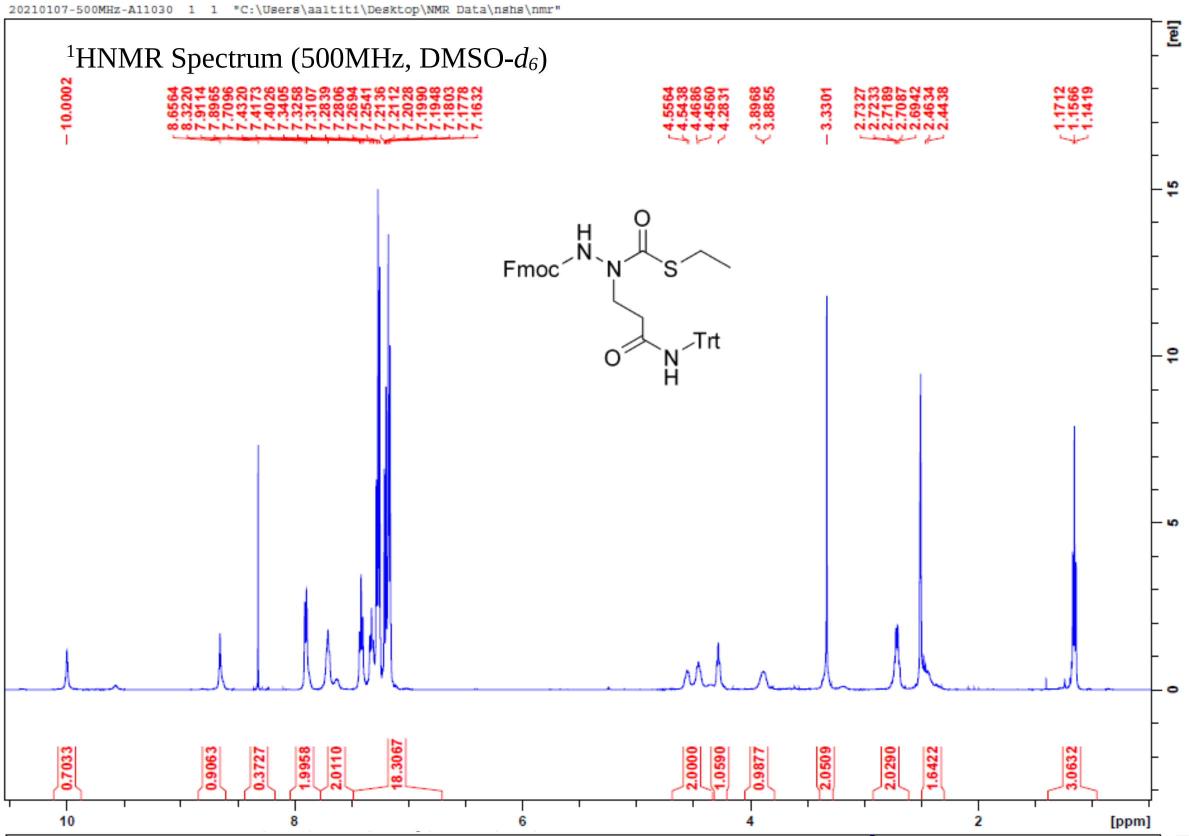

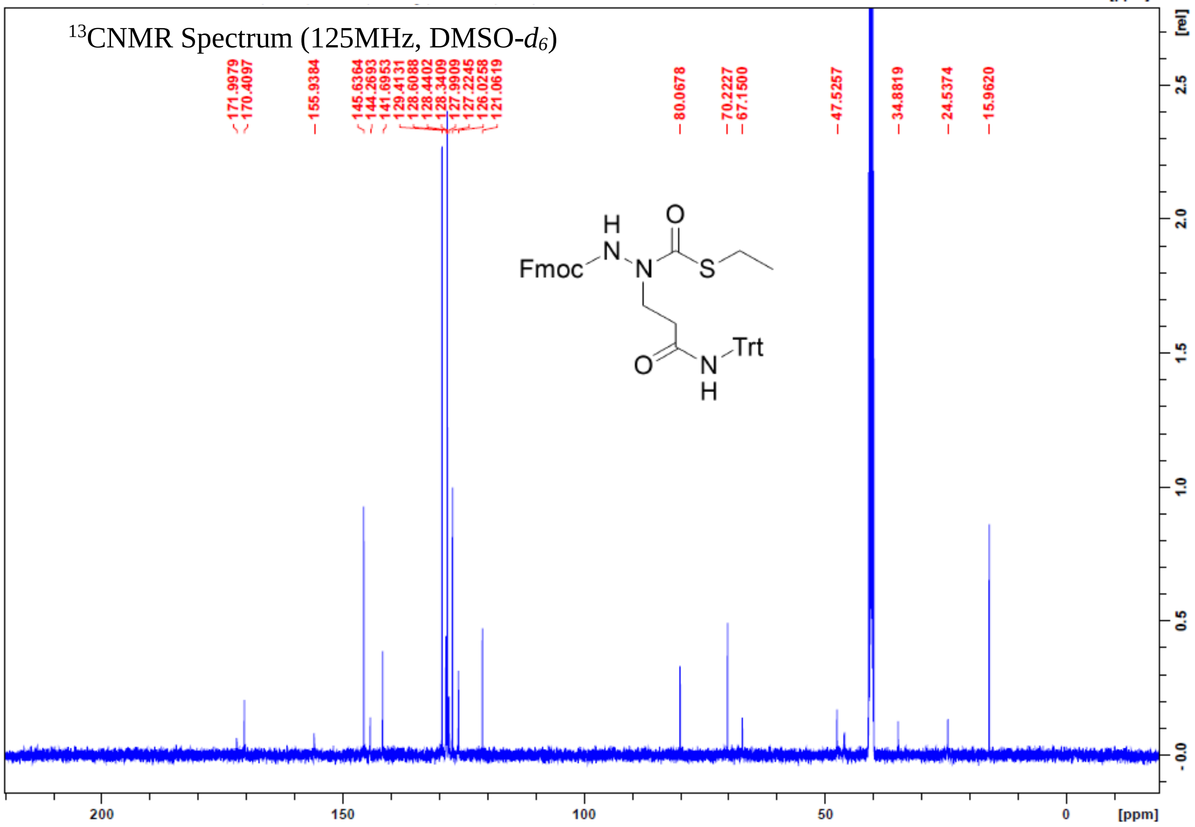

Supplementary Figure 20. NMR spectra of compound 11.

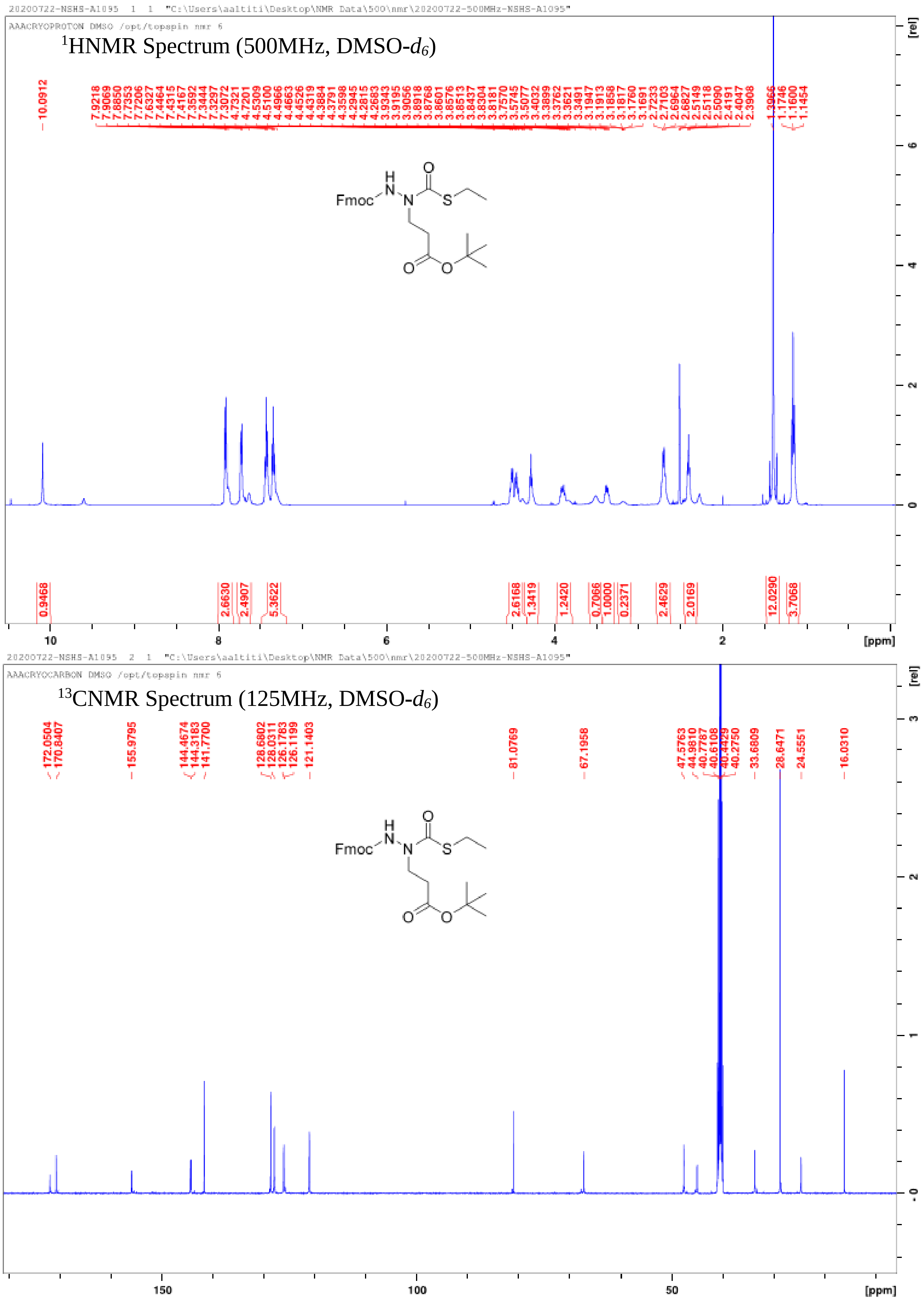

Supplementary Figure 21. NMR spectra of compound 12.

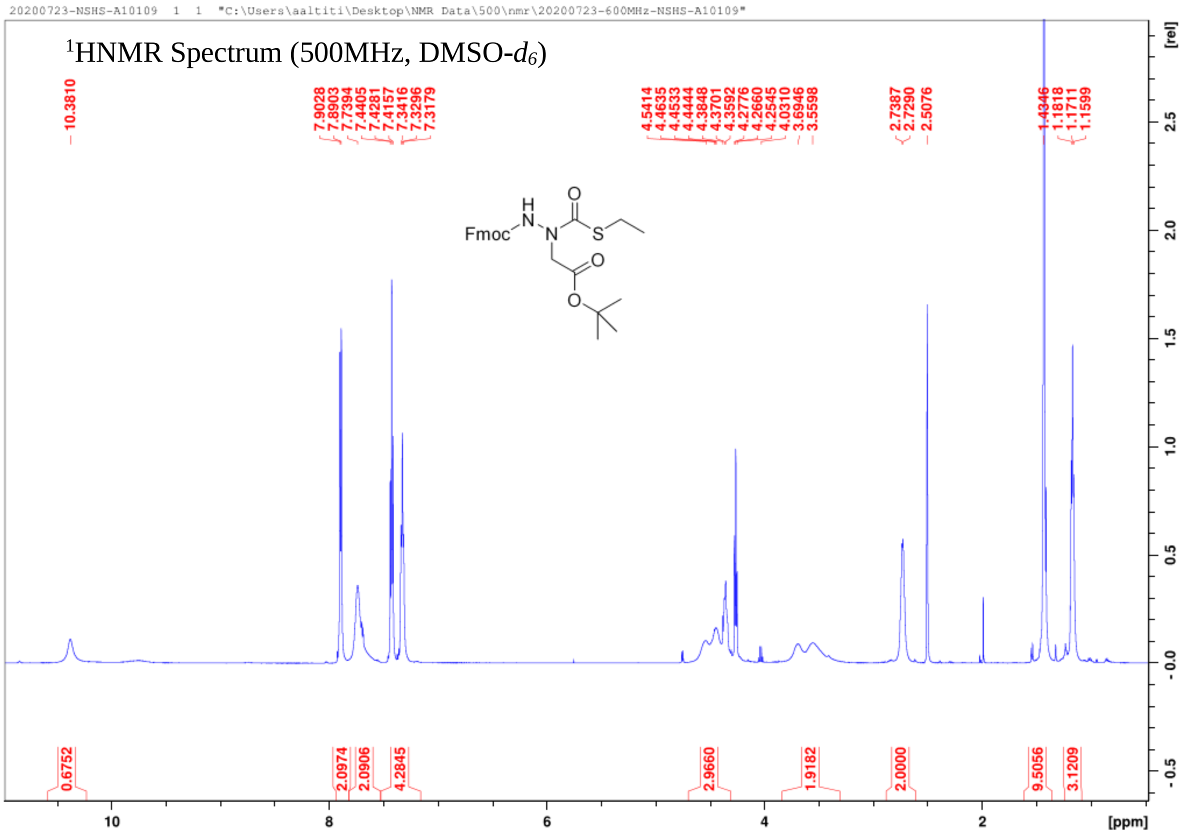

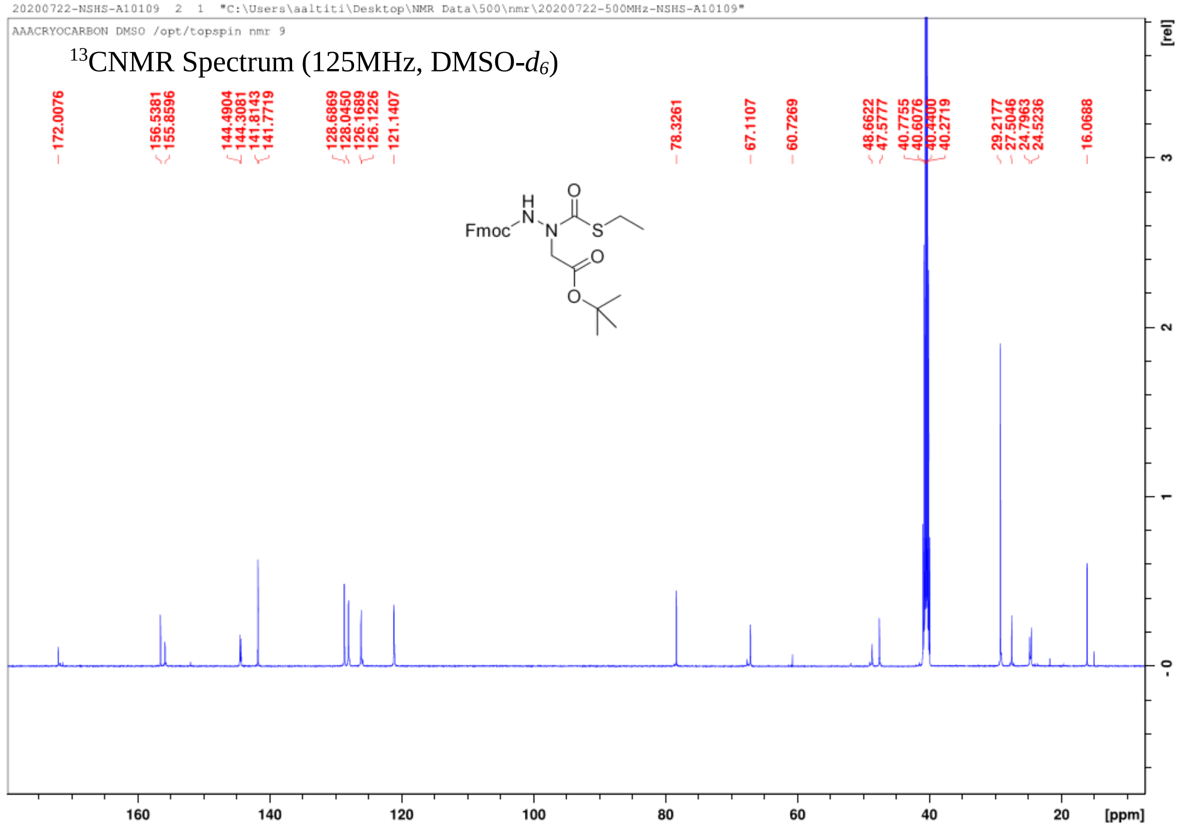

Supplementary Figure 22. NMR spectra of compound 13.

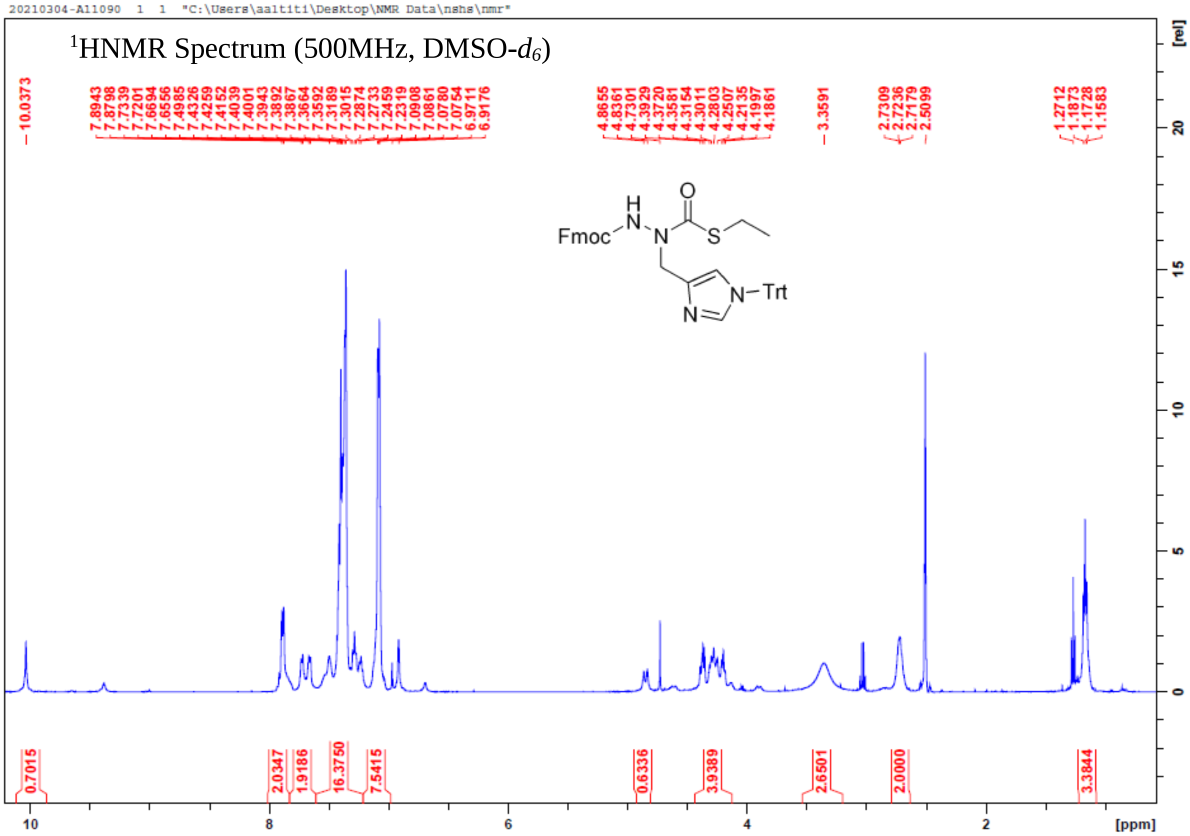

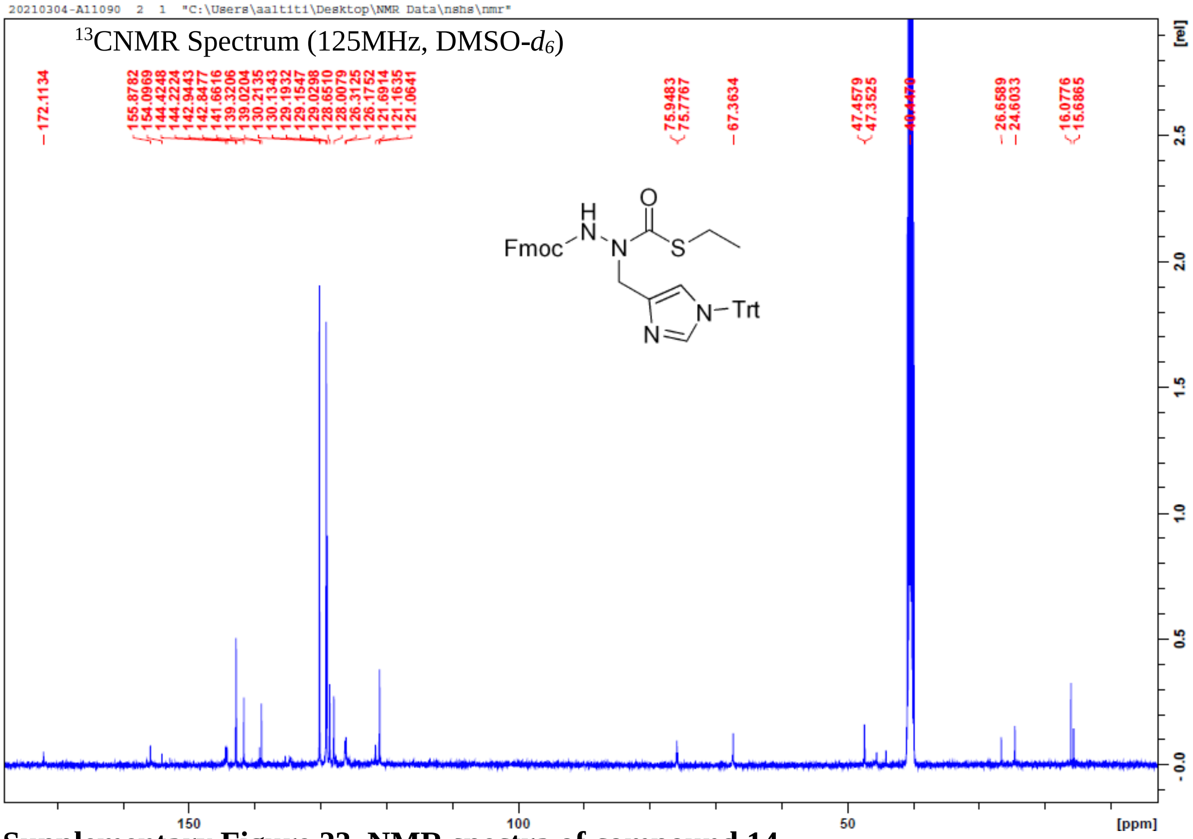

Supplementary Figure 23. NMR spectra of compound 14.

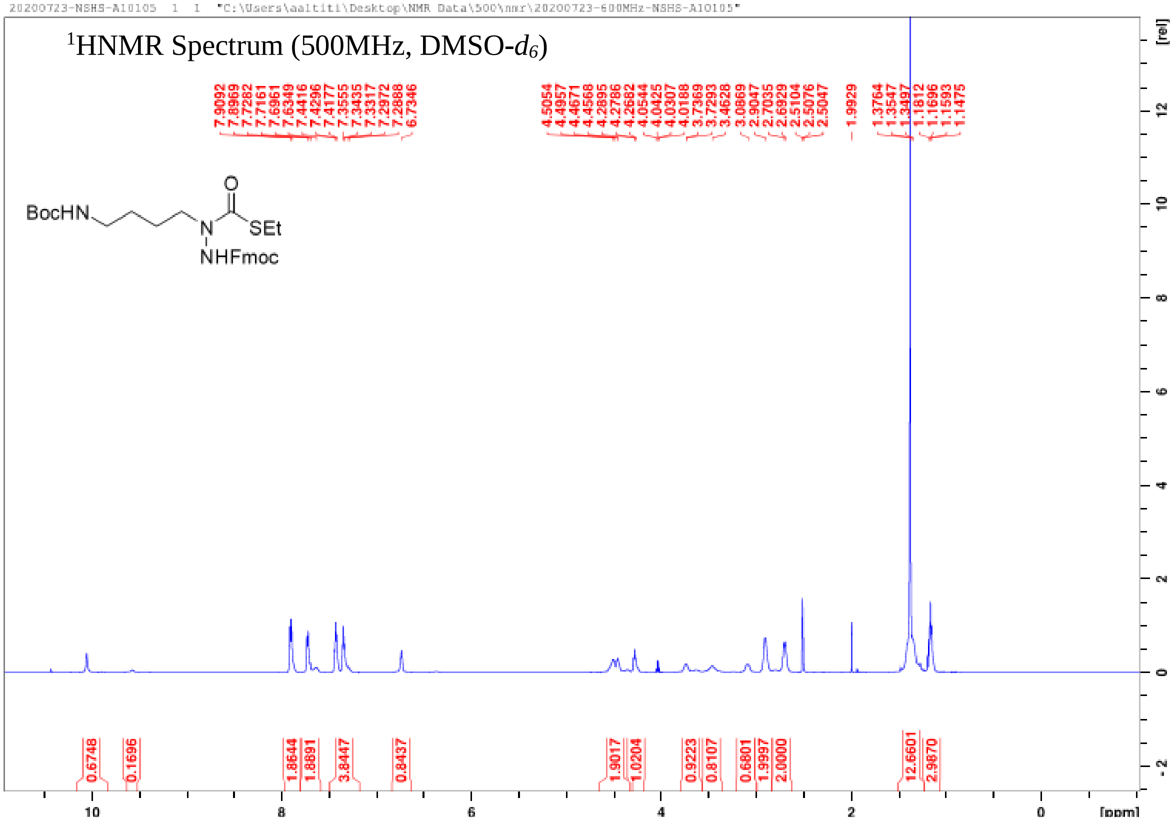

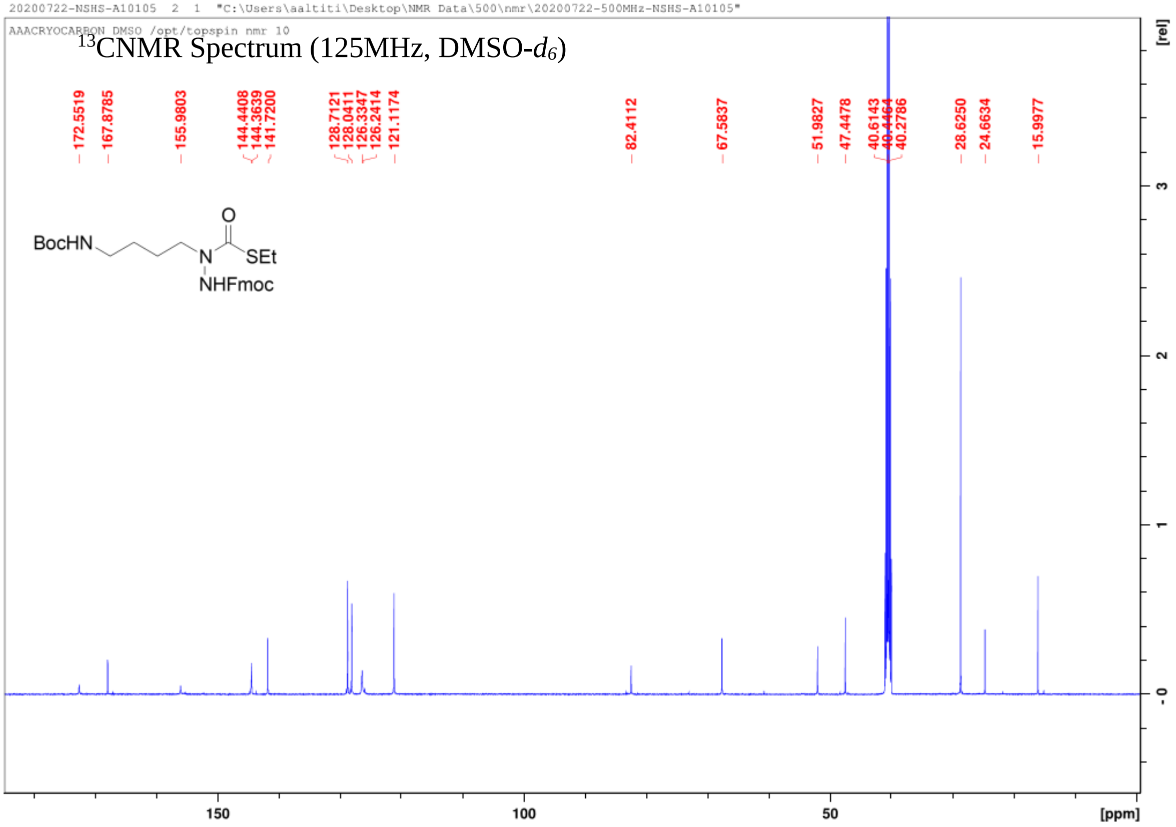

Supplementary Figure 24. NMR spectra of compound 15.

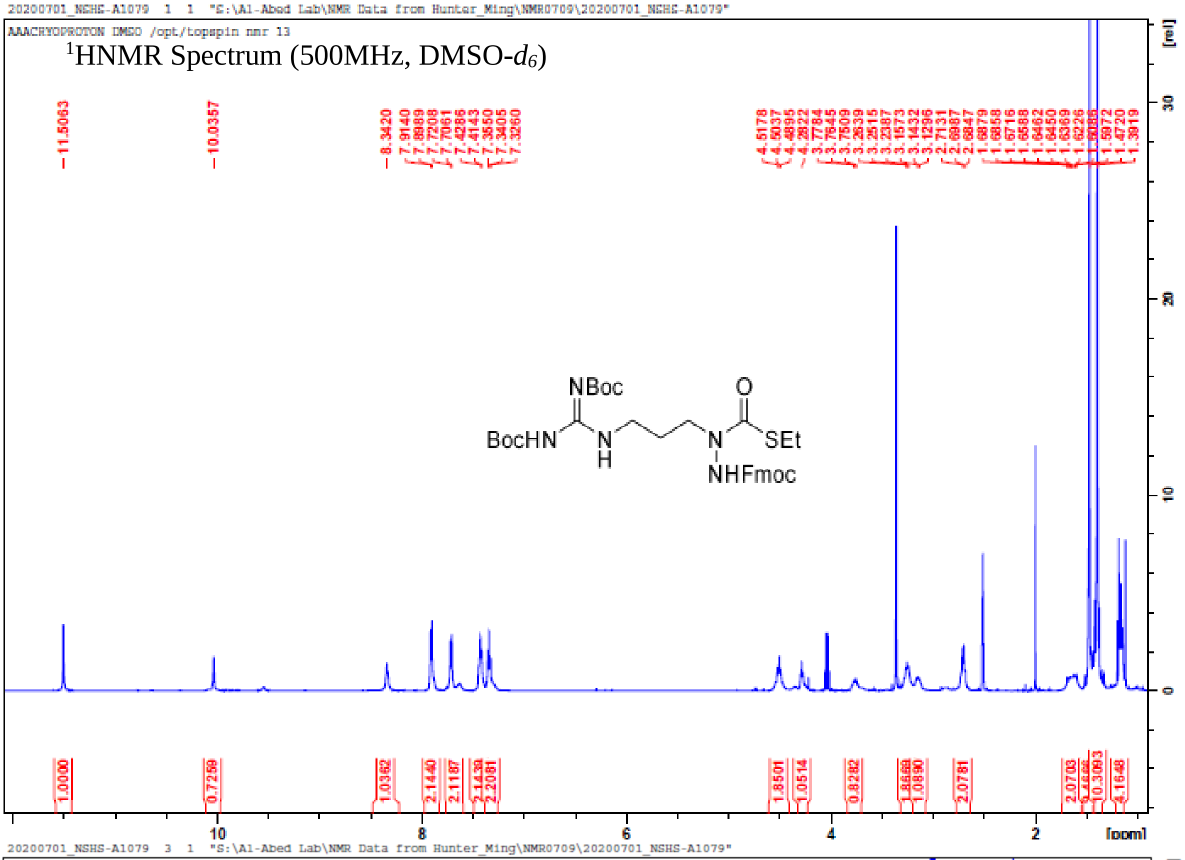

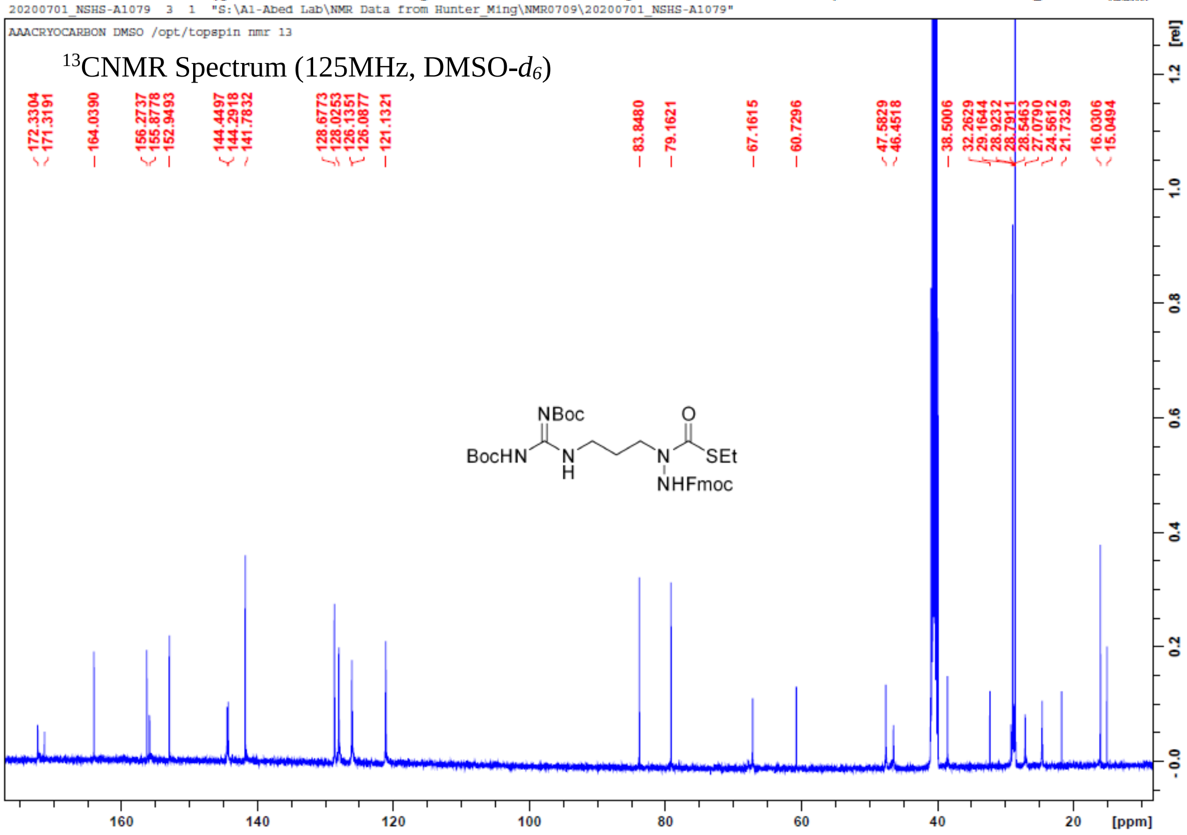

Supplementary Figure 25. NMR spectra of compound 16.

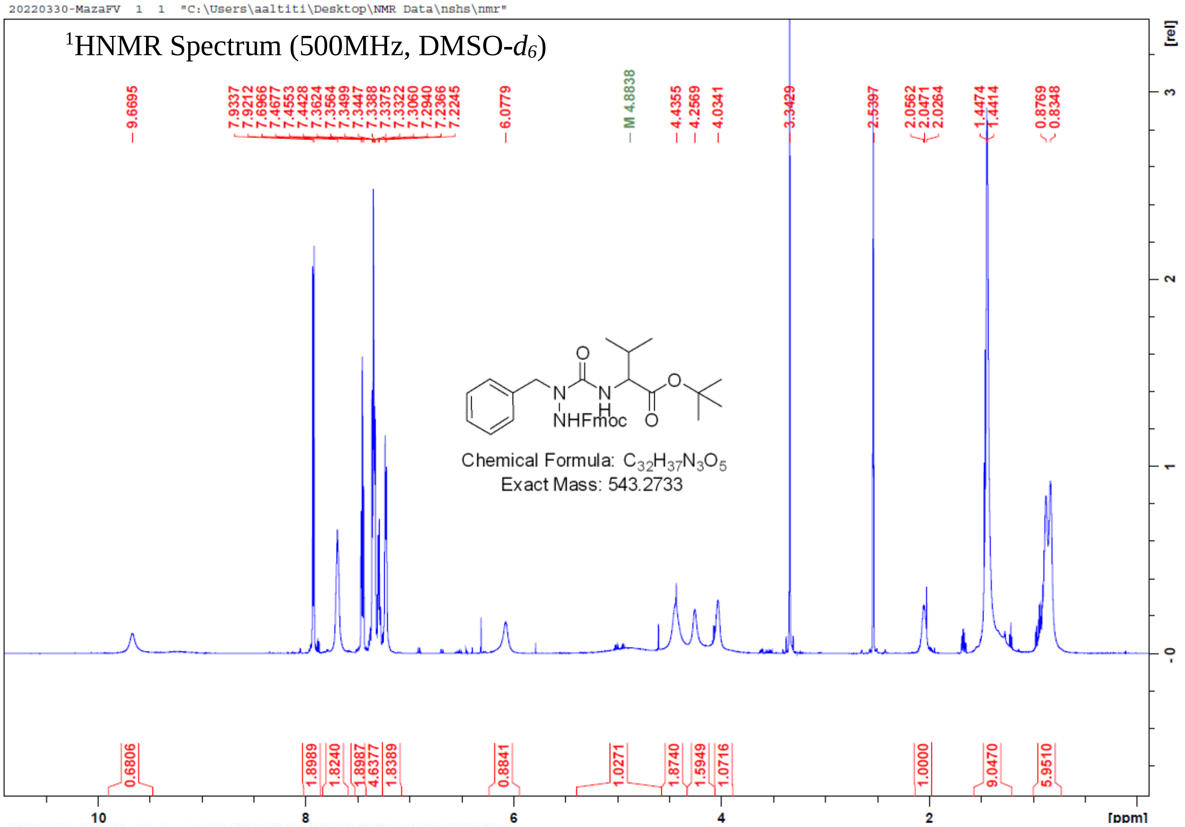

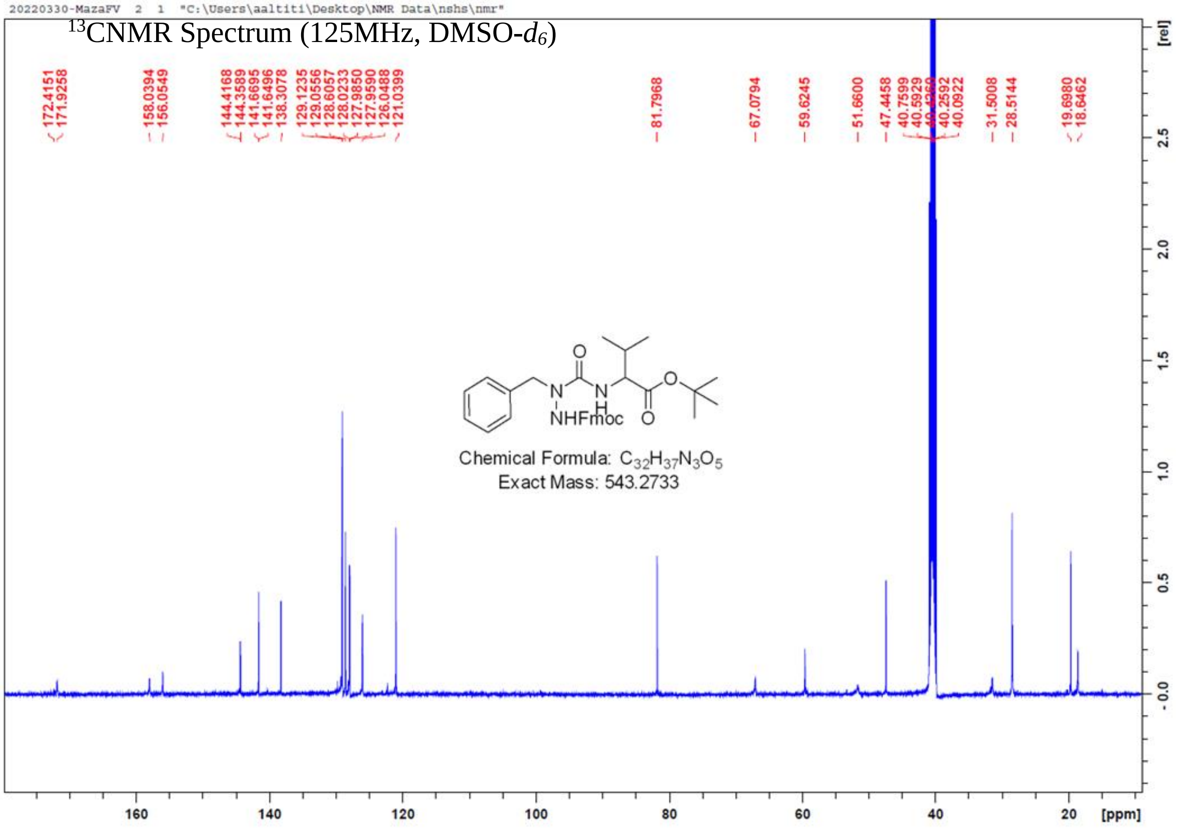

Supplementary Figure 26. NMR spectra of compound 18.

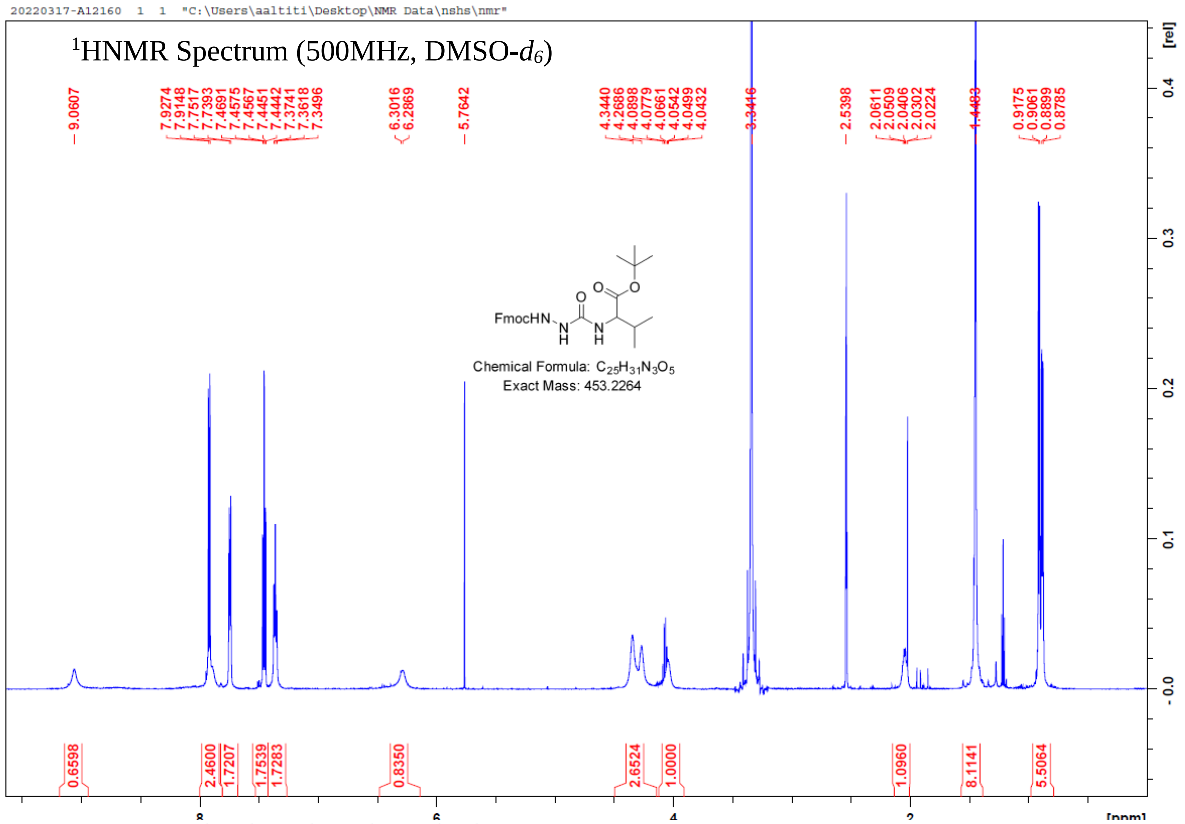

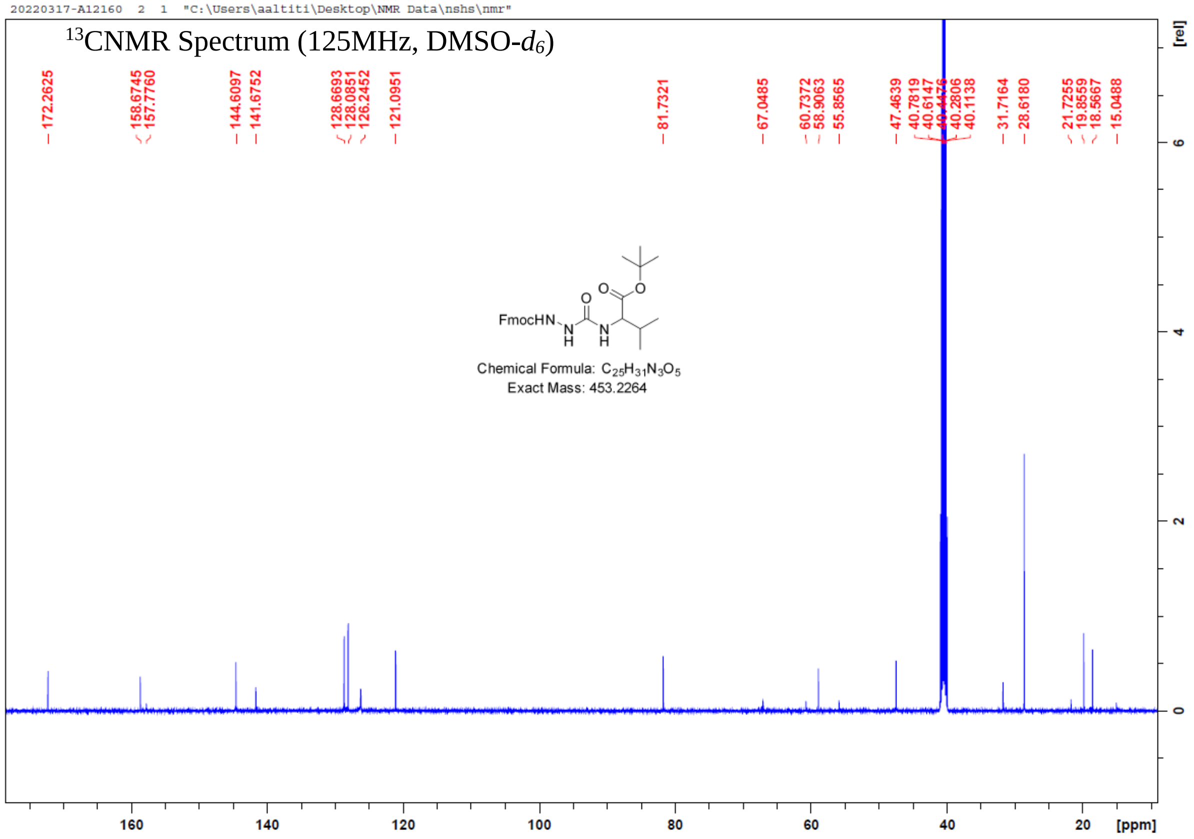

Supplementary Figure 27. NMR spectra of compound 19.

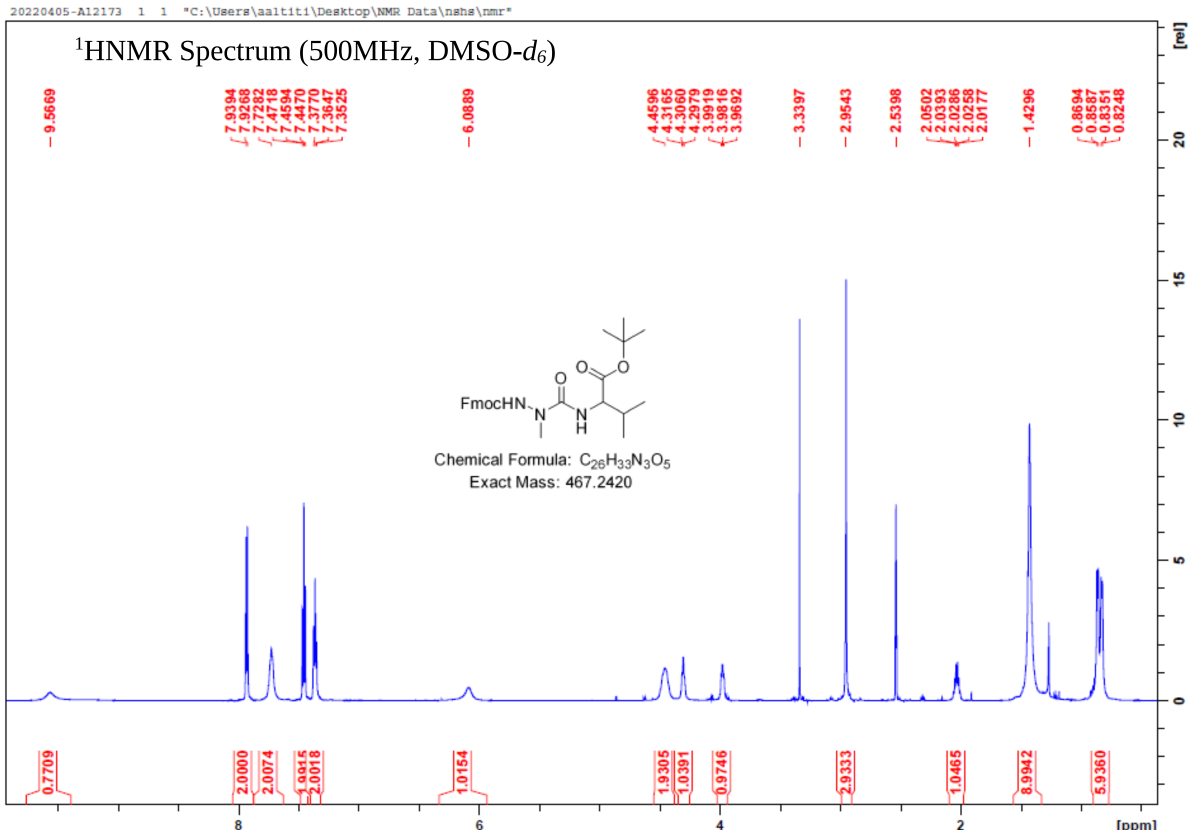

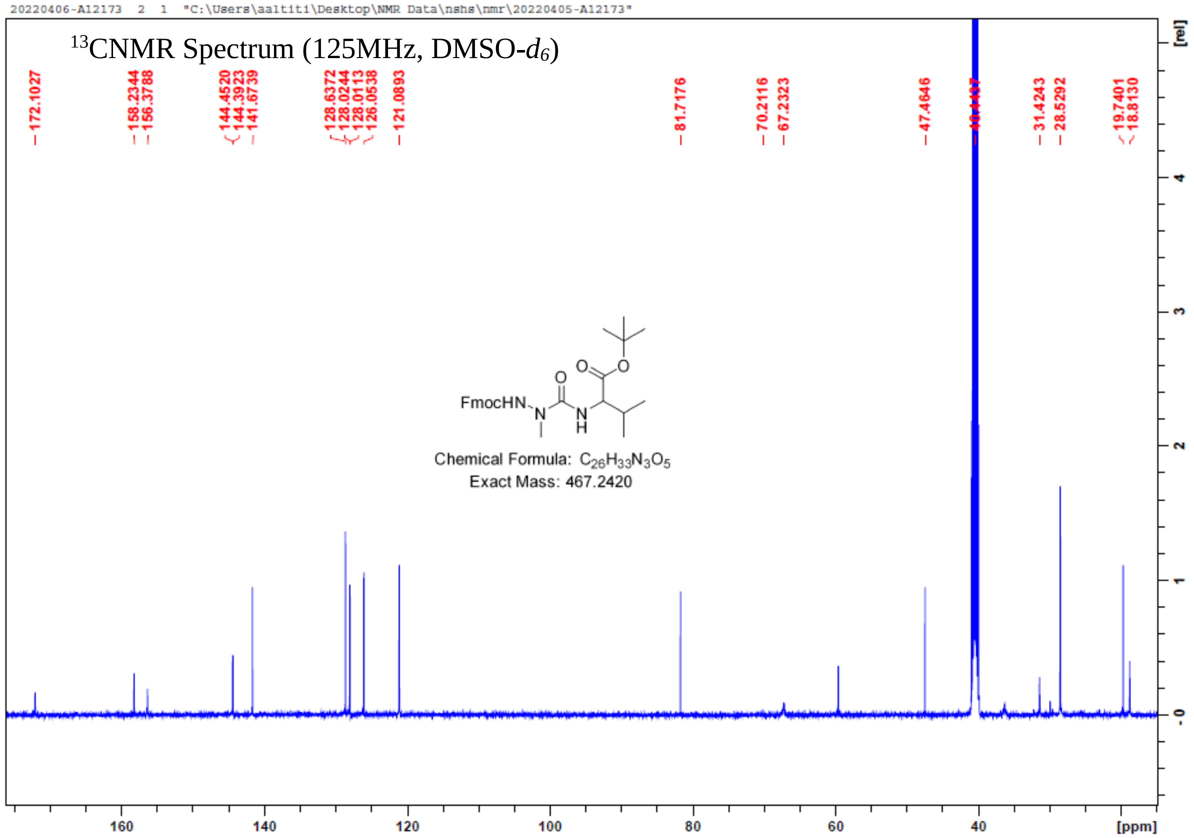

Supplementary Figure 28. NMR spectra of compound 20.

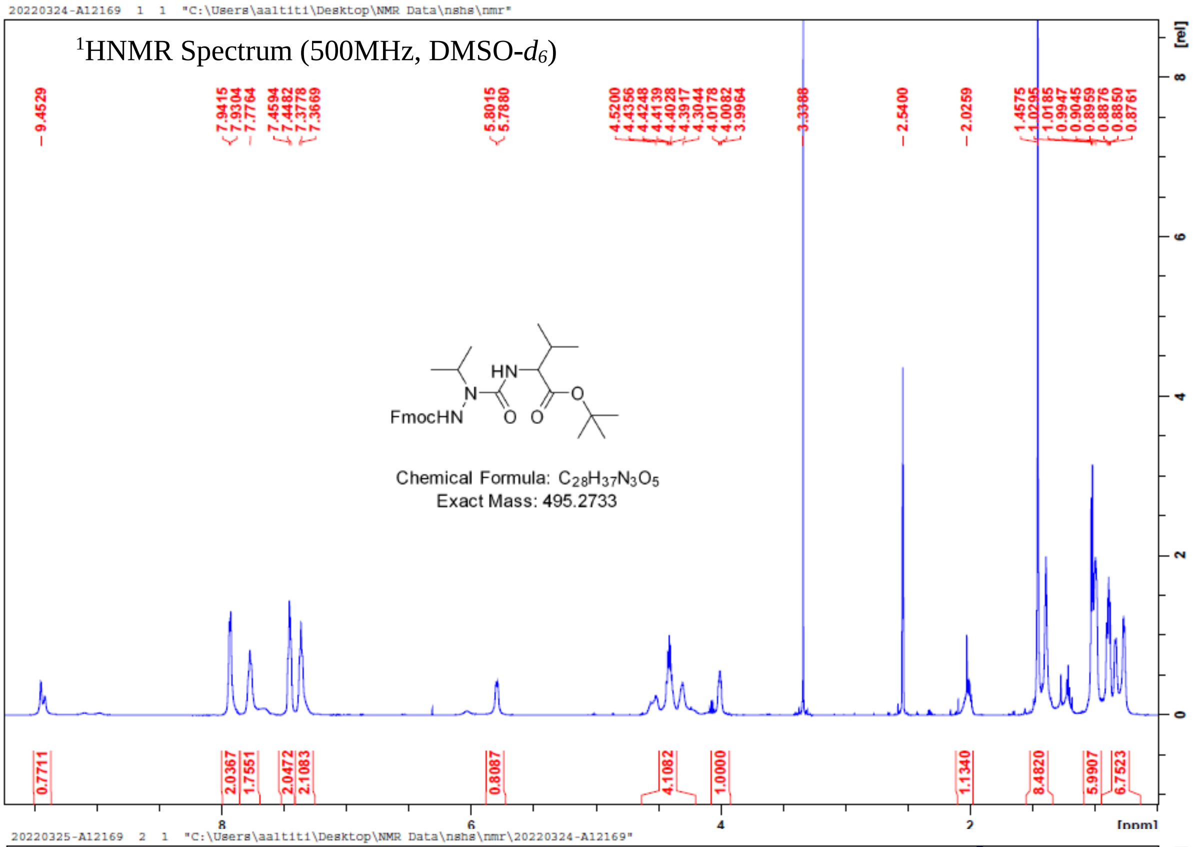

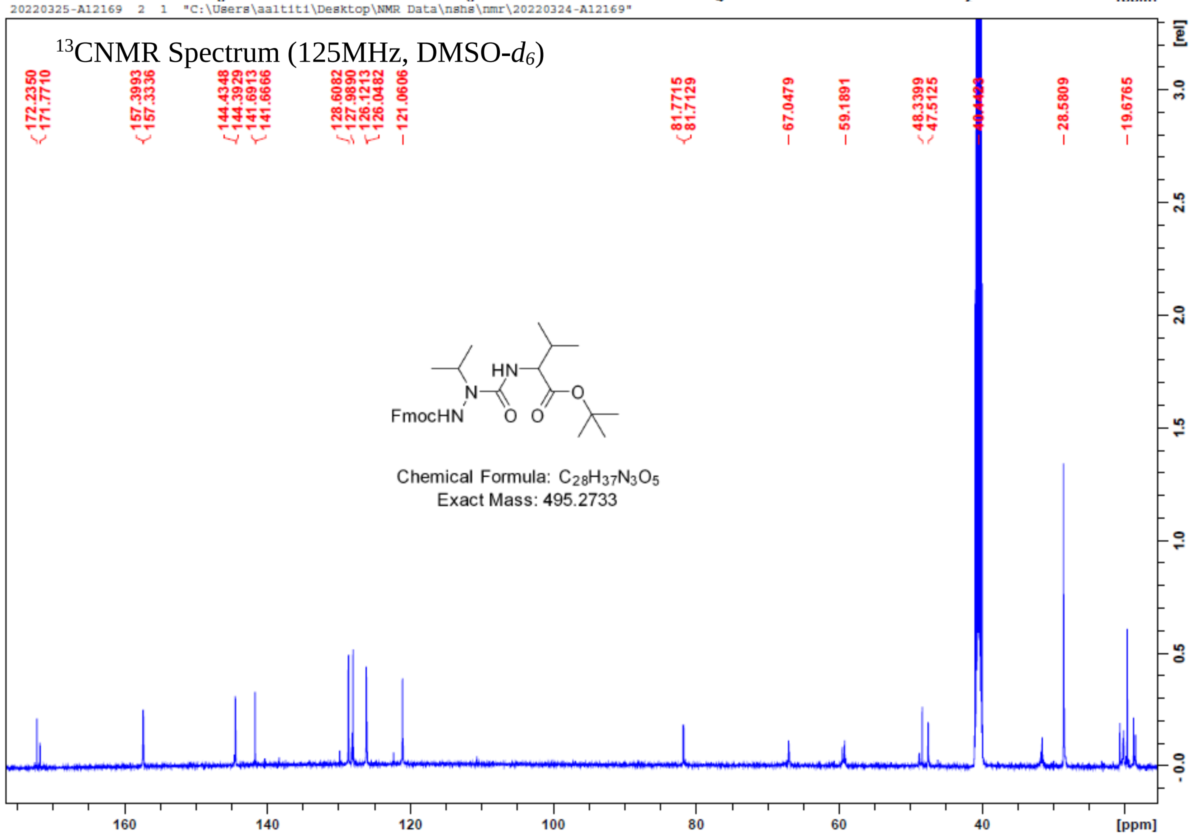

Supplementary Figure 29. NMR spectra of compound 21.

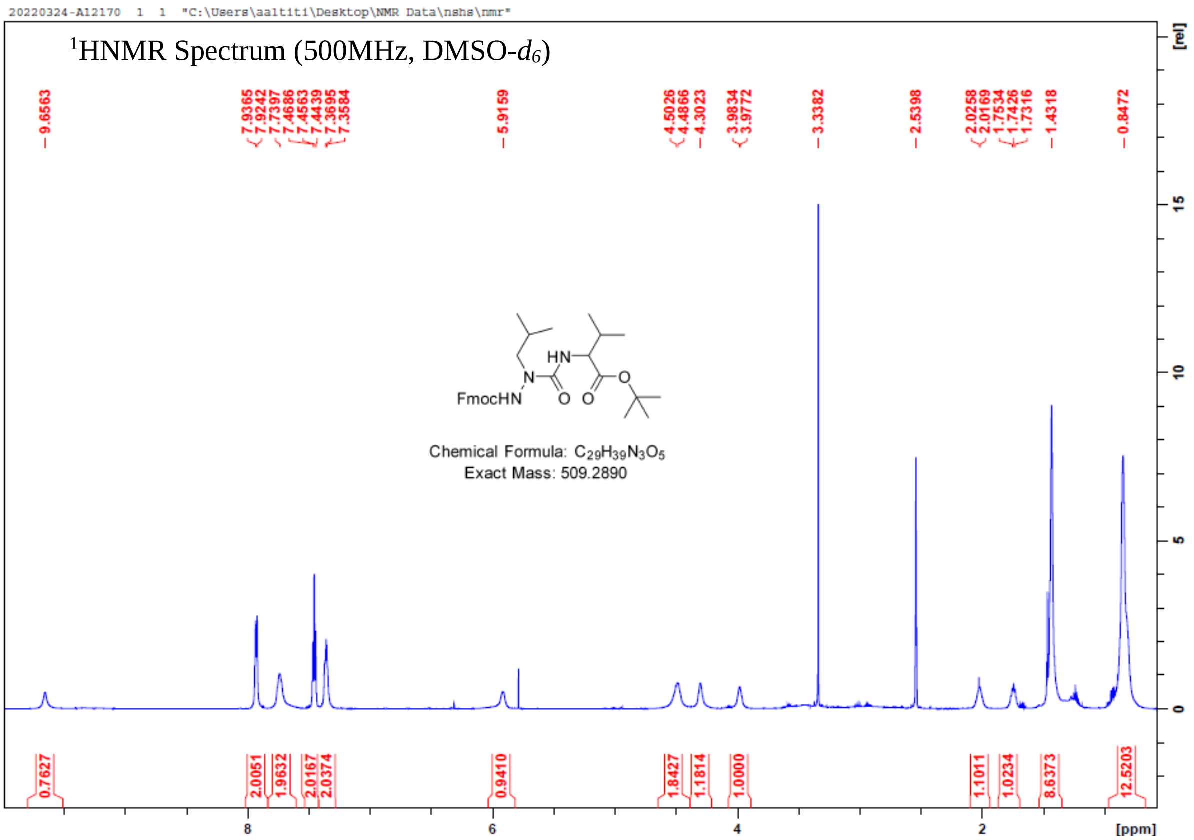

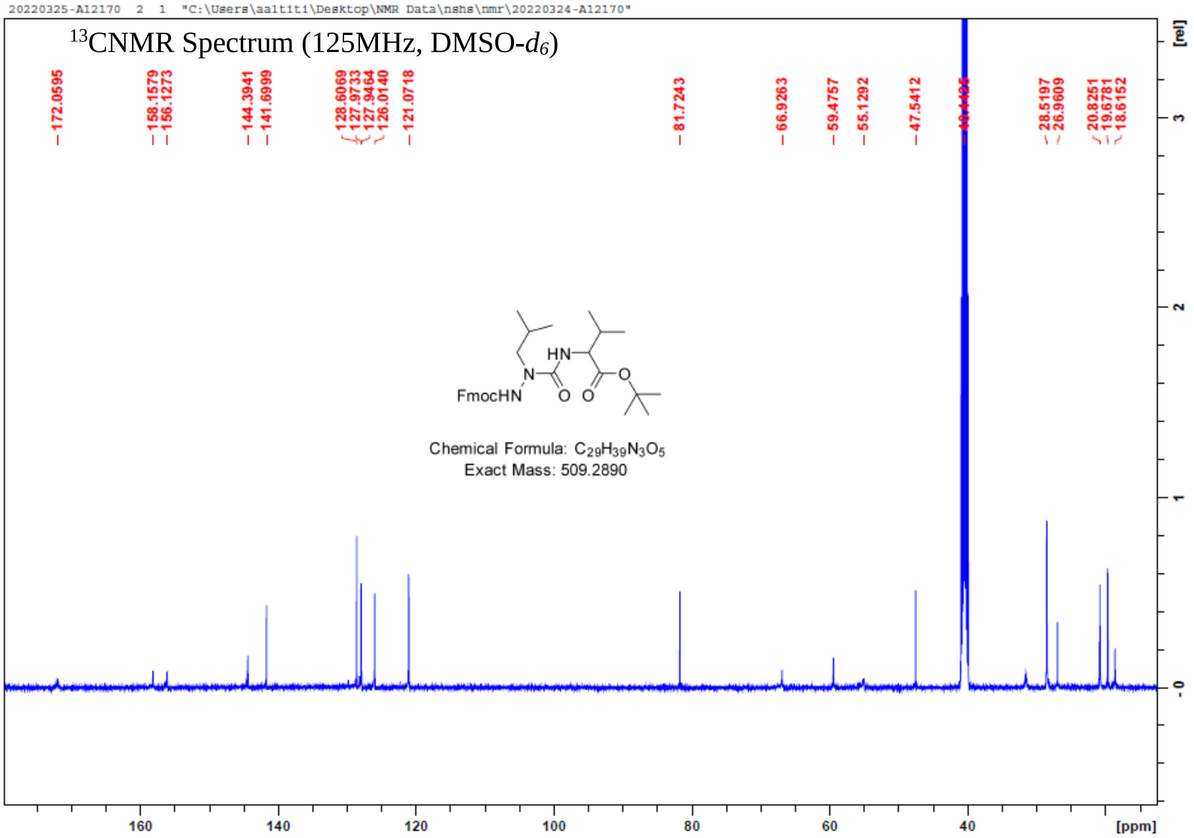

Supplementary Figure 30. NMR spectra of compound 22.

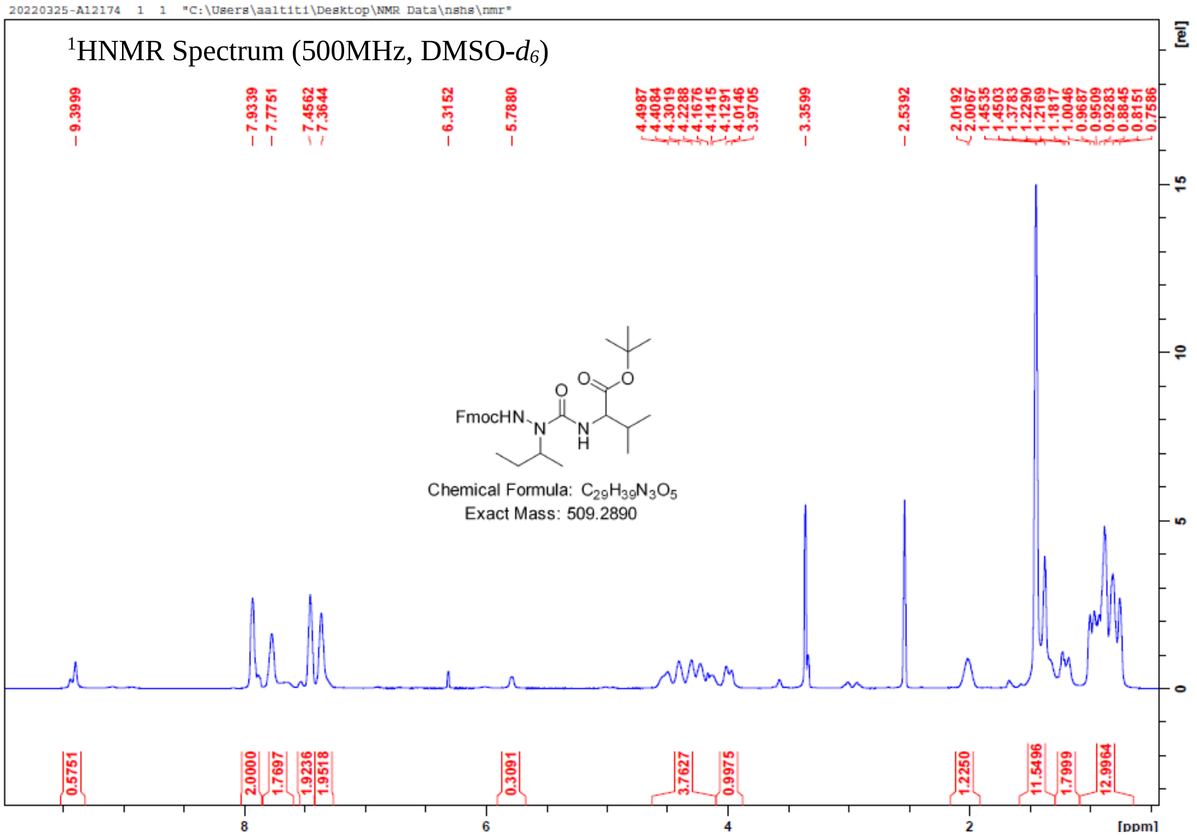

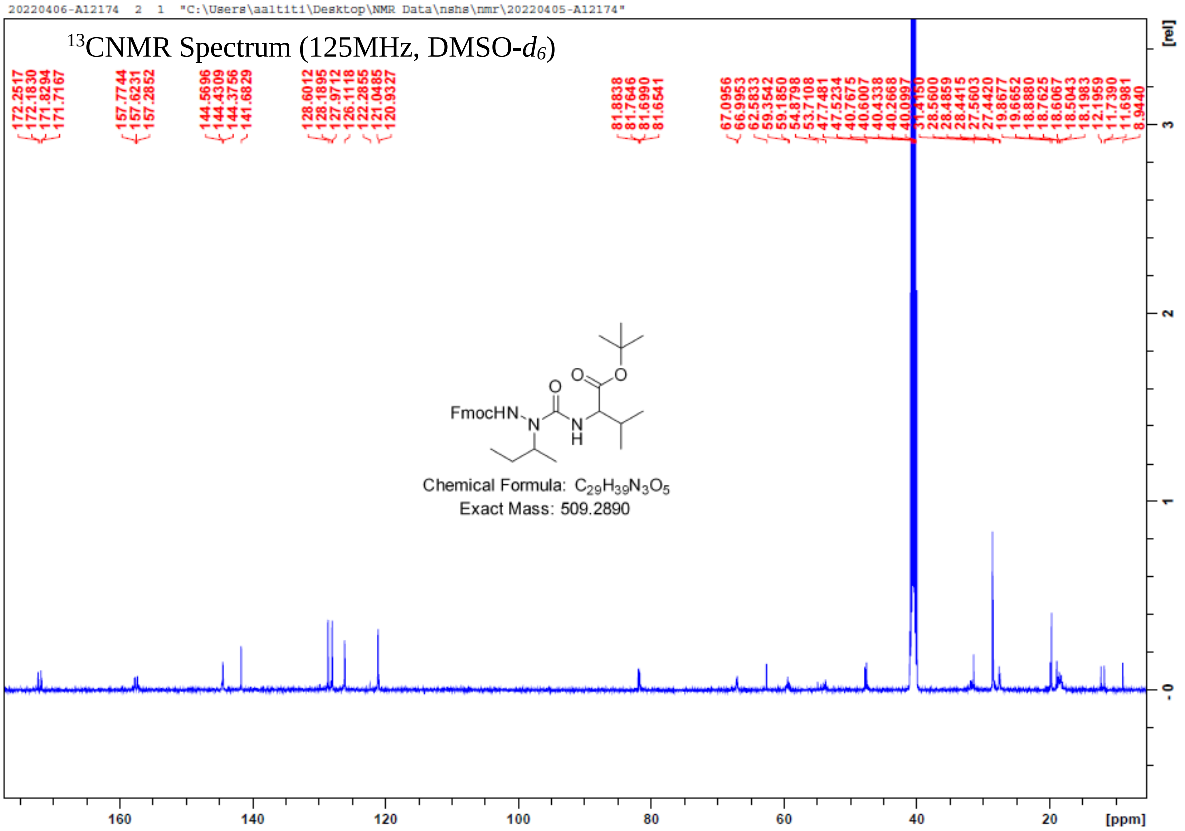

Supplementary Figure 31. NMR spectra of compound 23.

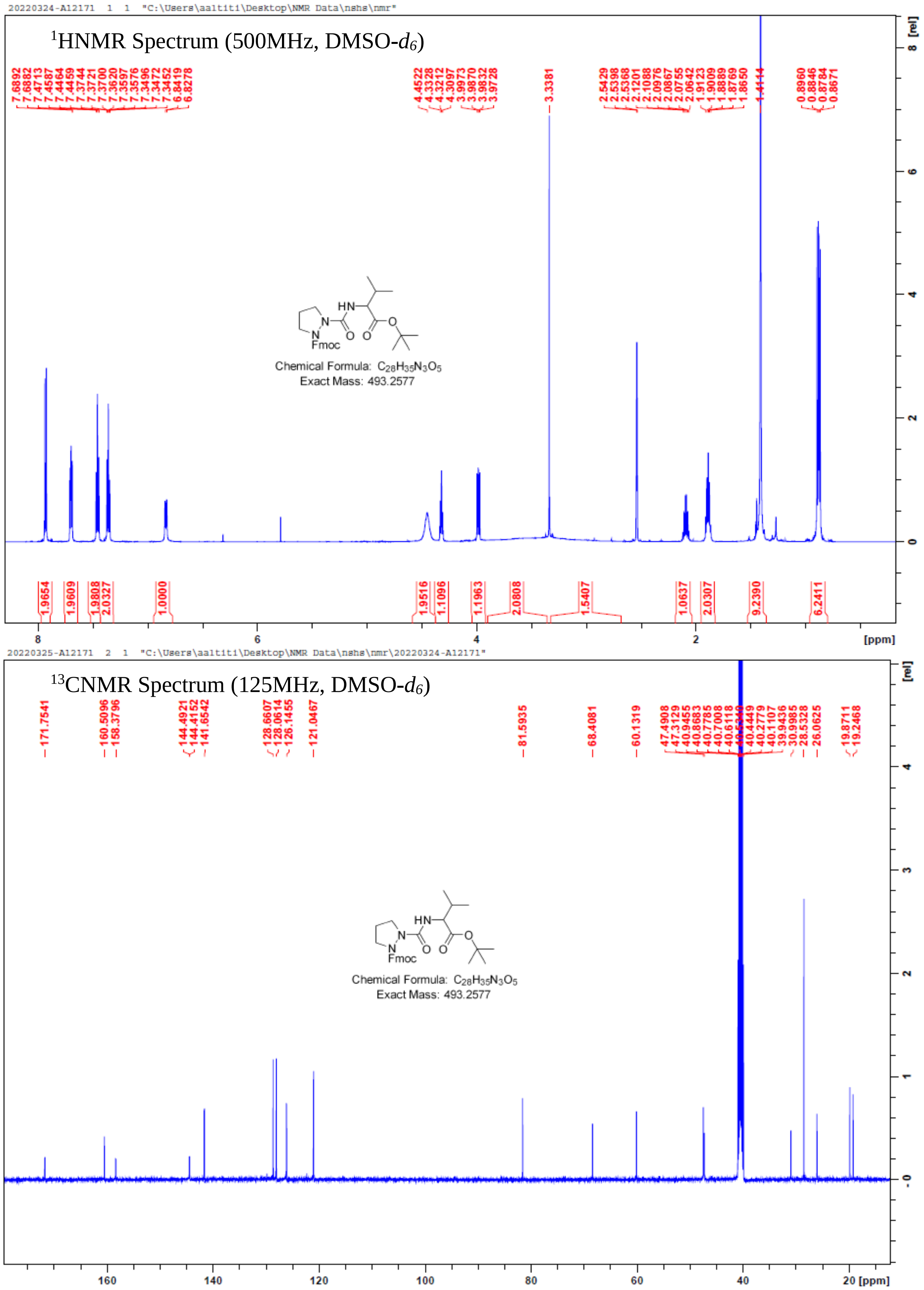

Supplementary Figure 32. NMR spectra of compound 24.

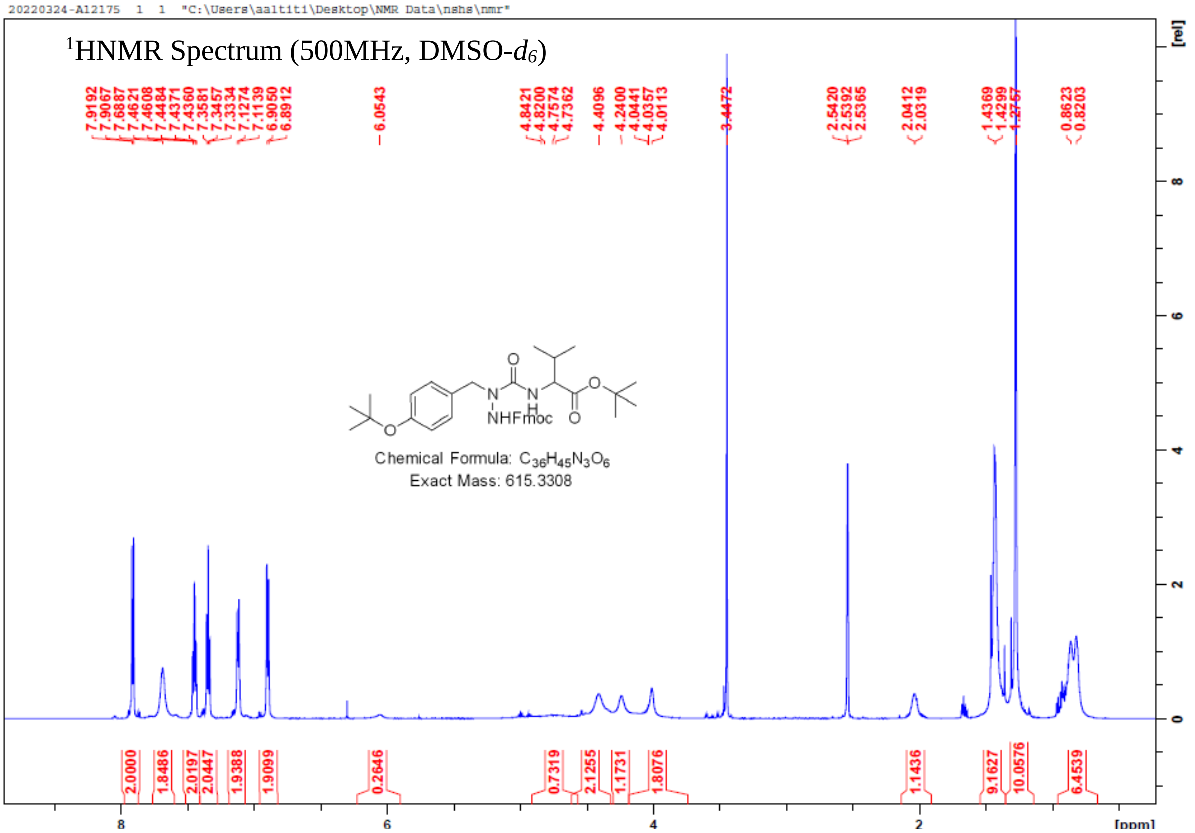

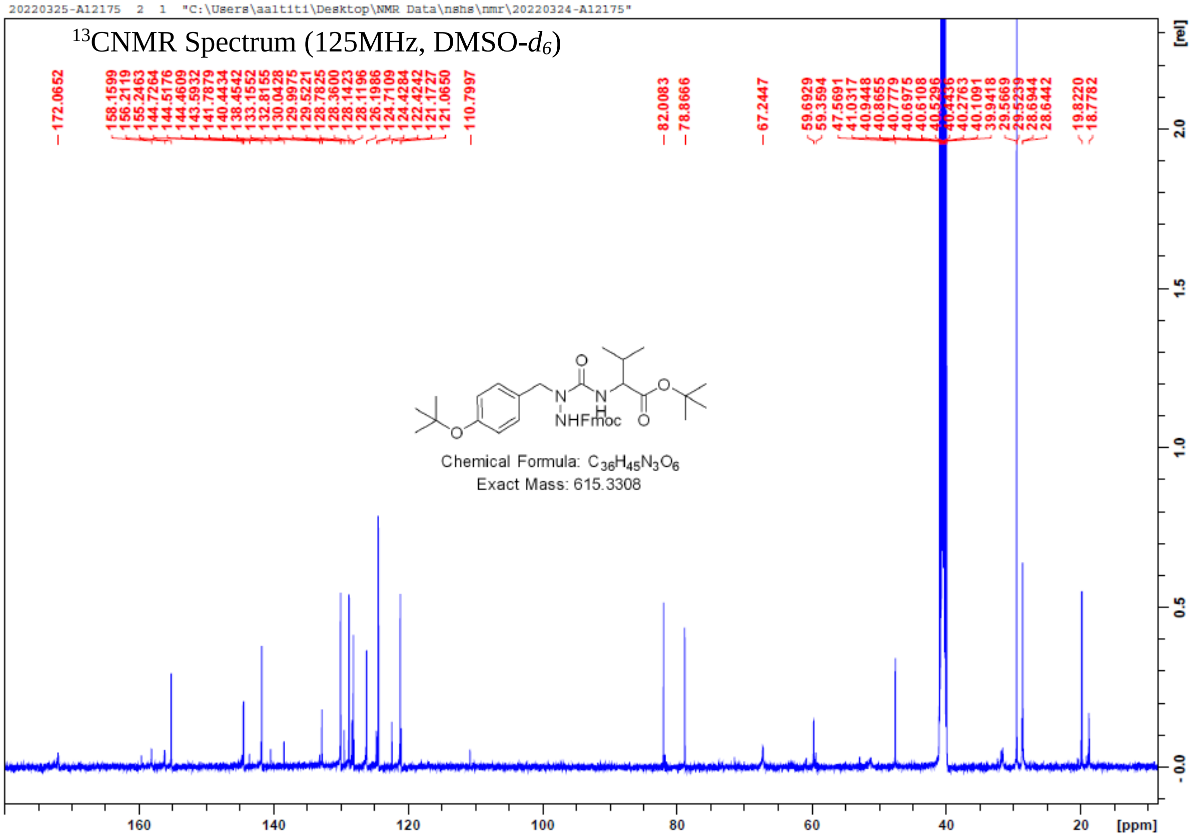

Supplementary Figure 33. NMR spectra of compound 25.

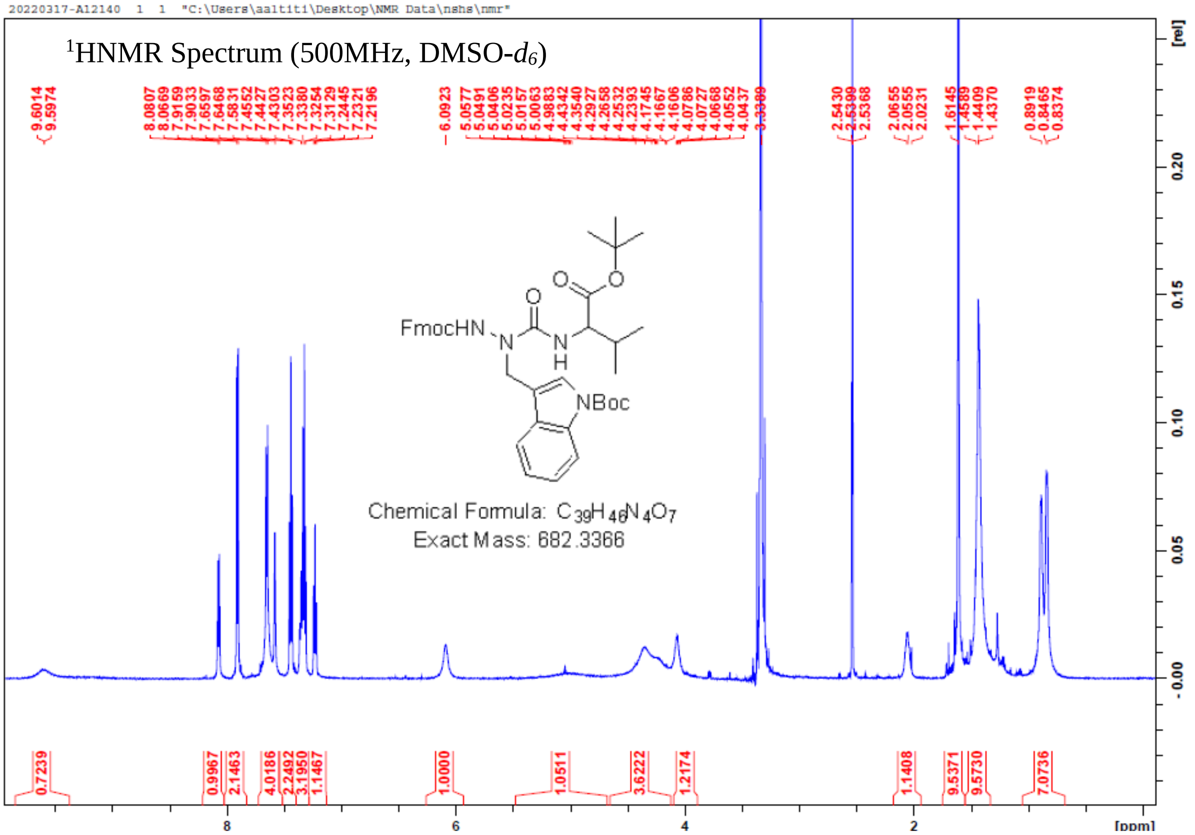

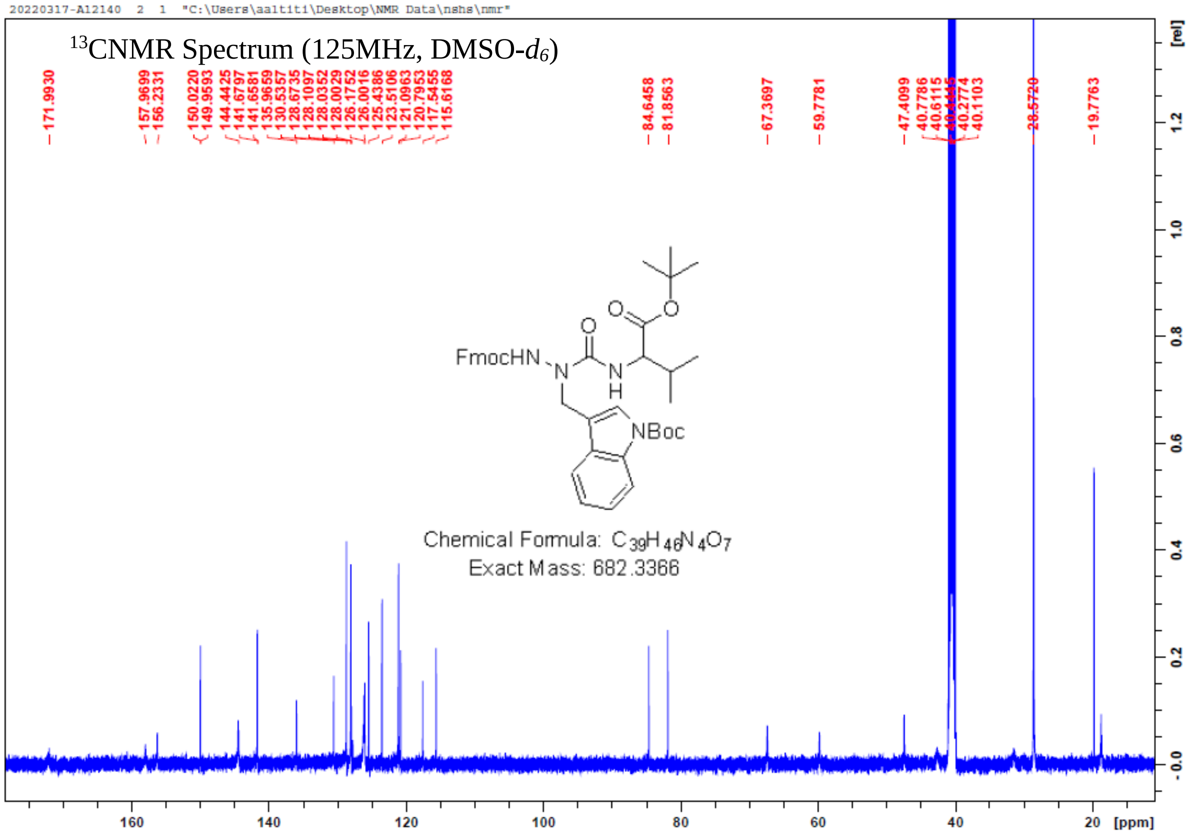

Supplementary Figure 34. NMR spectra of compound 26.

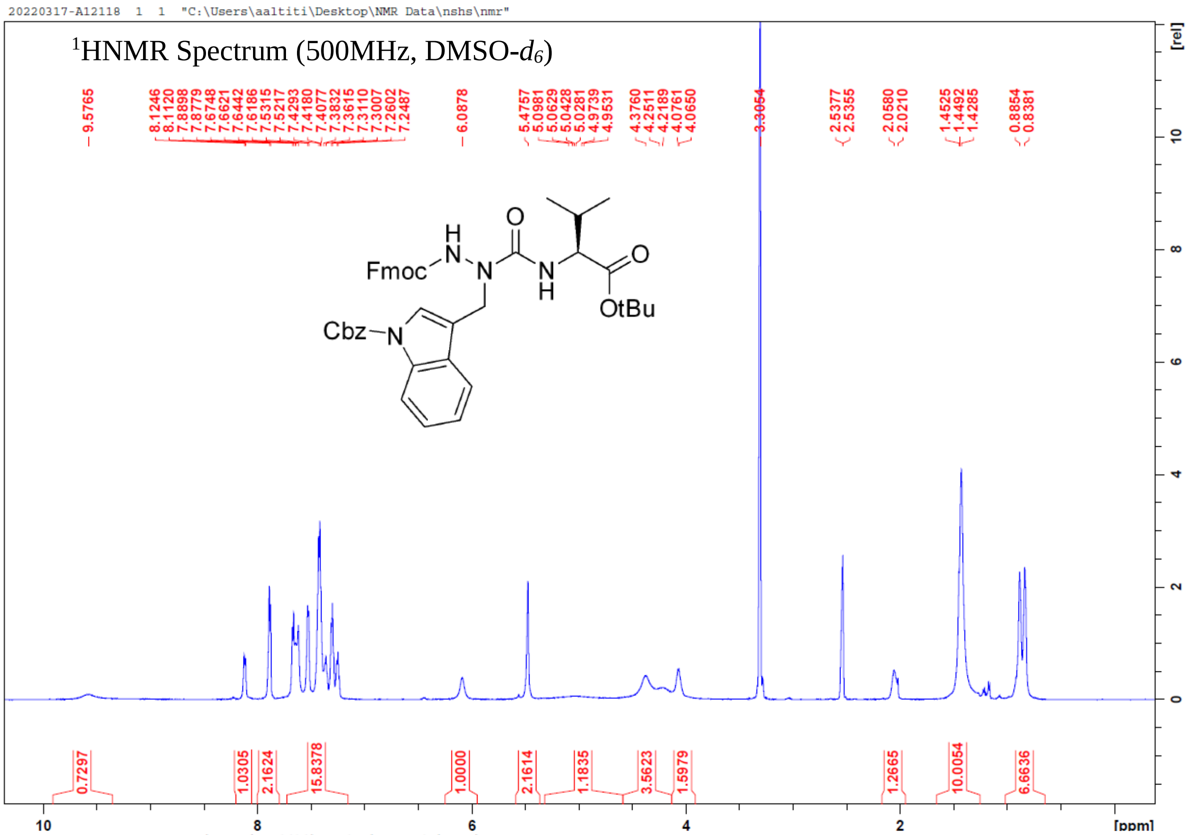

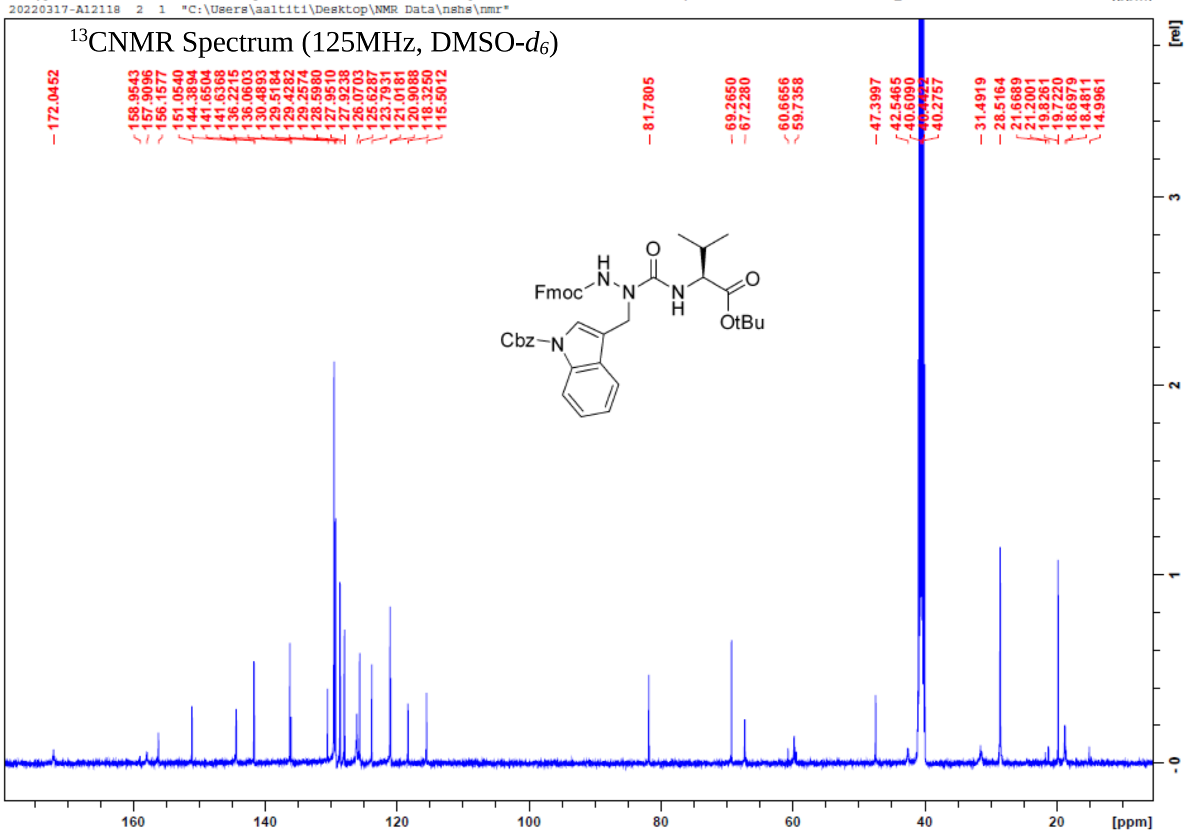

Supplementary Figure 35. NMR spectra of compound 27.

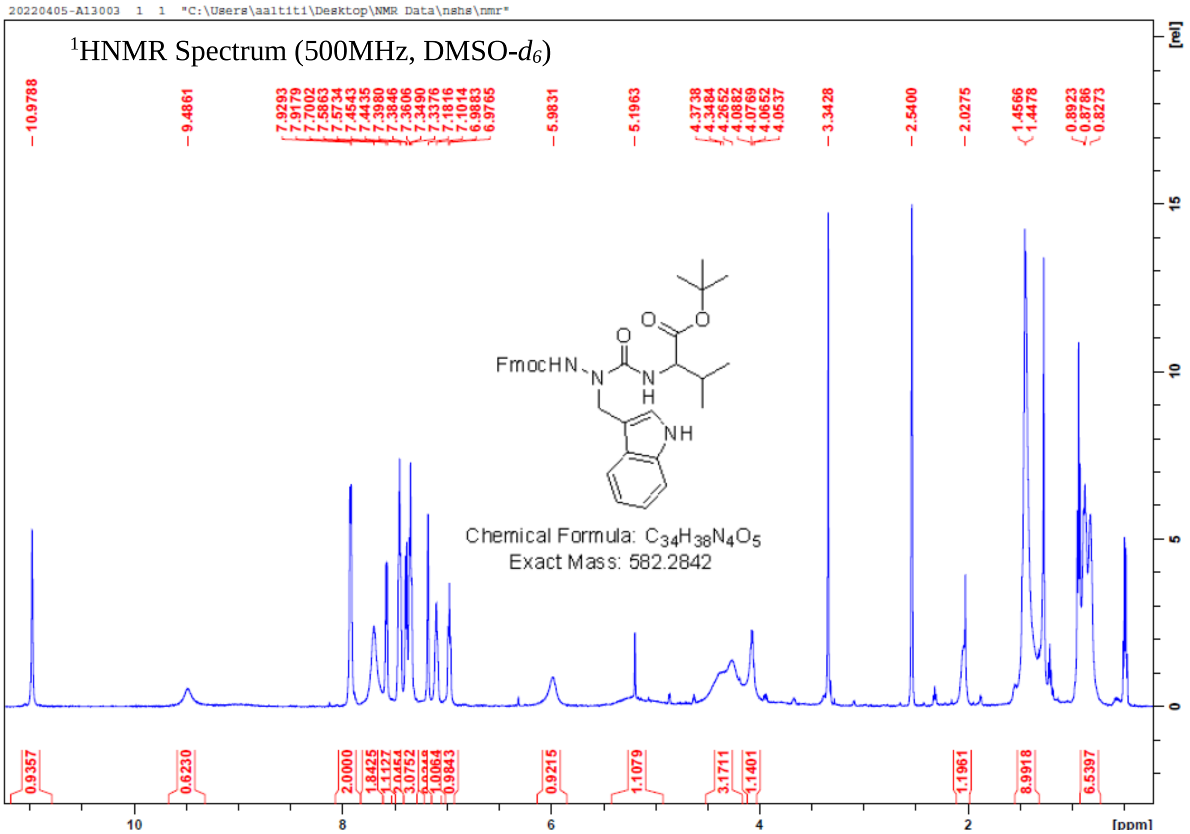

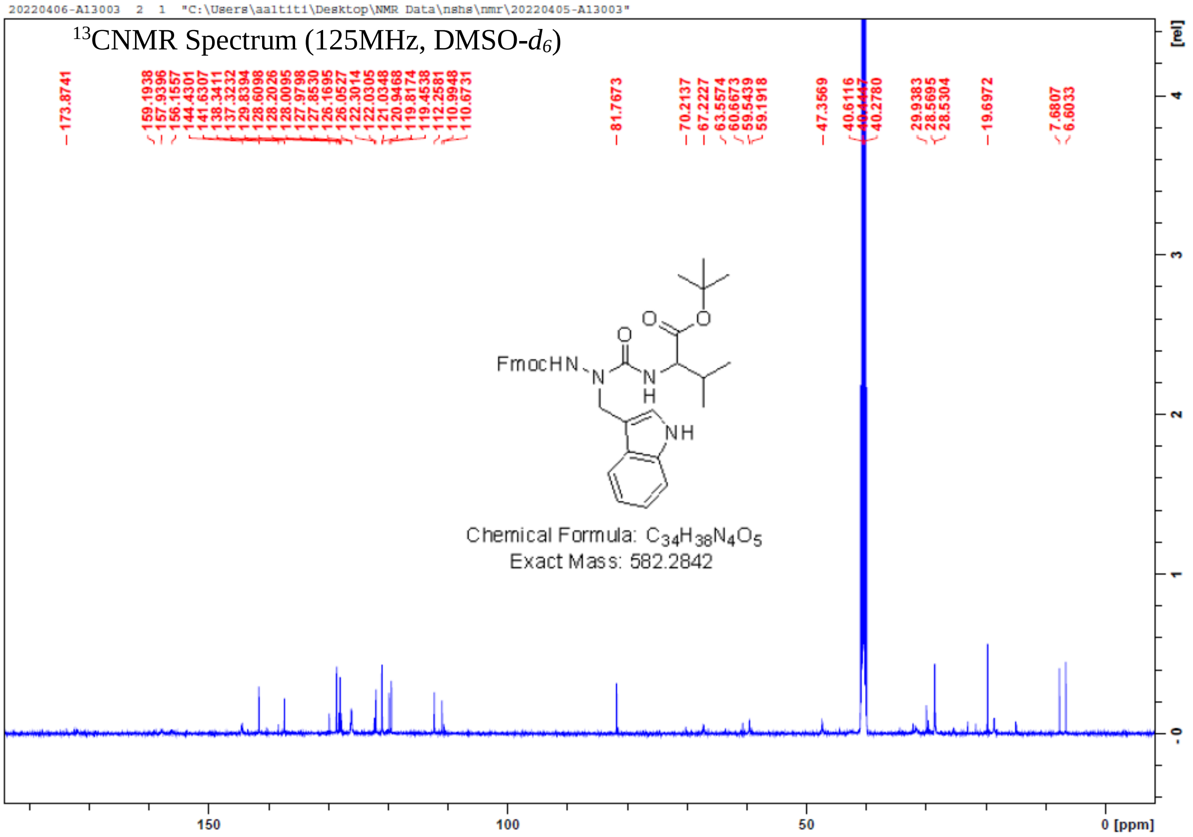

Supplementary Figure 36. NMR spectra of compound 110.

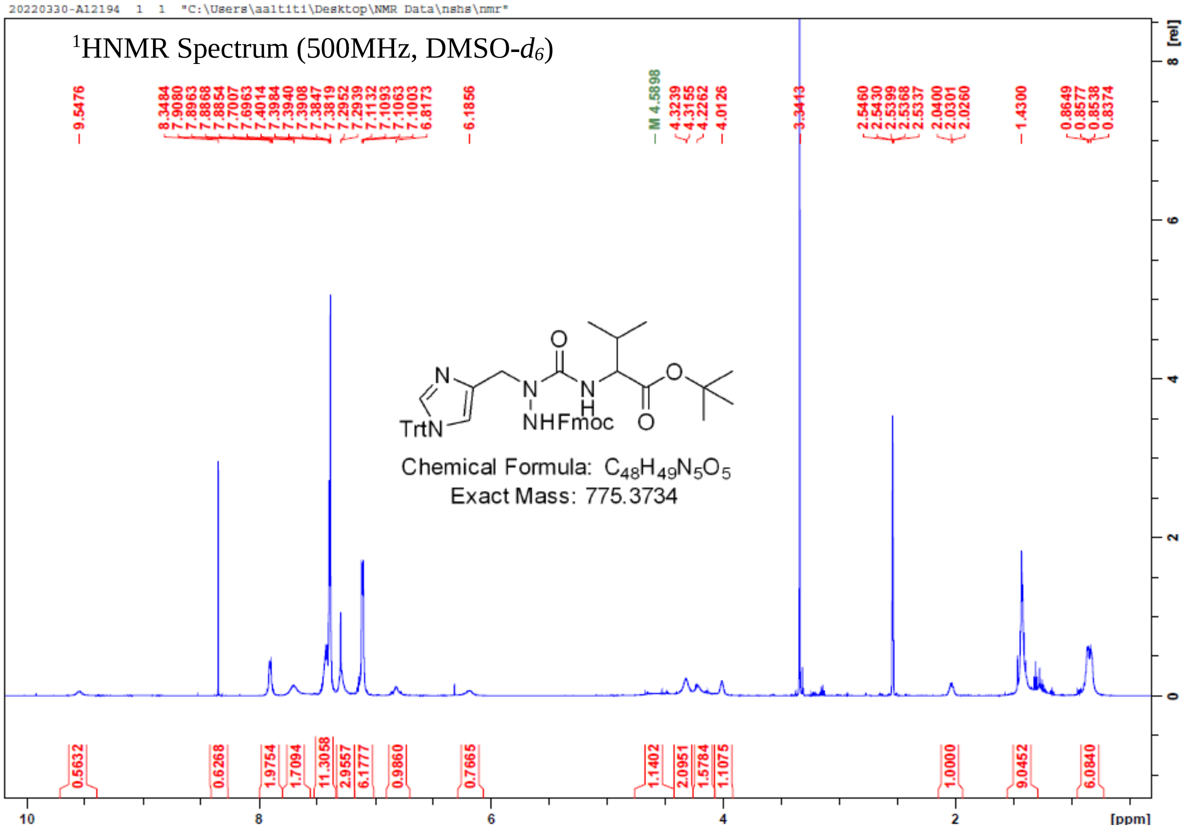

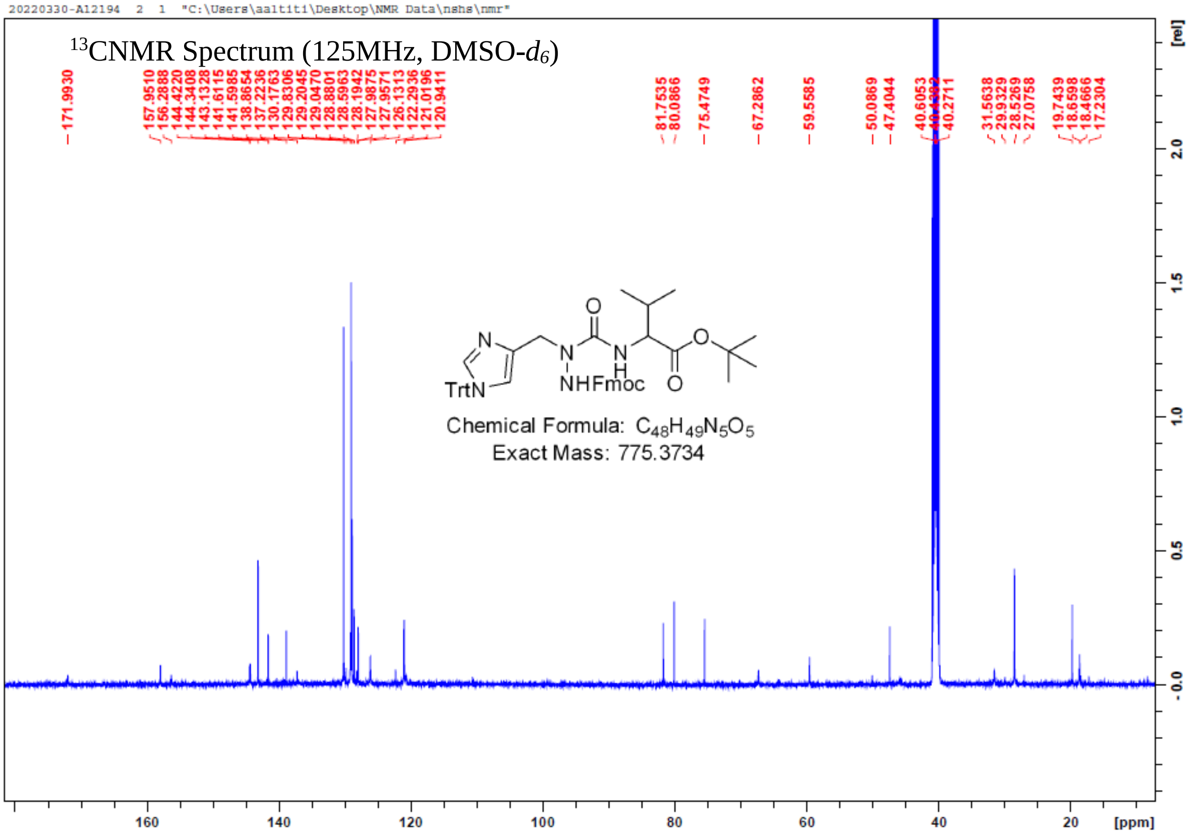

Supplementary Figure 37. NMR spectra of compound 28.

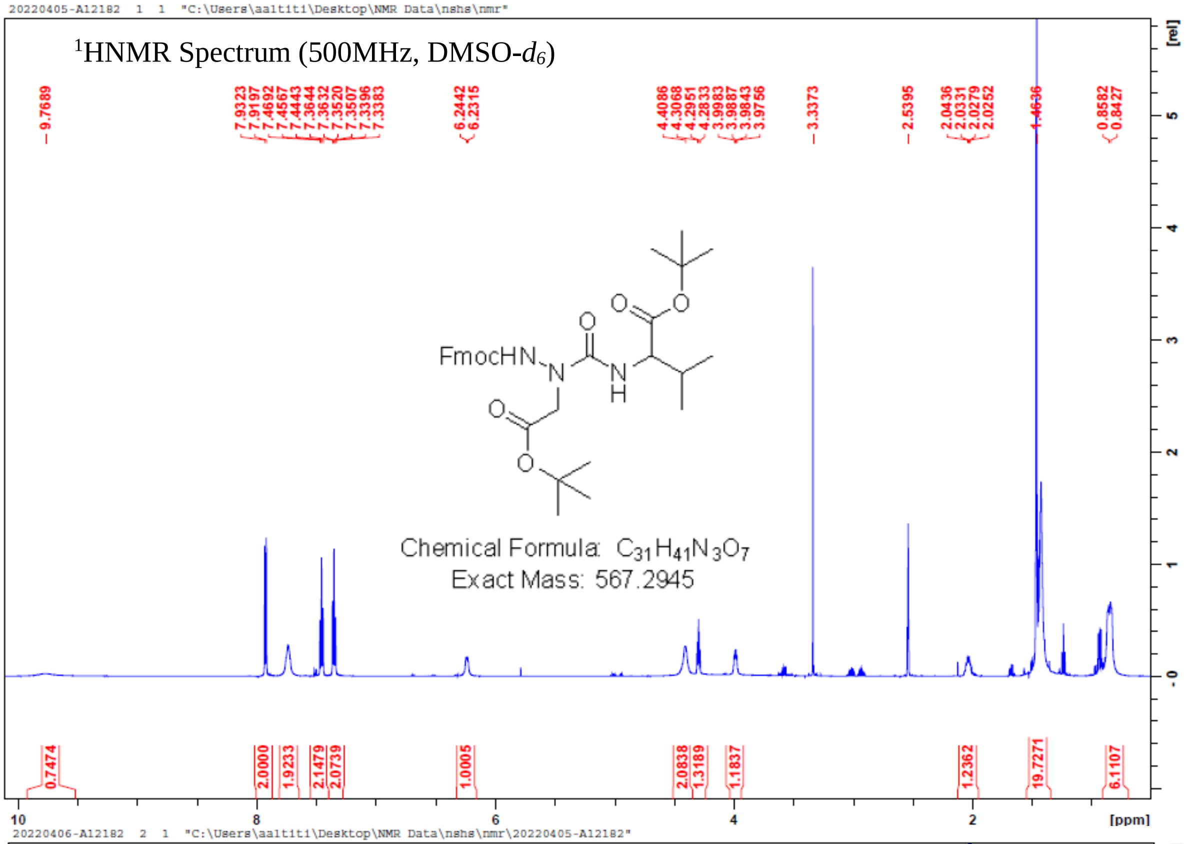

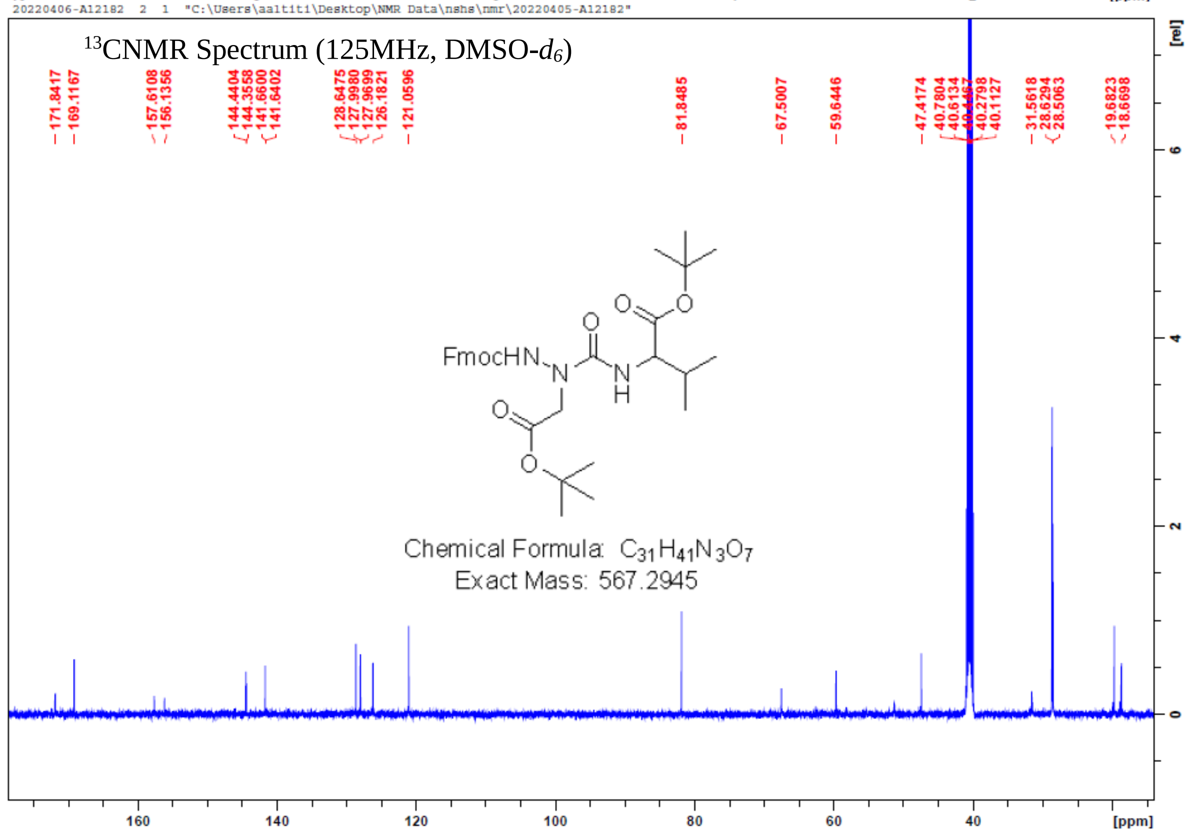

Supplementary Figure 38. NMR spectra of compound 29.

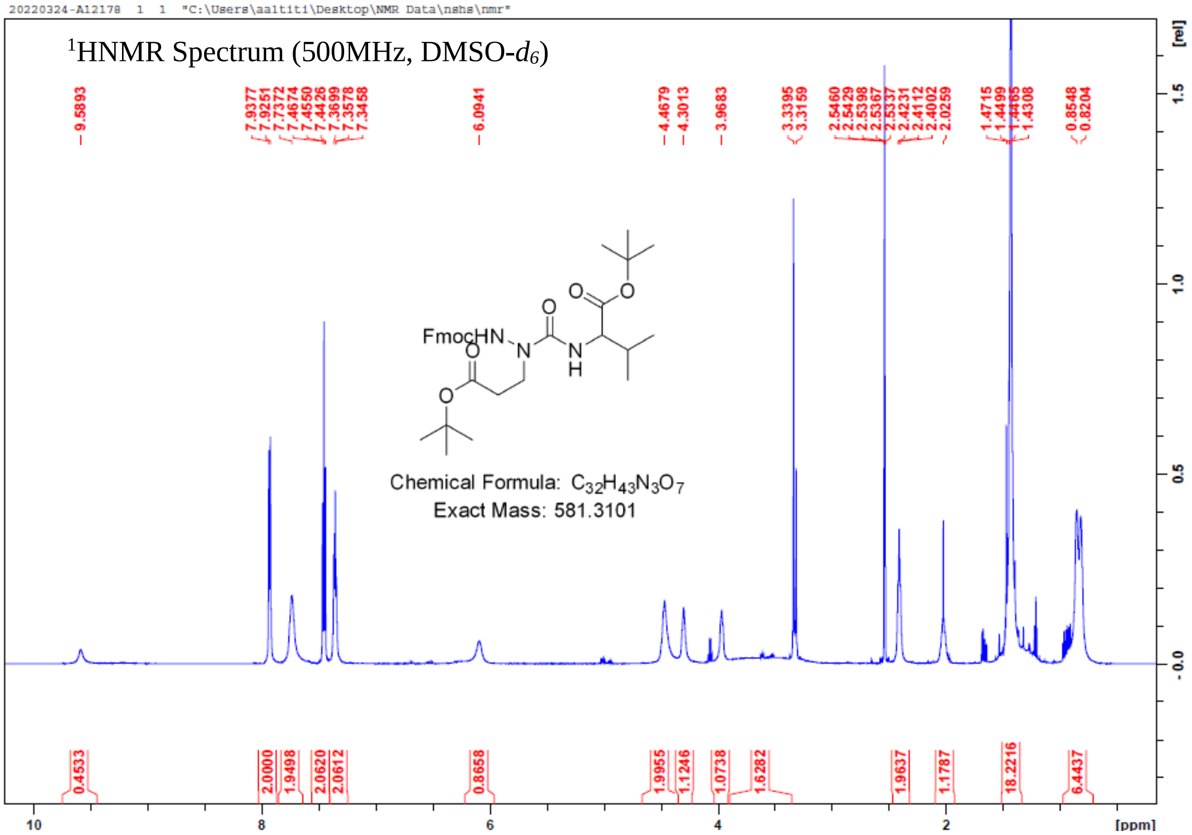

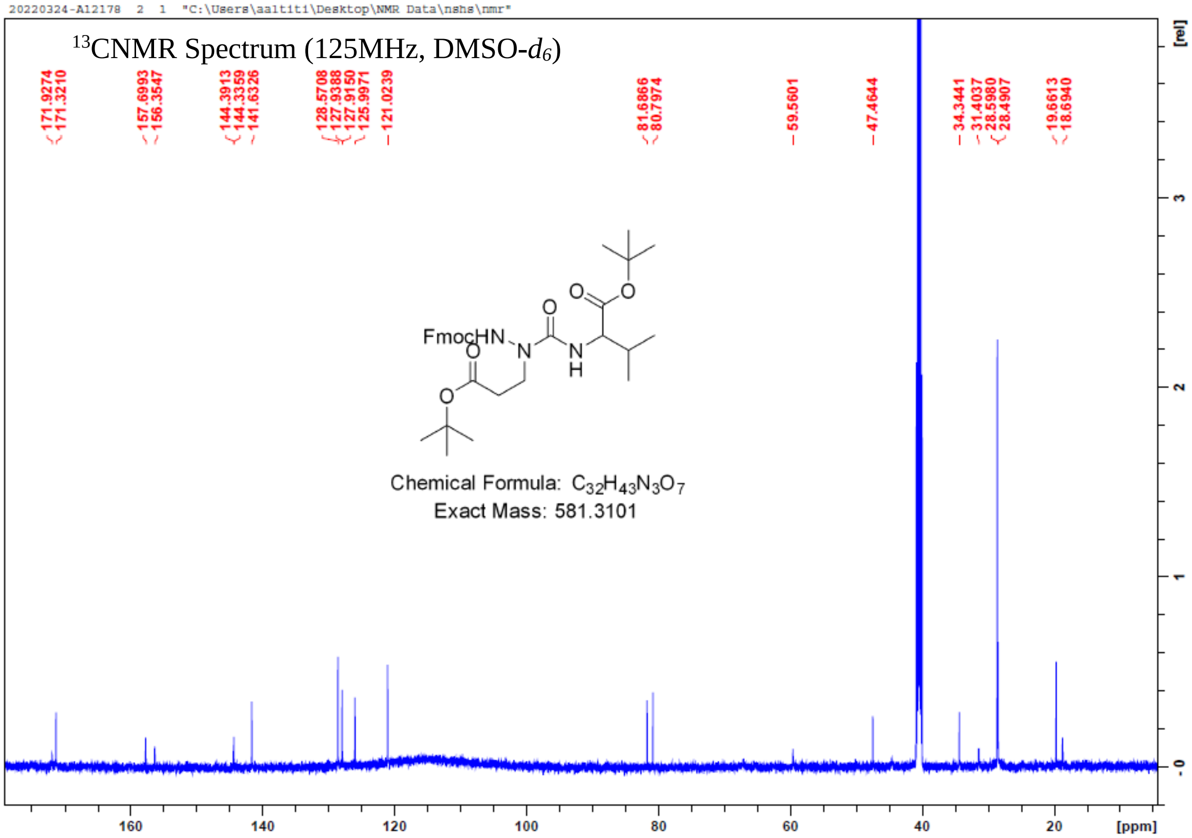

Supplementary Figure 39. NMR spectra of compound 30.

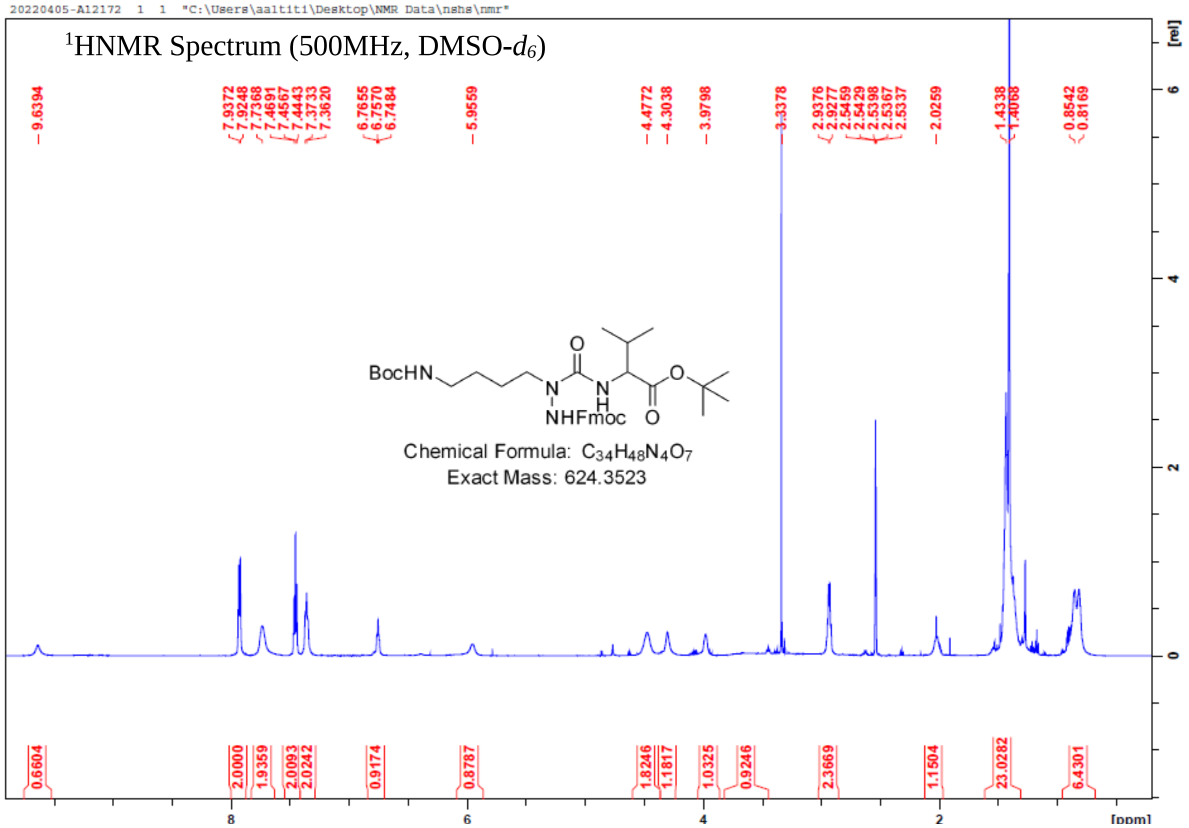

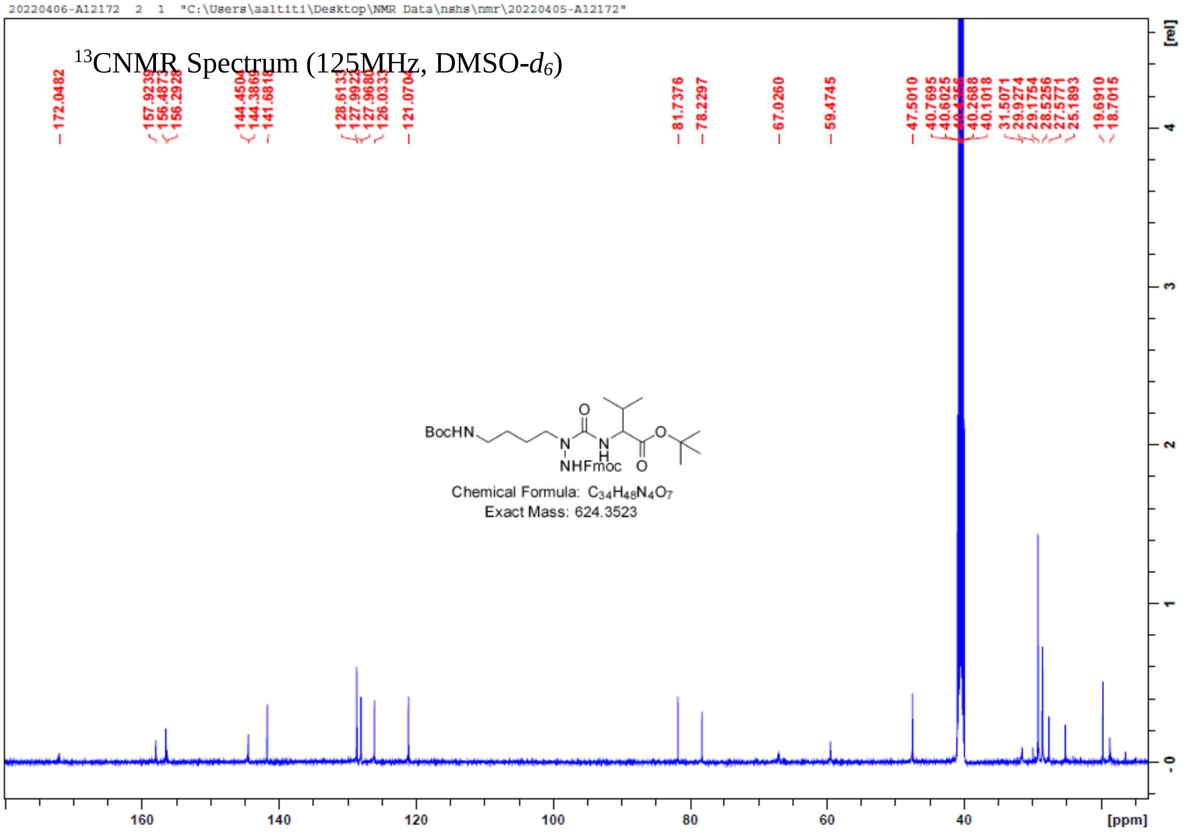

Supplementary Figure 40. NMR spectra of compound 31.

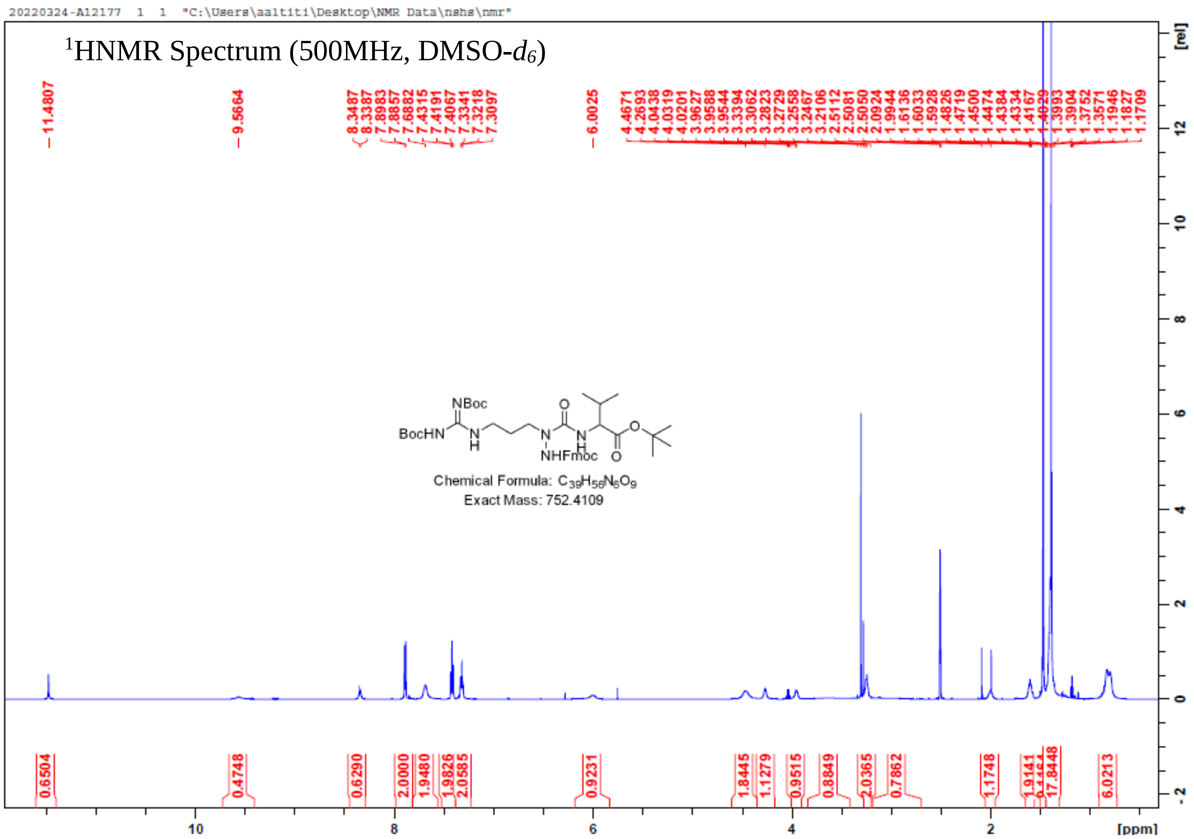

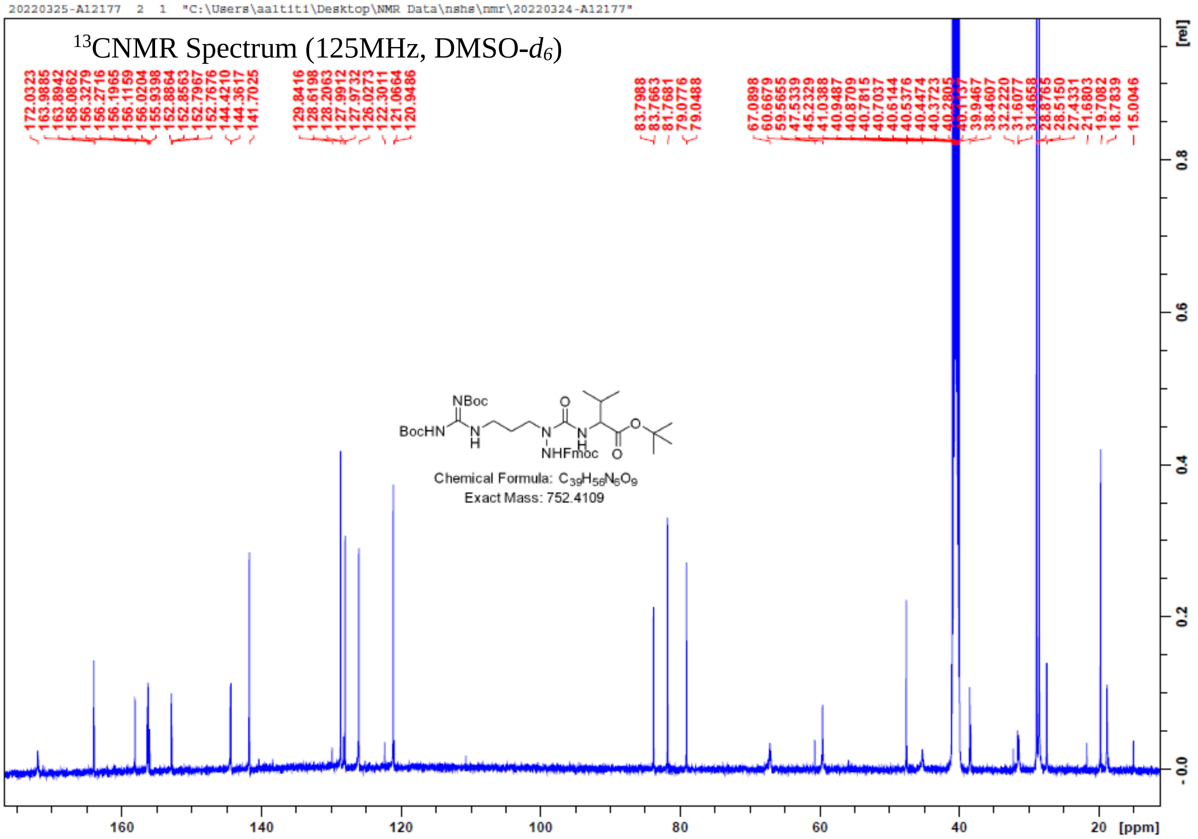

Supplementary Figure 41. NMR spectra of compound 32.

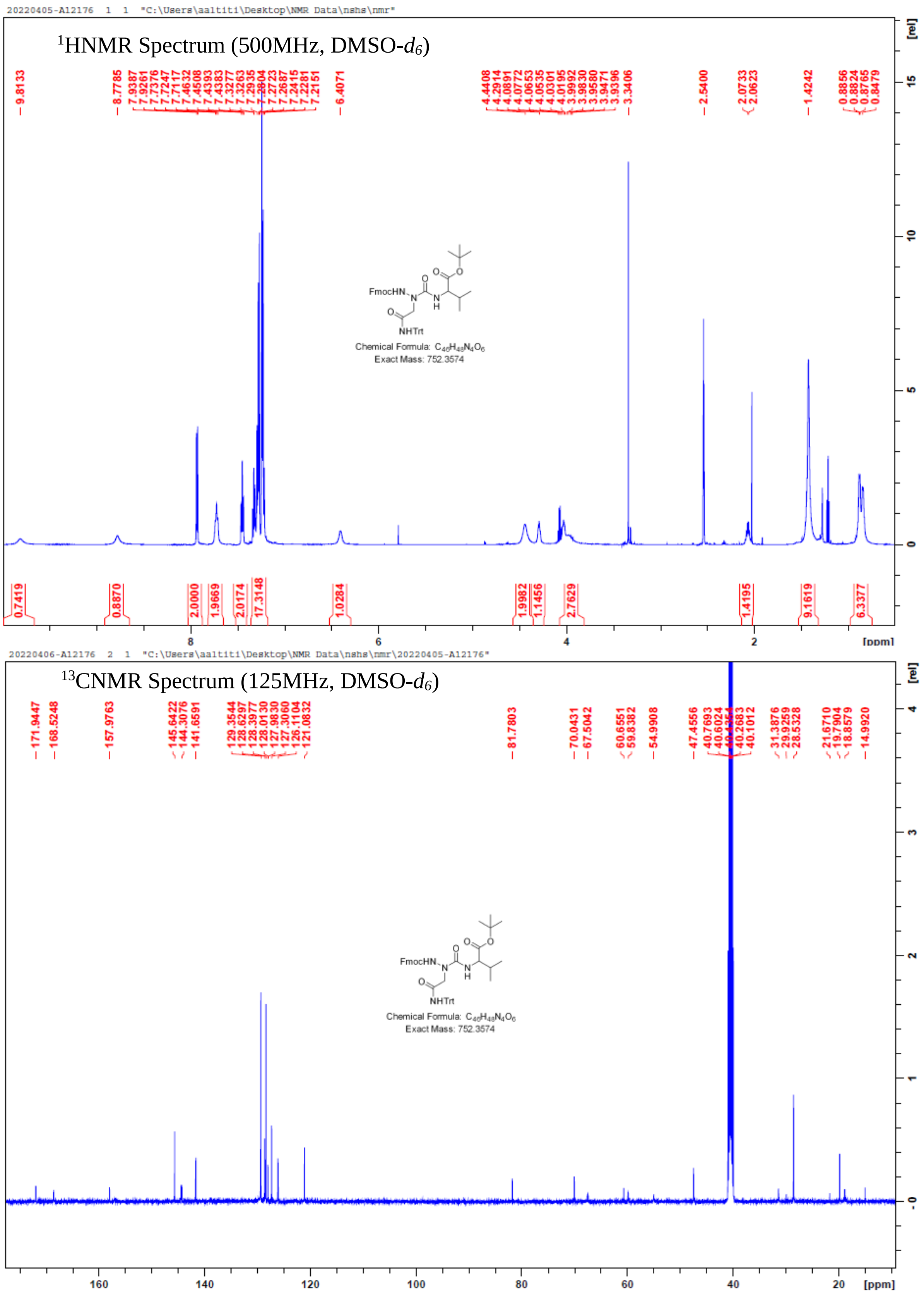

Supplementary Figure 42. NMR spectra of compound 33.

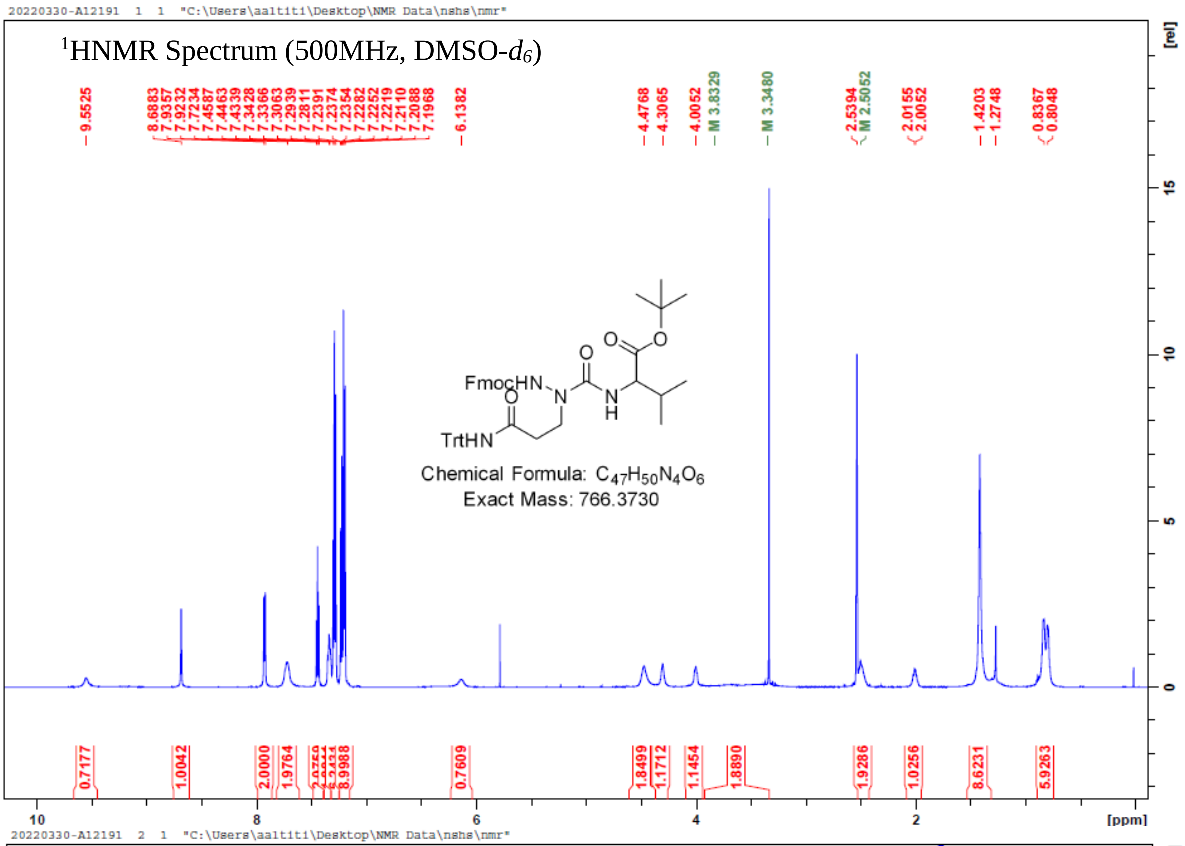

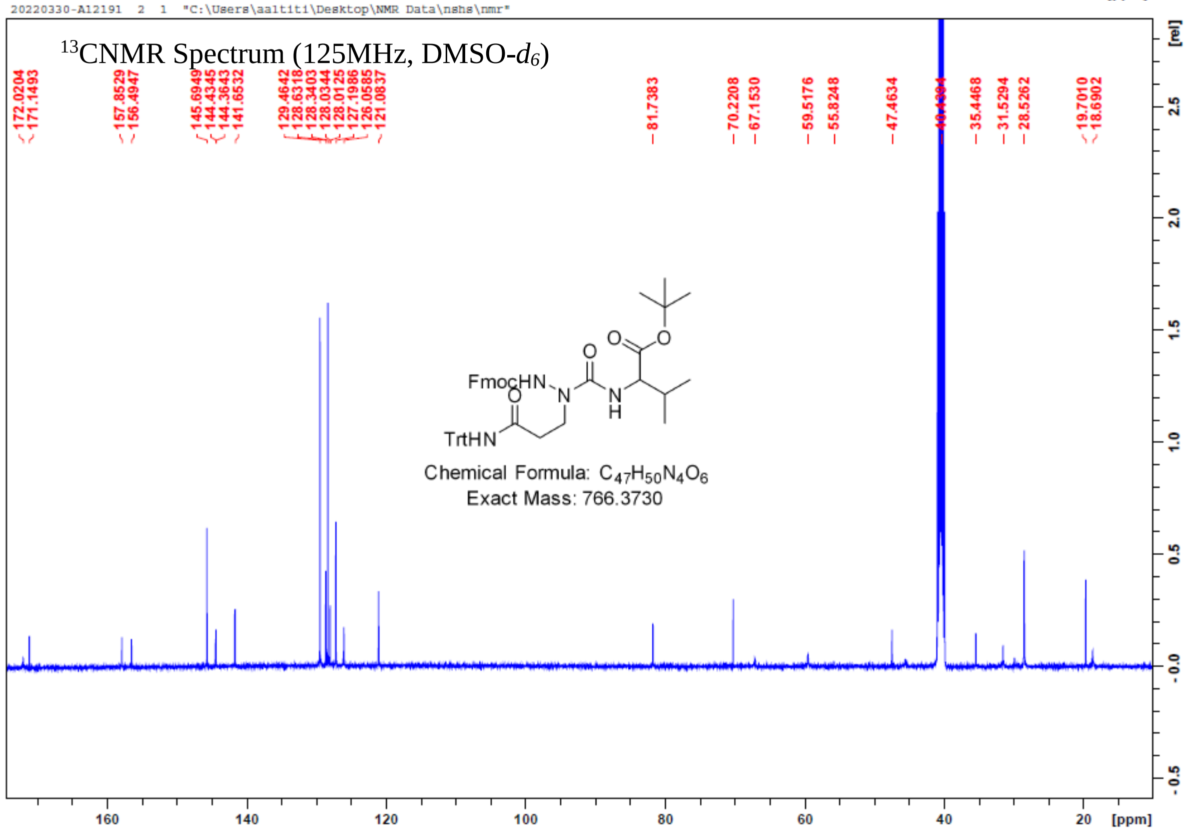

Supplementary Figure 43. NMR spectra of compound 34.

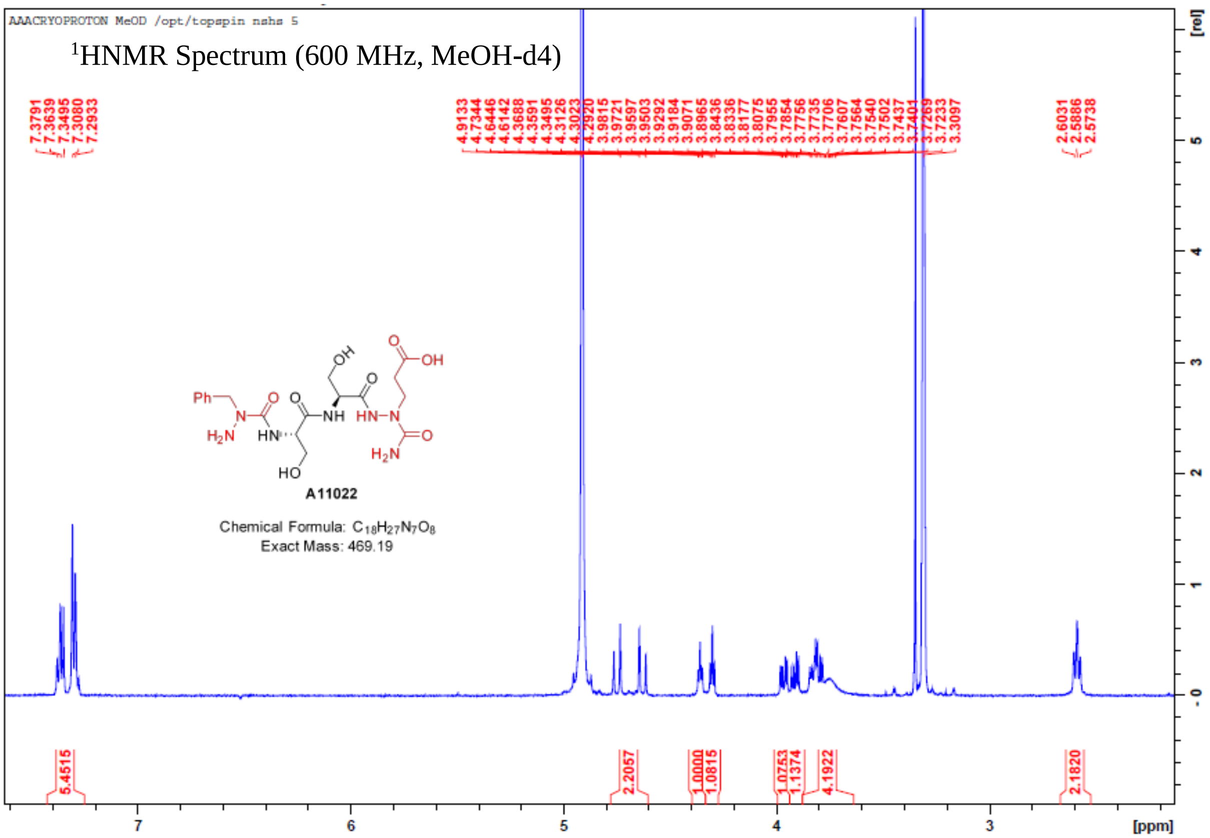

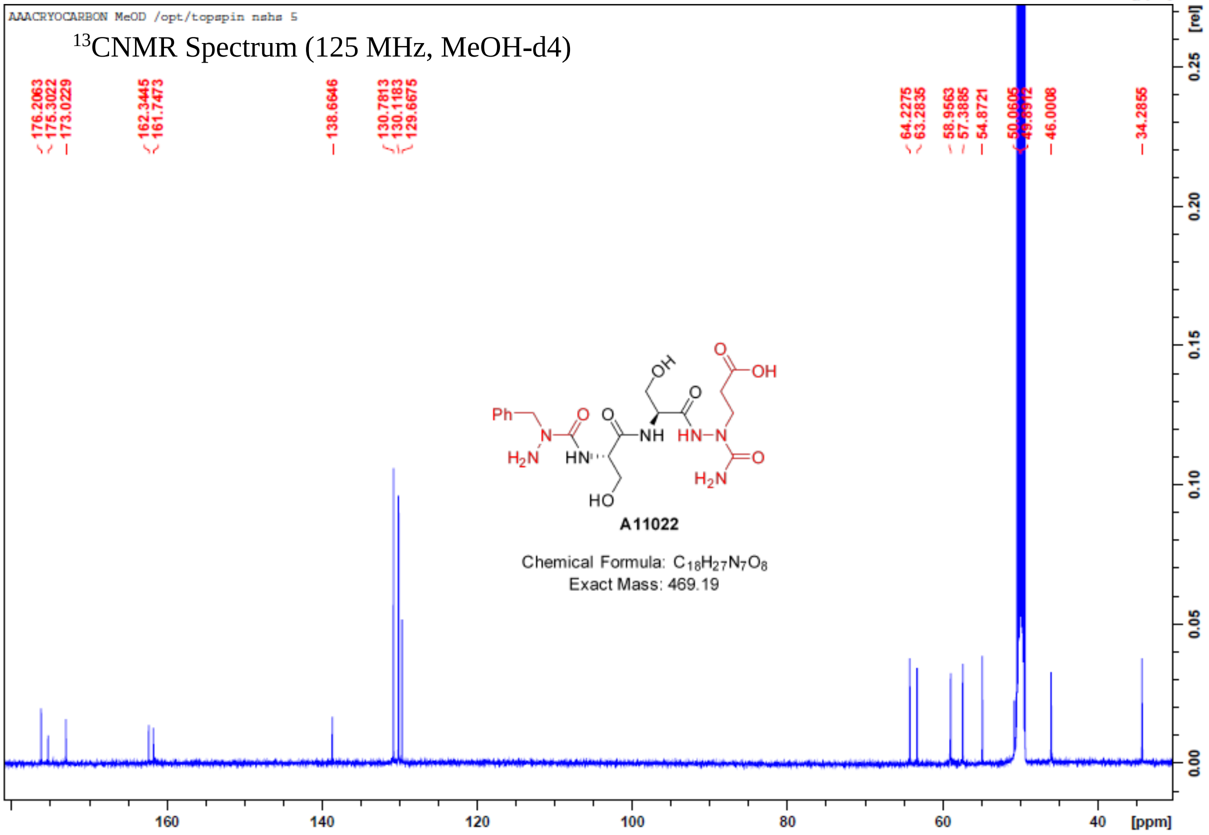

Supplementary Figure 44. NMR spectra of compound 51.

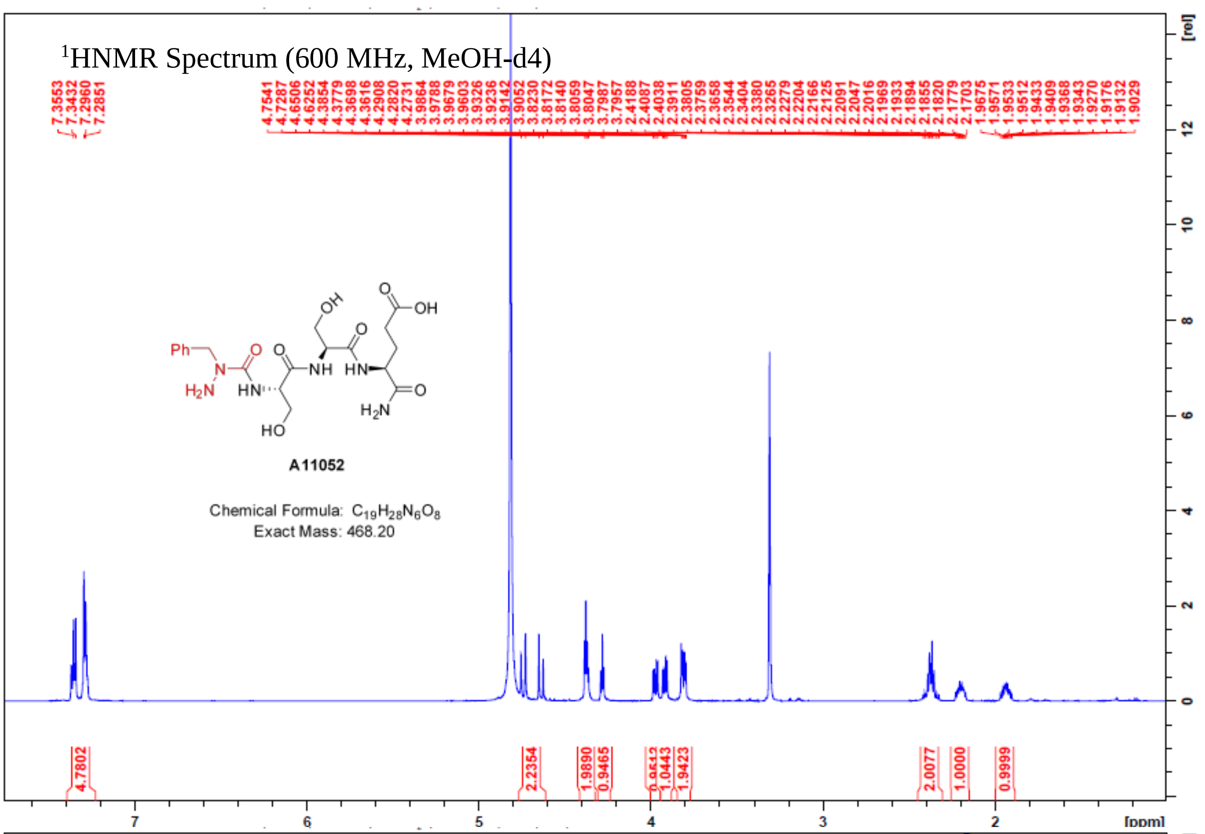

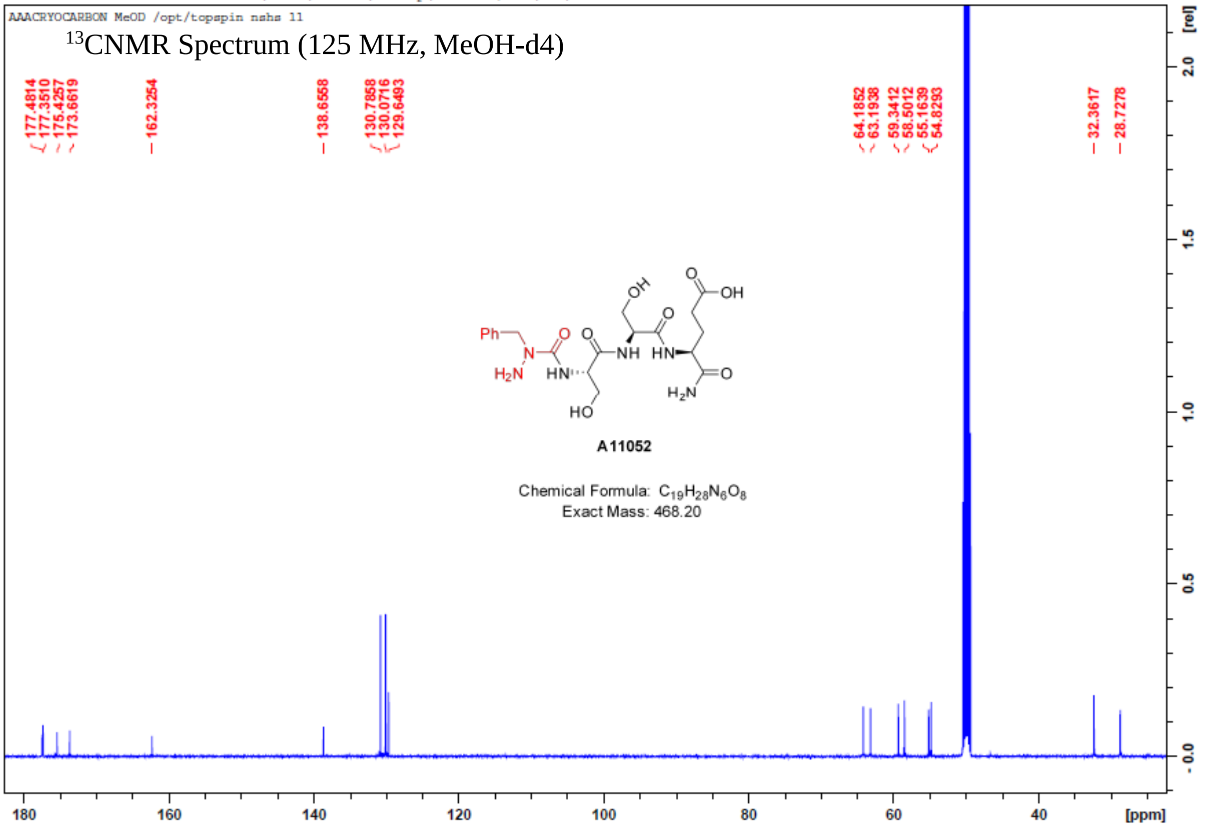

Supplementary Figure 45. NMR spectra of compound 52.

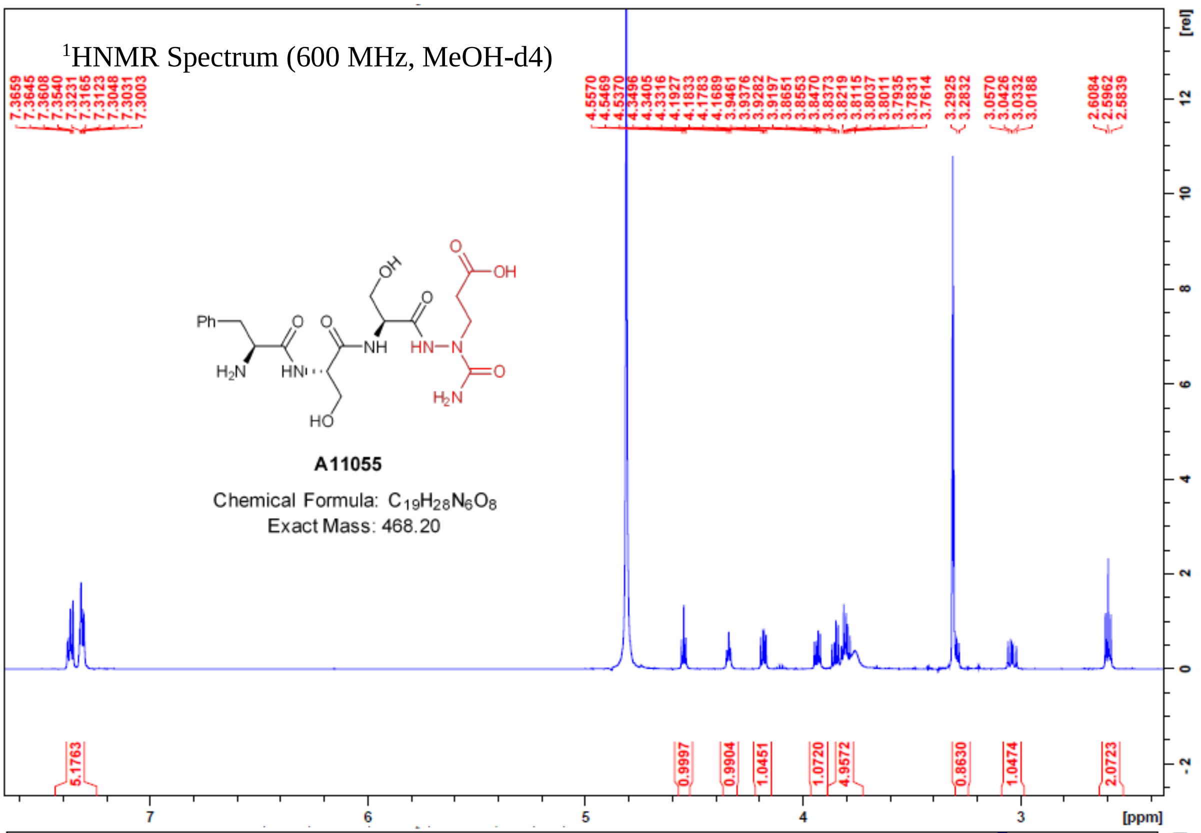

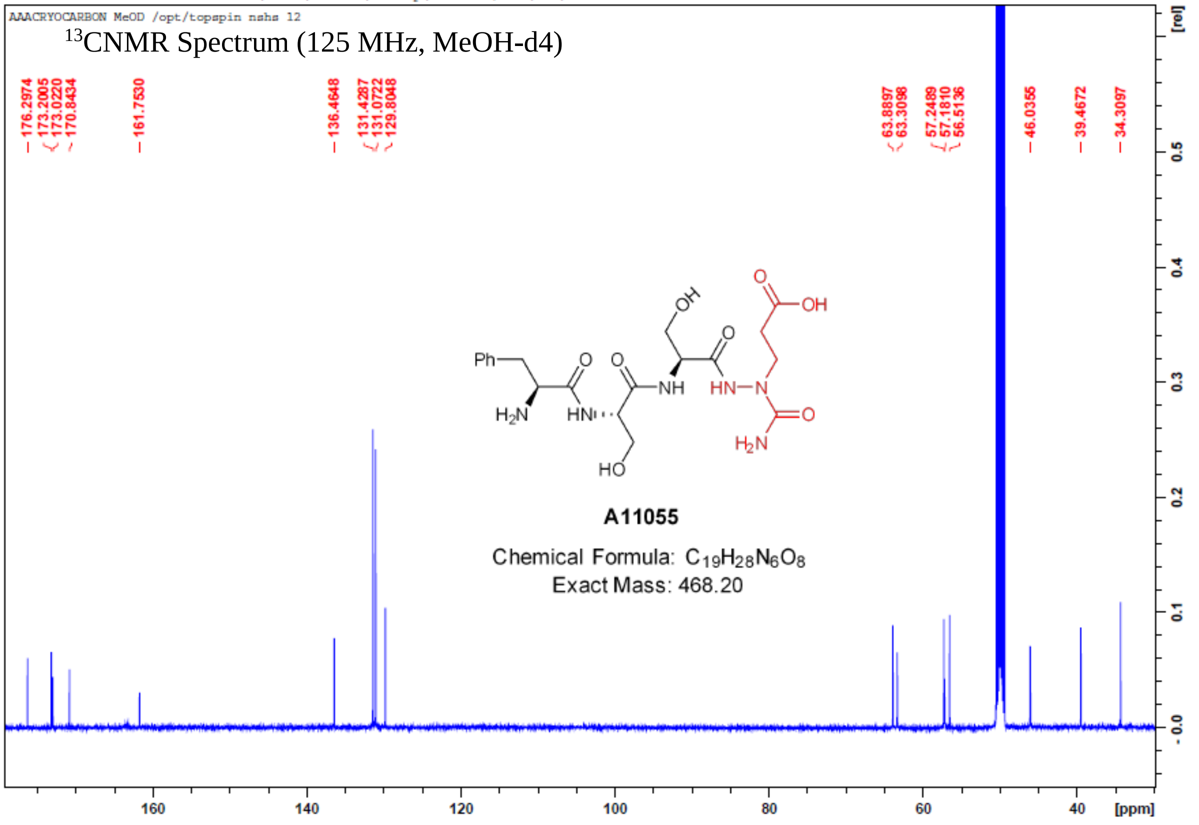

Supplementary Figure 46. NMR spectra of compound 53.

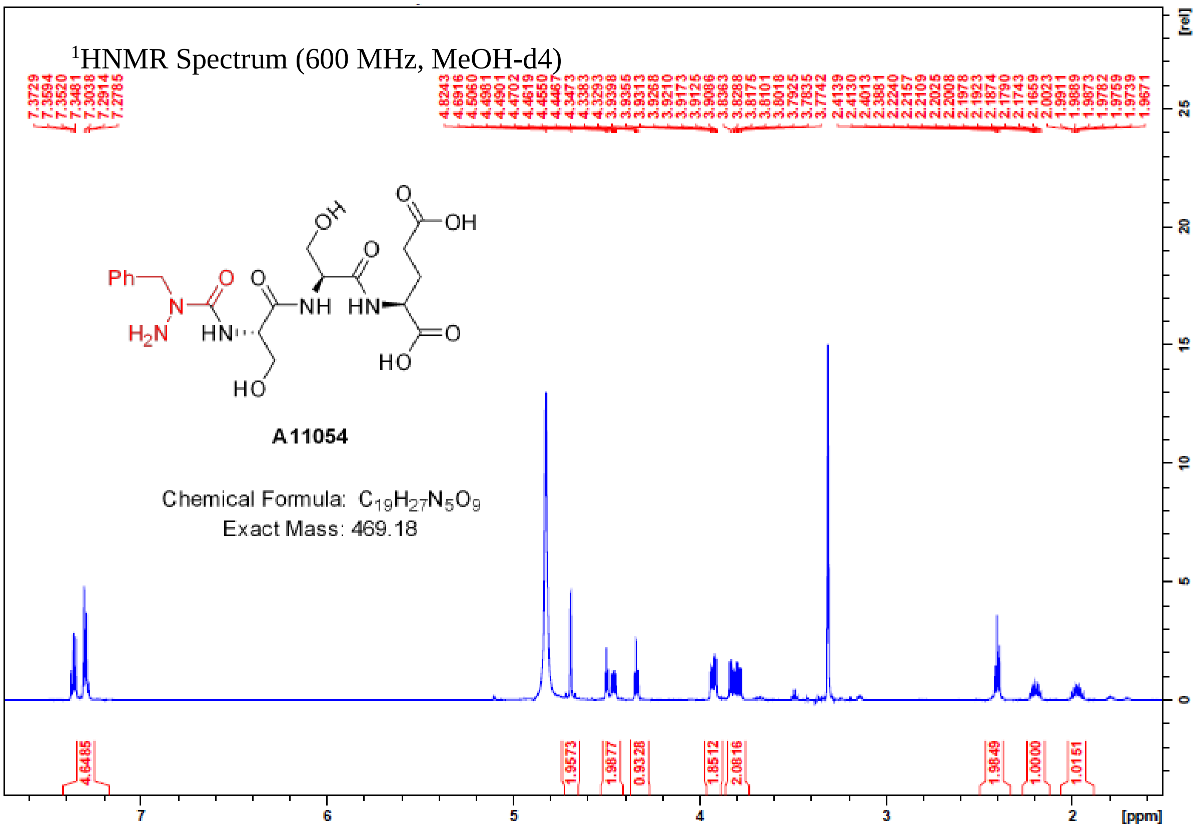

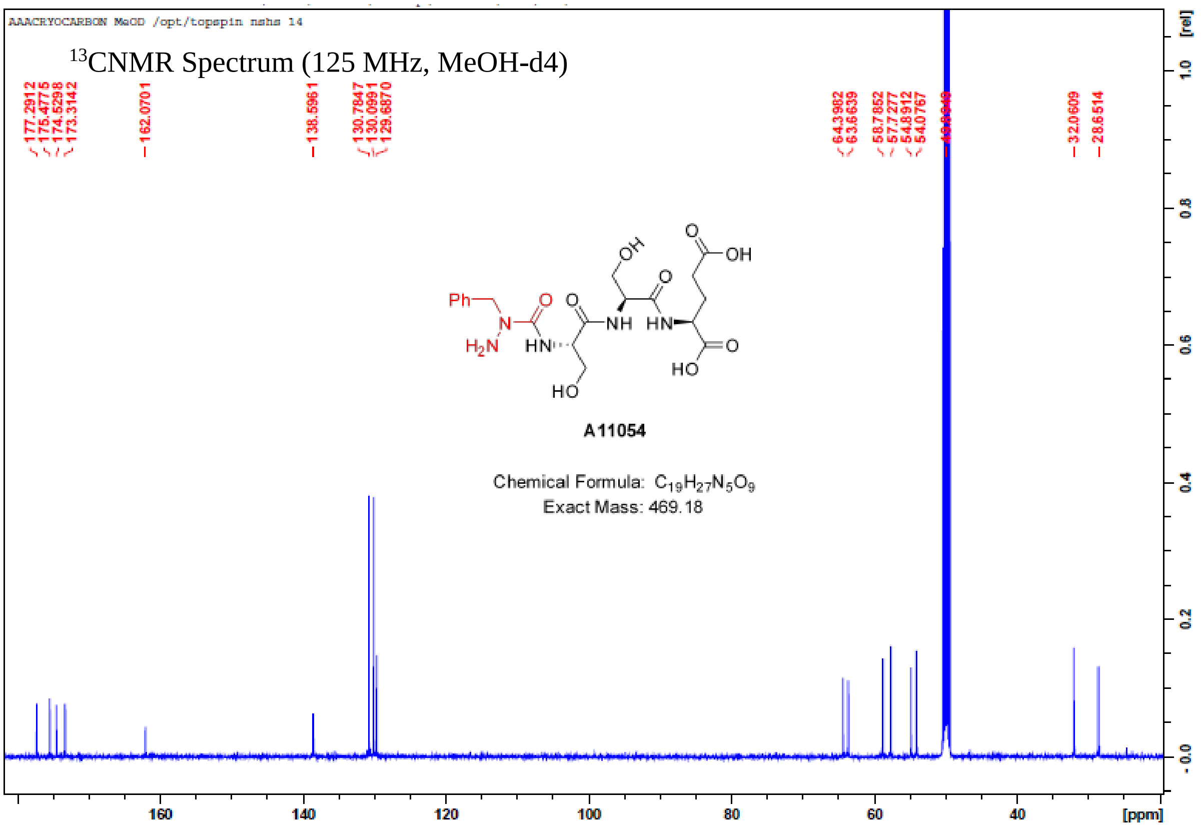

Supplementary Figure 47. NMR spectra of compound 54.

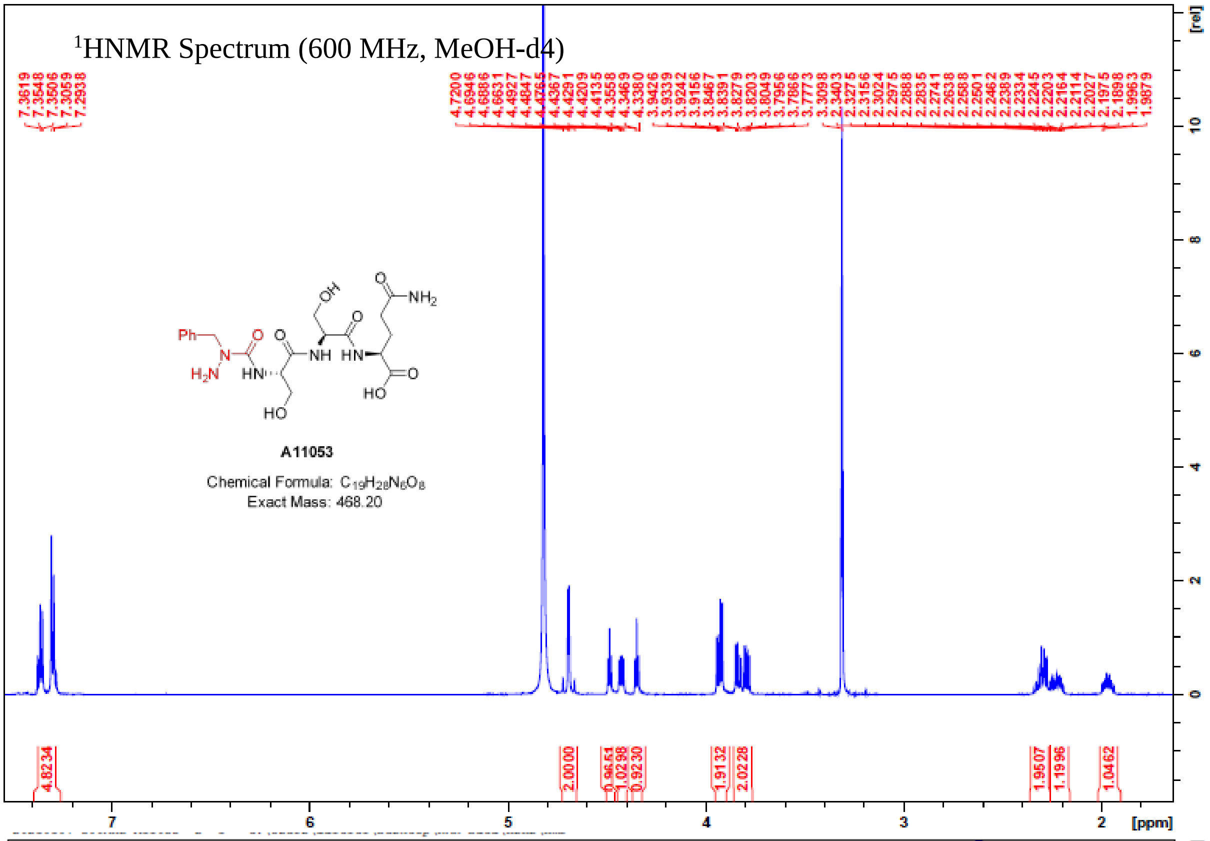

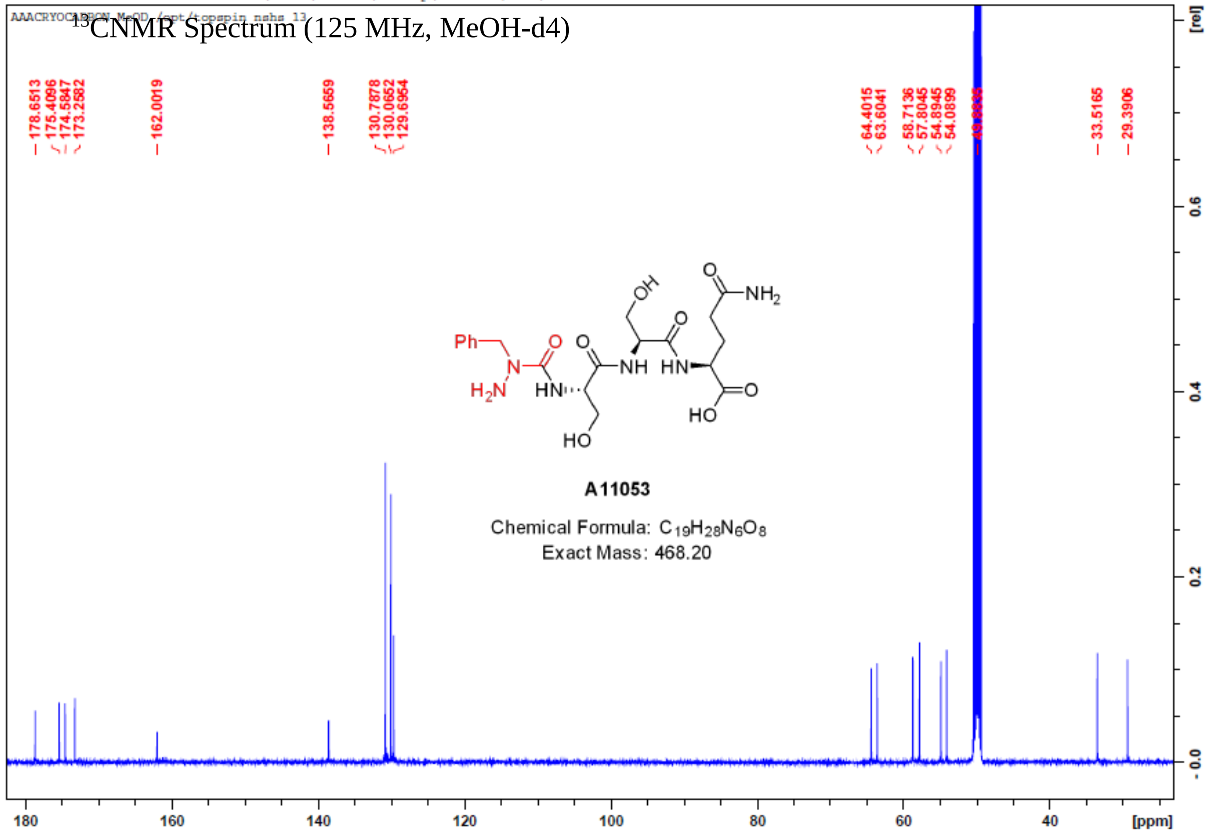

Supplementary Figure 48. NMR spectra of compound 55.

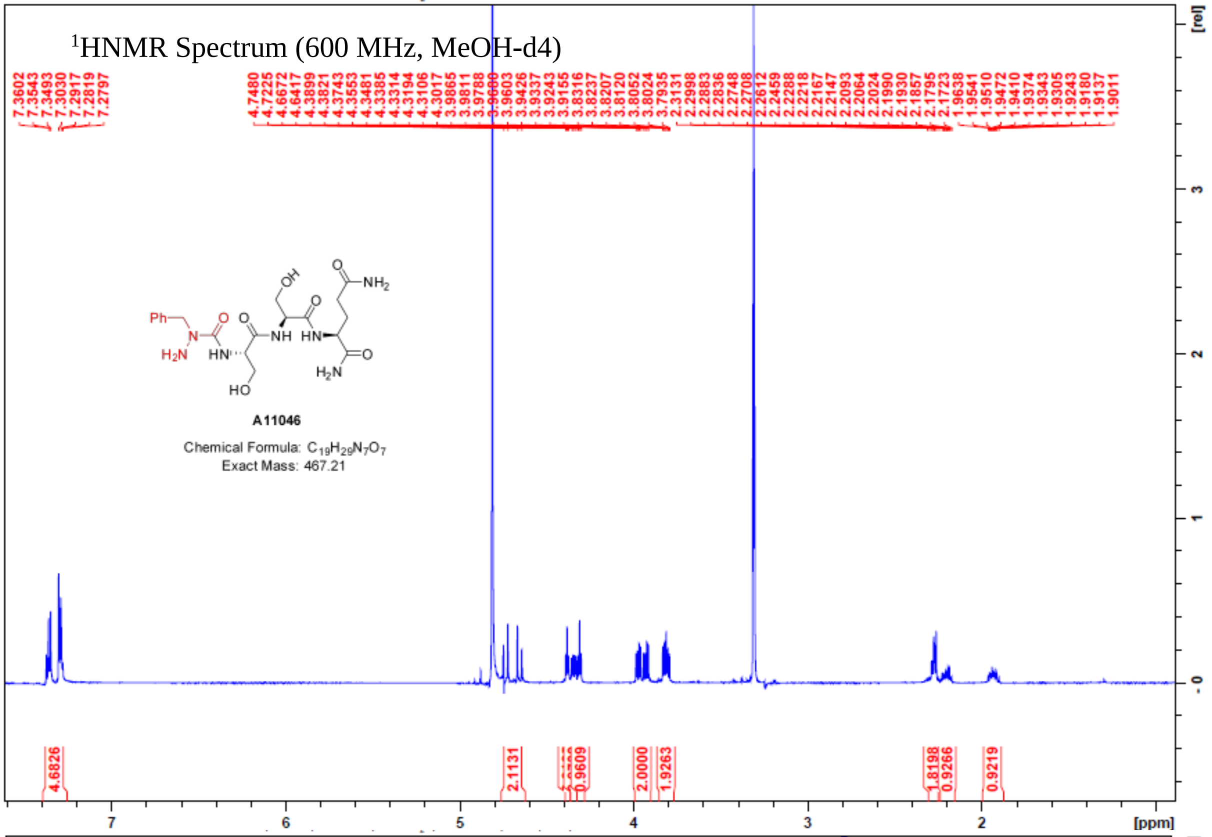

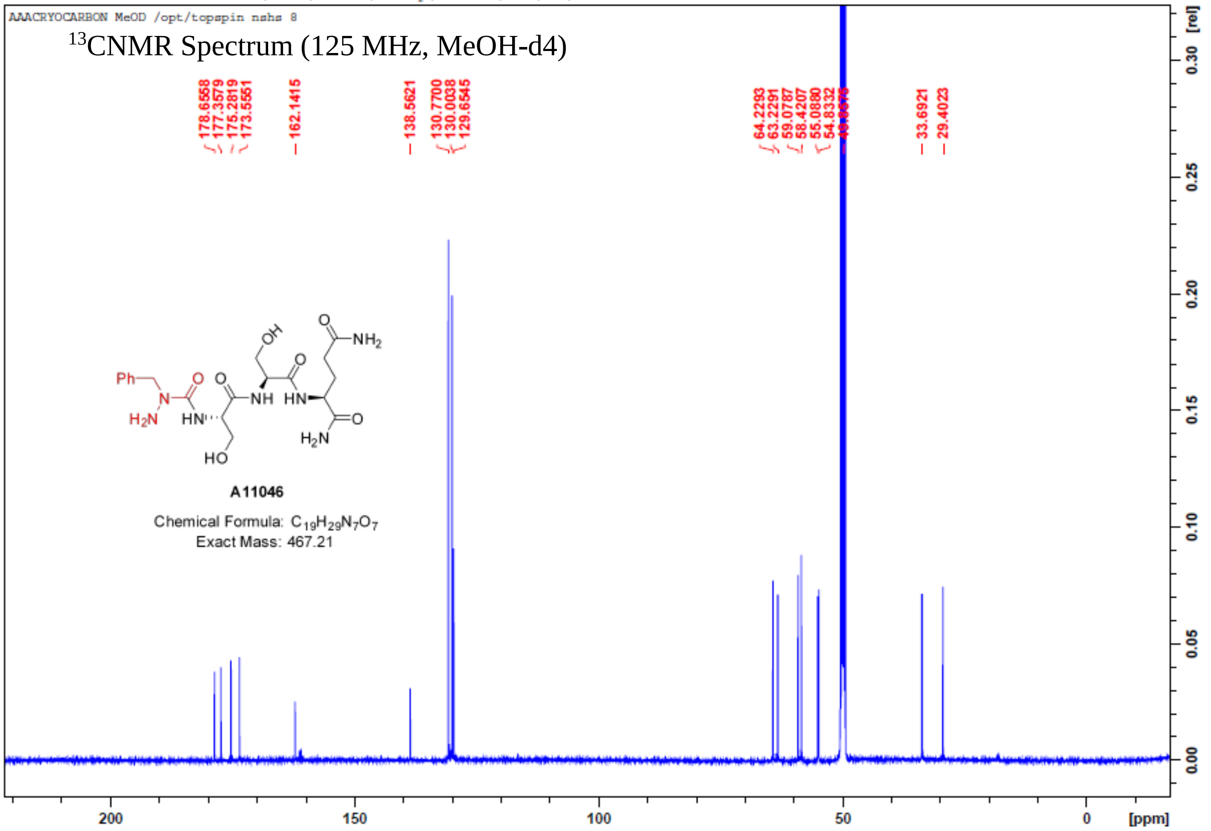

Supplementary Figure 49. NMR spectra of compound 56.

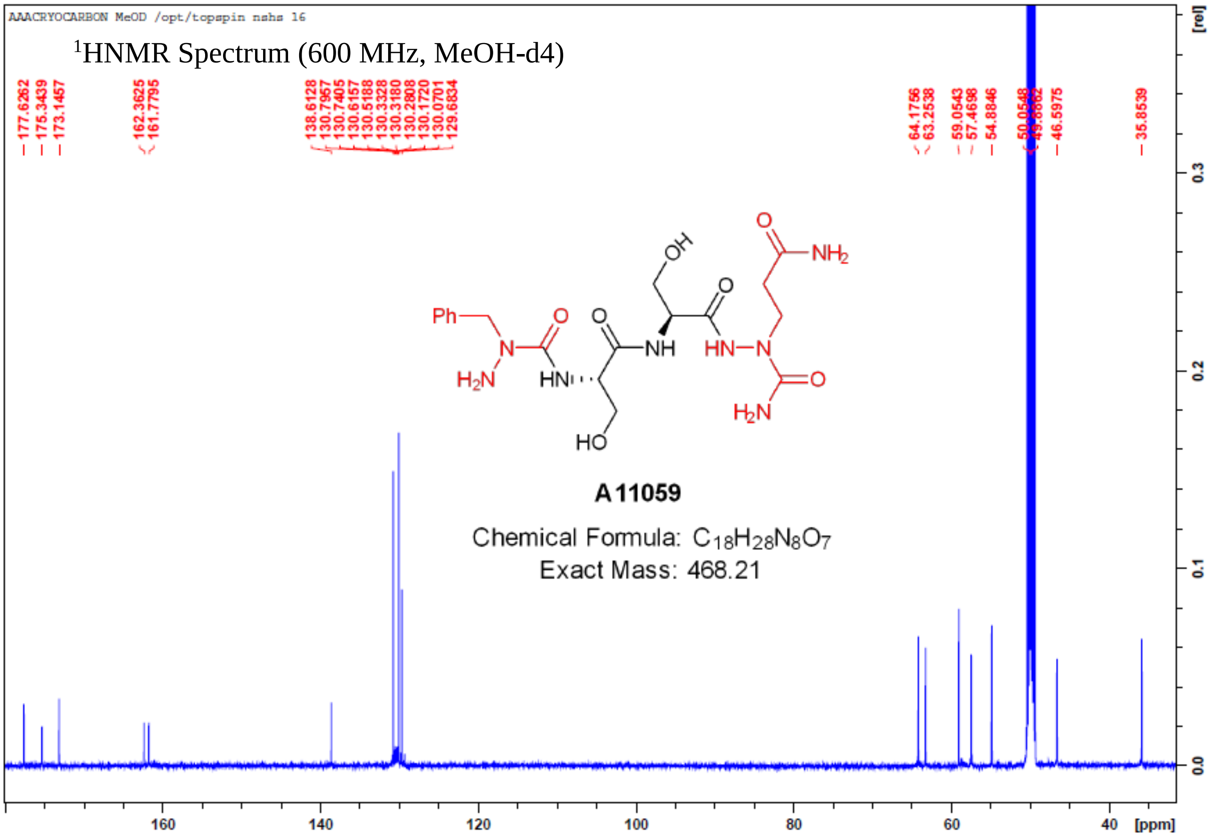

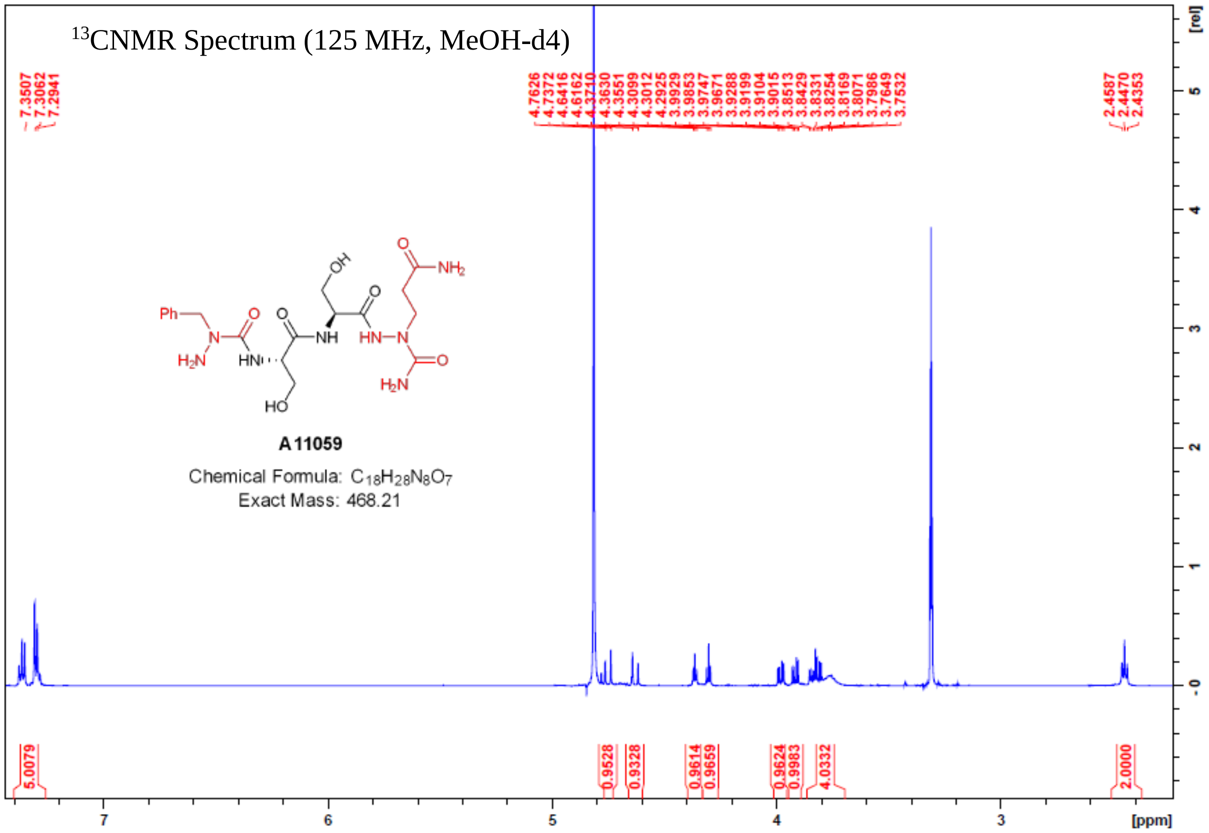

Supplementary Figure 50. NMR spectra of compound 57.

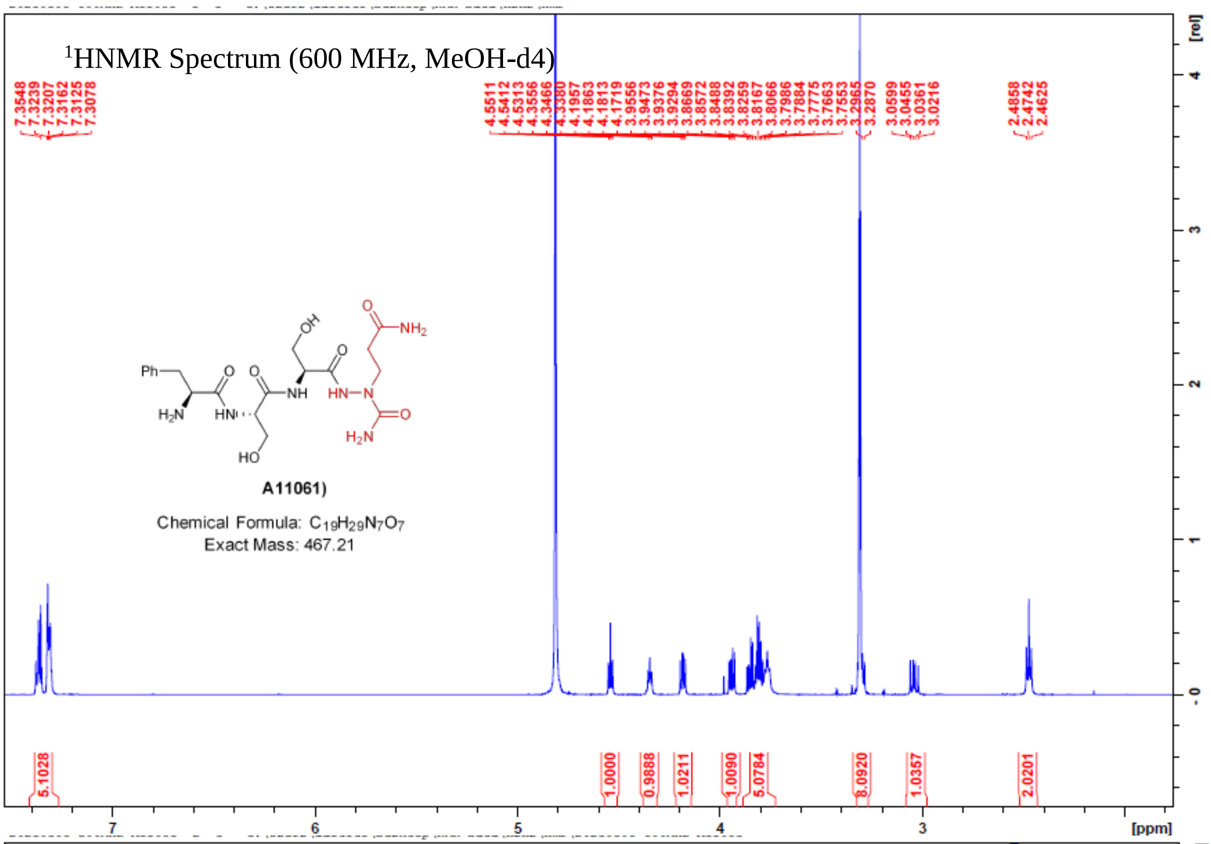

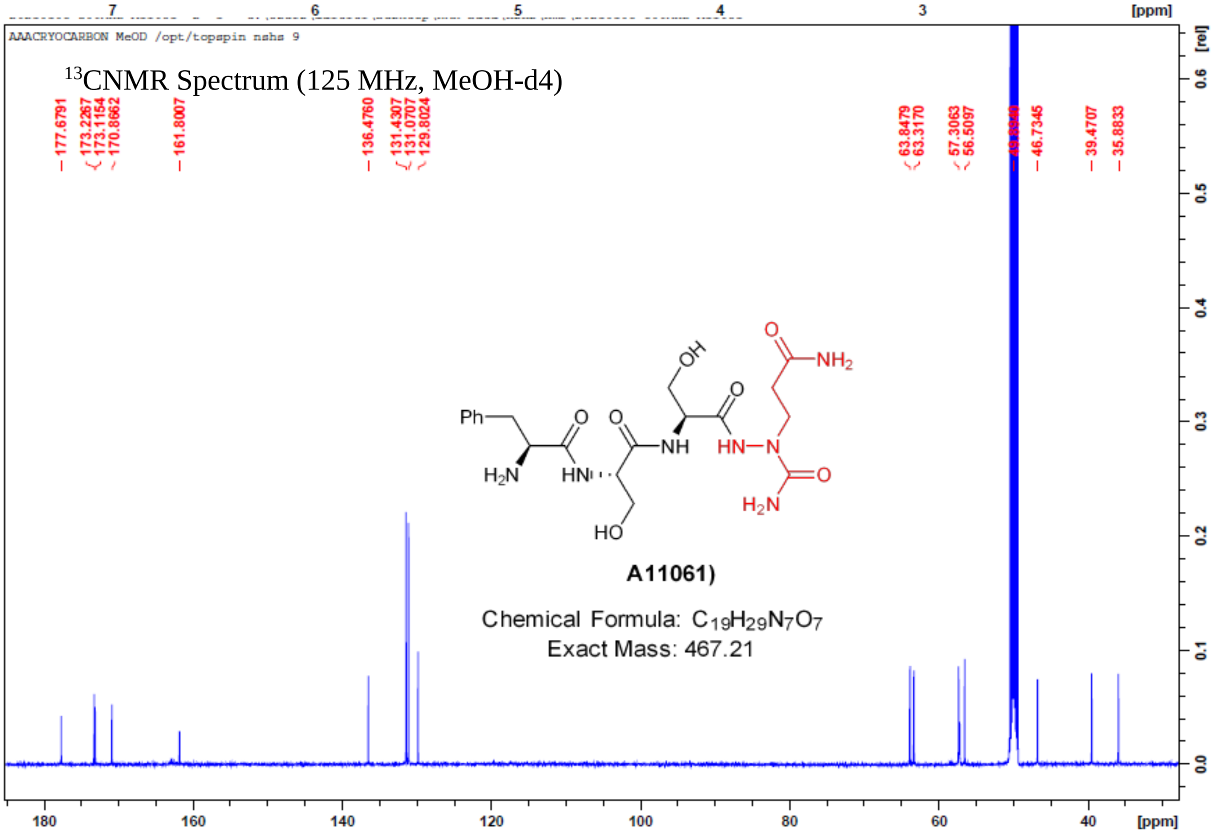

Supplementary Figure 51. NMR spectra of compound 58.

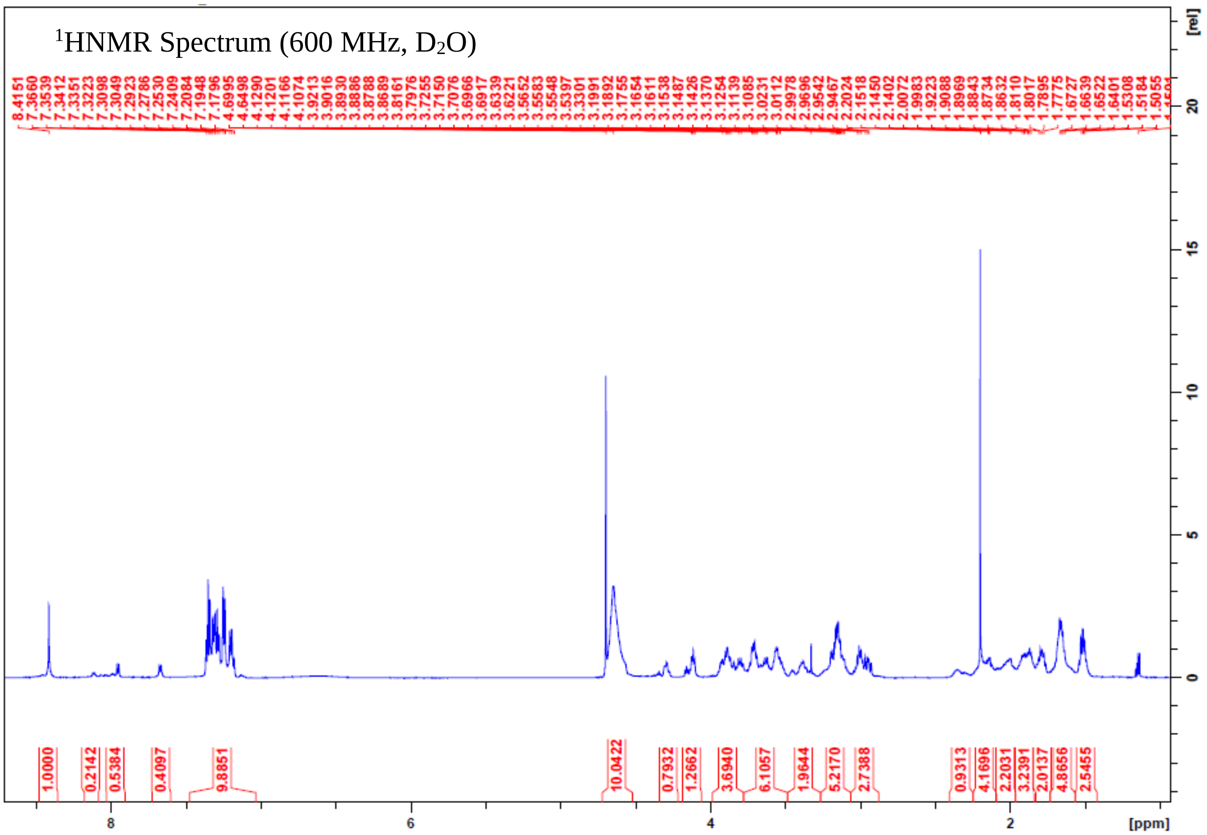

Supplementary Figure 52. NMR spectra of compound 63.

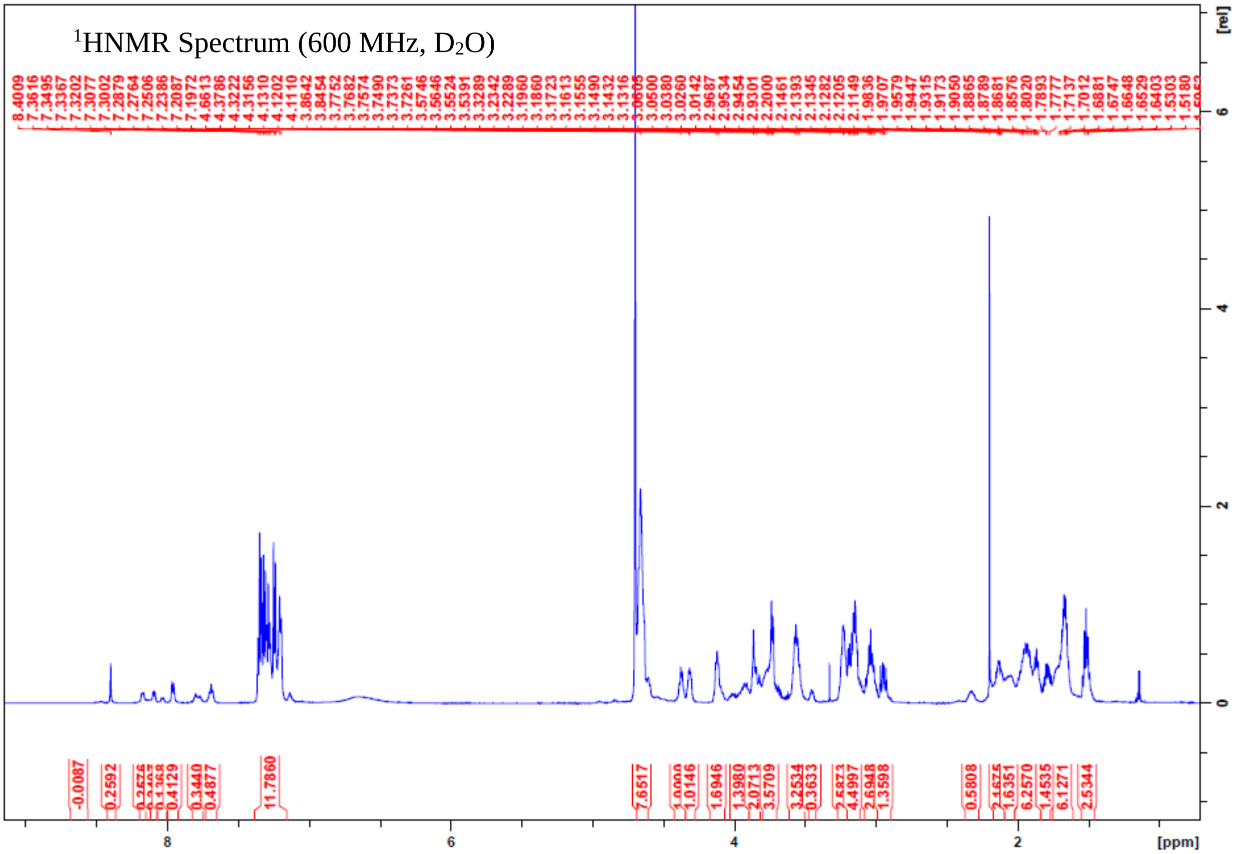

Supplementary Figure 53. NMR spectra of compound 64.

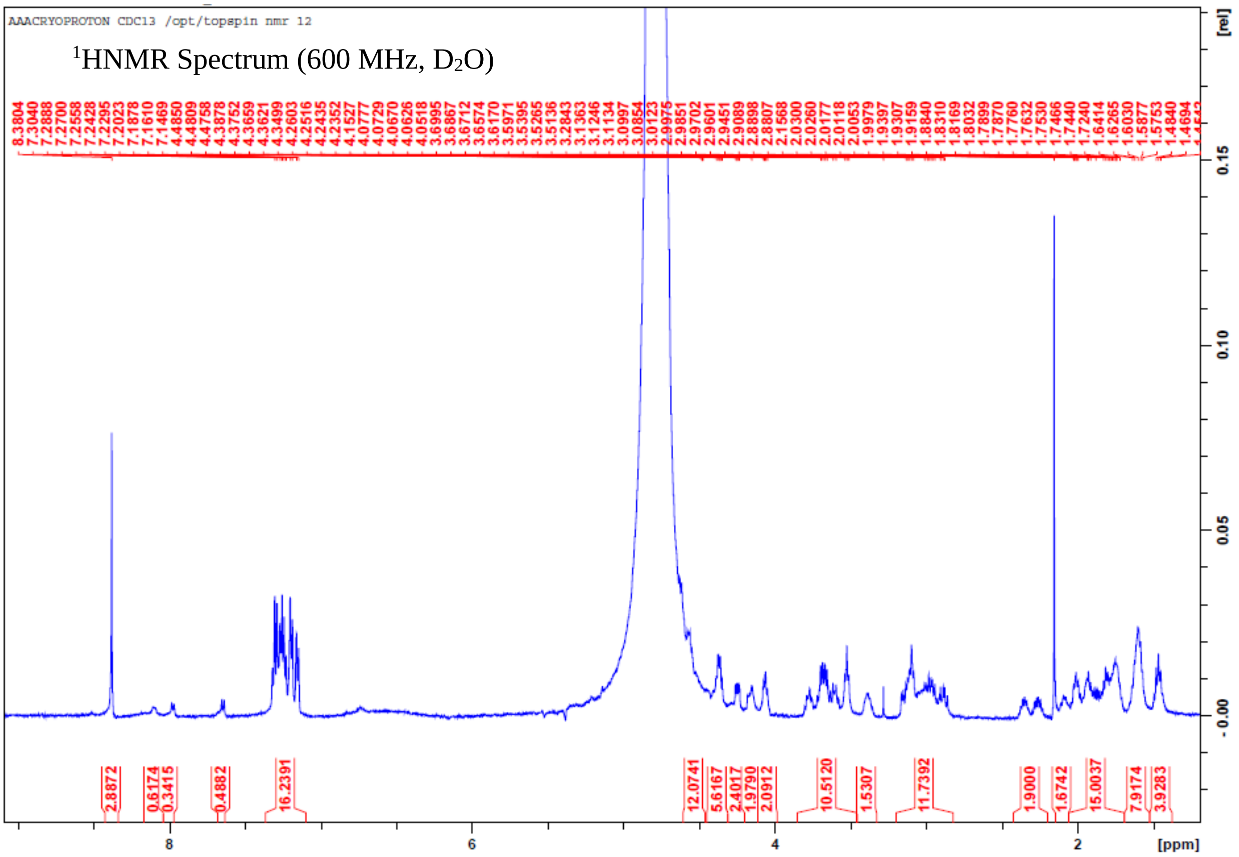

Supplementary Figure 54. NMR spectra of compound 65.

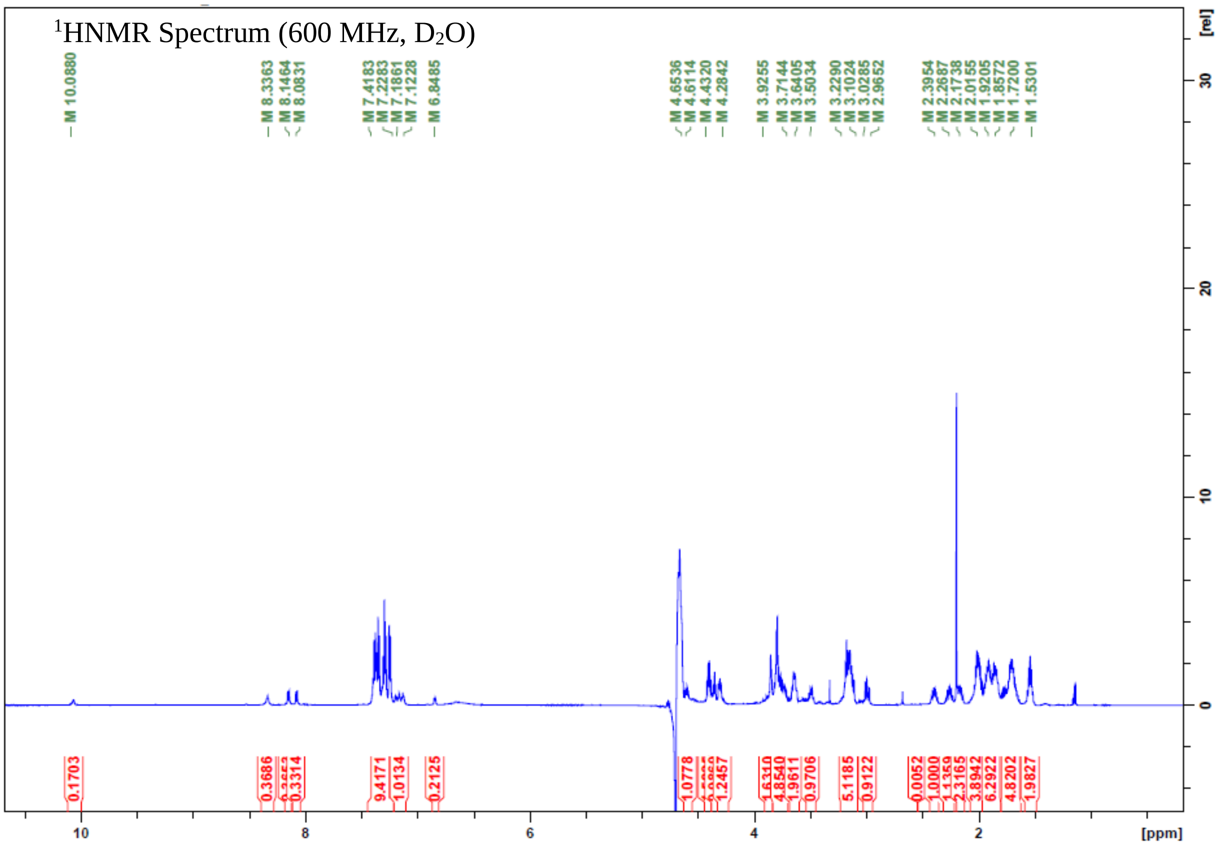

Supplementary Figure 55. NMR spectra of compound 66.

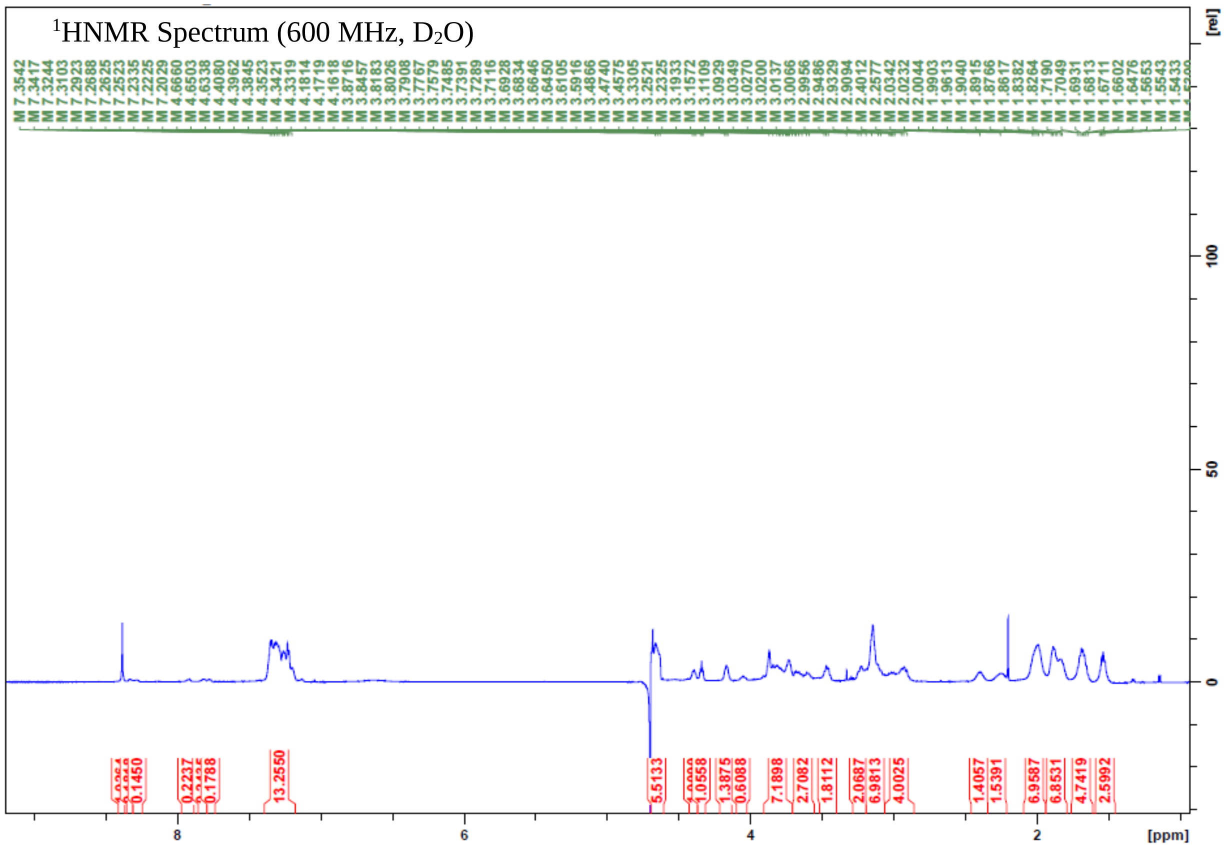

Supplementary Figure 56. NMR spectra of compound 67.

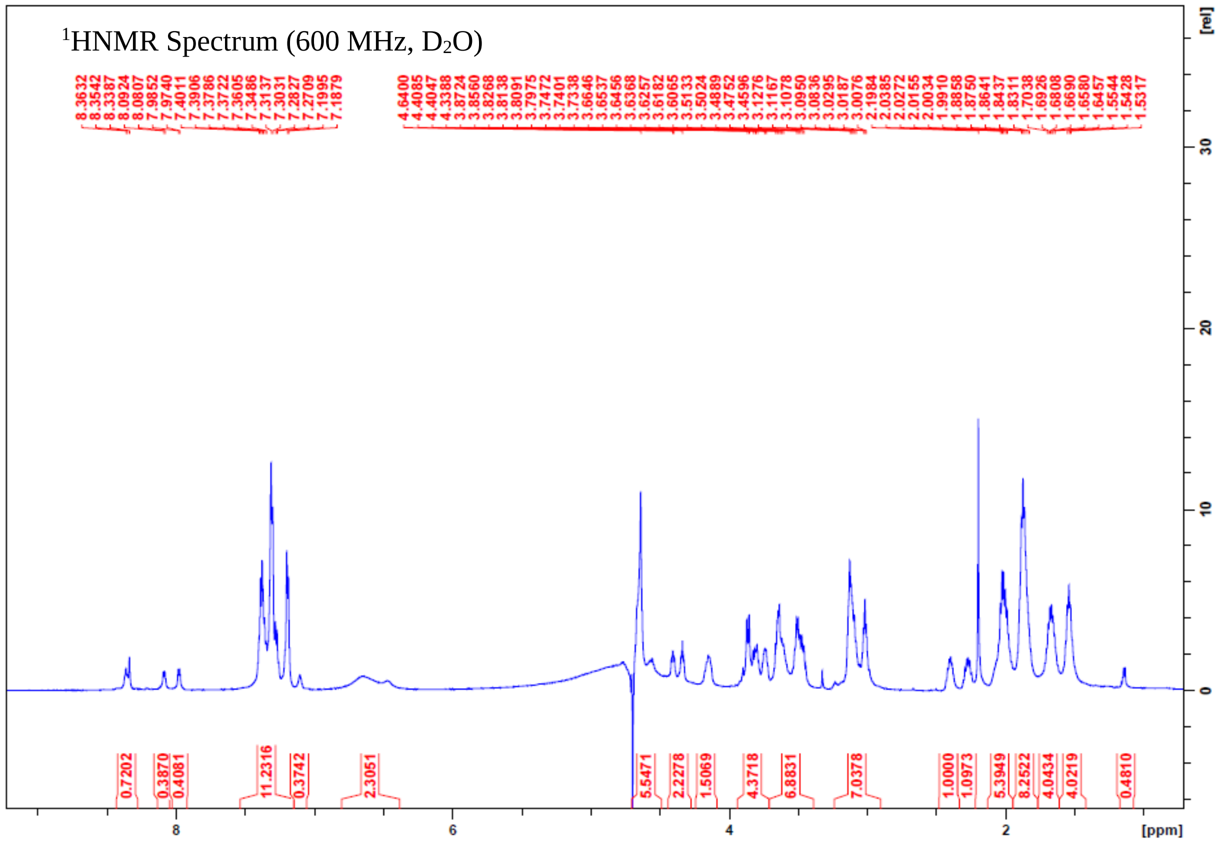

Supplementary Figure 57. NMR spectra of compound 68.

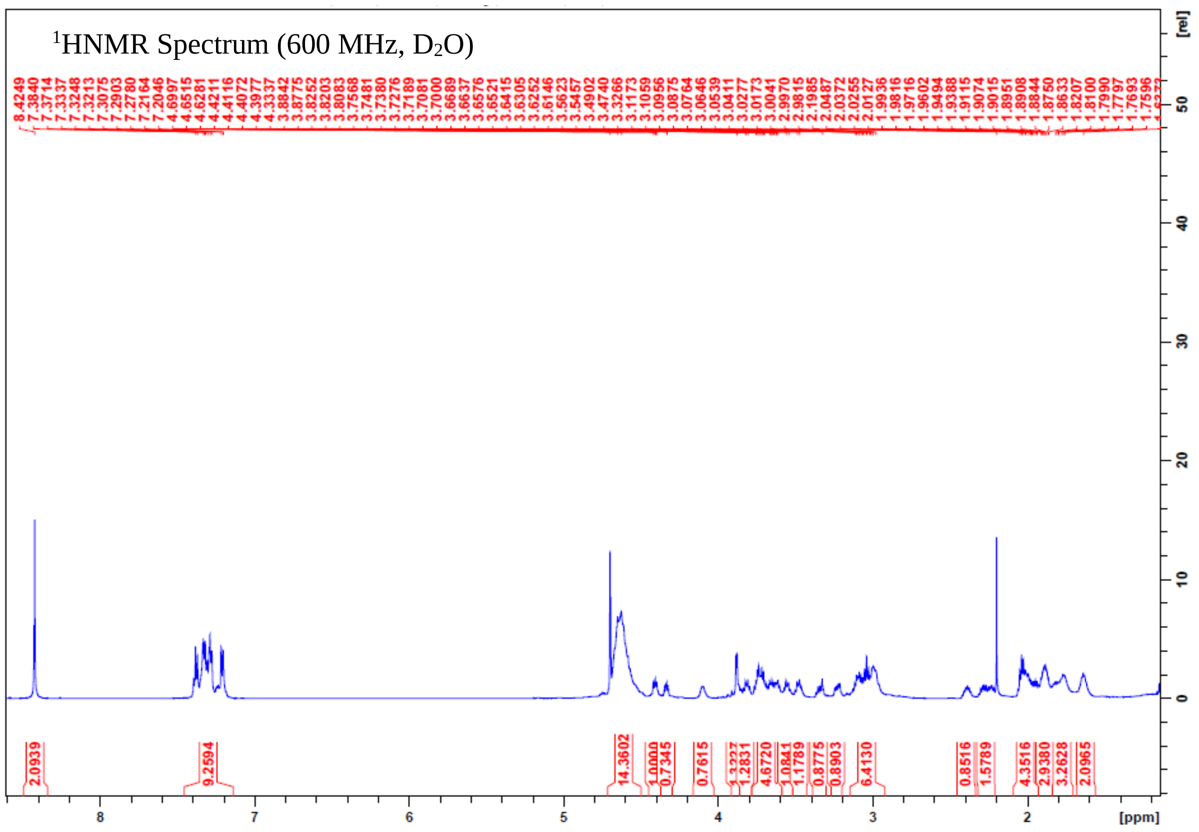

Supplementary Figure 58. NMR spectra of compound 69.

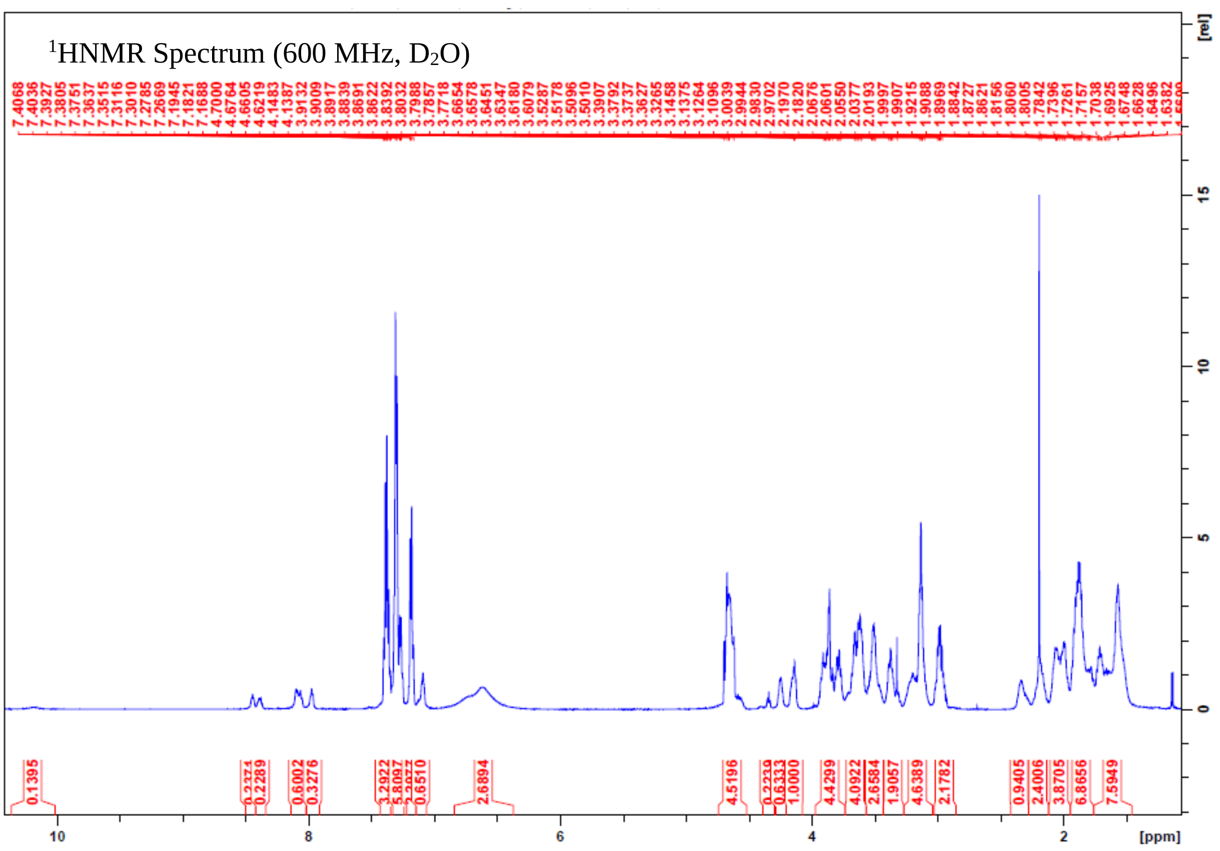

Supplementary Figure 59. NMR spectra of compound 70.

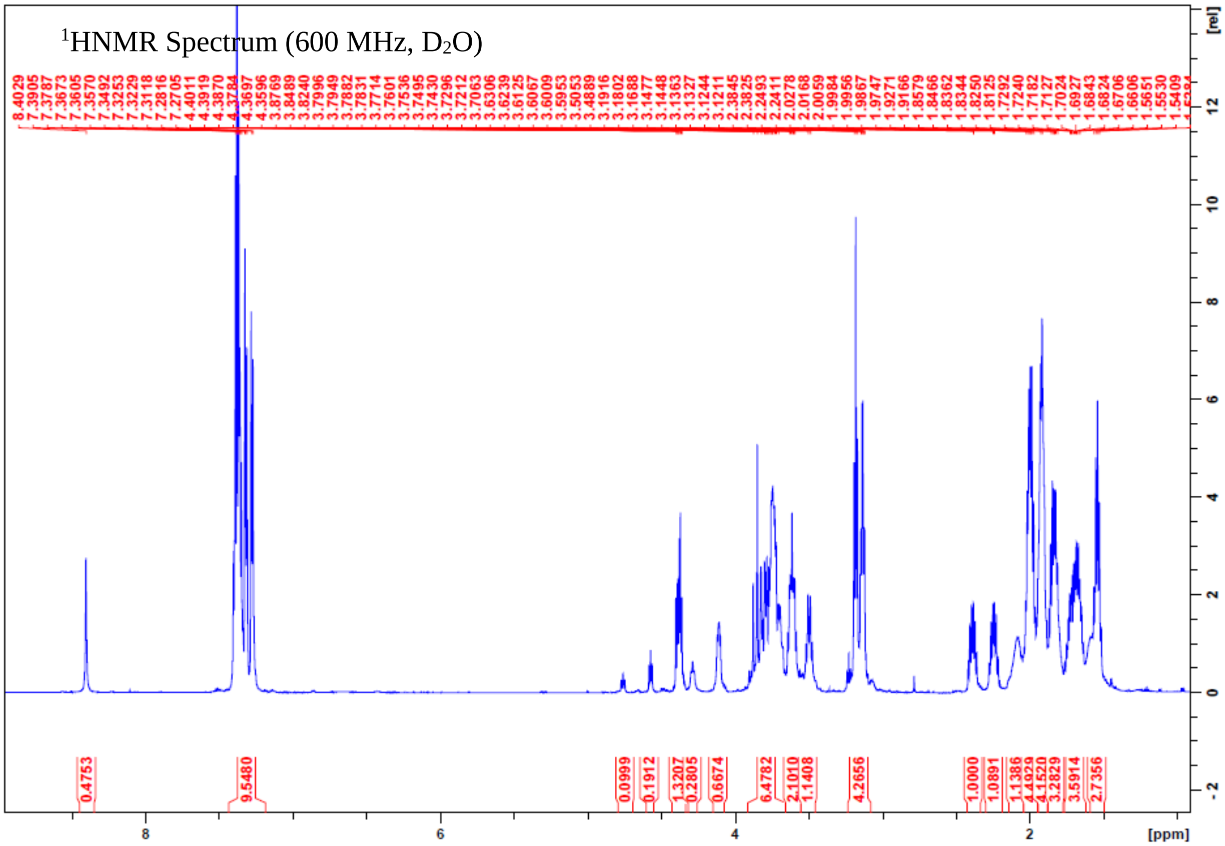

Supplementary Figure 60. NMR spectra of compound 71.

Supplementary References

1. Leiter, E.H. The NOD Mouse: A Model for Insulin-Dependent Diabetes Mellitus. *Curr. Protoc. Immunol.* **24**, 15.19.11-15.19.23 (1997).
2. Hudson, L.K. et al. Emetine Di-HCl Attenuates Type 1 Diabetes Mellitus in Mice. *Mol. Med.* **22**, 585-596 (2016).
3. Jiao, L., Liang, Y., Zhang, Q., Zhang, S. & Xu, J. Catalyst-free, High-yield, and Stereospecific Synthesis of 3-Phenylthio β-Lactam Derivatives. *Synthesis* **2006**, 659-665 (2006).
4. Casarini, M.E., Ghelfi, F., Libertini, E., Pagnoni, U.M. & Parsons, A.F. 1,2-Reduction of α,β-unsaturated hydrazones using dimethylamine–borane/p-toluenesulfonic acid: an easy route to allyl hydrazines. *Tetrahedron* **58**, 7925-7932 (2002).
5. Boeglin, D. & Lubell, W.D. Aza-Amino Acid Scanning of Secondary Structure Suited for Solid-Phase Peptide Synthesis with Fmoc Chemistry and Aza-Amino Acids with Heteroatomic Side Chains. *J. Comb. Chem.* **7**, 864-878 (2005).
6. Fuller, D.R. et al. Conformationally Regulated Peptide Bond Cleavage in Bradykinin. *J. Am. Chem. Soc.* **140**, 9357-9360 (2018).
7. Gibson, C., Goodman, S.L., Hahn, D., Hölzemann, G. & Kessler, H. Novel Solid-Phase Synthesis of Azapeptides and Azapeptoides via Fmoc-Strategy and Its Application in the Synthesis of RGD-Mimetics. *J. Org. Chem.* **64**, 7388-7394 (1999).
8. Freeman, N.S., Tal-Gan, Y., Klein, S., Levitzki, A. & Gilon, C. Microwave-Assisted Solid-Phase Aza-peptide Synthesis: Aza Scan of a PKB/Akt Inhibitor Using Aza-arginine and Aza-proline Precursors. *J. Org. Chem.* **76**, 3078-3085 (2011).
9. Yang, H. et al. MD-2 is required for disulfide HMGB1-dependent TLR4 signaling. *J Exp Med* **212**, 5-14 (2015).
10. Desmet, V.J. Formulation and application of a numerical scoring system for assessing histological activity in asymptomatic chronic active hepatitis [Hepatology 1981;1:431–435]. *J. Hepatol.* **38**, 382-386 (2003).
11. Ghali, M.G.Z. Microsurgical technique for femoral vascular access in the rat. *MethodsX* **4**, 498-507 (2017).
12. Au - Feng, J. et al. Catheterization of the Carotid Artery and Jugular Vein to Perform Hemodynamic Measures, Infusions and Blood Sampling in a Conscious Rat Model. *JoVE*, e51881 (2015).
13. Jean, M., Gera, L., Charest-Morin, X., Marceau, F. & Bachelard, H. In Vivo Effects of Bradykinin B2 Receptor Agonists with Varying Susceptibility to Peptidases. *Front. Pharmacol.* **6** (2016).
14. Sheldrick, G.M. SHELXT - Integrated space-group and crystal-structure determination. *Acta Crystallogr., Sect. A: Found. Adv.* **71**, 3-8 (2015).
15. Sheldrick, G.M.S. An Integrated System for Solving, Refining, and Displaying Crystal Structures from Diffraction Data. *University of Göttingen, Göttingen, Federal Republic of Germany* (1981).
