# Supplementary material for: Thiocarbazate building blocks enable the construction of azapeptides for rapid development of therapeutic candidates
Source: Nat Commun. 2022 Nov 28;13:7127. doi: 10.1038/s41467-022-34712-9 (PMC9705435; doi:10.1038/s41467-022-34712-9)
Supplement: Supplementary file 2 — Reporting Summary [file 41467_2022_34712_MOESM2_ESM.pdf]

nature research

Corresponding author(s): Yousef Al-Abed

Last updated by author(s): Oct 25, 2022

Reporting Summary

Nature Research wishes to improve the reproducibility of the work that we publish. This form provides structure for consistency and transparency in reporting. For further information on Nature Research policies, see our Editorial Policies and the Editorial Policy Checklist.

Statistics

For all statistical analyses, confirm that the following items are present in the figure legend, table legend, main text, or Methods section.

n/a	Confirmed	
☐	☒	The exact sample size (*n*) for each experimental group/condition, given as a discrete number and unit of measurement
☐	☒	A statement on whether measurements were taken from distinct samples or whether the same sample was measured repeatedly
☐	☒	The statistical test(s) used AND whether they are one- or two-sided<br>*Only common tests should be described solely by name; describe more complex techniques in the Methods section.*
☐	☒	A description of all covariates tested
☐	☒	A description of any assumptions or corrections, such as tests of normality and adjustment for multiple comparisons
☐	☒	A full description of the statistical parameters including central tendency (e.g. means) or other basic estimates (e.g. regression coefficient) AND variation (e.g. standard deviation) or associated estimates of uncertainty (e.g. confidence intervals)
☐	☒	For null hypothesis testing, the test statistic (e.g. *F*, *t*, *r*) with confidence intervals, effect sizes, degrees of freedom and *P* value noted<br>*Give P values as exact values whenever suitable.*
☒	☐	For Bayesian analysis, information on the choice of priors and Markov chain Monte Carlo settings
☒	☐	For hierarchical and complex designs, identification of the appropriate level for tests and full reporting of outcomes
☒	☐	Estimates of effect sizes (e.g. Cohen's *d*, Pearson's *r*), indicating how they were calculated

Our web collection on statistics for biologists contains articles on many of the points above.

Software and code

Policy information about availability of computer code

Data collection	For chemistry-Tribute® Peptide synthesizer (Gyros Protein Technologies, Inc. USA), Bruker 600 MHz Avance III Spectrometer, 500 MHz Avance DRX Cryoprobe Spectrometer. Thermo Scientific™ LTQ XL™ linear ion trap mass spectrometer. Waters Prep 150 LC system, Waters Breeze HPLC system, Thermo Scientific™ - Vanquish™ HPLC & UHPLC; For protein binding analysis -Biacore T200 (Cytiva, GE Healthcare, USA), Chirascan™; For structural investigations- circular dichroism spectrometer (Applied Photophysics Ltd, UK); For TNF ELISA experiments (three independent experiments with triplicates per sample), a Perkin Elmer Victor3 microplate reader was used to measure absorbance at 450 nm; For T1D histology experiments, images were captured by Zeiss Axiovert 20-inverted microscope, using the AxioVision V5 software (Zeiss). For bradykinin-PGE2 experiments each ELISA experiment was performed in three independent (or four independent in 1 experiment) replicates to ensure reproducibility using a Dynatech Microplate Reader Model MR 5000 to measure absorbance; For bradykinin experiments, the in-vivo data was collected using LabChart v8 software (ADInstruments, CO, USA).
Data analysis	For chemistry- NMR data was processed in TopSpin 4.0.8 (Bruker); Data of ex vivo blood stability (t1/2) were analyzed using Xcalibur 3.1 (Thermo Scientific, USA); Surface plasmon resonance (SPR) binding assays were analyzed by Biacore T200 Evaluation Software 3.1 (Cytiva, GE Healthcare, USA). The secondary structure of bradykinin azapeptides were determined by CDNN 2.1 (Circular Dichroism analysis using Neural Networks) software and were processed with Prism X (GraphPad Software Inc.); TNF ELISA data were analyzed using Microsoft Excel and GraphPad Prism Version 9.0.1; APAP, T1D data and PGE2 ELISA data were analyzed using GraphPad Prism Version 9.3.1; For bradykinin experiments, in-vivo animal data were analyzed using Matlab 2016a (MathWorks) software, MA, USA.

For manuscripts utilizing custom algorithms or software that are central to the research but not yet described in published literature, software must be made available to editors and reviewers. We strongly encourage code deposition in a community repository (e.g. GitHub). See the Nature Research guidelines for submitting code & software for further information.

Data

Policy information about availability of data

All manuscripts must include a data availability statement. This statement should provide the following information, where applicable:

- Accession codes, unique identifiers, or web links for publicly available datasets
- A list of figures that have associated raw data
- A description of any restrictions on data availability

From manuscript, "All relevant data to the manuscript generated for these studies are included in the article or supplemental information. The X-ray crystallographic coordinates for structures reported in this study have been deposited in the Cambridge Crystallographic Data Centre (CCDC), under deposition numbers CCDC-2195262 and CCDC-2195263."
Raw data from Figures (Figure 2, 3, 4, 5) are provided in supplementary information. Raw data for B2R binding inhibition are provided in a Source Data file.

Field-specific reporting

Please select the one below that is the best fit for your research. If you are not sure, read the appropriate sections before making your selection.

- [x] Life sciences
- [ ] Behavioural & social sciences
- [ ] Ecological, evolutionary & environmental sciences

For a reference copy of the document with all sections, see nature.com/documents/nr-reporting-summary-flat.pdf

Life sciences study design

All studies must disclose on these points even when the disclosure is negative.

Sample size	For ex-vivo blood stabilities (t1/2) means ± SD were obtained from three blood samples, each data point was run in duplicate; sample size was based on previous studies and available resources. Inhibitory effects against HMGB1/MD-2 binding analyzed by SPR were performed at least 3 independent replicates, based on previous studies; The CD spectra were plotted by using the average value of 3 repeats, determined by contract research organization. For in vitro FSSE-TNF experiments, we did not use any statistical tests to choose sample size a priori, but based sample size on previous established studies; For type 1 diabetes experiments, n=7-8 animals per group for blood glucose, body weight and serum levels of insulin measurement; n=2-3 animals, n=13-23 islets per group for insulitis score analysis, group size determination was made based on previous studies; For in vivo APAP experiments, n=5-8 each group for animal studies, total 50-60 C57BL/6 mice; For in vitro bradykinin-PGE2 experiments, based upon pilot studies, 3 independent dose response experiments each having at least 3 replicates (2 experiments had 3 replicates and 1 experiment had 4 replicates) were performed. For in vivo bradykinin experiments, sample size was not calculated for these experiments. We allocated 2-3 rats for testing each compound, and based on the consistent results we obtained in those animals we reason that the sample size is sufficient in our experiments.
Data exclusions	No data were excluded for all studies reported.
Replication	All experimental findings were reliably reproduced as described above for t1/2 in whole blood, SPR binding analyses, and CD analyses. At least 3 independent experiments or repeats were performed; In vitro TNF experiments-Samples are replicated at least an n of 3 for all experiments. All attempts at replication were successful; in vivo type 1 diabetes experiment was performed once and time course indicated reproducible results; in vivo APAP experiments, all experiments were reliably reproduced; in vitro bradykinin-PGE2 experiments -All experimental findings were reliably reproduced as described (3 independent experiments); in vivo-bradykinin experiments-Treatments in rat animal experiments were performed in at least two rats (n indicated in Figure legends).
Randomization	Randomization was not relevant for in vitro experiments including t1/2, SPR, or CD analyses, bradykinin-PGE2 experiments, and TNF release in isolated monocytes (cells were pooled and plated for experiments); For in vivo type 1 diabetes experiments and in vivo APAP experiments-animals for treatment groups were randomly selected by the researcher. For in vivo bradykinin experiments-Animals were initially randomly assigned to each experimental group containing different compounds using Matlab software (2016a).
Blinding	No blinding was used for t1/2 experiments, and SPR due to a single investigator carrying out all planning, labeling, and experiments. In vitro TNF experiments-Investigators were blinded to treatment conditions during analysis; in vivo type 1 diabetes experiments- no blinding was used; In vivo APAP experiments- no blinding was used; In vitro bradykinin-PGE2 experiments were blinded to the investigator; In vivo Bradykinin experiments-The investigator who performed the rat animal experiment was blinded to the treatment conditions. CD analyses analysis were performed by contract research organization which was blinded.

Reporting for specific materials, systems and methods

We require information from authors about some types of materials, experimental systems and methods used in many studies. Here, indicate whether each material, system or method listed is relevant to your study. If you are not sure if a list item applies to your research, read the appropriate section before selecting a response.

Materials & experimental systems

n/a	Involved in the study
x	Antibodies
	x Eukaryotic cell lines
x	Palaeontology and archaeology
	x Animals and other organisms
x	Human research participants
x	Clinical data
x	Dual use research of concern

Methods

n/a	Involved in the study
x	ChIP-seq
x	Flow cytometry
x	MRI-based neuroimaging

Eukaryotic cell lines

Policy information about cell lines

Cell line source(s)	For PGE2 experiments: 3T3 Swiss Albino cell line (ATCC CCL-92);
Authentication	For PGE2 experiments: Cell line was purchased from ATCC, and came with a detailed product report and authentication. Pilot studies showed this that the 3T3 cell line received from ATCC responded to bradykinin by release of PGE2, as reported in the literature. The cell line was not further authenticated by authors.
Mycoplasma contamination	For PGE2 exps: We did not run mycoplasma test on 3T3 cells; But, the product report from ATCC, from whom we purchased the 3T3 cell line, stated that the line had tested negative for mycoplasma; We used 3T3 cells at early passage numbers (passage < 8).
Commonly misidentified lines (See ICLAC register)	For PGE2 exps: the 3T3 line used in the study is not listed as a misidentified cell line in the ICLAC register.

Animals and other organisms

Policy information about studies involving animals; ARRIVE guidelines recommended for reporting animal research

Laboratory animals	For in vitro TNF experiments -Male C56BL/6J (6-8 weeks old) from Jackson Labs were used to obtain monocytes (protocol #2009-048); For in vivo T1D, Male C57Bl/6J mice (6-8 weeks old) for diabetes study were acquired from Taconic Biosciences (Rensselaer, NY) (protocol #2011-035); For in vivo APAP experiments, Male C57BL/6J mice (8-12 weeks old) were obtained from the Jackson Laboratory (Bar Harbor, ME) and acclimated for at least 1 week before conducting experiments (protoocl #2013-021) ; For bradykinin exps- male sprague-Dawley rats, age 8-10 weeks from Charles River Labs were used (protocol #2018-011). Mice and rats were given free access to water and standard rodent chow and were acclimated to their environment for at least 1 week before experimentation. All animal procedures were approved by the Feinstein Institutes for Medical Research Institutional Animal Care and Use Committee (IACUC, protocol 2009-048, 2011-035, 2013-021, 2018-011). Mice and rats were housed in the Center for Comparative Physiology of the Feinstein Institutes for Medical Research under standard temperature and humidity, 12 hour light and dark cycle conditions.
Wild animals	The study did not involve wild animals.
Field-collected samples	The study did not involve samples collected from the field.
Ethics oversight	The Institutional Animal Care and Use Committee at Feinstein Institutes for Medical Research approved all animal experiments (protocol #'s 2009-048, 2011-035, 2013-021, 2018-011), details are within manuscript.

Note that full information on the approval of the study protocol must also be provided in the manuscript.
