# Supplementary material for: Thiocarbazate building blocks enable the construction of azapeptides for rapid development of therapeutic candidates
Source: Nat Commun. 2022 Nov 28;13:7127. doi: 10.1038/s41467-022-34712-9 (PMC9705435; doi:10.1038/s41467-022-34712-9)
Supplement: Supplementary file 3 — Source Data [file 41467_2022_34712_MOESM3_ESM.zip › Supplementary Figure 7_report.pdf]

FINAL REPORT

eurofins | DISCOVERY

In Vitro Pharmacology: Human B2(h) (agonist radioligand) Receptor Binding Assay

Study of Several Compounds

STUDY ID: FR095-0018916

STUDY NUMBER
100053815

August 19, 2020

CONFIDENTIAL

1. STUDY REFERENCES

**Study title**	*In Vitro* **Pharmacology: Human B2(h) (agonist radioligand) Receptor Binding Assay**<br>**Study of Several Compounds**	
**Study number**	**100053815**	**FINAL REPORT**<br>**August 19, 2020**
**Study ID**	**FR095-0018916**	
**Experimental period**	August 11, 2020 - August 18, 2020	

2. PERSONS INVOLVED IN THE STUDY

**Technical contact**	**Eurofins Cerep**<br>Le Bois l'Evêque<br>B.P. 30001<br>86 600 Celle l'Evescault<br>France	**Annie OTTO-BRUC,Ph.D.**<br>Principal Scientist, Pharmacology<br>AnnieOtto-Bruc@eurofins.com
**Study sponsor**	**Feinstein Institutes for Medical Research**<br>350 Community Drive<br>Manhasset, NY 11030<br>U.S.A.	**Sonya VANPATTEN**

3. APPROVAL

Head of laboratory statement

This study was conducted according to the procedures described in this report.

Eurofins Cerep
Le Bois l'Evêque
B.P. 30001
86 600 Celle l'Evescault
France

Sophie SEIGNEURIN,Mrs
Operations Director
SophieSeigneurin@eurofins.com

Signature

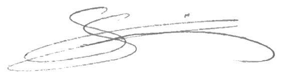

Quality assurance statement

This study was inspected by Eurofins Cerep Quality Control Unit, the results and methods presented in this report accurately reflect the methods used and the data collected for this study.

Eurofins Cerep
Le Bois l'Evêque
B.P. 30001
86 600 Celle l'Evescault
France

Eric BOUCHET
Quality Site Group Leader
EricBouchet@eurofins.com

Signature

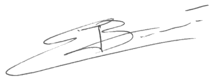

4. TABLE OF CONTENTS

1. STUDY REFERENCES 2

2. PERSONS INVOLVED IN THE STUDY 2

3. APPROVAL 3

4. TABLE OF CONTENTS 4

5. SUMMARY 5

5.1. Study Design 5

5.2. Measurements 5

5.3. Results 5

5.3.1. Compound FI-U 5

5.3.2. Compound FI-2V 5

5.3.3. Compound FI-8G 5

6. COMPOUNDS 6

6.1. Test Compounds 6

6.2. Reference Compounds 6

7. RESULTS 7

7.1. *In Vitro* Pharmacology: Binding Assays 7

7.1.1. IC_{50} Determination: Test Compound Results 7

7.1.2. Reference Compound Results 8

8. RESULTS INTERPRETATION GUIDE 9

9. MATERIALS AND METHODS 10

9.1. Experimental Conditions 10

9.1.1. *In Vitro* Pharmacology: Binding Assays 10

9.2. Analysis and expression of results 11

9.2.1. *In Vitro* Pharmacology: Binding Assays 11

10. BIBLIOGRAPHY 12

5. SUMMARY

The purpose of this study was to test 3 compounds in the B2 (h) (agonist radioligand) assay.

5.1. Study Design

3 compounds were tested at several concentrations for IC_{50} or EC_{50} determination .

5.2. Measurements

Compound binding was calculated as a % inhibition of the binding of a radioactively labeled ligand specific for each target.

5.3. Results

Results showing an inhibition or stimulation higher than 50% are considered to represent significant effects of the test compounds.

Such effects were observed here and are listed in the following tables.

Only the calculable IC_{50} and EC_{50} are reported below.

5.3.1. Compound FI-U

Assay	IC_{50}	K_i	K_B	EC_{50}	nH
B_2 *(h)* (agonist radioligand)	1.1E-08 M	5.6E-09 M			1

5.3.2. Compound FI-2V

Assay	IC_{50}	K_i	K_B	EC_{50}	nH
B_2 *(h)* (agonist radioligand)	7.9E-09 M	4.1E-09 M			1

5.3.3. Compound FI-8G

Assay	IC_{50}	K_i	K_B	EC_{50}	nH
B_2 *(h)* (agonist radioligand)	8.8E-09 M	4.5E-09 M			1.3

6. COMPOUNDS

6.1. Test Compounds

Manufacturer: **Feinstein Institutes for Medical Research**

Client Compound ID	Compound ID	Reference Number	Batch Number	FW	MW	Purity	Received Form	Stock solution	Flag
FI-U	100053815-1	-	-	1061.0	-	100.0	Powder	1.E-02 M H2O	-
FI-2V	100053815-2	-	-	1060.0	-	100.0	Powder	1.E-02 M H2O	-
FI-8G	100053815-3	-	-	1060.0	-	100.0	Powder	1.E-02 M H2O	-

FW: Formula Weight - MW: Molecular Weight

6.2. Reference Compounds

In each experiment and if applicable, the respective reference compound was tested concurrently with the test compounds, and the data were compared with historical values determined at Eurofins. The experiment was accepted in accordance with Eurofins validation Standard Operating Procedure.

7. RESULTS

7.1. *In Vitro* Pharmacology: Binding Assays

7.1.1. IC_{50} Determination: Test Compound Results

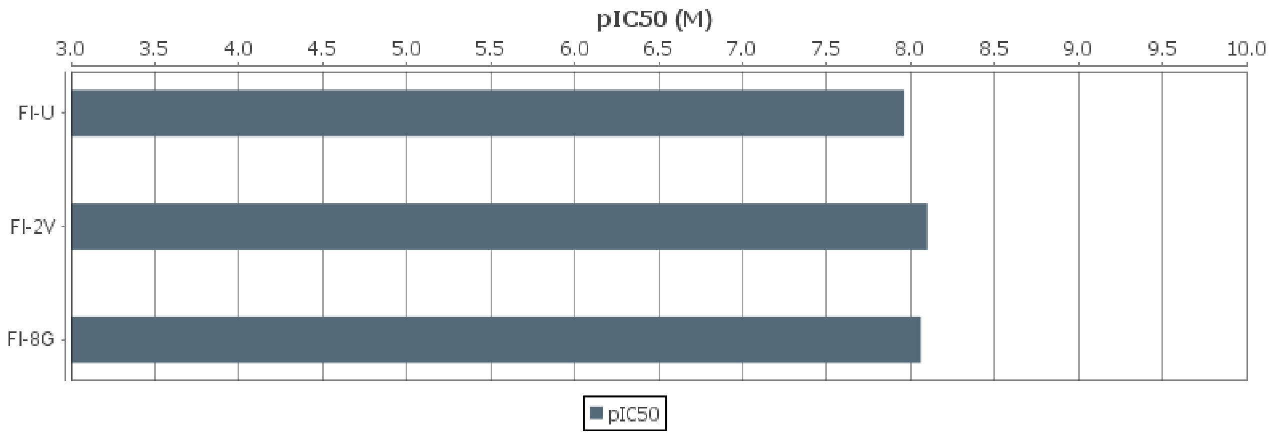

Figure 1. Histogram for B2 (h) (agonist radioligand)

Compound I.D.	Client Compound I.D.	IC_{50}(M)	K_i(M)	nH	Test Concentration	% Inhibition of Control Specific Binding 1st	2nd	Mean
**B_2 *(h)* (agonist radioligand)**								
100053815-1	FI-U	**1.1E-08 M**	5.6E-09 M	1.0	1.0E-09 M	-9.2	8.0	-0.6
					1.0E-08 M	36.3	48.4	42.3
					3.0E-08 M	67.6	73.7	70.6
					1.0E-07 M	84.7	91.4	88.1
					1.0E-06 M	97.1	99.5	98.3

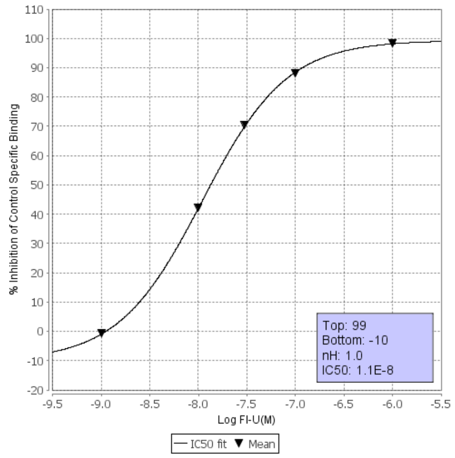

Figure 2. FI-U on B_2 *(h)* (agonist radioligand)

Compound I.D.	Client Compound I.D.	IC_{50}(M)	K_i(M)	nH	Test Concentration	% Inhibition of Control Specific Binding 1st	2nd	Mean
100053815-2	FI-2V	**7.9E-09 M**	4.1E-09 M	1.0	1.0E-09 M	8.0	2.9	5.4
					1.0E-08 M	61.5	66.7	64.1
					3.0E-08 M	72.4	64.6	68.5
					1.0E-07 M	98.0	95.2	96.6
					1.0E-06 M	99.7	100.1	99.9

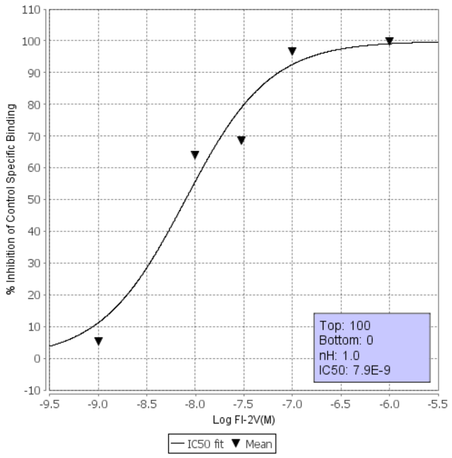

Figure 3. FI-2V on B_2 *(h)*
(agonist radioligand)

Compound I.D.	Client Compound I.D.	IC_{50}(M)	K_i(M)	nH	Test Concentration	% Inhibition of Control Specific Binding 1st	2nd	Mean
100053815-3	FI-8G	**8.8E-09 M**	4.5E-09 M	1.3	1.0E-09 M	0.2	-3.4	-1.6
					1.0E-08 M	58.5	56.4	57.4
					3.0E-08 M	86.3	63.9	75.1
					1.0E-07 M	94.6	95.1	94.9
					1.0E-06 M	98.9	98.6	98.8

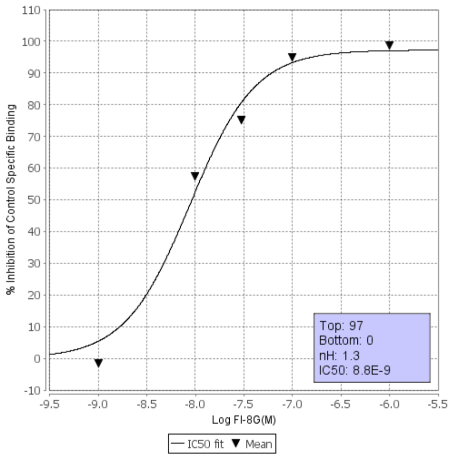

Figure 4. FI-8G on B_2 *(h)*
(agonist radioligand)

7.1.2. Reference Compound Results

Compound I.D.	IC_{50}(M)	K_i(M)	nH
**B_2 *(h)* (agonist radioligand)**			
NPC 567	9.6E-09 M	5.0E-09 M	0.6
NPC 567	1.4E-08 M	7.3E-09 M	0.9

8. RESULTS INTERPRETATION GUIDE

In Vitro Pharmacology

Results showing an inhibition (or stimulation for assays run in basal conditions) higher than 50% are considered to represent significant effects of the test compounds. 50% is the most common cut-off value for further investigation (determination of IC_{50} or EC_{50} values from concentration-response curves) that we would recommend.
Results showing an inhibition (or stimulation) between 25% and 50% are indicative of weak to moderate effects (in most assays, they should be confirmed by further testing as they are within a range where more inter-experimental variability can occur).
Results showing an inhibition (or stimulation) lower than 25% are not considered significant and mostly attributable to variability of the signal around the control level.
Low to moderate negative values have no real meaning and are attributable to variability of the signal around the control level. High negative values (≥ 50%) that are sometimes obtained with high concentrations of test compounds are generally attributable to non-specific effects of the test compounds in the assays. On rare occasion they could suggest an allosteric effect of the test compound.

9. MATERIALS AND METHODS

9.1. Experimental Conditions

Minor variations to the experimental protocol described below may have occurred during the testing, they have no impact on the quality of the results obtained.

9.1.1. *In Vitro* Pharmacology: Binding Assays

Assay	Source	Ligand	Conc.	Kd	Non Specific	Incubation	Detection Method	Bibl.
**Receptors**								
**B_2 *(h)* (agonist radioligand)**	human recombinant (CHO cells)	$[^3H]$bradykinin	0.3 nM	0.32 nM	bradykinin (1 µM)	60 min RT	Scintillation counting	346

9.2. Analysis and expression of results

9.2.1. *In Vitro* Pharmacology: Binding Assays

The results are expressed as a percent of control specific binding

$$\frac{\text{measured specific binding}}{\text{control specific binding}} * 100$$

and as a percent inhibition of control specific binding

$$100-\left(\frac{\text{measured specific binding}}{\text{control specific binding}} * 100\right)$$

obtained in the presence of the test compounds.

The IC_{50} values (concentration causing a half-maximal inhibition of control specific binding) and Hill coefficients (nH) were determined by non-linear regression analysis of the competition curves generated with mean replicate values using Hill equation curve fitting

$$Y=D+\left[\frac{A-D}{1+(C/C_{50})^{nH}}\right]$$

where Y = specific binding, A = left asymptote of the curve, D = right asymptote of the curve, C = compound concentration, C_{50} = IC_{50}, and nH = slope factor. This analysis was performed using software developed at Cerep (Hill software) and validated by comparison with data generated by the commercial software SigmaPlot® 4.0 for Windows® (© 1997 by SPSS Inc.).
The inhibition constants (K_i) were calculated using the Cheng Prusoff equation

$$K_i=\frac{IC_{50}}{(1+L/K_D)}$$

where L = concentration of radioligand in the assay, and K_D = affinity of the radioligand for the receptor. A scatchard plot is used to determine the K_D.

10. BIBLIOGRAPHY

346. Pruneau, D. et al. (1998), *Brit. J. Pharmacol.,* 125*: 365-372.*
